# Biological Properties of the Mucus and Eggs of *Helix aspersa* Müller as a Potential Cosmetic and Pharmaceutical Raw Material: A Preliminary Study

**DOI:** 10.3390/ijms25189958

**Published:** 2024-09-15

**Authors:** Anna Herman, Patrycja Wińska, Małgorzata Białek, Andrzej P. Herman

**Affiliations:** 1 Faculty of Chemistry, Warsaw University of Technology, Noakowskiego 3 Street, 00-664 Warsaw, Poland; patrycja.winska@pw.edu.pl; 2 The Kielanowski Institute of Animal Physiology and Nutrition, Polish Academy of Sciences, Instytucka 3 Street, 05-110 Jabłonna, Poland; m.bialek@ifzz.pl (M.B.); a.herman@ifzz.pl (A.P.H.)

**Keywords:** *Helix aspersa* Müller, snail mucus, snail eggs, antimicrobial activity, antioxidant activity, anticancer activity

## Abstract

In recent years, snail mucus (SM) has become popular as an active ingredient in cosmetic and pharmaceutical products. In turn, snail eggs (SEs) also seem to be a promising active compound, but the biological activities of SEs are significantly less known. Therefore, our preliminary study aimed to compare the biological activities of the SEs and SM of *Helix aspersa* Müller. The metabolomic analysis (LC–MS technique), determination of the antimicrobial activity (agar diffusion test, broth microdilution methods), antioxidant activity (ABTS assay), cytotoxicity assay (MTT), and proapoptotic properties (flow cytometry) of the SEs and SM were evaluated. It was found that the SEs and SM contain 8005 and 7837 compounds, respectively. The SEs showed antibacterial activity against *S. aureus* (MIC 12.5 mg/mL) and *P. aeruginosa* (MIC 3.12 mg/mL). The EC50 estimation of the antioxidant activity is 89.64 mg/mL and above 100 mg/mL for the SEs and SM, respectively. The SEs also inhibited the cell proliferation of cancer cell lines (HCT-116, MCF-7, HT-29) more strongly compared to the SM. The highest proportion of apoptotic cells in HCT-116 was observed. The reach composition of the compounds in the SEs and SM may be crucial for the creation of new cosmetic and pharmaceutical raw materials with different biological activities. However, further extended studies on the biological activities of *H. aspersa*-delivered materials are still necessary.

## 1. Introduction

In recent years, snail mucus (SM) has become popular as an active ingredient in cosmetic and pharmaceutical products [1]. SM from *Helix aspersa* Müller (also known as *Cornu aspersum*) is a rich source of valuable active ingredients, such as elastin, collagen, allantoin, mucopolysaccharides, acetic acid, glycolic acid, lactic acid, hyaluronic acid, glycerol, cyclotrisiloxane hexamethyl, vitamins (A, E, C, B3, B12), and amino acids [2,3,4,5,6,7]. These active compounds have well-established cosmetic properties. The mucus from *H. aspersa* may influence various skin processes in a multidirectional way by promoting fibroblast proliferation [8], stimulating regenerative processes and wound healing [4], protecting against transepidermal water loss (TEWL), restoring the skin’s lipid balance [9], and having antioxidant [10] and antibacterial properties [4]. SM could be a proper source of beauty products through the intake of oral supplementation [11] as well as topical application [9]. Moreover, SM and its active compounds may also be pharmaceutically used. It was found that the topical application of an SM gel can increase angiogenesis during wound healing in rat skin [12]. Also, Zizioli et al. [13] showed that the purified extracts from *H. aspersa* possess pro-angiogenetic activity via maintaining the adequate microvascular and vascular density in the normal and suffering tissues and organs. The glycosaminoglycan isolated from SM markedly accelerated the healing of full-thickness wounds in diabetic mice by alleviating the inflammation and dermal edema, as well as by promoting angiogenesis [14]. The hydrogel with snail glycosaminoglycan and methacrylated gelatin accelerates diabetic wound healing via inflammatory cytokines suppression and macrophage polarization [15]. Moreover, snail *H. aspersa* extract significantly improved the cognitive deficits induced by scopolamine as well as possessed acetylcholinesterase inhibitory activity, moderate antioxidant properties (increased catalase, glutathione peroxidase activities, glutathione and superoxide dismutase activity), the ability to modulate the monoamine (dopamine, noradrenaline, and serotonin) content in the brain structures, and an upregulated expression of brain-derived neurotrophic factor (BDNF) and cAMP response element-binding protein (CREB), causing beneficial effects in an experimental model of Alzheimer’s type dementia in rats [16,17]. The ophthalmic solution (GlicoPro^®^) extracted from snail mucus showed anti-inflammatory and analgesic properties, as well as an induced regeneration and bio-adhesivity in corneal cells, and it may play an important role in the management of dry eye disease [18]. Moreover, 10% GlicoPro^®^ is the main compound in the ophthalmic lubricating solution, Lacricomplex (HelixPharma, a spinoff of the University of Ferrara, Ferrara, Italy). SM extracted from various species of land snails exhibited antimicrobial activity against Gram-positive and Gram-negative bacteria strains [19]. SM has shown therapeutic potential against many cancers, including melanoma [20], breast cancer [21,22], prostate cancer [23], colorectal adenocarcinoma [24], myeloid tumor [25], and erythromyeloid leukemia [26].

However, snail eggs (SE) may also be considered a promising material that can be used in the cosmetics and pharmaceutical industry, but the biological activities of SE are significantly lesser known than in the case of other snail raw materials. Data on the composition of metabolites and the antimicrobial and anticancer activities of SE of *H. aspersa* remain unknown. Therefore, this study aims to compare the biological activities of the SEs and SM of *H. aspersa*.

## 2. Results

### 2.1. The Basic Composition of SM and SEs

The basic composition of the raw materials derived from *H. aspersa* snail is shown in Table 1.

The data show that both the SE samples consisted mostly of crude protein, and the levels are higher than in the SM. In turn, the SM contains much more crude fat than the SEs. The crude ash content is higher in the SEs than in the SM.

### 2.2. The Fatty Acids Profile of SM and SE

The fatty acid profiles of SE and SM of the *H. aspersa* snail are shown in Table 2.

The total amount of saturated fatty acid (SFA) was higher for the freeze-dried SEs (837 ± 326 μg/g) than for the freeze-dried SM (469 ± 80.2 μg/g). The amount of total monounsaturated fatty acid (MUFA) of the freeze-dried SEs and freeze-dried SM were comparable for the SEs (74.3 ± 48.1 μg/g) and SM (64.8 ± 15.6 μg/g). The total amount of polyunsaturated fatty acid (PUFA) in the SM reached a value of 29.7 ± 8.11 μg/g, while the freeze-dried SEs did not contain these fatty acids.

### 2.3. The Amino Acids Content of SM and SE

The amino acid profile of the SEs and SM from the *H. aspersa* snail are shown in Table 3. The amount of individual and total amino acids in the SE samples was higher than in the SM sample. The total quantity of amino acids in the freeze-dried SE samples amounted to 101 ± 0.17 mg/g, while in the freeze-dried SM, it amounted to 15.7 ± 0.14 mg/mL. Asparagine, Arginine, Glycine, Treonine, Tyrosine, Taurine, Methionine, Leucine, and Homocysteine were not present in the SM samples.

### 2.4. Metabolites Identified in Fresh SE, Freeze-Dried SE, and Freeze-Dried SM

Chromatograms of the fresh SE, freeze-dried SE, and freeze-dried SM derived from *H. aspersa* in the water/methanolic/water–methanol/acetonitrile/acetonitrile–water solvents were presented in Figures S1–S30, respectively. Metabolites identified in the fresh SEs, freeze-dried SEs, and freeze-dried SM derived from *H. aspersa* in the water/methanolic/water–methanol/acetonitrile/acetonitrile–water extracts were listed in Tables S1–S15, respectively. Water seems to be the least effective extracting agent, as the fewest compounds were identified in the aqueous extracts of all samples, regardless of the ionization mode (Figures S1, S6, S11, S16, S21 and S26 and Tables S1, S6 and S11). The best solvent for the fresh SEs was methanol (Figure S7), while for the freeze-dried SEs and SM, the mixture of methanol and water (Figures S18 and S28), as evidenced by the largest number of identified compounds (471, 521, and 532, respectively) in the positive ionization mode (Tables S2, S8 and S13). In the negative ionization mode, almost eight times fewer analytes were identified than in the positive ionization mode, regardless of the sample type and solvent used (on average, 51 compounds in the negative ionization mode compared to 405 compounds in the positive ionization mode).

### 2.5. Antimicrobial Activity of SM and SE

The antimicrobial activity of the SM and SEs was determined in the diffusion test (Table 4) and broth microdilution method (Table 5). The SM, even at the highest tested concentration (100 mg/mL), did not inhibit the growth of all tested bacteria, yeast, and mold. The SEs showed antibacterial activity against *S. aureus* and *P. aeruginosa* but did not inhibit *E. coli* and fungi growth.

Moreover, the SEs showed bacteriostatic activity rather than bactericidal activity against *S. aureus* and *P. aeruginosa* in tested concentration. The MIC value for the SEs against *S. aureus* and *P. aeruginosa* is 12.5 mg/mL and 3.12 mg/mL, respectively.

### 2.6. Antioxidant Activity of SM and SE

**Figure 1:**
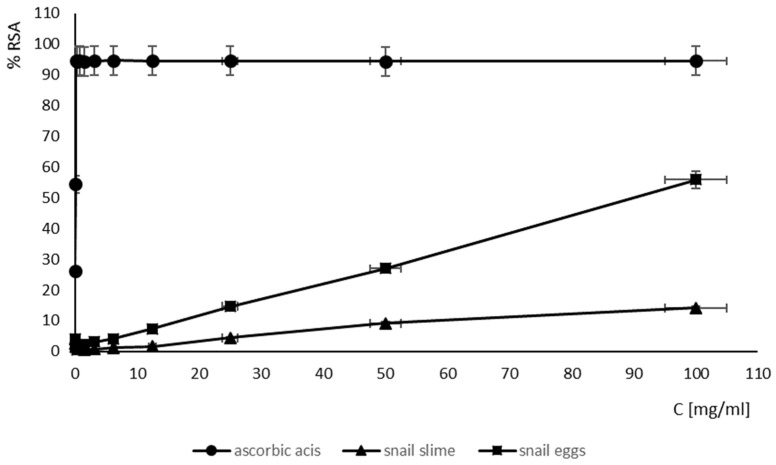
Radical scavenging activity of SM and SEs in ABTS methods.

The tested SM and SEs showed very weak antioxidant activity compared to ascorbic acid (Figure 1).

EC50 estimation of the antioxidant activity of the SM and SEs showed that the SEs had the most effective concentration of antioxidant compounds that resulted in the 50% inhibition of radical formation (EC50 = 89.64 mg/mL), while the SM had the weakest antioxidant activity (EC50 above 100 mg/mL) (Table 6). The antioxidant activity of the SEs was weaker compared to the antioxidant activity of ascorbic acid (EC50 = 0.1 mg/mL).

### 2.7. Effect of SEs and SM on the Viability of MCF-7, HCT-116, HT-29, and Vero Cell Lines

**Figure 2:**
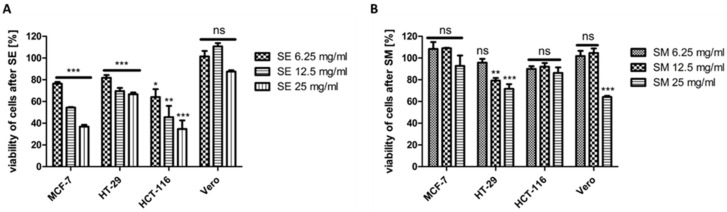
Viability of MCF-7, HT-29, HCT-116, and Vero cell lines after the treatment with SE (**A**) or SM (**B**). After 72 h of incubation, MTT test was performed. The data for the treated cells were analyzed by Dunnett’s multiple comparison test as follows: * *p* < 0.05, ** *p* < 0.01, and *** *p* < 0.001 relative to control served as 100%; ns—not significant.

The effect of SEs (Figure 2A) as well as SM (Figure 2B) on the viability of three cancer cell lines, i.e., MCF-7 (human breast adenocarcinoma), HCT-116 (human colon adenocarcinoma), HT-29 (human colon adenocarcinoma), and one non-cancerous cell line (Vero, Cercopithecus aethiops kidney), was tested. A dose-dependent decrease in the viability of the cancer lines was detected after SE treatment and only partially after SM treatment.

In addition, the results obtained for the SE-treated tumor cell lines were statistically significant. The viability of cells after SE treatment was lower than after SM treatment (Figure 2), with the lowest value of 35% viable cells obtained for HCT-116 at 25 mg/mL SE. A similar result of 37% viable cells was detected for MCF-7 at 25 mg/mL SEs. The least sensitive to SEs of the tested cancer cell lines turned out to be the HT-29 cells, with the viability of 67% at 25 mg/mL SEs. Interestingly, of all the tested cell lines, the non-cancerous Vero cells proved to be the least sensitive to SEs, with the lowest value of 88% viable cells at 25 mg/mL and with non-significant differences in the viability of the control vs. SE-treated cells (Figure 2A). Interestingly, the Vero cells proved to be the most sensitive to SM at 25 mg/mL (statistically significant result) of all the tested cell lines, with 64% viable cells at 25 mg/mL (Figure 2B), whereas the most sensitive cell line to SM among cancer cells was the HT-29 cell line, with 72% of viable cells at 25 mg/mL, as well as statistically significant results for both the 12.5 mg/mL and 25 mg/mL concentrations of SM. The differences in the viability of the control vs. SM-treated cells were not statistically significant for both the MCF-7 and HCT-116 cell lines.

### 2.8. Induction of Apoptosis in MCF-7 and HCT-116 Cells

**Figure 3:**
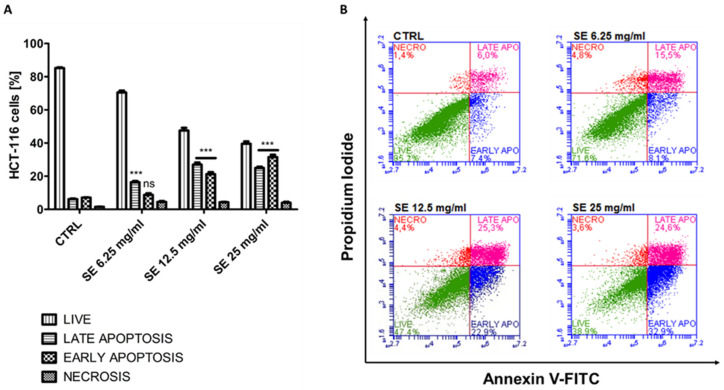
Induction of apoptosis in HCT-116 cells. The data were determined by Accuri C6 Plus flow cytometer (BD Biosciences, San Jose, CA, USA) after 72 h of treatment with SE. Cells were stained with annexin V-FITC and PI (propidium iodide). (**A**) Mean and standard deviation (SD) of necrosis, as well as viable, early, and late apoptosis, as a percentage from three independent experiments each. (**B**) Representative cytograms for control HTC-116 cells (CTRL) and after treatment with SE. The data for early and late apoptotic cells were analyzed by Dunnett’s multiple comparison test as follows: *** *p* < 0.001 relative to control; ns—not significant.

To evaluate the proapoptotic properties of SE, we analyzed the annexin V-binding to phosphatidylserine using flow cytometry. Regarding the MTT-based results, two of the most sensitive cancer cell lines, i.e., MCF-7 as well as HTC-116, were subjected to the study. The SEs were used in three concentrations, i.e., 6.25 mg/mL, 12.5 mg/mL, and 25 mg/mL. The results are shown in Figure 3A,B and Table S16.

**Figure 4:**
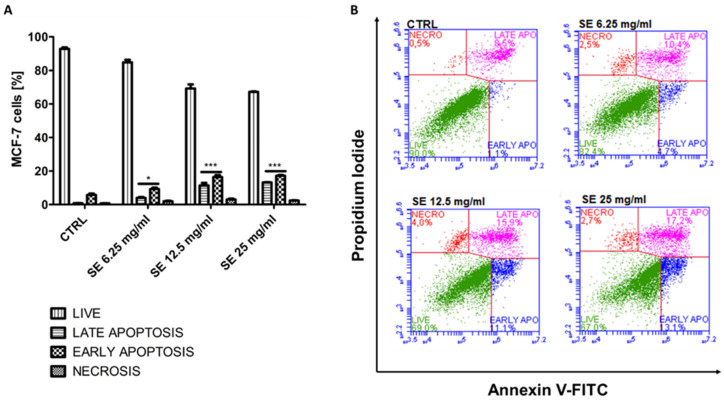
Induction of apoptosis in MCF-7 cells. The data were determined by Accuri C6 Plus flow cytometer after 72 h of treatment with SE. Cells were stained with annexin V-FITC and PI (propidium iodide). (**A**) Mean and standard deviation (SD) of necrosis, as well as viable, early, and late apoptosis, as a percentage from three independent experiments each. (**B**) Representative cytograms for control MCF-7 cells (CTRL) and after treatment with SE. The data for early and late apoptotic cells were analyzed by Dunnett’s multiple comparison test as follows: * *p* < 0.05, and *** *p* < 0.001 relative to control.

The obtained results demonstrate a dose-dependent and statistically significant induction of apoptosis in all the treated cells, with the highest proportion of apoptotic cells close to 56% (sum of early and late apoptotic cells) at 25 mg/mL SEs in the HCT-116 cells (Figure 4A,B). Similarly to HCT-116, the highest number of apoptotic cells in the MCF-7 cells, equal to 30%, was also observed at 25 mg/mL SEs.

The obtained results are in good agreement with the MTT-based results, demonstrating the sensitivity of both the MCF-7 as well as HTC-116 cell lines to SEs. The reason for the weaker induction of apoptosis in the MCF-7 cells than in HCT-116 may be the deficiency of caspase-3 in MCF-7 [27], a crucial effector in a family of caspases, which can directly lead to cellular destruction and death.

## 3. Discussion

The composition of the SE and SM of *H. aspersa* determines the biological activity and their potential use as cosmetic and pharmaceutical raw materials. Several studies have reported data on the composition of SM of *H. aspersa* determined by different methods, including gas chromatography coupled to mass spectrometry (GC-MS) [4,28], nuclear magnetic resonance (NMR) spectroscopy [2], inductively coupled argon plasma atomic emission spectrometry (ICP-OES) [4], tandem mass spectrometry (MALDI-TOF/TOF) [29], high-performance liquid chromatography with fluorescence detection (HPLC-FLD), and ion-exchange chromatography with spectrophotometric detection (IEC-VIS) [30]. Our team used untargeted metabolomic analysis by the LC–MS technique to analyze the composition of phytochemicals in SEs and SM. Each of the above methods has its own peculiarities, and it would be difficult to reliably compare the composition and amount of active compounds in the SEs and SM. Moreover, in the case of SEs, this is impossible because there are no such studies. However, it was found that metabolites determined in the samples with fresh or freeze-dried SEs slightly differ from the composition of metabolites in the SM from *H. aspersa*. Moreover, the freeze-drying process affects the composition and amount of active compounds in the fresh and freeze-dried SE samples. It turned out that the amount of some compounds remained at a comparable level in both the fresh and freeze-dried samples. It was also observed that some compounds after lyophilization were concentrated by more than two times. These differences translate into better antimicrobial and antioxidant activity as well as anticancer activity of the SEs than the SM. The amount of certain saturated fatty acids in the SEs is higher compared to the SM, especially the amount of C12:0 (lauric acid), C14:0 (myristic acid), C16:0 (palmitic acid), C17:0 (margaric acid), and C18:0 (stearic acid). The bactericidal and antifungal properties of fatty acids are well known [31]. It was found that Gram-negative bacteria are affected by short-chain fatty acids of less than six carbons, while long-chain fatty acids are effective against Gram-positive species [32]. Moreover, the most effective saturated fatty acid against Gram-positive bacteria is C12:0. SEs contain three times more C12:0 (24.2 ± 2.2 μg/g) than SM (7.05 ± 1.14 μg/g) and strongly inhibits the growth of *S. aureus*. The higher amount of saturated fatty acids in SEs compared to SM is also associated with the greater antioxidant activity of SEs than SM. It was found that antioxidant activity increases with the increasing chain length of the fatty acids from octanoic acid to myristic acid (C8:0–C14:0) and a decrease thereafter [33]. Moreover, it was found that the saturated medium-chain fatty acid lauric acid triggers anti-proliferative and apoptotic effects in breast cancer cells [34] and colon cancer cells [35,36]. Furthermore, SEs are a rich source of amino acids, especially cysteine which is much higher in SEs (70.3 ± 0.10 mg/mL) compared to MS (7.99 ± 0.06 mg/mL). It was found that L-cysteine showed strong antimicrobial activity against *S. aureus* and acted via damage of the bacterial cell membrane to further cause cell death [37]. Mytimacin-AF, a cysteine-rich antimicrobial peptide isolated from mucus of the snail *Achatina fulica* showed potent antimicrobial activities against *S. aureus* [38]. Some studies have suggested that cysteine-based dipeptides may be potential anticancer agents [39]. Cysteine inhibited proliferation, delayed cell cycle, and induced cell senescence in melanoma but did not induce cell death [40]. The variety and richness of biologically active compounds present in SEs make it a valuable raw material for use in the cosmetic or pharmaceutical industry. However, further extended studies on the selection of compounds responsible for the biological properties of ES are still needed.

The data received by our team are the first results verifying the antimicrobial activities of the SEs of *H. aspersa*. The SEs showed antibacterial activity against *S. aureus* and *P. aeruginosa*, while the SM did not inhibit the growth of all the tested bacteria and fungi. Some research found that the raw SM of *H. aspersa* showed antibacterial activity [4,41]. Aouji et al. [4] found that the raw mucus of *H. aspersa* snail in the concentration range of 25–100 μg/mL possesses antibacterial activity against *P. aeruginosa*, *E. coli*, *Listeria monocytogenes*, and *S. aureus*. The raw mucus from *H. aspersa* and the isolated mucus proteins (37.4 kDa, 17.5 kDa, 18.6 kDa) demonstrated antimicrobial activity against *P. aeruginosa* [41,42]. The mucus fraction (MW > 20 kDa) from the *Cornu aspersum* snail showed promising antimicrobial activity against *Bacillus cereus*, *Propionibacterium acnes*, *Salmonella enterica*, *Enterococcus faecalis*, and *Enterococcus faecium* comparable to vancomycin [43], while peptide fractions (below 3 kDa to 50 kDa) showed such activity against *P. aureofaciens*, *E. coli*, *Clostridium perfringens* and *Brevibacillus laterosporus* [44]. Moreover, the antimicrobial activity of the peptide fractions from SM seems to be related to the rich content of glycine, proline, tryptophan, and valine in peptide sequences. Also, glycine-rich peptides from *Cornu aspersum* showed antibacterial activity against *Escherichia coli NBIMCC 8785* [45]. The antimicrobial activities of *H. aspersa* mucus focus mainly on raw SM and peptide fractions isolated from SM. None of the above-described studies were performed on freeze-dried SM, so it is difficult to compare the results obtained by us with the literature data. However, the antimicrobial activities of raw SM obtained from certain species other than *H. aspersa* are well documented. The SM from the giant African land snails (*Achatina marginata*, *A. achatina*, and *A. fulica*) showed antibacterial activity against *P. aeruginosa*, *S. aureus*, *E. coli*, *S. typhi*, *B. subtilis*, and *K. pnuemoniae* [46]. The mucus from the *A. marginata saturalis*, *A. marginata ovum*, and *A. fulica* snails inhibited the growth of *Staphylococcus* sp., *Pseudomonas* sp., and *Streptococcus* sp. isolated from wound infection [47]. SM from *A. fulica* showed antimicrobial activity against *Mycobacterium tuberculosis* isolated from tuberculosis patients [48]. Mucus from *Eremina desertorum* snails has significantly higher inhibitory activity against *P. aeruginosa*, *E. coli*, *K. pneumonia*, *C. albicans*, *A. niger*, *Trichoderma harzianum*, and *Rhizopus stolonifera* compared to the mucus of *H. aspersa* [28]. The SM from *A. maginata* with lincomycin combination (1:1) improves the synergistic antimicrobial effect in the treatment of acute pneumonia caused by *Streptococcus pneumoniae* isolates [49]. The antimicrobial peptides isolated from the hemolymph of *Helix lucorum* snail showed antibacterial activity against *S. aureus*, *S. epidermidis,* and *E. coli* [50]. Also, proline-rich antimicrobial peptides isolated from the hemolymph of *Rapana venosa* snails showed strong antimicrobial activities against *S. aureus* and *Klebsiella pneumonia* [51]. Furthermore, the mucus from *H. aspersa* appears to have weaker antimicrobial activities compared to other species of land snails [28].

The SEs and SM of *H. aspersa* showed antioxidant activity. Moreover, the antioxidant activity of the SEs is stronger compared to the SM of *H. aspersa*. Matusiewicz et al. [30,52] also showed that both the SEs and SM of *H. aspersa* possess antioxidant activity. Higher antioxidant activity was observed for the lyophilized SEs of *Helix maxima* than the lyophilized SEs of *H. aspersa*. It was also found that low molecular weight fractions of *Cornu aspersum* mucus counteract the formation of reactive free radicals better than total SM [10]. The beneficial effect of these mucus fractions on eukaryotic organisms resulted from reducing the levels of intracellular oxidative damage and increasing the antioxidant capacity of the cell [43].

The obtained MTT-based results indicate that SE demonstrates higher cytotoxicity against HCT-116, HT-29, and MCF-7 cancer cell lines than SM; however, the particular sensitivity of the tested cells is different and seems to be dependent upon the cell line. Only one literature data have reported the anticancer activity of SEs of *H. aspersa* [30]. It was found that the water extract of *H. aspersa* eggs showed a statistically significant reduction in the viability of Caco-2 colon cancer cells after 24 h incubation compared to the control cells treated with water. During this time, the SEs caused damage to the cell membranes, the induction of apoptosis, and the reduction in necrosis of Caco-2 cells. In turn, the mucus of *H. aspersa* increased the viability of the Caco-2 cells [52]. The mucus of the *H. aspersa* snail also showed cytotoxicity activity against breast cancer cells Hs578T via inducing necrosis and downregulating BcL2 expression and NF-κB [21], while the mucus fractions (>50 kDa) isolated from *H. aspersa* exhibited a slight anti-proliferative effect on breast cancer lines (MCF-7 and MDA-MB-231) [53]. Furthermore, the combination of the mucus fractions with cisplatin or tamoxifen resulted in significant reductions in the half-effective dose, indicating possible synergistic effects which were more pronounced with the fraction having Mw > 20 kDa.

Some research has found that hemocyanin (copper-containing glycoproteins) isolated from *H. aspersa* showed anticancer activity. The hemocyanin subunit (βc-HaH) exhibits cytotoxic activity on the A549 and H1299 cells (lung cancers), MDA and MCF-7 cells (breast cancers), and HeLa cells (cervical cancer) [54]. The mucus and α-subunit of hemocyanin from the snail *H. aspersa* have a higher antiproliferative activity against the HT-29 human colorectal carcinoma cell line compared to hemocyanins isolated from marine snails *Rapana venosa* and garden snails *Helix lucorum* [24]. Cytomorphological analysis revealed that the observed antitumor effects were associated with the induction of apoptosis in the tumor cells. The anticancer activity of hemocyanins isolated from *H. aspersa*, *Helix lucorum*, and *Rapana venosa* was examined in the Graffi myeloid tumor model [25]. The results of in vitro studies indicated that the tested hemocyanins induced significant antiproliferative and apoptogenic effects, while the in vivo investigations demonstrated that these bioactive compounds exert distinct immunostimulating and antitumor effects without any histopathological signs of toxicity.

Some SM from certain species other than *H. aspersa* have shown anticancer activity. SM from *Achatina fulica* significantly decreased the proliferation and viability of human breast cancer MDA-MB-231 TNBC (Triple-negative breast cancers) cells with relatively lower cytotoxicity to normal breast epithelial cells and enhanced their response to chemotherapy [55]. The mucus fractions (F2 and F5) separated from the *Achatina fulica* showed cytotoxicity against the breast cancer cell line (MCF-7) and normal epithelium cell line (Vero) [22]. The hemolymph of the desert snail *Helix desertorum* led to a decrease in the Ehrlich ascetic carcinoma tumor volume and an increase in the percentage of the apoptotic cells, arresting the cancer cell cycle [56]. Moreover, treatment with hemolymph improved the hematological and biochemical parameters of tumor-bearing mice.

Our study showed that the garden snail *H. aspersa* is a rich source of biologically active natural substances that might be an important source of cosmetic and pharmaceutical raw materials. The biological activities of *H. aspersa* mucus are relatively extensively described. However, the literature data regarding the biological properties of SEs are still very poor. We found that the SEs showed stronger antimicrobial and antioxidant activities compared to the SM of *H. aspersa*. The SEs also inhibited cell proliferation of cancer cell lines more strongly compared to the SM. The stronger antimicrobial, antioxidant, and anticancer properties of the SEs compared to SM are due to the difference in the composition of biologically active compounds in the SEs and SM. It seems that SEs act as a storehouse of nutrients for the young snail, so their composition may be richer in biologically active ingredients compared to SM. Among them may be a very promising active compound with biological activities desirable for the cosmetic and pharmaceutical industries. However, further extended studies on the biological activities of *H. aspersa*-delivered materials are still necessary.

## 4. Materials and Methods

### 4.1. SM and SEs from H. aspersa

The SM (freeze-dried) and SEs (fresh and freeze-dried) of the organic snail *H. aspersa* were kindly donated by Posada^®^ Organic Snail Farm (Posada, Poland).

### 4.2. Microorganisms

*Pseudomonas aeruginosa* ATCC 9027, *Escherichia coli* ATCC 8739, *Staphylococcus aureus* ATCC 6538, *Candida albicans* ATCC 10231 and *Aspergillus brasiliensis* ATCC 16404 were purchased from the American Type Culture Collection (ATCC, Manassas, VA, USA). The microorganisms were activated through double passaging: bacteria on TSA medium (Trypticase Soy Agar; BioMerieux, Marcy l’Etoile, France) (37 °C, 24 h), yeast on SDA medium (Sabouraud Dextrose Agar; BioMerieux, Marcy l’Etoile France) (30 °C, 48 h), and mold on SDA medium (BioMerieux, Marcy l’Etoile, France) (30 °C, 5 days).

### 4.3. Cell Cultures

Vero cells (Cercopithecus aethiops kidney; ATCC no. CCL-8), HCT-116 (human colon adenocarcinoma; ATCC no. CCL-247), HT-29 (human colon adenocarcinoma; ATCC no. HTB-38), and MCF-7 (human breast adenocarcinoma; ATCC no. HTB-22) were purchased from the American Type Culture Collection (ATCC, Manassas, VA, USA). HCT-116 and HT-29 cells were cultured in McCoy’s 5A, MCF-7 cells were cultured in DMEM (Sigma-Aldrich Chemical Company, St. Louis, MO, USA), whereas the Vero cells were cultured in Minimum Essential Medium Eagle (Sigma-Aldrich Chemical Company, St. Louis, MO, USA). All the cell lines were supplemented with 10% fetal bovine serum (FBS, Sigma-Aldrich Chemical Company, St. Louis, MO, USA), 2 mM L-glutamine, and antibiotics (100 U/mL penicillin, 100 µg/mL streptomycin) (Sigma-Aldrich Chemical Company, St. Louis, MO, USA). Cells were grown in 75 cm^2^ cell culture flasks (Sarstedt, Nümbrecht, Germany) in a humidified atmosphere of CO_2_/air (5%/95%) at 37 °C. All the experiments were performed in exponentially growing cultures.

Lyophilizates of the SEs as well as the SM were prepared in MilliQ water in the concentration of 100 mg/mL by suspension and dissolution, then they were centrifuged at 16,000 RCF/10 min and, subsequently, they were filtered with sterile filters (0.22 µM).

Cisplatin (Sigma-Aldrich) was used as a reference compound (Figure S31 and Tables S17 and S18). The stock solution of cisplatin (20 mM) was prepared in DMF, and the final concentration of DMF in the assays did not exceed 0.5%.

### 4.4. Chemicals

All organic solvents used for extraction (methanol, acetonitrile) and purchased from Avantor Performance Materials (Gliwice, Poland) were of HPLC super gradient grade. Ultrapure water was obtained from PURITE laboratory water purification system (SUEZ, Water Technologies, and Solutions, Bristol, UK). Organic solvents and reagents (acetonitrile, formic acid, ammonium formate) used for chromatographic analyses, purchased from JT Baker, were of LC–MS grade.

### 4.5. Analysis of the Basic Composition of SM and SEs

The basic chemical compositions of the SM and SEs were determined according to AOAC methods [57].

### 4.6. Analysis of Fatty Acids Profile of SM and SEs

Samples (~50 mg) of eggs and mucus were homogenized and saponificated using KOH water and methanol solutions, and then the analytes were extracted by hexane and methylene chloride and methylated, as previously described [58]. Fatty acids methyl esters (FAME) were identified and quantified on a SHIMADZU GC-MS-QP2010 Plus EI (Shimadzu, Kyoto, Japan) equipped with a BPX70 fused silica capillary column (120 m × 0.25 mm i.d. × 0.25 μm film thickness) (SHIM-POL, Warsaw, Poland) and quadruple mass selective detector (Model 5973N) by comparison with FAME standards (Sigma, St. Louis, MO, USA) and NIST 2007 reference mass spectra library (National Institute of Standard and Technology, Gaithersburg, MD, USA). Helium (He), as the carrier gas, operated at a constant pressure (223.4 kPa) and flow rate of 1 mL/min. Injector and detector temperatures were 200 °C and 240 °C, respectively. The FAME profiles in 1 μL samples at the split ratio of 10:1 were determined using the column temperature gradient program described previously [59].

### 4.7. Analysis of Amino Acids Content of SM and SEs

The content of protein amino acids was analyzed in the SEs and SM according to a previously published method [60]. Briefly, the biological samples (approx. 100 mg) were subjected to acid hydrolysis in 50 mL of 6 M HCl at the temperature of 104 ± 2 °C for approx. 12 h in sealed ampoules. After cooling, the hydrolysates were filtered, transferred to a volumetric flask (100 mL), and then filled with distilled water up to the mark. From the solution thus obtained, 10 mL of solution were taken twice and evaporated on an evaporator to remove the aqueous HCl solution. Each residue in the flask was rinsed three times with distilled water. Finally, after a two-fold evaporation, the residue obtained was dissolved in 1 mL of 0.4 M borate buffer (pH 9.8) and then derivatized.

Derivatization of the amino acids in all the samples (after hydrolysis) was performed using 1 mL of the reaction mixture of O-phthalic dialdehyde (OPA, Sigma Aldrich, St Louis, MO, USA) and ethanethiol (ESH, Aldrich, Warsaw, Poland) and 10–20 μL 1 M NaOH (alkaline pH 9–10 required). The reaction mixture was prepared for a minimum of 2 h before use by dissolving 75 mg of OPA in 4.5 mL of methanol and 0.5 mL of borate buffer at pH 9.8 in the presence of 70 µL of ESH. Every two or three days, 10 µL ESH was added to the mixture to regenerate ‘reagent strength’. The reaction mixture was protected from light and stored at low temperature (−18 °C) for no longer than two weeks.

An Alliance separation module (model 2690, Waters, Milford, MA, USA) with Waters 996 photodiode array detector (PAD) was used for the gradient elution systems. An autosampler was thermostated at ~5 °C. The OPA derivatives were monitored at λ = 274 nm using a PAD detector operated in a UV range from 190 to 400 nm, with a spectral resolution of 1.2 nm and a measurement frequency of 1 spectrum per second. The analytical column used was a Nova Pack (4 μm, 250 × 4.6 mm I.D., Waters) in conjunction with a guard Nova Pak column (10 × 6 mm I.D., Waters) containing RP phase C18 (30–40 μm) operating at 55 °C. The maximal system pressure was 36.3 ± 0.2 MPa. The injection volume was 10 μL. Amino acids were identified by comparing their retention times with those obtained for a standard (Amino Acid Standard H, Thermo Scientific Pierce, Rockford, IL, USA, Lot. AF40688).

### 4.8. Untargeted Metabolomic Analysis by LC–MS Technique

The applied extraction procedure was based on the earlier published research concerning the analysis of fish skin mucus [61]. Briefly, the assayed samples (approx. 1 mg of freeze-dried and 13 mg of fresh) were placed in Eppendorf tubes, aliquots of 1 mL of each solvent (water, methanol, acetonitrile, methanol/water (1:1, *v*/*v*), acetonitrile/water (1:1, *v*/*v*)) was added, mixed for 1 min and then centrifuged at 4 °C for 15 min at 12,300 rpm (~14,000 g). Next, the solution was filtered through a syringe filter (0.2 μm, PP—polypropylene, Whatman) into an HPLC glass vial and then subjected to chromatographic analysis.

Analyses were performed using an Infinity II1290 UHPLC Agilent system (Agilent, San Jose, CA, USA). The ZORBAX Rapid Resolution HD Eclipse Plus C18 column (2.1 × 50 mm, 1.8 µm, Agilent Technologies, San Jose, CA, USA) was maintained at 40 °C and operated at a flow rate of 0.5 mL/min. A 2 μL sample injection was used with an elution gradient of solvent A (H_2_O/ACN, 95/5 (*v*/*v*)) and solvent B (ACN/H_2_O, 95/5 (*v*/*v*)), each containing 0.1% formic acid and 5 mM ammonium formate. The applied linear gradient was as follows: 0 min at 100% A, 11 min at 100% B, and 15 min at 100% A. The total time of analysis was 20 min. Before injection of the next sample, the column was rinsed and re-equilibrated at 100% solvent A. A liquid chromatograph was coupled with the Agilent 6546 Q-TOF mass spectrometer (Agilent, San Jose, CA, USA) via Agilent JetStream Dual ESI ionization source, operating in positive and negative modes, under the following conditions: heater temperature: 250 °C; sheath gas temperature: 350 °C; sheath gas flow: 11 L/min; and drying gas flow: 8 L/min; nebulizer: 45 psi. The instrument was tuned in high-resolution mode up to 1700 *m*/*z* mass range. Data were collected in MS Scan mode in the range of 50–1200 *m*/*z* with an acquisition rate of 2 spectra per second for both positive and negative ionization. The collected data were recorded in centroid mode.

To perform data mining MassHunter Qualitative Analysis software (Version 10) was used in the Compound Discovery mode using the Molecular Feature Extraction (MFE) algorithm. A Metlin Metabolites AM PCDL database with a library was used for compound identification.

### 4.9. Determination of the Antimicrobial Activity of SM and SEs—Agar Diffusion Method

The antibacterial and antifungal activity of the lyophilized SM and SEs were evaluated by using the methods described by EUCAST [62]. The lyophilized SM and SEs (100 mg) were resuspended in 1 mL MilliQ water and then centrifuged at 16,000 RCF for 10 min at 21 °C. The obtained supernatants were used for further studies. Several colonies of overnight cultures for bacteria, 48 h for yeast, and 5 days for fungi were suspended in saline to obtain a density equal to 0.5 McFarland turbidity standard (approximate cell density of 1.5 × 10^8^ CFU/mL for bacteria, and 1.5 × 10^6^ CFU/mL for fungi). Suspensions of microorganisms were spread over the TSA and SDA agar plates (BioMerieux, Marcy l’Etoile, France), respectively, using sterile cotton swabs. Then, 10 µL lyophilized SM and SEs (100 mg/mL) were placed on the agar surface. Ofloxacin (5 μg) (BTL, Poland) and nystatin (100 IU) (BTL, Łódź, Poland) were used as controls. All bacterial plates were incubated at 37 °C for 24 h and fungal plates at 25 °C for 48 h (*C. albicans*) and 5 days (*A. brasiliensis*), respectively. The diameter of the zone of inhibition was measured in mm. All tests were conducted in triplicate and data from the experiments were calculated as mean ± SD.

### 4.10. Determination of Minimum Inhibition Concentration (MIC), Minimum Bactericidal Concentration (MBC), and Minimum Fungicidal Concentration (MFC) of SM and SE by Broth Microdilution Methods

The MIC, MBC, and MFC of the lyophilized SM and SEs were evaluated using the broth microdilution methods described by EUCAST [63,64,65]. Several colonies of overnight cultures for bacteria, 48 h for yeast, and 5 days for mold were suspended in saline to obtain a density equal to 0.5 McFarland turbidity standard and then suspended in medium broth (Muller–Hinton for bacteria, Sabouraud for fungi) to 10^5^ CFU/mL. Lyophilized SM and SEs were resuspended in medium broth in a concentration range of 0.78–100 mg/mL by twofold dilution in a 96-well microplate. Novobiocin [1 mg/mL] (Oxoid, Basingstoke, Hampshire, UK) and fluconazol [1 mg/mL] (Oxoid, Basingstoke, Hampshire, UK) were positive controls. The sterility control (only medium broth) and microorganisms growth control (bacteria/yeast in appropriate medium broth) were also prepared. All plates were incubated at 37 °C for 24 h. The MIC is the lowest concentration of the antimicrobial agent that completely inhibits the growth of the organism as detected by the unaided eye. For all the wells with clear broth, 100 μL of the sample was spread over the TSA and SDA agar plates. All bacterial plates were incubated at 37 °C for 24 h and fungal plates at 25 °C for 48 h (*C. albicans*) and 5 days (*A. brasiliensis*), respectively. The MBC and MFC demonstrate the lowest concentration of the antimicrobial agent that results in bactericidal/fungicidal activity (no growth observed on plates).

### 4.11. Determination of the Antioxidant Activity of Lyophilized SM and SEs—ABTS Assay

The ABTS (2,2′-azinobis-(3-ethylbenzthiazolin-6-sulfonic acid)) assay measures the ability of an antioxidant to stabilize the ABTS radical cation (ABTS^+^). The ABTS^+^ is a green-blue chromophore produced through a reaction between 7 mM aqueous ABTS and 2.45 mM potassium persulfate (K_2_S_2_O_8_) mixed in a ratio of 1:1 and incubated overnight (12–16 h) at room temperature in the dark. The ABTS solution was diluted to obtain 0.7 absorbance at 734 nm. In a 96-well microtiter plate, a 10 μL sample of lyophilized SM or SEs, or ascorbic acid as a positive control (in the concentration range of 0.78–100 mg/mL obtained by two times dilution in water), was mixed with 190 μL of ABTS radical solution and incubated for 30 min at room temperature in the dark. The blank well was obtained by mixing 10 μL water and 190 μL of ABTS radical solution. For each test, three replicates were performed. The samples and blank absorbance were determined at 734 nm wavelengths in a microplate reader (Synergy H4, BioTek, Agilent, Palo Alto, CA, USA).

Radical scavenging activity (RSA) of fermented vegetable extract was calculated using the following formula:

% RSA =((Abs blank − Abs sample)/Abs blank) × 100

where:

% RSA—percent of radical scavenging activity.

Abs blank—absorbance of ABTS.

Abs sample—absorbance of a sample.

The antiradical activity of lyophilized SM, SE, or ascorbic acid was also expressed as EC50 (mg/mL), the concentration of sample required to cause 50% ABTS inhibition. The EC50 value was calculated by a graphical method as the effective concentration that results in a 50% inhibition of radical formation.

### 4.12. 3-(4,5-Dimethylthiazol-2-yl)-2,5-diphenyltetrazolium bromide (MTT)—Based Viability Assay

The cytotoxicity of the SEs and SM were examined by MTT assay (3-(4,5-dimethylthiazol-2-yl)-2,5-diphenyltetrazolium bromide). The MCF-7, HT-29, HCT-116, and Vero cells were seeded at the density of 6 × 104 cells/mL in a 96-well micro-culture plate at 100 µ/well and incubated at 37 °C and 5% CO_2_ overnight. The following day, stock solutions of SEs or SM were added in the final following concentrations: 6.25 mg/mL, 12.5 mg/mL, and 25 mg/mL, whereas the control cells were treated with the equivalent volume of sterile MilliQ water. Cisplatin was used in the 2-fold serial dilution range of 1.56 µM to 100 µM. After incubation with the tested extracts/cisplatin, the MTT test was performed as described previously [66]. Optical densities were measured at 570 nm using a BioTek microplate reader (Winooski, VT, USA). All measurements were carried out in a minimum of three replicates.

### 4.13. Detection of Apoptosis by Annexin V/Propidium Iodide (PI) Labeling

The MCF-7 and HCT-116 cells were seeded in 12-well plates (Sarstedt, Nümbrecht, Germany) at the density of 7 × 10^4^/mL in 1 mL/well. After 18 h of incubation, SEs/cisplatin was added. SEs were used in the final following concentrations: 6.25 mg/mL, 12.5 mg/mL, and 25 mg/mL, whereas the control cells were treated with the equivalent volume of sterile MilliQ water. Cisplatin was used in 3 concentrations corresponding to 0.5 × IC50, IC50, and 2 × IC50 (Figure S31). After exposure to the examined SE/cisplatin, the cells were collected and subjected to the procedure described by us earlier [67]. Flow cytometric measurements were performed within 1 h after labeling. Viable, necrotic, early, and late apoptotic cells were detected by flow cytometry using BD Accuri C6 Plus flow cytometer and analyzed using the BD Accuri™ C6 Plus Analysis Software for PC or Mac, cat. no 664022 (BD Biosciences, San Jose, CA, USA).

### 4.14. Statistical Analysis

Results are represented as mean ± s.e.m. of at least three independent experiments. Statistical analysis was performed using GraphPad Prism 5.0 software (GraphPad Software Inc., San Diego, CA, USA). Significance was determined using a one-way ANOVA analysis. The statistical significance of the differences was indicated in the figures with asterisks as follows: * *p* ≤ 0.05, ** *p* ≤ 0.01, and *** *p* ≤ 0.001.

## Figures and Tables

**Table 1:** Basic composition of raw materials derived from organic *H. aspersa* snail.

	Sample	SE (Fresh)	SE (Freeze-Dried)	SM (Freeze-Dried)
Variables				
Nitrogen [%]		0.74 ± 0.06	3.63 ± 0.07	0.29 ± 0.03
Crude protein [%]		4.63 ± 0.38	22.7 ± 0.40	1.81 ± 0.16
Dry matter [%]		15.6 ± 0.28	95.8 ± 0.05	97.4 ± 0.11
Ash [%]		3.60 ± 0.05	25.8 ± 0.04	1.98 ± 0.04
Crude fat [%]		0.23 ± 0.03	0.38 ± 0.02	1.86 ± 0.06
Energy [cal/g]		3758 ± 6.43	4224 ± 3.06	7071 ± 6.66

Legends: SE—snail eggs; SM—snail mucus.

**Table 2:** Fatty acids [μg/g] profile of raw materials derived from organic *H. aspersa* snail.

	Sample	SE (Fresh)	SE (Freeze-Dried)	SM (Freeze-Dried)
Fatty Acid				
ƩFAs		325 ± 68.1	911 ± 272	564 ± 149
C10:0		2.63 ± 1.54	4.63 ± 1.84	4.35 ± 1.34
C12:0		5.78 ± 2.89	24.2 ± 2.2	7.05 ± 1.14
C14:0		15.2 ± 5.37	42.6 ± 11.2	24.7 ± 6.29
C15:0		3.22 ± 1.09	3.52 ± 1.90	0.00 ± 0.00
C16:0		102 ± 34.1	318 ± 140	193 ± 19.8
C17:0		3.48 ± 0.91	3.45 ± 1.37	0.65 ± 0.17
C18:0		136 ± 30.2	437 ± 101	237 ± 43.5
C20:0		1.13 ± 0.31	3.77 ± 1.32	2.32 ± 0.57
Ʃ SFA		269 ± 66.6	837 ± 326	469 ± 80.2
c7 C16:1		7.40 ± 2.52	2.02 ± 3.21	5.05 ± 5.03
c9 C16:1		4.94 ± 2.91	4.09 ± 6.91	4.64 ± 2.79
c9 C18:1		27.0 ± 11.2	59.6 ± 39.1	51.9 ± 26.8
c11 C18:1		1.88 ± 1.79	8.57 ± 11.9	3.23 ± 3.51
Ʃ MUFA		41.2 ± 13.9	74.3 ± 48.1	64.8 ± 15.6
c9c12 C18:2 (LA)		13.1 ± 1.8	0.00 ± 0.00	27.7 ± 5.14
c5c8c11c14 C20:4 (AA)		0.80 ± 1.96	0.00 ± 0.00	1.95 ± 0.21
Ʃ PUFA		13.9 ± 1.32	0.00 ± 0.00	29.7 ± 8.11

Legends: SE—snail eggs; SM—snail mucus.

**Table 3:** The content of amino acids [mg/g] in raw materials derived from organic *H. aspersa* snail.

	Sample	SE (Fresh)	SE (Freeze-Dried)	SM (Freeze-Dried)
Amino Acid				
Cysteine		4.83 ± 0.06	5.19 ± 0.04	5.30 ± 0.04
Aspartic acid		1.85 ± 0.02	3.91 ± 0.04	0.44 ± 0.02
Glutamic acid		0.72 ± 0.04	0.62 ± 0.03	0.26 ± 0.02
Asparagine		0.00 ± 0.00	0.00 ± 0.00	0.00 ± 0.00
Glutamine		0.67 ± 0.02	1.83 ± 0.04	0.09 ± 0.02
Histidine		0.84 ± 0.04	1.83 ± 0.02	0.20 ± 0.02
Serine		0.99 ± 0.03	2.27 ± 0.04	0.70 ± 0.05
Arginine		1.48 ± 0.02	3.48 ± 0.07	0.00 ± 0.00
Glycine		0.26 ± 0.03	0.56 ± 0.04	0.00 ± 0.00
Threonine		0.29 ± 0.04	0.52 ± 0.02	0.00 ± 0.00
Tyrosine		0.69 ± 0.04	1.53 ± 0.03	0.00 ± 0.00
Alanine		0.38 ± 0.01	0.88 ± 0.03	0.16 ± 0.03
Taurine		0.00 ± 0.00	0.62 ± 0.06	0.00 ± 0.00
Methionine		0.00 ± 0.00	2.48 ± 0.06	0.00 ± 0.00
Valine		0.00 ± 0.00	0.19 ± 0.03	0.06 ± 0.01
Phenylalanine		1.01 ± 0.06	0.00 ± 0.00	0.19 ± 0.02
Isoleucine		0.41 ± 0.03	0.47 ± 0.03	0.10 ± 0.01
Leucine		0.41 ± 0.04	1.12 ± 0.03	0.00 ± 0.00
Cysteine		29.4 ± 0.10	70.3 ± 0.10	7.99 ± 0.06
Homocysteine		0.14 ± 0.03	0.33 ± 0.04	0.00 ± 0.00
Lysine		1.51 ± 0.02	2.93 ± 0.03	0.20 ± 0.01
ƩAA		45.9 ± 0.19	101 ± 0.17	15.7 ± 0.14
ƩexoAA		5.96 ± 0.08	13.0 ± 0.09	0.75 ± 0.07
ƩsulfuricAA		34.2 ± 0.16	78.0 ± 0.06	13.3 ± 0.05

Legends: SE—snail eggs; SM—snail mucus.

**Table 4:** Antimicrobial activity of SM and SE in agar diffusion method.

Diameter inhibition Zone [mm]					
	*S. aureus*	*P. aeruginosa*	*E. coli*	*C. albicans*	*A. brasiliensis*
SM	0 ± 0	0 ± 0	0 ± 0	0 ± 0	0 ± 0
SE	12 ± 0.9	3 ± 0.8	0 ± 0	0 ± 0	0 ± 0
ofloxacin 5 μg	29 ± 0.5	25 ± 0.5	35 ± 0	-	-
nystatin 100 IU	-	-	-	21 ± 0.4	15 ± 0

Legends: SE—snail eggs; SM—snail mucus.

**Table 5:** Minimum inhibitory concentration (MIC), minimum bactericidal concentration (MBC), and minimum fungicidal concentration (MFC) values of SM and SE against bacteria and yeast.

Microorganism	SM		SE		Novobiocin		Fluconazol	
	MIC [mg/mL]	MBC/MFC [mg/mL]	MIC [mg/mL]	MBC/MFC [mg/mL]	MIC [μg/mL]	MBC [μg/mL]	MIC [μg/mL]	MFC [μg/mL]
*S. aureus*	>50.00	>50.00	12.50	>50.00	0.49	0.98	-	-
*P. aeruginosa*	>50.00	>50.00	3.12	>50.00	15.6	31.25	-	-
*E. coli*	>50.00	>50.00	>50.00	>50.00	125	250	-	-
*C. albicans*	>50.00	>50.00	>50.00	>50.00	-	-	0.49	0.98

Legends: SE—snail eggs; SM—snail mucus.

**Table 6:** Radical scavenging activity of SM and SEs in ABTS methods.

EC50 [mg/mL]	
snail mucus	↑100
snail eggs	89.64
ascorbic acid	0.10

## Data Availability

Data are contained within the article.

## Supplementary Materials

The following supporting information can be downloaded at: https://www.mdpi.com/article/10.3390/ijms25189958/s1.
