# Supplementary material for: Biological Properties of the Mucus and Eggs of Helix aspersa Müller as a Potential Cosmetic and Pharmaceutical Raw Material: A Preliminary Study
Source: Int J Mol Sci. 2024 Sep 15;25(18):9958. doi: 10.3390/ijms25189958 (PMC11432642; doi:10.3390/ijms25189958)

Figure S2. Chromatogram of methanolic extract of fresh egg in negative ionisation (A – total ion chromatogram (TIC), B – total compound chromatogram (TCC).

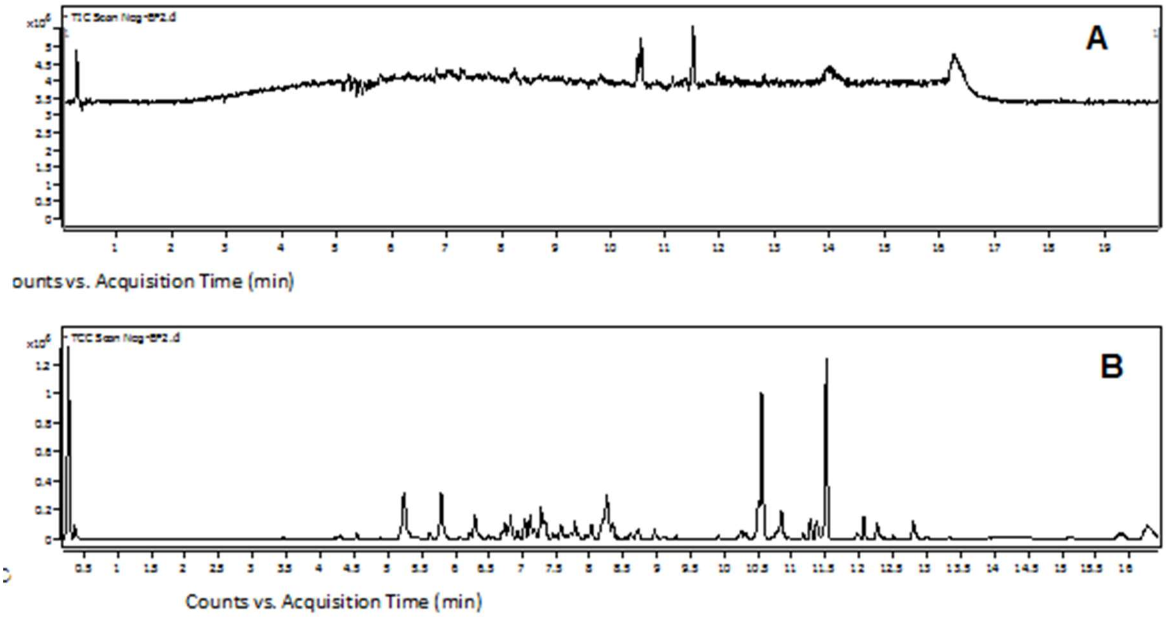

Supplement: Supplementary file 1 [file ijms-25-09958-s001.zip › Herman Anna - Figure S2.pdf]
