# Supplementary material for: Biological Properties of the Mucus and Eggs of Helix aspersa Müller as a Potential Cosmetic and Pharmaceutical Raw Material: A Preliminary Study
Source: Int J Mol Sci. 2024 Sep 15;25(18):9958. doi: 10.3390/ijms25189958 (PMC11432642; doi:10.3390/ijms25189958)

Figure S31. Sigmoidal dose-response curves for cisplatin determined for MCF-7, HT-29, HCT-116and Vero cells after 72 h of treatment. Plots were generated by GraphPad Prism after fitting the MTT data to sigmoidal dose-response equation: Y=100/(1+10^((LogIC50–X)*HillSlope)).

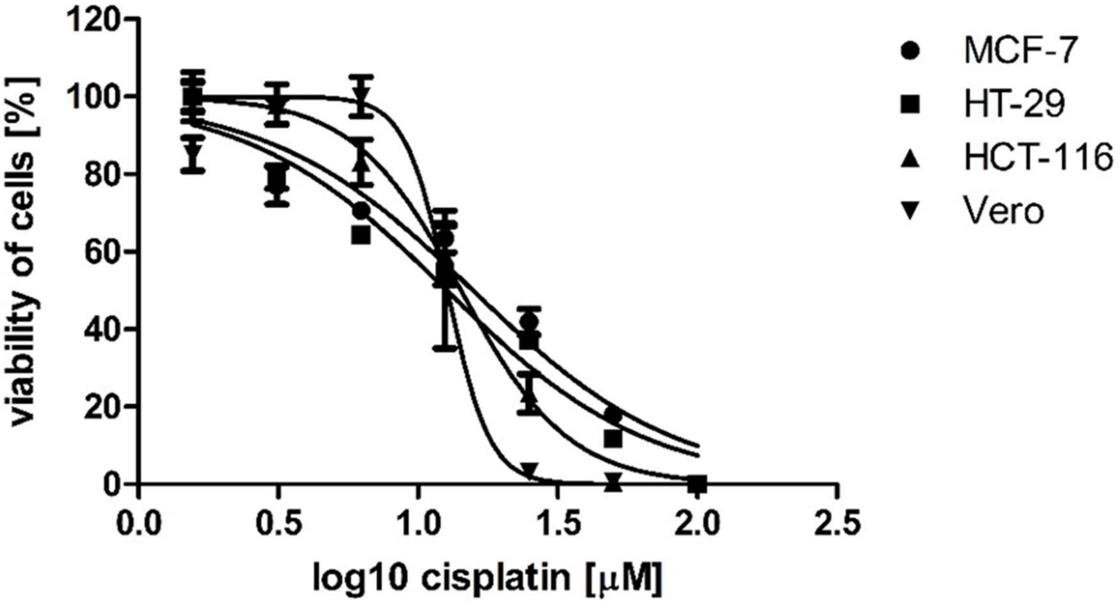

Supplement: Supplementary file 1 [file ijms-25-09958-s001.zip › Herman Anna - Figure S31.pdf]
