# Supplementary material for: Biological Properties of the Mucus and Eggs of Helix aspersa Müller as a Potential Cosmetic and Pharmaceutical Raw Material: A Preliminary Study
Source: Int J Mol Sci. 2024 Sep 15;25(18):9958. doi: 10.3390/ijms25189958 (PMC11432642; doi:10.3390/ijms25189958)
Supplement: Supplementary file 1 [file ijms-25-09958-s001.zip › Herman Anna - Table S1.pdf]

Table S1. Compounds identified in water extract of fresh egg of organic *Helix aspersa* snail using LC-MS.

No	Metabolite	RT[a] [min]	Mass [*m/z*]	Detection mode[b]
1	Methyl *N*-(a-methylbutyryl) glycine	4.305	188.1049	N
2	Ethiprole	5.805	395.9833	N
3	Zingerone	6.228	194.0944	N
4	Bismuth subsalicylate	6.705	361.9976	N
5	Eremopetasinorol	6.771	208.1464	N
6	Nordihydrocapsiate	6.832	294.1833	N
7	4-Hydroxy-5-phenyltetrahydro-1,3-oxazin-2-one	7.056	193.0740	N
8	(*S,Z*)-Lyratol acetate	7.114	194.1305	N
9	3b-Allotetrahydrocorticosterone	7.122	350.2456	N
10	Lauryl hydrogen sulfate	7.284	266.1553	N
11	L-Tyrosine methyl ester	7.342	195.0896	N
12	*N*-Undecylbenzenesulfonic acid	7.837	312.1758	N
13	2-Dodecylbenzenesulfonic acid	8.168	326.1916	N
14	Sodium Tetradecyl Sulfate	8.205	294.1862	N
15	Dinoterb	8.249	240.0749	N
16	Alcaftadine	8.887	307.1684	N
17	Gemfibrozil	8.957	250.1572	N
18	Furmecyclox	9.28	251.1522	N
19	(5b,7a,12a)-2-(3-methoxyphenyl)-2-oxoethyl ester-7,12-dihydroxy-cholan-24-oic acid	10.295	540.3454	N
20	Enalkiren	10.846	656.4289	N
21	Oleoylglycerone phosphate	11.167	434.2453	N
22	Adlupone	11.363	482.3394	N
23	(3beta,22E,24R)-3-Hydroxyergosta-5,8,22-trien-7-one	12.503	410.3183	N
24	Propyl 1-(propylsulfinyl)propyl disulfide	13.303	240.0667	N
25	L-Xylonate	13.304	166.0478	N
1	L-Homocysteic acid	0.241	183.0209	P

2	Trolamine	0.257	149.1055	P
3	2-Methyl-4,5-benzoxazole	0.259	133.0530	P
4	Dihydrocaffeic acid 3-Oglucuronide	0.260	358.0907	P
5	2-Amino-2-methyl-1,3-propanediol	0.261	105.0791	P
6	Dulcitol	0.262	182.0791	P
7	3-Hydroxyisoheptanoic acid	0.263	146.0944	P
8	Isoamyl nitrite	0.264	117.0790	P
9	Choline chloride	0.265	103.0997	P
10	Pandamarilactam 3x	0.265	235.1210	P
11	3-Methyl-2-butenoic acid	0.270	100.0524	P
12	Tetrahydrofurfuryl acetate	0.270	144.0792	P
13	Sucrose	0.271	342.1164	P
14	Asulam	0.275	230.0364	P
15	4'-Hydroxy-2-biphenylcarboxylic acid	0.278	214.0626	P
16	1-Aminocyclohexanecarboxylic acid	0.284	143.0948	P
17	(2*E*,11*Z*)-5-[5-(Methylthio)-4-penten-2-ynyl]-2-furanacrolein	0.300	232.0559	P
18	1-Deoxy-D-glucitol	0.301	166.0843	P
19	Aminocaproic acid	0.396	131.0947	P
20	L-Pipecolic acid	0.397	129.0791	P
21	(2*R*,3*R*,4*R*)-2-Amino-4-hydroxy-3-methylpentanoic acid	0.401	147.0897	P
22	L-2-Amino-5-hydroxypentanoic acid	0.437	133.0738	P
23	*R*-2-Hydroxy-3-methylbutanoic acid 3-Methylbutanoyl	0.672	202.1206	P
24	Dexpanthenol	0.879	205.1314	P
25	Isoleucyl-Threonine	0.905	232.1423	P
26	2,5-Dihydro-2,4,5-trimethyloxazole	1.536	113.0841	P
27	Pirimicarb	1.682	238.1420	P
28	Amyl 2-furoate	2.109	182.0944	P
29	2*E*-Decenedioic acid	2.398	200.1049	P
30	Propionyl-L-carnitine	2.542	218.1392	P

31	Octylamine	2.598	129.1517	P
32	5-Heptyltetrahydro-2-oxo-3-furancarboxylic acid	3.142	228.1362	P
33	Sedanonic acid	3.245	210.1255	P
34	Wine lactone	3.313	166.0995	P
35	Homoarecoline	3.472	169.1104	P
36	*N*-n-Hexanoylglycine methyl ester	3.472	187.1208	P
37	Varenicline	3.515	211.1110	P
38	Istamycin C1	3.587	431.2732	P
39	Gabapentin	3.616	171.1258	P
40	Netilmicin	3.738	475.2996	P
41	Sanshodiol	3.786	358.1417	P
42	Solanocapsine	3.786	430.3545	P
43	Monomenthyl succinate	3.854	256.1674	P
44	*N*-(3-oxo-octanoyl)-homoserine lactone	3.986	241.1312	P
45	Mukaadial	4.386	266.1521	P
46	1-Octen-3-yl glucoside	4.500	290.1729	P
47	Halstoctacosanolide A	4.526	844.5360	P
48	(*E*)-3-decen-1-ol	4.548	156.1514	P
49	Ethyl decanoate	4.553	200.1778	P
50	1,2,3-Tris(1-ethoxyethoxy)propane	4.674	308.2201	P
51	C12:1n-7	4.688	198.1620	P
52	Humulinic acid A	4.688	266.1519	P
53	*Gamma*-CEHC	4.689	248.1414	P
54	11-Hydroxy-9-tridecenoic acid	4.698	228.1723	P
55	Ruscopine	4.701	306.2047	P
56	2-Ethylacrylylcarnitine	4.733	244.1550	P
57	Methyl 3-(2,3-dihydroxy-3-methylbutyl)-4-hydroxybenzoate	4.814	254.1158	P
58	1,1,2-Triphenylpropane	5.021	272.1554	P
59	Sterebin E	5.079	338.2460	P

60	(*S*)-3-Octanol glucoside	5.101	292.1887	P
61	(-)-*trans*-Carveol glucoside	5.137	314.1734	P
62	Gibberellin A105	5.138	330.1469	P
63	Cyclonormammein	5.274	374.1721	P
64	Toxin T2 tetrol	5.310	298.1415	P
65	Acetyl tributyl citrate	5.385	402.2255	P
66	Asteltoxin	5.386	418.1991	P
67	Jasmolone glucoside	5.43	342.1678	P
68	Hydrocortisone succinate	5.481	462.2255	P
69	Corchoionol C 9-glucoside	5.536	386.1943	P
70	Isopulegone caffeate	5.538	316.1676	P
71	Eremopetasinorol	5.657	208.1463	P
72	Hexanal octane-1,3-diol acetal	5.704	228.2090	P
73	(5*alpha*,10*alpha*)-3,7(11)-Eudesmadien-2-one	5.765	218.1672	P
74	Avocadienofuran	5.766	246.1985	P
75	NAc-FnorLRF-amide	5.772	622.3566	P
76	Blumenol C *O*-[rhamnosyl-(1->6)-glucoside]	5.786	518.2728	P
77	Glaucamine	5.861	385.1523	P
78	*N*-Acetyl-2,6-diethylaniline	5.961	191.1312	P
79	Homodihydrojasmone	6.069	180.1516	P
80	16b-Hydroxyestrone	6.078	286.1567	P
81	2-Hydroxyestrone	6.146	286.1570	P
82	(*Z*)-6-Nonenal	6.150	140.1202	P
83	4-Hydroxy-3-methoxy-2,10-bisaboladien-9-one	6.212	266.1879	P
84	*alpha*-Butyl-omegahydroxypoly(oxyethylene) poly(oxypropylene)	6.273	248.1988	P
85	Gravelliferone	6.305	298.1571	P
86	*N*,2,3-Trimethyl-2-(1-methylethyl)butanamide	6.338	171.1618	P
87	(+)-Prosopinine	6.348	313.2617	P
88	1,1-Diethoxy-2-hexene	6.353	172.1462	P

89	Cuscohygrine	6.372	224.1889	P
90	Canavalioside	6.643	546.2674	P
91	10-Hydroxy-2,8- decadiene-4,6-diynoic acid	6.664	176.0473	P
92	Monoisobutyl phthalic acid	6.666	222.0892	P
93	C16 Sphinganine	6.683	273.2668	P
94	Cinegalline	6.782	430.2105	P
95	Erysothiopine	6.783	407.1023	P
96	Porson	6.783	386.1729	P
97	16-hydroxy hexadecanoic acid	6.790	272.2349	P
98	Funtumine	6.845	317.2718	P
99	5-Dodecyldihydro-2(3H)-furanone	6.883	254.2248	P
100	Chrycolide	6.942	232.0184	P
101	2,3-Dehydrosalvipisone	6.988	310.1575	P
102	Muricatacin	6.989	284.2354	P
103	Nonyl octanoate	6.990	270.2563	P
104	Lauroyl diethanolamide	7.006	287.2458	P
105	Acetyl Tyrosine Ethyl Ester	7.030	251.1159	P
106	Palmitic amide	7.031	255.2562	P
107	BILA 2185BS	7.043	618.3259	P
108	Cyclotetradecane	7.064	196.2191	P
109	Carbophenothion	7.090	341.9752	P
110	Terbucarb	7.093	277.2040	P
111	2-Tetradecanone	7.184	212.2143	P
112	7-Hydroxy-3-(4-methoxyphenyl)-4-methylcoumarin	7.188	282.0889	P
113	10,16-dihydroxy-palmitic acid	7.193	288.2301	P
114	Armillaric acid	7.243	416.1833	P
115	Cincassiol B	7.243	400.2100	P
116	Allopumiliotoxin 267A	7.250	267.2198	P
117	Bleekerine	7.318	409.1759	P

118	Testolactone	7.337	300.1727	P
119	Physagulin C	7.438	542.2509	P
120	2,2-Dimethyl-3,4-bis(4-methoxyphenyl)-2H-1-benzopyran-7-ol acetate	7.461	430.1778	P
121	Armillaripin	7.462	414.2045	P
122	Austalide L	7.462	428.2198	P
123	Erythroskyrin	7.462	455.2310	P
124	Vilazodone	7.462	441.2153	P
125	Methyl (9*Z*)-10'-oxo-6,10'-diapo-6-carotenoate	7.500	312.1726	P
126	Spiredine	7.870	353.1988	P
127	Phenethyl decanoate	7.875	276.2090	P
128	Spiroxamine	7.916	297.2670	P
129	*N-trans*-Feruloyloctopamine	7.919	329.1276	P
130	(10*S*)-Juvenile hormone III diol	7.958	284.1986	P
131	Dodecanamide	7.962	199.1937	P
132	Stearamide	8.012	283.2876	P
133	Undecylprodigiosin	8.174	393.2786	P
134	Lyngbyatoxin	8.277	437.3040	P
135	Tributyl phosphate	8.307	266.1646	P
136	Kukoamine D	8.408	530.3119	P
137	SB 221284	8.412	353.0819	P
138	Triphenyl phosphate	8.412	326.0708	P
139	Clavamycin B	8.413	362.1423	P
140	3L,7D,11D-phytanic acid	8.517	312.3025	P
141	*N*-(14-Methylhexadecanoyl)pyrrolidine	8.524	323.3188	P
142	8,8-Diethoxy-2,6-dimethyl-2-octanol	8.546	246.2193	P
143	(3a,5b,7a,12a)-24-[(carboxymethyl)amino]-1,12-dihydroxy-24-oxocholan-3-yl-b DGlucopyranosiduronica	8.547	641.3407	P
144	Protoprimulagenin A 3-[rhamnosyl-(1->4)-rhamnosyl-(1->4)-[rhamnosyl-(1->2)]-glucosyl-(1->?)-glucuronide]	8.547	1234.6226	P
145	Polysorbate 20	8.618	522.3408	P

146	Laserpitin	8.768	450.2614	P
147	Polysorbate 60	8.769	434.2882	P
148	Hexyl heptanoate	8.789	638.2368	P
149	9-Acetoxyfukinanolide	8.870	292.1678	P
150	MG(0:0/20:1(11*Z*)/0:0)	8.927	384.3242	P
151	Phytal	8.994	294.2924	P
152	3-Cyclohexyldodecane	9.012	252.2818	P
153	Isoacitretin	9.039	326.1885	P
154	Anofinic acid	9.111	204.0786	P
155	*Alpha*-CEHC	9.112	278.1519	P
156	22-Oxo-docosanoate	9.136	354.3136	P
157	(*E,E*)-1,6-bis(4-methoxyphenyl)-1,5-hexadiene	9.190	294.1621	P
158	1-(3-Hydroxy-4-methoxyphenyl)-1,2-ethanediol	9.207	184.0735	P
159	18-Oxocortisol	9.207	376.1883	P
160	Misoprostol	9.207	382.2703	P
161	Bioresmethrin	9.370	338.1883	P
162	Chloropyramine	9.371	289.1354	P
163	MG(0:0/16:0/0:0)	9.373	330.2769	P
164	(3'x,5'a,9'x,10'b)-O-(3-Hydroxy-6-oxo-7-drimen-11-yl)umbelliferone	9.433	396.1936	P
165	(E)-3-(2-Hydroxyphenyl)- 2-propenal	9.433	148.0525	P
166	[6]-Gingerdiol 3,5-diacetate	9.433	380.2200	P
167	Lilac alcohol	9.433	170.1308	P
168	Methandriol dipropionate	9.433	416.2913	P
169	A 77003	9.434	794.4300	P
170	(3b,6b,8b,12a)-8,12-Epoxy-7(11)-eremophilene-6-angeloyloxy-8,12-dimethoxy-3-ol	9.435	394.2357	P
171	Polidocanol	9.554	582.4346	P
172	Philanthotoxin 343	9.737	435.3202	P
173	Palmitoyl glucuronide	9.740	418.2935	P
174	Palmitoyl-EA	9.799	299.2825	P

175	Kamahine C	9.941	268.1310	P
176	Ampalex	9.942	241.1205	P
177	Cymorcin monoglucoside	9.942	328.1523	P
178	2,5-Furandicarboxylic acid	9.943	156.0057	P
179	Arbutin	9.943	272.0896	P
180	1b,3a,7a,12a-Tetrahydroxy-5b cholanoic acid	9.944	424.2810	P
181	4-Carboxy-2-hydroxy-6-methoxy-6-oxohexa-2,4-dienoate	9.944	216.0270	P
182	Vanillactic acid	9.944	212.0685	P
183	Tamoxifen	10.197	371.2250	P
184	Argentine	10.199	406.2022	P
185	DU 122290	10.200	362.1650	P
186	Drotaverine	10.381	397.2256	P
187	Petromyzonol	10.414	394.3084	P
188	Dodemorph	10.493	281.2719	P
189	Capsi-amide	10.643	269.2718	P
190	Drospirenone	10.683	366.2195	P
191	D-Glucosyldihydrosphingosine	10.804	463.3505	P
192	Enalkiren	10.846	656.4294	P
193	Propinol adenylate	10.894	403.0898	P
194	Cavipetin D	10.895	418.2719	P
195	Sorbitan palmitate	10.896	402.2985	P
196	Ganodermic acid TQ	11.094	510.3342	P
197	Camptothecin	11.242	348.1102	P
198	Pteroyltriglutamic acid	12.061	699.2259	P
199	Armillatin	12.145	610.4253	P
200	PC(14:0/22:5(4*Z*,7*Z*,10*Z*,13*Z*,16*Z*))	12.256	780.5533	P
201	12-Ketodeoxycholic acid	12.260	390.2768	P
202	PC(16:0/18:1(9*Z*))[*S*]	12.270	760.5848	P
203	Dioctyl hexanedioate	12.280	370.3082	P

204	Luffariellolide	12.281	386.2821	P
205	Gallocatechin-(4*alpha*->8)-gallocatechin-(4*alpha*->8)-gallocatechin	13.313	914.1876	P
206	*Beta*-Citraurol	13.669	434.3181	P
207	Epifisetinidol-(4*beta*->8)-epicatechin-(6->4*beta*)-epifisetinidol	14.041	834.2124	P
208	DG(14:0/22:1(13*Z*)/0:0)	14.151	622.5532	P
209	PE(P-16:0/20:5(5*Z*,8*Z*,11*Z*,14*Z*,17*Z*))	15.856	721.5028	P

[a] – retention time [min]

[b] –compound detection in positive (P) or in negative (N) ionization mode.
