# Supplementary material for: Biological Properties of the Mucus and Eggs of Helix aspersa Müller as a Potential Cosmetic and Pharmaceutical Raw Material: A Preliminary Study
Source: Int J Mol Sci. 2024 Sep 15;25(18):9958. doi: 10.3390/ijms25189958 (PMC11432642; doi:10.3390/ijms25189958)
Supplement: Supplementary file 1 [file ijms-25-09958-s001.zip › Herman Anna - Table S10.pdf]

Table S10. Compounds identified in acetonitrile-water* extract of lyophilized egg of organic *Helix aspersa* snail using LC-MS.

No	Metabolite	RT[a] [min]	Mass [*m/z*]	Detection mode[b]
1	Trifluoroacetic acid	0.254	113.9929	N
2	Dulcitol	0.256	182.0791	N
3	Dimethyl carbonate	0.259	90.0318	N
4	D-Fructose	0.260	180.0634	N
5	Spirodiclofen	0.263	410.1032	N
6	3-b-Galactopyranosyl glucose	0.27	342.1161	N
7	Methylpyrazine	0.315	94.0533	N
8	*N*-n-Hexanoylglycine methyl ester	3.465	187.1209	N
9	Methyl *N*-(amethylbutyryl)glycine	4.295	188.1051	N
10	Ethiprole	5.804	395.9833	N
11	Zingerone	6.231	194.0945	N
12	Azobenzene	6.298	182.0846	N
13	Flupropanate	6.563	145.9991	N
14	Bismuth subsalicylate	6.709	361.9979	N
15	Eremopetasinorol	6.776	208.1465	N
16	Nordihydrocapsiate	6.833	294.1832	N
17	3-Hydroxy-6,8-dimethoxy-7(11)-eremophilen-12,8-olide	7.035	310.1783	N
18	BILA 2185BS	7.041	618.3252	N
19	(*S,Z*)-Lyratol acetate	7.118	194.1309	N
20	3b-Allotetrahydrocorticosterone	7.120	350.2458	N
21	(3b,6b,8b,12a)-8,12-Epoxy-7(11)-eremophilene-6-angeloyloxy-8,12-dimethoxy-3-ol	7.201	394.2354	N
22	Lauryl hydrogen sulfate	7.281	266.1552	N
23	Zanthodioline	7.281	305.1269	N
24	4'-Methoxymucidin	7.283	288.1362	N
25	Methotrexate	7.316	454.1731	N
26	Losartan	7.317	422.1623	N

27	L-Tyrosine methyl ester	7.346	195.0897	N
28	Dinoterb	7.582	240.0748	N
29	*N*-Undecylbenzenesulfonic acid	7.723	312.1760	N
30	Ethyl 2-hydroxy-3-(3-indolyl)propanoate glucoside	7.782	395.1589	N
31	2-Dodecylbenzenesulfonic acid	8.159	326.1915	N
32	Sodium Tetradecyl Sulfate	8.203	294.1864	N
33	(+)-Prosopinine	8.276	313.2620	N
34	Docusate	8.341	422.2334	N
35	Kukoamine D	8.408	530.3120	N
36	Alcaftadine	8.888	307.1685	N
37	Gemfibrozil	8.960	250.1570	N
38	Furmecyclox	9.281	251.1523	N
39	Nisoldipine	9.390	388.1635	N
40	3-Oxochola-4,6-dien-24-oic Acid	10.249	370.2512	N
41	(5b,7a,12a)-2-(3-methoxyphenyl)-2-oxoethyl ester-7,12-dihydroxy-cholan-24-oic acid	10.488	540.3440	N
42	Oleamide	10.489	281.2718	N
43	5-Dodecyldihydro-2(3H)-furanone	10.504	254.2245	N
44	Auricularine	10.549	494.3399	N
45	DG(18:1(11*Z*)/22:5(4*Z*,7*Z*,10*Z*,13*Z*,16*Z*)/0:0)	10.550	668.5401	N
46	MG(0:0/16:0/0:0)	10.552	330.2769	N
47	Enalkiren	10.843	656.4285	N
48	3-Hydroxy-2-(4-morpholinylmethyl)estra-1,3,5(10)-trien-17-one	10.948	369.2306	N
49	Butroxydim	11.270	399.2409	N
50	Adlupone	11.366	482.3393	N
51	Drotaverine	11.448	397.2250	N
52	(3*beta*,22*E*,24*R*)-3-Hydroxyergosta-5,8,22-trien-7-one	11.504	410.3183	N
53	Callystatin A	11.504	456.3252	N
54	DG(20:5(5*Z*,8*Z*,11*Z*,14*Z*,17*Z*)/24:1(15*Z*)/0:0)	11.504	724.6025	N
55	MG(18:0/0:0/0:0)	11.506	358.3080	N

56	Pubesenolide	11.506	458.3052	N
1	3-hydroxy-4-methoxymandelate	0.256	198.0530	P
2	2-Methyl-4,5-benzoxazole	0.257	133.0531	P
3	2-Amino-2-methyl-1,3-propanediol	0.260	105.0789	P
4	Trolamine	0.261	149.1053	P
5	3-Hydroxyisoheptanoic acid	0.262	146.0943	P
6	Dihydrocaffeic acid 3-*O*-glucuronide	0.263	358.0903	P
7	Choline chloride	0.265	103.0997	P
8	Dulcitol	0.265	182.0791	P
9	L-Leucine	0.265	131.0941	P
10	Isoamyl nitrite	0.267	117.0790	P
11	3-Deoxyarabinohexonic acid	0.269	180.0636	P
12	Tetrahydrofurfuryl acetate	0.270	144.0790	P
13	3-b-Galactopyranosyl glucose	0.273	342.1163	P
14	4-Amino-2-methylenebutanoic acid	0.274	115.0633	P
15	1-Aminocyclohexanecarboxylic acid	0.275	143.0948	P
16	Sucrose	0.275	342.1163	P
17	Phenethicillin	0.311	364.1095	P
18	Purine	0.316	120.0435	P
19	(2*R*,4*S*)-2,4-Diaminopentanoate	0.318	132.0899	P
20	Bethanidine	0.318	177.1271	P
21	Naphtho[2,3-e]pyrene	0.318	302.1094	P
22	p-Coumaroylputrescine	0.320	234.1370	P
23	Dexpanthenol	0.873	205.1317	P
24	2,5-Dihydro-2,4,5-trimethyloxazole	1.452	113.0839	P
25	5-Heptyltetrahydro-2-oxo-3-furancarboxylic acid	2.963	228.1363	P
26	Geranyl acetoacetate	3.034	238.1568	P
27	3-hydroxytetradecanedioic acid	3.036	274.1781	P
28	Sedanonic acid	3.132	210.1260	P

29	Capryloylglycine	3.150	201.1365	P
30	2-Isopropyl-1,4-benzenediol	3.225	152.0839	P
31	Homoarecoline	3.466	169.1103	P
32	*N*-n-Hexanoylglycine methyl ester	3.466	187.1209	P
33	(*E*)-2-Methyl-2-buten-1-ol *O*-beta-DGlucopyranoside	3.569	248.1260	P
34	Istamycin C1	3.585	431.2729	P
35	2,3-Dihydro-5-(5-methyl-2-furanyl)-1H-pyrrolizine	3.609	187.0991	P
36	4-Vinylcyclohexene	3.613	108.0939	P
37	Slaframine	3.614	198.1368	P
38	Phlorin	3.712	288.0845	P
39	Threo-Syringoylglycerol	3.712	244.0944	P
40	Netilmicin	3.736	475.2995	P
41	1-[(5-Amino-5-carboxypentyl)amino]-1-deoxyfructose	3.815	308.1597	P
42	Monomenthyl succinate	3.851	256.1675	P
43	*N*-(3-oxo-octanoyl)-homoserine lactone	3.984	241.1316	P
44	Tributyrin	4.024	302.1729	P
45	Alanyl-Valine	4.026	188.1164	P
46	*N*-Methylmescaline	4.256	225.1365	P
47	Jasmine ketolactone	4.257	208.1100	P
48	Octyl gallate	4.289	282.1468	P
49	Humulinic acid A	4.388	266.1518	P
50	Triethylenemelamine	4.401	204.1131	P
51	Amyl 2-furoate	4.406	182.0942	P
52	1-Octen-3-yl glucoside	4.497	290.1731	P
53	Halstoctacosanolide A	4.526	844.5363	P
54	Gentamicin C2b	4.539	463.3019	P
55	(*E*)-3-decen-1-ol	4.548	156.1513	P
56	Diethofencarb	4.551	267.1469	P
57	Ethyl decanoate	4.553	200.1777	P

58	Flumetover	4.553	367.1392	P
59	Ethyl 3-(*N*-butylacetamido)propionate	4.651	215.1523	P
60	1,2,3-Tris(1-ethoxyethoxy)propane	4.673	308.2199	P
61	2,2,7,7-Tetramethyl-1,6-dioxaspiro[4.4]nona-3,8-diene	4.679	180.1152	P
62	Aspergillic acid	4.679	224.1526	P
63	C12:1n-7	4.686	198.1619	P
64	*Gamma*-CEHC	4.687	248.1410	P
65	11-Hydroxy-9-tridecenoic acid	4.693	228.1724	P
66	Ruscopine	4.703	306.2044	P
67	(5*R*)-5-Hydroxyhexanoic acid	4.730	132.0786	P
68	1-Phenyl-6,7-dihydroxyisochroman	4.732	242.0944	P
69	2,3-dihydrobenzofuran	4.732	120.0574	P
70	2-Ethylacrylylcarnitine	4.732	244.1550	P
71	2-Phenylbutyric acid	4.732	164.0837	P
72	3-Indolecarboxylic acid	4.733	253.1311	P
73	6'-Methoxypolygoacetophe noside	4.761	360.1062	P
74	Alanyl-Isoleucine	4.780	202.1318	P
75	N-Isobutyl-2,4,8,10,12-tetradecapentaenamide	4.803	273.2094	P
76	Gravolenic acid	4.804	280.0946	P
77	(*E*)-3-(2-Hydroxyphenyl)-2-propenal	4.820	148.0524	P
78	Pinidine	4.841	139.1362	P
79	Diphenylcarbazide	4.849	242.1159	P
80	Glycerol tripropanoate	4.850	260.1260	P
81	Methyl propionate	4.963	88.0524	P
82	Tetrahydroaldosterone-3-glucuronide	4.963	540.2553	P
83	4'-Hydroxy-3,4,5-trimethoxystilbene	4.964	286.1208	P
84	1,1,2-Triphenylpropane	5.022	272.1560	P
85	5,7-Megastigmadien-9-ol glucoside	5.041	356.2201	P
86	Cinncassiol A 19-glucoside	5.049	544.2521	P

87	Sterebin E	5.078	338.2458	P
88	2'-*N*-Acetylparomamine	5.093	365.1816	P
89	(*S*)-3-Octanol glucoside	5.100	292.1884	P
90	(-)-*trans*-Carveol glucoside	5.137	314.1732	P
91	Gibberellin A105	5.138	330.1466	P
92	7,8-Dihydrovomifoliol 9-[rhamnosyl-(1->6)-glucoside]	5.159	534.2681	P
93	Glycerol 1-(5-hydroxydodecanoate)	5.230	290.2095	P
94	Toxin T2 tetrol	5.243	298.1417	P
95	Cyclonormammein	5.275	374.1729	P
96	Elaeokanine C	5.287	211.1572	P
97	4,11,13,15-Tetrahydroridentin B	5.293	268.1680	P
98	Artabsinolide A	5.311	280.1310	P
99	Jasmolone glucoside	5.372	342.1682	P
100	Asteltoxin	5.385	418.1988	P
101	Triethyl citrate	5.409	276.1213	P
102	Valyl-Valine	5.428	216.1477	P
103	Hydrocortisone succinate	5.481	462.2255	P
104	Corchoionol C 9-glucoside	5.484	386.1943	P
105	*O*-Methylsomniferine	5.500	622.2657	P
106	(*E,E,E*)-*N*-(2-Methylpropyl)hexadeca-2,6,8-trien-10-ynamide	5.507	301.2406	P
107	4-Butyl-5-ethylthiazole	5.514	169.0925	P
108	Isopulegone caffeate	5.537	316.1674	P
109	Polyethylene, oxidized	5.544	244.1307	P
110	Taraxacolide 1-*O*-b-D- glucopyranoside	5.544	428.2046	P
111	Satratoxin H	5.568	528.2335	P
112	Convallatoxin	5.642	550.2772	P
113	Terazosin	5.651	387.1892	P
114	Eremopetasinorol	5.656	208.1462	P
115	*N*-Jasmonoylisoleucine	5.684	323.2097	P

116	(2xi,6xi)-7-Methyl-3-methylene-1,2,6,7-octanetetrol	5.701	204.1363	P
117	Hexanal octane-1,3-diol acetal	5.706	228.2091	P
118	2-Methylundecanal	5.727	184.1827	P
119	(5*alpha*,10*alpha*)-3,7(11)-Eudesmadien-2-one	5.769	218.1667	P
120	2-Furanmethanol	5.769	98.0367	P
121	Avocadienofuran	5.769	246.1984	P
122	NAc-FnorLRF-amide	5.774	622.3563	P
123	Blumenol C *O*-[rhamnosyl-(1->6)-glucoside]	5.789	518.2728	P
124	Fluspirilene	5.806	475.2417	P
125	2,2,4,4,-Tetramethyl-6-(1-oxopropyl)-1,3,5 cyclohexanetrione	5.822	238.1202	P
126	2-Hydroxymyristic Acid	5.834	244.2040	P
127	Ssioriside	5.835	554.2364	P
128	Glaucamine	5.860	385.1522	P
129	C14:1n-9	5.877	226.1933	P
130	Eriojaposide A	5.878	502.2412	P
131	Canavalioside	5.941	546.2678	P
132	*N*-Acetyl-2,6-diethylaniline	5.960	191.1312	P
133	Fluocinolone	5.979	412.1711	P
134	Capsoside A	6.015	694.3774	P
135	Capsaicin	6.062	305.1994	P
136	Homodihydrojasmone	6.070	180.1515	P
137	Tricycloekasantal	6.071	178.1357	P
138	Lauroyl diethanolamide	6.075	287.2462	P
139	2-Hydroxyestrone	6.081	286.1571	P
140	Momilactone B	6.131	330.1831	P
141	20-COOH-Leukotriene B4	6.145	366.2046	P
142	(*Z*)-6-Nonenal	6.149	140.1202	P
143	Penbutolol	6.179	291.2201	P
144	10,11-Epoxy-3,7,11-trimethyl-2*E*,6*E*-tridecadienoic acid	6.212	266.1883	P

145	Heliosupine	6.225	397.2104	P
146	(+)-Prosopinine	6.230	313.2621	P
147	*alpha*-Butyl-*omega*-hydroxypoly(oxyethylene) poly(oxypropylene)	6.270	248.1990	P
148	Chalciporone	6.288	243.1619	P
149	Gravelliferone	6.306	298.1571	P
150	Eucalyptol	6.335	154.1358	P
151	Plantaricin BN	6.339	484.2306	P
152	1,1-Diethoxy-2-hexene	6.360	172.1464	P
153	Cuscohygrine	6.373	224.1890	P
154	Dihydrocapsaicin	6.386	307.2148	P
155	Pterosin O	6.395	232.1464	P
156	Metoprolol	6.396	267.1833	P
157	Chaksine	6.604	450.2965	P
158	Monoisobutyl phthalic acid	6.664	222.0892	P
159	10-Hydroxy-2,8-decadiene-4,6-diynoic acid	6.665	176.0474	P
160	C16 Sphinganine	6.683	273.2668	P
161	Sphinganine	6.683	301.2980	P
162	Demethoxyegonol	6.687	296.1039	P
163	Acetyllycopsamine	6.695	341.1839	P
164	AF Toxin II	6.695	324.1571	P
165	Fetidine	6.695	682.3256	P
166	Glyceollidin II	6.696	340.1306	P
167	Senkirkine	6.696	365.1842	P
168	17-Methylandrosta-2,4-dieno[2,3-d]isoxazol-17*beta*-ol	6.706	327.2195	P
169	Glicoisoflavanone	6.712	384.1569	P
170	2-Tetradecanone	6.717	212.2140	P
171	1-Isomangostin hydrate	6.735	428.1833	P
172	5-(2,3-Dihydroxy-3-methylbutyl)-4-(3,4-epoxy-4-methylpentanoyl)-3,4-dihydroxy-2-isopentanoyl-2-cyclopenten-1-one	6.735	412.2099	P

173	Cerberoside	6.735	858.4196	P
174	D1927	6.754	456.2384	P
175	Cyanidin 3-O-(2"-xylosyl-6"-(6"-sinapoylglucosyl)-galactoside)	6.758	474.5086	P
176	Ximelagatran	6.758	473.2629	P
177	2-Methoxy-estradiol-17β 3-glucuronide	6.759	478.2181	P
178	Deacetylnomilin	6.759	472.2094	P
179	1alpha,3beta,22RTrihydroxyergosta-5,24Edien-26-oic acid 3-O-b-D-glucoside 26-O-[b-Dglucosyl-(1->2)-b-Dglucosyl] ester	6.760	946.4639	P
180	Austalide A	6.781	516.2359	P
181	Mycalamide B	6.781	517.2893	P
182	Trilobolide	6.782	522.2442	P
183	Cinegalline	6.786	430.2103	P
184	Porson	6.786	386.1729	P
185	16-hydroxyhexadecanoic acid	6.793	272.2354	P
186	Canescein	6.803	566.2708	P
187	Ipecoside	6.804	565.2180	P
188	Scilliroside	6.826	604.2878	P
189	Notoginsenoside Fc	6.827	1210.6248	P
190	Funtumine	6.848	317.2720	P
191	Phenethyl decanoate	6.860	276.2091	P
192	(*S*)-Nerolidol 3-*O*-[a-LRhamnopyranosyl-(1->4)-a-Lrhamnopyranosyl-(1->2)-b-Dglucopyranoside]	6.864	676.3672	P
193	2,4,12-Octadecatrienoic acid isobutylamide	6.864	333.3018	P
194	5-Dodecyldihydro-2(3H)-furanone	6.884	254.2247	P
195	2,6-Di-tert-butyl-4-ethylphenol	6.895	234.1985	P
196	Herculin	6.895	251.2250	P
197	Pumiliotoxin 251D	6.895	251.2249	P
198	1-Methyl-2-nonyl-4(1H)-quinolinone	6.897	285.2089	P
199	Genipin 1-betagentiobioside	6.901	550.1898	P
200	3-Hydroxy-6,8-dimethoxy-7(11)-eremophilen-12,8-olide	6.908	310.1779	P

201	1-Tridecene	6.916	182.2036	P
202	Zizybeoside II	6.920	594.2161	P
203	2-Hexadecanone	6.934	240.2454	P
204	Kanokoside C	6.938	638.2424	P
205	Chrycolide	6.944	232.0184	P
206	4,4-Difluoropregn-5-ene-3,20-dione	6.946	350.2069	P
207	Coriandrone D	6.962	352.1525	P
208	4,5-Dihydroniveusin A	6.964	396.1784	P
209	7-(4-Hydroxy-3-methoxyphenyl)-5-methoxy-1-phenyl-3-heptanone	6.965	342.1839	P
210	10-Deacetyl-2-debenzoylbaccatin III	6.977	440.2046	P
211	3'-Hydroxy-HT2 toxin	6.977	440.2046	P
212	Nonyl octanoate	6.992	270.2561	P
213	Muricatacin	6.995	284.2349	P
214	Coccinin	7.002	528.2569	P
215	2,5-Dimethyl-3(2H)-furanone	7.020	112.0526	P
216	Acetyl Tyrosine Ethyl Ester	7.021	251.1156	P
217	Palmitic amide	7.033	255.2564	P
218	BILA 2185BS	7.044	618.3256	P
219	Capsianoside I	7.045	660.3350	P
220	Cyclotetradecane	7.062	196.2192	P
221	Imidaprilat	7.083	377.1582	P
222	Glutamyl-Tryptophan	7.089	333.1323	P
223	Myxochelin A	7.089	404.1588	P
224	Terbucarb	7.093	277.2039	P
225	Acebutolol	7.098	336.2062	P
226	Spiroxamine	7.100	297.2669	P
227	5-Hexyltetrahydro-2-furanoctanoic acid	7.101	298.2509	P
228	2-Methoxyestradiol-3-methylether	7.124	316.2024	P
229	Sarcodon scabrosus Depsipeptide	7.126	485.2738	P

230	Finaconitine	7.128	630.3152	P
231	Taraxinic acid glucosyl ester	7.176	424.1731	P
232	Paucin	7.177	468.1996	P
233	7-Hydroxy-3-(4-methoxyphenyl)-4-methylcoumarin	7.185	282.0892	P
234	Z-Gly-Pro-Leu-Gly-Pro	7.190	573.2784	P
235	10,16-dihydroxy-palmitic acid	7.192	288.2299	P
236	*Cis*-5-Tetradecenoylcarnitine	7.199	370.2967	P
237	Cinnzeylanine	7.214	426.2257	P
238	Armillaric acid	7.243	416.1835	P
239	Cincassiol B	7.244	400.2101	P
240	Allopumiliotoxin 267A	7.253	267.2200	P
241	Panaquinquecol 1	7.253	292.2039	P
242	*trans*-9, *trans*-11-octadecadienoic acid; C18:2n-7,9	7.254	280.2405	P
243	Zygadenine	7.256	493.3027	P
244	Dodecylguanidine	7.265	227.2364	P
245	Armillaripin	7.329	414.2038	P
246	Ethyl (4*Z*)-4,7-octadienoate	7.356	168.1150	P
247	6-*trans*-LTB4	7.360	336.2294	P
248	PGB1	7.360	336.2298	P
249	Sambacin	7.361	540.2199	P
250	Estrane-3α,17α-diol	7.379	278.2244	P
251	3-Methyl-*alpha*-ionyl acetate	7.382	250.1932	P
252	*N*-Dealkylatedtolterodine	7.382	283.1931	P
253	Physagulin C	7.440	542.2511	P
254	Gabapentin	7.441	171.1259	P
255	Etiocholan-3α-ol-17-one3-glucuronide	7.457	466.2561	P
256	Austalide L	7.462	428.2198	P
257	2,2-Dimethyl-3,4-bis(4-methoxyphenyl)-2H-1-benzopyran-7-ol acetate	7.463	430.1778	P
258	DHAP(18:0)	7.463	436.2596	P

259	Erythroskyrin	7.463	455.2305	P
260	Methyl (9*Z*)-10'-oxo-6,10'-diapo-6-carotenoate	7.503	312.1727	P
261	Norpropoxyphene	7.514	325.2037	P
262	Angiotensin IV	7.542	774.4035	P
263	Fumonisin A2	7.558	747.4042	P
264	Steviolbioside	7.583	642.3249	P
265	8-Pentanoylneosolaniol	7.659	466.2208	P
266	Dihomo-γ-linolenoyl-EA	7.678	349.2967	P
267	Parsonsine	7.679	439.2208	P
268	Biperiden	7.681	311.2247	P
269	1-(4-Amino-2-methylpyrimid-5-ylmethyl)-3-(*beta*-hydroxyethyl)-2-methylpyridinium	7.748	259.1551	P
270	7,10-Hexadecadienoic acid	7.776	252.2092	P
271	6,10,14-Trimethyl-5,9,13-pentadecatrien-2-one	7.778	262.2286	P
272	Glycosides	7.779	584.2844	P
273	Methyl 15-cyanopentadecanoate	7.792	281.2356	P
274	Phlegmarine	7.838	250.2409	P
275	Sanshodiol	7.840	358.1418	P
276	Methadone	7.875	309.2089	P
277	Methyloctatropine	7.881	282.2435	P
278	Dihydrodioscorine	7.906	223.1570	P
279	Pristanic acid	7.923	298.2865	P
280	9-HOTE	7.940	294.2195	P
281	Elaiophylin	7.945	1024.5933	P
282	(3a,5b)-24-oxo-24-[(2-sulfoethyl)amino]cholan-3-yl-b-D-Glucopyranosiduronic acid	7.952	659.3343	P
283	Hematoporphyrin	7.956	598.2813	P
284	Dodecanamide	7.961	199.1938	P
285	Asparagoside D	7.966	902.4883	P
286	Scopoloside II	8.004	770.4086	P
287	2-Methoxyestrone 3-sulfate	8.015	380.1299	P

288	Stearamide	8.015	283.2874	P
289	MG(0:0/18:1(11*Z*)/0:0)	8.018	356.2926	P
290	Leucomycin A9	8.019	743.4090	P
291	Corchorusoside B	8.036	682.3564	P
292	Dihydro-5-(2-octenyl)-2(3H)-furanone	8.119	196.1464	P
293	1,26-Dicaffeoylhexacosanediol	8.141	722.4750	P
294	Lymecycline	8.143	602.2580	P
295	Undecylprodigiosin	8.171	393.2782	P
296	2,2,7,7-Tetramethyl-1,6-dioxaspiro[4.4]non-3-ene	8.193	182.1308	P
297	2-oxophytanic acid	8.204	326.2826	P
298	Erinacine G	8.270	464.2421	P
299	Lyngbyatoxin	8.271	437.3048	P
300	2-(4-Methylphenyl)-2-propanol	8.275	150.1043	P
301	Pipericine	8.275	335.3172	P
302	17*beta*-Acetamidoandrost-4-en-3-one	8.276	329.2354	P
303	Pipercitine	8.276	349.3328	P
304	p-Mentha-1,3,5,8-tetraene	8.276	132.0938	P
305	6-Oxocineole	8.277	168.1150	P
306	MG(0:0/20:2(11*Z*,14*Z*)/0:0)	8.294	382.3083	P
307	Tributyl phosphate	8.308	266.1649	P
308	1-Phenyl-1,3-dodecanedione	8.315	274.1934	P
309	3-Ethyl-2-hydroxy-4-methyl-2-cyclopenten-1-one	8.344	140.0838	P
310	Lentiginosine	8.344	157.1104	P
311	1-Methyl-1,3-cyclohexadiene	8.345	94.0782	P
312	Isopentylideneisopentylamine	8.345	155.1675	P
313	Santene	8.353	122.1095	P
314	N-Methylpelletierine	8.360	155.1311	P
315	Methyl 2-octynoate	8.362	154.0995	P
316	Isometheptene	8.363	141.1517	P

317	Homostachydrine	8.369	158.1185	P
318	Isopentyl *beta*-D-glucoside	8.384	250.1418	P
319	Flabellidine	8.387	288.2204	P
320	Mycinamicin VIII	8.397	505.3388	P
321	Kukoamine D	8.404	530.3123	P
322	Clavamycin B	8.412	362.1424	P
323	Triphenyl phosphate	8.412	326.0710	P
324	B 823-08	8.413	353.0820	P
325	Carpaine	8.450	478.3767	P
326	Methyprylon	8.451	183.1260	P
327	Dicyclomine	8.473	309.2667	P
328	12S-HEPE	8.513	318.2202	P
329	3L,7D,11D-phytanic acid	8.515	312.3029	P
330	Polidocanol	8.517	582.4347	P
331	Linoleoyl Ethanolamide	8.524	323.2827	P
332	*N*-(14-Methylhexadecanoyl)pyrrolidine	8.526	323.3189	P
333	Dodemorph	8.540	281.2718	P
334	8,8-Diethoxy-2,6- dimethyl-2-octanol	8.547	246.2197	P
335	Protoprimulagenin A 3-[rhamnosyl-(1->4)-rhamnosyl-(1->4)-[rhamnosyl-(1->2)]-glucosyl-(1->?)-glucuronide]	8.547	1234.6251	P
336	Madlongiside C	8.548	636.3863	P
337	Palmitoyl glucuronide	8.589	418.2927	P
338	Vaccenyl carnitine	8.607	425.3508	P
339	Polysorbate 20	8.619	522.3406	P
340	Palmitoyl-EA	8.631	299.2827	P
341	Isopimara-7,15-dienol	8.682	288.2453	P
342	LysoPC(14:0)	8.687	468.3097	P
343	Tecostanine	8.744	183.1622	P
344	Polysorbate 60	8.769	434.2882	P
345	Laserpitin	8.770	450.2610	P

346	Hexyl heptanoate	8.789	638.2359	P
347	Dehydrocarpaine II	8.820	474.3439	P
348	PS(18:0/22:5(7*Z*,10*Z*,13*Z*,16*Z*,19*Z*))	8.854	837.5561	P
349	9-Acetoxyfukinanolide	8.867	292.1674	P
350	13-heptadecyn-1-ol	8.883	252.2453	P
351	MG(0:0/20:1(11*Z*)/0:0)	8.925	384.3242	P
352	Tris(butoxyethyl)phosphate	8.928	398.2437	P
353	20,21,21-Trifluoro-3-methoxy-19-nor-17*alpha*-pregna-1,3,5(10),20-tetraen-17-ol	8.945	366.1812	P
354	Oleoyl Ethanolamide	8.948	325.2983	P
355	Phytal	8.994	294.2924	P
356	3-Cyclohexyldodecane	9.014	252.2819	P
357	Isoacitretin	9.040	326.1883	P
358	(*E,E*)-1,6-bis(4-methoxyphenyl)-1,5-hexadiene	9.042	294.1620	P
359	24-Hydroxycalcitriol	9.091	432.3236	P
360	*Alpha*-CEHC	9.111	278.1520	P
361	Anofinic acid	9.111	204.0786	P
362	22-Oxo-docosanoate	9.136	354.3134	P
363	(E)-1-[4-Hydroxy-3-(3-methyl-1,3-butadienyl)phenyl]-2-(3,5-dihydroxyphenyl)ethylene	9.161	294.1252	P
364	MG(0:0/22:2(13*Z*,16*Z*)/0:0)	9.170	410.3397	P
365	(6*beta*,7*alpha*,12*beta*,13*beta*)-7-Hydroxy-11,16-dioxo-8,14-apianadien-22,6-olide	9.189	384.1932	P
366	18-Oxocortisol	9.207	376.1884	P
367	1-(3-Hydroxy-4-methoxyphenyl)-1,2-ethanediol	9.210	184.0735	P
368	Misoprostol	9.211	382.2705	P
369	Tsangane L 3-glucoside	9.213	374.2303	P
370	Lansioside A	9.216	659.4371	P
371	Guaioxide	9.236	222.1981	P
372	Neogrifolin	9.252	328.2403	P
373	Gentamicin	9.269	477.3147	P
374	PE(15:0/22:6(4*Z*,7*Z*,10*Z*,13*Z*,16*Z*,19*Z*))	9.334	749.5010	P

375	Pravastatin	9.365	424.2467	P
376	Bioresmethrin	9.370	338.1883	P
377	MG(0:0/16:0/0:0)	9.375	330.2774	P
378	Cyclopassifloside II	9.376	682.4272	P
379	MG(0:0/22:6(4*Z*,7*Z*,10*Z*,13*Z*,16*Z*,19*Z*)/0:0)	9.425	402.2755	P
380	(3'x,5'a,9'x,10'b)-*O*-(3-Hydroxy-6-oxo-7-drimen-11-yl)umbelliferone	9.428	396.1935	P
381	PE(14:0/18:3(6*Z*,9*Z*,12*Z*))	9.428	685.4665	P
382	(3b,6b,8b,12a)-8,12-Epoxy-7(11)-eremophilene-6-angeloyloxy-8,12-dimethoxy-3-ol	9.429	394.2357	P
383	Methandriol dipropionate	9.431	416.2911	P
384	Sphinganine-phosphate	9.431	381.2658	P
385	Lilac alcohol	9.435	170.1308	P
386	Calendulaglycoside E	9.436	794.4263	P
387	Monoiodothyronine	9.436	398.9963	P
388	Vitisidin A	9.436	399.0714	P
389	3-(5,6,6-Trimethylbicyclo[2.2.1]hept-1-yl)cyclohexanol	9.460	236.2141	P
390	MG(0:0/18:3(6*Z*,9*Z*,12*Z*)/0:0)	9.467	352.2614	P
391	Lauroyl peroxide	9.477	398.3396	P
392	Galbanic acid	9.498	398.2086	P
393	Piscerythramine	9.593	451.2008	P
394	4*beta*-(2-Aminoethylthio)catechin	9.642	365.0927	P
395	2-(4-Chloro-3,5-dimethylphenoxy)-*N*-(2-phenyl-2H-benzotriazol-5-yl)-acetamide	9.643	406.1203	P
396	Monocrotaline	9.644	325.1530	P
397	Glycidyl oleate	9.698	338.2820	P
398	Lycopersiconol	9.764	334.2505	P
399	MG(0:0/22:1(13*Z*)/0:0)	9.838	412.3552	P
400	1b,3a,7a,12a-Tetrahydroxy-5bcholanoic acid	9.942	424.2809	P
401	Vanillactic acid	9.943	212.0684	P
402	Acetyl tributyl citrate	9.944	402.2254	P
403	Cinitapride	9.944	402.2257	P

404	Cymorcin monoglucoside	9.944	328.1523	P
405	2,5-Furandicarboxylic acid	9.945	156.0058	P
406	4-Carboxy-2-hydroxy-6-methoxy-6-oxohexa-2,4-dienoate	9.945	216.0270	P
407	Arbutin	9.945	272.0896	P
408	Kamahine C	9.945	268.1310	P
409	Gorgostane skeleton	10.090	412.4057	P
410	(*Z*)-9-Cycloheptadecen-1-one	10.118	250.2298	P
411	Balofloxacin	10.184	389.1759	P
412	Hellebrin	10.191	724.3296	P
413	DU 122290	10.193	362.1652	P
414	Drotaverine	10.382	397.2256	P
415	[6]-Gingerdiol 3,5-diacetate	10.410	380.2200	P
416	7-Ketodeoxycholic acid	10.426	406.2705	P
417	DG(15:0/20:1(11*Z*)/0:0)	10.490	608.5355	P
418	(±)-(*Z*)-2-(5-Tetradecenyl)cyclobutanone	10.491	264.2452	P
419	Oleamide	10.491	281.2722	P
420	Dodecylbenzene	10.493	246.2346	P
421	Lucidumol A	10.502	472.3551	P
422	DG(18:1(11*Z*)/22:5(4*Z*,7*Z*,10*Z*,13*Z*,16*Z*)/0:0)	10.551	668.5405	P
423	DG(20:3(5*Z*,8*Z*,11*Z*)/22:6(4*Z*,7*Z*,10*Z*,13*Z*,16*Z*,19*Z*)/0:0)	10.552	690.5221	P
424	DG(14:0/22:4(7*Z*,10*Z*,13*Z*,16*Z*)/0:0)	10.567	616.5045	P
425	4-Nerolidylcatechol	10.593	314.2249	P
426	Drospirenone	10.683	366.2196	P
427	(3*S*,6*E*,10*E*)-1,6,10,14-Phytatetraen-3-ol	10.737	290.2608	P
428	Spirolide F	10.791	727.5029	P
429	2-Pentadecylfuran	10.835	278.2612	P
430	Enalkiren	10.846	656.4288	P
431	D-myo-Inositol-1,4,5-triphosphate	10.895	419.9629	P
432	Doripenem	10.895	420.1133	P

433	Methyl 3,4-dihydroxy-5-prenylbenzoate 3-glucoside	10.895	398.1586	P
434	Cavipetin D	10.896	418.2722	P
435	Sorbitan palmitate	10.896	402.2984	P
436	Aspidospermine	11.071	354.2320	P
437	L-365260	11.083	398.1746	P
438	Mitragynine	11.086	398.2210	P
439	Mepanipyrim	11.087	223.1117	P
440	4-(Methylnitrosamino)-1-(3-pyridyl)-1-butanol glucuronide	11.099	385.1469	P
441	Merodesmosine	11.104	402.2460	P
442	Stearidonyl carnitine	11.110	420.3125	P
443	Eletriptan	11.125	382.1710	P
444	Stearoylethanolamide	11.132	327.3138	P
445	Arginyl-Tryptophan	11.135	360.1911	P
446	Withanolide B	11.349	454.2700	P
447	*Beta*-Elemonic acid	11.372	454.3442	P
448	*N*-Hexadecanoylpyrrolidine	11.387	309.3034	P
449	1,2-Epoxypropane	11.507	58.0417	P
450	DG(20:5(5*Z*,8*Z*,11*Z*,14*Z*,17*Z*)/24:1(15*Z*)/0:0)	11.508	724.6034	P
451	MG(18:0/0:0/0:0)	11.508	358.3084	P
452	Tridemorph	11.667	297.3031	P
453	Coenzyme F430	12.122	905.2912	P
454	Armillatin	12.146	610.4260	P
455	PC(14:0/22:5(4*Z*,7*Z*,10*Z*,13*Z*,16*Z*))	12.253	780.5546	P
456	12-Ketodeoxycholic acid	12.258	390.2775	P
457	PC(16:0/18:1(9*Z*))[*S*]	12.270	760.5860	P
458	Dioctyl hexanedioate	12.281	370.3085	P
459	Testosterone isocaproate	12.281	386.2817	P
460	*Beta*-Citraurol	13.666	434.3188	P
461	DG(14:0/22:1(13*Z*)/0:0)	14.097	622.5535	P

462	DG(14:1(9*Z*)/24:1(15*Z*)/0:0)	14.256	648.5692	P
463	DG(14:0/24:1(15*Z*)/0:0)	15.755	650.5846	P

* - acetonitrile: water (1:1, *v/v*)

[a] – retention time [min]

[b] –compound detection in positive (P) or in negative (N) ionization mode.
