# Supplementary material for: Biological Properties of the Mucus and Eggs of Helix aspersa Müller as a Potential Cosmetic and Pharmaceutical Raw Material: A Preliminary Study
Source: Int J Mol Sci. 2024 Sep 15;25(18):9958. doi: 10.3390/ijms25189958 (PMC11432642; doi:10.3390/ijms25189958)
Supplement: Supplementary file 1 [file ijms-25-09958-s001.zip › Herman Anna - Table S11.pdf]

Table S11. Compounds identified in water extract of lyophilized mucus of organic *Helix aspersa* snail using LC-MS.

**No**	**Metabolite**	**RT[a] [min]**	**Mass [*m/z*]**	**Detection mode[b]**
1	4-Sulfolactone	0.258	221.9826	N
2	Acrylic acid	0.268	72.0211	N
3	L-Rhamnulose	0.269	164.0687	N
4	Dulcitol	0.270	182.0792	N
5	Propargyl alcohol	0.270	56.0263	N
6	Dimethyl carbonate	0.271	90.0317	N
7	2(3H)-Furanone	0.273	84.0213	N
8	Citric acid	0.275	192.0273	N
9	Maltitol	0.275	344.1319	N
10	2-Succinyl-5-enolpyruvyl-6-hydroxy-3-cyclohexene-1-carboxylate	0.313	328.0784	N
11	2-[(5-Methylsulfinyl)-4-penten-2-ynylidene]-1,6-dioxaspiro[4.4]non-3-ene	0.318	250.0666	N
12	TRIBOA	4.207	197.0328	N
13	Methyl *N*-(amethylbutyryl)glycine	4.301	188.1049	N
14	3,5-Dinitroguaiacol	4.672	214.0227	N
15	Ethiprole	5.805	395.9833	N
16	Zingerone	6.231	194.0944	N
17	Bismuth subsalicylate	6.709	361.9978	N
18	Nordihydrocapsiate	6.833	294.1831	N
19	BILA 2185BS	7.040	618.3253	N
20	Methyl 2-benzamidoacetate	7.057	193.0738	N
21	(*S,Z*)-Lyratol acetate	7.116	194.1308	N
22	Erinacine G	7.117	464.2392	N
23	3b-Allotetrahydrocorticosterone	7.122	350.2456	N
24	Lauryl hydrogen sulfate	7.282	266.1554	N
25	Methotrexate	7.314	454.1733	N
26	Losartan	7.315	422.1623	N

27	L-Tyrosine methyl ester	7.345	195.0897	N
28	Dinoterb	7.578	240.0744	N
29	*N*-Undecylbenzenesulfonic acid	7.725	312.1758	N
30	2-Dodecylbenzenesulfonic acid	8.160	326.1912	N
31	Sodium Tetradecyl Sulfate	8.202	294.1863	N
32	Kukoamine D	8.407	530.3116	N
33	Alcaftadine	8.887	307.1688	N
34	Gemfibrozil	8.960	250.1570	N
35	Furmecyclox	9.281	251.1522	N
36	3-Oxochola-4,6-dien-24-oic Acid	10.249	370.2509	N
37	(5b,7a,12a)-2-(3-methoxyphenyl)-2-oxoethyl ester-7,12-dihydroxy-cholan-24-oic acid	10.292	540.3445	N
38	Enalkiren	10.849	656.4284	N
39	Butroxydim	11.271	399.2410	N
40	Adlupone	11.367	482.3394	N
41	Hydroxysintaxanthin 5,6-epoxide	11.946	462.3134	N
42	(3*beta*,22*E*,24*R*)-3-Hydroxyergosta-5,8,22-trien-7-one	12.505	410.3183	N
43	3-Deoxy-D-glycero-Dgalacto-2-nonulosonic acid	13.309	268.0790	N
44	L-Xylonate	13.311	166.0478	N
45	Nonanoyl-CoA	13.311	907.2338	N
1	L-Homocysteic acid	0.241	183.0208	P
2	Temocaprilat	0.262	448.1108	P
3	Norvaline	0.266	117.0790	P
4	6-Hydroxymusizin 8-*O*-b-D-glucopyranoside	0.267	394.1257	P
5	Glucoerucin	0.267	421.0551	P
6	2-Acetylfuran	0.272	110.0369	P
7	3-deoxyfructose	0.272	164.0684	P
8	Osmundalactone	0.272	128.0473	P
9	2-methyl-glutaric acid	0.273	146.0578	P
10	Dulcitol	0.273	182.0791	P

11	2-Furanmethanol	0.274	98.0364	P
12	Isradipine	0.276	371.1472	P
13	3-hydroxy-4-methoxymandelate	0.279	198.0530	P
14	Tetrahydrofurfuryl acetate	0.281	144.0787	P
15	Isoamyl nitrite	0.282	117.0791	P
16	Maltitol	0.282	344.1319	P
17	4-Guanidinobutanoic acid	0.313	145.0851	P
18	(2*R**,3*R**)-1,2,3-Butanetriol	0.355	106.0636	P
19	Trolamine	0.393	149.1052	P
20	L-Leucine	0.397	131.0946	P
21	Isoleucyl-Threonine	0.898	232.1424	P
22	Polypropylene glycol (mw 1,200-3,000)	1.019	134.0944	P
23	2,5-Dihydro-2,4,5-trimethyloxazole	1.536	113.0842	P
24	Istamycin C1	3.588	431.2734	P
25	1,2-Bis(1-ethoxyethoxy)propane	3.726	220.1676	P
26	Netilmicin	3.738	475.2993	P
27	Penciclovir	3.918	253.1177	P
28	1,11-Undecanedicarboxylic acid	4.102	244.1673	P
29	Toxin T2 tetrol	4.186	298.1420	P
30	Riesling acetal	4.190	226.1569	P
31	1-Octen-3-yl glucoside	4.501	290.1730	P
32	Halstoctacosanolide A	4.528	844.5367	P
33	(*E*)-3-decen-1-ol	4.551	156.1514	P
34	Ethyl decanoate	4.555	200.1778	P
35	1,2,3-Tris(1-ethoxyethoxy)propane	4.674	308.2200	P
36	C12:1n-7	4.687	198.1618	P
37	11-Hydroxy-9-tridecenoic acid	4.697	228.1719	P
38	2-Ethylacrylylcarnitine	4.734	244.1550	P
39	Methyl 3-(2,3-dihydroxy-3-methylbutyl)-4-hydroxybenzoate	4.815	254.1154	P

40	Pinidine	4.847	139.1361	P
41	Ganglioside GM3 (d18:1/16:0)	4.949	1152.7196	P
42	1,1,2-Triphenylpropane	5.021	272.1557	P
43	Sterebin E	5.079	338.2456	P
44	(*S*)-3-Octanol glucoside	5.103	292.1887	P
45	(-)-*trans*-Carveol glucoside	5.130	314.1720	P
46	Ganglioside GM3 (d18:0/18:1(11*Z*))	5.257	1180.7500	P
47	Alizapride	5.310	315.1685	P
48	Triethyl citrate	5.410	276.1208	P
49	Gravacridonetriol glucoside	5.412	519.1723	P
50	Jasmolone glucoside	5.432	342.1678	P
51	4-Butyl-5-ethylthiazole	5.499	169.0926	P
52	Corchoionol C 9-glucoside	5.538	386.1936	P
53	Eremopetasinorol	5.657	208.1464	P
54	Hexanal octane-1,3-diol acetal	5.707	228.2089	P
55	Avocadienofuran	5.768	246.1984	P
56	(5*alpha*,10*alpha*)-3,7(11)-Eudesmadien-2-one	5.769	218.1670	P
57	2,2,4,4,-Tetramethyl-6-(1-oxopropyl)-1,3,5-cyclohexanetrione	5.818	238.1203	P
58	Glaucamine	5.861	385.1526	P
59	Sanshodiol	5.861	358.1418	P
60	Homodihydrojasmone	6.071	180.1515	P
61	16b-Hydroxyestrone	6.080	286.1568	P
62	2-Hydroxyestrone	6.147	286.1570	P
63	(*Z*)-6-Nonenal	6.151	140.1202	P
64	4-Hydroxy-3-methoxy-2,10-bisaboladien-9-one	6.213	266.1881	P
65	*alpha*-Butyl-*omega*hydroxypoly(oxyethylene) poly(oxypropylene)	6.272	248.1989	P
66	Chalciporone	6.285	243.1626	P
67	Gravelliferone	6.307	298.1570	P
68	*N*,2,3-Trimethyl-2-(1-methylethyl)butanamide	6.335	171.1625	P

69	1,1-Diethoxy-2-hexene	6.354	172.1457	P
70	1,2-Epoxypropane	6.359	58.0418	P
71	Cuscohygrine	6.373	224.1889	P
72	Lilac alcohol	6.612	170.1305	P
73	Canavalioside	6.643	546.2675	P
74	10-Hydroxy-2,8-decadiene-4,6-diynoic acid	6.667	176.0473	P
75	Monoisobutyl phthalic acid	6.668	222.0892	P
76	Momilactone B	6.677	330.1829	P
77	C16 Sphinganine	6.688	273.2667	P
78	16-hydroxy hexadecanoic acid	6.793	272.2350	P
79	Funtumine	6.848	317.2721	P
80	Chrycolide	6.944	232.0186	P
81	7-(4-Hydroxy-3-methoxyphenyl)-5-methoxy-1-phenyl-3-heptanone	6.971	342.1830	P
82	Lauroyl diethanolamide	7.011	287.2460	P
83	Acetyl Tyrosine Ethyl Ester	7.030	251.1158	P
84	Palmitic amide	7.033	255.2562	P
85	BILA 2185BS	7.043	618.3258	P
86	Phosphoric acid	7.043	97.9769	P
87	Carbophenothion	7.092	341.9753	P
88	Terbucarb	7.094	277.2043	P
89	Finaconitine	7.126	630.3158	P
90	Nonyl octanoate	7.154	270.2559	P
91	10,16-dihydroxy-palmitic acid	7.193	288.2294	P
92	Cincassiol B	7.243	400.2100	P
93	Armillaric acid	7.244	416.1833	P
94	Bleekerine	7.318	409.1755	P
95	Testolactone	7.343	300.1726	P
96	Physagulin C	7.440	542.2504	P
97	Armillaripin	7.463	414.2043	P

98	Vilazodone	7.465	441.2155	P
99	Methyl (9*Z*)-10'-oxo-6,10'-diapo-6-carotenoate	7.501	312.1725	P
100	Glycosides	7.778	584.2850	P
101	p-Hydroxyphenethyl *trans*-ferulate	7.785	314.1155	P
102	Ethyl menthane carboxamide	7.832	211.1939	P
103	*N*-*trans*-Feruloyloctopamine	7.918	329.1279	P
104	Pristanic acid	7.919	298.2873	P
105	Dodecanamide	7.963	199.1937	P
106	Stearamide	8.013	283.2875	P
107	MG(0:0/18:1(11*Z*)/0:0)	8.021	356.2927	P
108	Lymecycline	8.145	602.2579	P
109	Undecylprodigiosin	8.172	393.2772	P
110	Lyngbyatoxin	8.274	437.3046	P
111	Tributyl phosphate	8.309	266.1648	P
112	Dipyridamole	8.349	504.3148	P
113	Kukoamine D	8.406	530.3129	P
114	D-Pantothenoyl-Lcysteine	8.413	322.1190	P
115	3L,7D,11D-phytanic acid	8.519	312.3031	P
116	8,8-Diethoxy-2,6-dimethyl-2-octanol	8.547	246.2197	P
117	Madlongiside C	8.549	636.3866	P
118	Polysorbate 20	8.620	522.3400	P
119	Dodemorph	8.633	281.2717	P
120	Laserpitin	8.769	450.2621	P
121	Polysorbate 60	8.770	434.2882	P
122	Hexyl heptanoate	8.790	638.2369	P
123	9-Acetoxyfukinanolide	8.870	292.1675	P
124	Austrobailignan 7	8.909	342.1466	P
125	MG(0:0/20:1(11*Z*)/0:0)	8.929	384.3240	P
126	Phytal	8.993	294.2925	P

127	3-Cyclohexyldodecane	9.012	252.2816	P
128	10-hydroperoxy-8*E*,12*Z*octadecadienoic acid	9.016	312.2295	P
129	Isoacitretin	9.041	326.1884	P
130	24-Hydroxycalcitriol	9.094	432.3244	P
131	*Alpha*-CEHC	9.117	278.1517	P
132	(*E*)-1-[4-Hydroxy-3-(3-methyl-1,3-butadienyl)phenyl]-2-(3,5-dihydroxyphenyl)ethylene	9.119	294.1258	P
133	(*Z*)-13-Oxo-9-octadecenoic acid	9.149	296.2346	P
134	Anofinic acid	9.165	204.0787	P
135	Palmitoyl glucuronide	9.183	418.2938	P
136	(*E,E*)-1,6-bis(4-methoxyphenyl)-1,5-hexadiene	9.193	294.1621	P
137	18-Oxocortisol	9.207	376.1884	P
138	Misoprostol	9.208	382.2704	P
139	10-Eicosene	9.330	280.3132	P
140	Pravastatin	9.365	424.2458	P
141	Bioresmethrin	9.371	338.1880	P
142	Chloropyramine	9.372	289.1357	P
143	MG(0:0/16:0/0:0)	9.373	330.2773	P
144	(*E*)-3-(2-Hydroxyphenyl)-2-propenal	9.435	148.0525	P
145	Calendulaglycoside E	9.435	794.4292	P
146	Phenkapton	9.435	375.9355	P
147	(3'x,5'a,9'x,10'b)-O-(3-Hydroxy-6-oxo-7-drimen-11-yl)umbelliferone	9.436	396.1936	P
148	[6]-Gingerdiol 3,5-diacetate	9.436	380.2202	P
149	Asebotoxin II	9.436	408.2504	P
150	Methandriol dipropionate	9.436	416.2912	P
151	MG(0:0/22:6(4*Z*,7*Z*,10*Z*,13*Z*,16*Z*,19*Z*)/0:0)	9.436	402.2755	P
152	(3b,6b,8b,12a)-8,12-Epoxy-7(11)-eremophilene-6-angeloyloxy-8,12-dimethoxy-3-ol	9.437	394.2356	P
153	MG(0:0/18:3(6*Z*,9*Z*,12*Z*)/0:0)	9.469	352.2615	P
154	6*alpha*-Fluoropregn-4-ene-3,20-dione	9.529	332.2145	P
155	Polidocanol	9.554	582.4343	P

156	Piscerythramine	9.587	451.2010	P
157	17-*O*-Acetylnorajmaline	9.594	354.1961	P
158	2-oxophytanic acid	9.620	326.2824	P
159	2-(4-Chloro-3,5-dimethylphenoxy)-*N*-(2-phenyl-2H-benzotriazol-5-yl)-acetamide	9.645	406.1194	P
160	4*beta*-(2-Aminoethylthio)catechin	9.645	365.0924	P
161	Monocrotaline	9.645	325.1526	P
162	Lycopersiconol	9.767	334.2506	P
163	Palmitoyl-EA	9.800	299.2826	P
164	6,8a-Seco-6,8a-deoxy-5-oxoavermectin "2a"aglycone	9.820	586.3508	P
165	2,5-Furandicarboxylic acid	9.945	156.0060	P
166	Arbutin	9.945	272.0898	P
167	Cymorcin monoglucoside	9.945	328.1525	P
168	1-(3-Hydroxy-4-methoxyphenyl)-1,2-ethanediol	9.946	184.0736	P
169	1b,3a,7a,12a-Tetrahydroxy-5bcholanoic acid	9.946	424.2810	P
170	4-Carboxy-2-hydroxy-6-methoxy-6-oxohexa-2,4-dienoate	9.946	216.0271	P
171	Acetyl tributyl citrate	9.946	402.2257	P
172	Asteltoxin	9.946	418.1993	P
173	Kamahine C	9.946	268.1313	P
174	Vanillactic acid	9.946	212.0685	P
175	Forasartan	9.948	416.2417	P
176	Argentine	10.200	406.2021	P
177	DU 122290	10.201	362.1649	P
178	2E-Eicosenoic acid	10.297	310.2871	P
179	Drotaverine	10.384	397.2252	P
180	Drospirenone	10.392	366.2198	P
181	Petromyzonol	10.417	394.3084	P
182	Capsi-amide	10.645	269.2720	P
183	Enalkiren	10.849	656.4293	P
184	Spiramycin	10.896	840.5365	P

185	Cavipetin D	10.897	418.2719	P
186	D-myo-Inositol-1,4,5-triphosphate	10.897	419.9636	P
187	Propinol adenylate	10.897	403.0902	P
188	Azoxystrobin	10.898	403.1163	P
189	Sorbitan palmitate	10.898	402.2984	P
190	Ganodermic acid TQ	11.095	510.3341	P
191	Camptothecin	11.240	348.1103	P
192	Phenolic phosphate	11.767	174.0081	P
193	12-Ketodeoxycholic acid	12.259	390.2773	P
194	PC(16:0/18:1(9*Z*))[*S*]	12.271	760.5856	P
195	Dioctyl hexanedioate	12.281	370.3085	P
196	DG(14:0/22:1(13*Z*)/0:0)	14.116	622.5545	P
197	PE(P-16:0/18:2(9*Z*,12*Z*))	15.818	699.5210	P

[a] – retention time [min]

[b] –compound detection in positive (P) or in negative (N) ionization mode.
