# Supplementary material for: Biological Properties of the Mucus and Eggs of Helix aspersa Müller as a Potential Cosmetic and Pharmaceutical Raw Material: A Preliminary Study
Source: Int J Mol Sci. 2024 Sep 15;25(18):9958. doi: 10.3390/ijms25189958 (PMC11432642; doi:10.3390/ijms25189958)
Supplement: Supplementary file 1 [file ijms-25-09958-s001.zip › Herman Anna - Table S12.pdf]

Table S12. Compounds identified in metanolic extract of lyophilized mucus of organic *Helix aspersa* snail using LC-MS.

No	Metabolite	RT[a] [min]	Mass [*m/z*]	Detection mode[b]
1	Tetrachlorobisphenol A	0.256	363.9610	N
2	Trifluoroacetic acid	0.256	113.9928	N
3	Sulfadiazine	0.257	250.0536	N
4	Ginkgolide C	0.265	440.1311	N
5	2-methyl-3-oxopropanoic acid	0.266	102.0317	N
6	Dulcitol	0.267	182.0792	N
7	Glycolaldehyde	0.267	60.0211	N
8	L-Rhamnulose	0.267	164.0687	N
9	Acrylic acid	0.268	72.0211	N
10	Maltitol	0.270	344.1320	N
11	2-Hydroxypropylphosphonate	0.273	140.0238	N
12	Epibatidine	0.274	208.0757	N
13	Estradiol-17β 3-sulfate	0.276	352.1350	N
14	(2Z,4'Z)-2-(5-Methylthio-4-penten-2-ynylidene)-1,6-dioxaspiro[4.4]non-3-ene	0.279	234.0719	N
15	Dimethyl carbonate	0.281	90.0317	N
16	Glyceric acid	0.284	106.0263	N
17	Flupropanate	0.299	145.9993	N
18	2-Benzothiazolesulfonamide	0.300	213.9867	N
19	2-[(5-Methylsulfinyl)-4-penten-2-ynylidene]-1,6-dioxaspiro[4.4]non-3-ene	0.319	250.0666	N
20	Inabenfide	0.370	338.0822	N
21	*N*-Valerylglycine methyl ester	2.379	173.1053	N
22	*N*-n-Hexanoylglycine methyl ester	3.464	187.1211	N
23	Capryloylglycine	4.101	201.1366	N
24	Methyl *N*-(amethylbutyryl)glycine	4.306	188.1050	N
25	*N*-heptanoyl-homoserine lactone	4.455	213.1365	N
26	Ethyl 3-(*N*butylacetamido)propionate	4.650	215.1524	N

27	D-Ribose 1-diphosphate	5.617	293.9900	N
28	Blumenol C glucoside	5.730	372.2144	N
29	Ethiprole	5.804	395.9832	N
30	Zingerone	6.229	194.0945	N
31	Bismuth subsalicylate	6.707	361.9975	N
32	Eremopetasinorol	6.771	208.1463	N
33	Nordihydrocapsiate	6.832	294.1832	N
34	3-Hydroxy-6,8-dimethoxy-7(11)-eremophilen-12,8-olide	7.031	310.1777	N
35	BILA 2185BS	7.039	618.3250	N
36	Methyl 2-benzamidoacetate	7.060	193.0738	N
37	(*S,Z*)-Lyratol acetate	7.115	194.1306	N
38	3b-Allotetrahydrocorticosterone	7.118	350.2459	N
39	Lauryl hydrogen sulfate	7.280	266.1551	N
40	Losartan	7.314	422.1623	N
41	L-Tyrosine methyl ester	7.343	195.0896	N
42	Dinoterb	7.579	240.0748	N
43	(+)-Prosopinine	7.701	313.2617	N
44	*N*-Undecylbenzenesulfonic acid	7.728	312.1759	N
45	2-Dodecylbenzenesulfonic acid	8.162	326.1914	N
46	Sodium Tetradecyl Sulfate	8.201	294.1864	N
47	Docusate	8.341	422.2338	N
48	Gemfibrozil	8.957	250.1571	N
49	Furmecyclox	9.280	251.1523	N
50	3-Oxochola-4,6-dien-24-oic acid	10.249	370.2508	N
51	(5b,7a,12a)-2-(3-methoxyphenyl)-2-oxoethyl ester-7,12-dihydroxy-cholan-24-oic acid	10.490	540.3452	N
52	Methyl tetradecanoate	10.500	242.2246	N
53	5-Dodecyldihydro-2(3H)-furanone	10.503	254.2248	N
54	Piritramide	10.548	430.2739	N
55	DG(18:1(11*Z*)/22:5(4*Z*,7*Z*,10*Z*,13*Z*,16*Z*)/0:0)	10.551	668.5403	N

56	DG(20:3(5*Z*,8*Z*,11*Z*)/22:6(4*Z*,7*Z*,10*Z*,13*Z*,16*Z*,19*Z*)/0:0)	10.551	690.5221	N
57	MG(0:0/16:0/0:0)	10.551	330.2769	N
58	Schidigeragenin B	10.551	428.2943	N
59	(3*R*,2'*S*)-Myxol 2'-(2,4-di-*O*-methyl-α-L-fucoside)	10.552	758.5094	N
60	Auricularine	10.554	494.3406	N
61	Enalkiren	10.844	656.4282	N
62	3-Hydroxy-2-(4-morpholinylmethyl)estra-1,3,5(10)-trien-17-one	10.950	369.2300	N
63	Butroxydim	11.271	399.2409	N
64	Adlupone	11.363	482.3395	N
65	Drotaverine	11.451	397.2254	N
66	DG(20:5(5*Z*,8*Z*,11*Z*,14*Z*,17*Z*)/24:1(15*Z*)/0:0)	11.507	724.6029	N
67	MG(18:0/0:0/0:0)	11.508	358.3081	N
68	(22*E*,24*R*)-Stigmasta-4,22-diene-3,6-dione	11.963	424.3340	N
69	5-Heptadecyl-1,3-benzenediol	11.997	348.3027	N
70	24-Acetyl-25-cinnamoylvulgaroside	12.066	608.3355	N
71	(3*beta*,22*E*,24*R*)-3-Hydroxyergosta-5,8,22-trien-7-one	12.502	410.3185	N
72	Propyl 1-(propylsulfinyl)propyldisulfide	13.963	240.0667	N
1	L-Homocysteic acid	0.238	183.0207	P
2	Norvaline	0.255	117.0788	P
3	Edetate	0.258	292.0921	P
4	6-Hydroxymusizin 8-*O*-b-D-glucopyranoside	0.261	394.1261	P
5	Dulcitol	0.265	182.0790	P
6	Chlorphentermine	0.271	183.0822	P
7	Isradipine	0.271	371.1475	P
8	L-Valine	0.274	117.0789	P
9	(4-Hydroxybenzoyl)choline	0.279	224.1285	P
10	4'-Hydroxy-3',5,6,7,8-pentamethoxyflavone	0.279	388.1173	P
11	Artonol B	0.279	420.1223	P
12	4-Guanidinobutanoic acid	0.281	145.0850	P

13	Coriandrin	0.281	230.0573	P
14	(2*R**,3*R**)-1,2,3-Butanetriol	0.371	106.0630	P
15	(2*R*,3*R*,4*R*)-2-Amino-4-hydroxy-3-methylpentanoic acid	0.398	147.0895	P
16	Trolamine	0.398	149.1052	P
17	*R*-2-Hydroxy-3-methylbutanoic acid 3-Methylbutanoyl	0.644	202.1205	P
18	2-Amino-4-hydroxy-6-(hydroxymethyl)-7,8-dihydropteridine	0.855	195.0761	P
19	1-nitroheptane	0.975	145.1104	P
20	Pseudoecgonine	1.469	185.1051	P
21	2,5-Dihydro-2,4,5-trimethyloxazole	1.487	113.0841	P
22	2E-Decenedioic acid	2.327	200.1050	P
23	*N*-Valerylglycine methyl ester	2.377	173.1052	P
24	Retronecine	2.378	155.0946	P
25	Propionyl-L-carnitine	2.483	218.1393	P
26	2,3-Dimethyl-2-cyclohexen-1-one	2.560	124.0888	P
27	DL-2-amino-octanoic acid	2.561	159.1260	P
28	8-Acetoxy-4-acoren-3-one	2.814	278.1878	P
29	5-Heptyltetrahydro-2-oxo-3-furancarboxylic acid	2.950	228.1364	P
30	3-hydroxytetradecanedioic acid	3.033	274.1782	P
31	Tussilagine	3.077	199.1211	P
32	Sedanonic acid	3.125	210.1255	P
33	Platydesminium	3.143	274.1431	P
34	Pyracarbolid	3.144	217.1107	P
35	Wine lactone	3.291	166.0996	P
36	Hexamethylene bisacetamide	3.293	200.1525	P
37	2-Amino-3,4,8-trimethyl-3H-imidazo[4,5-f]quinoxaline	3.386	227.1161	P
38	1,4-Ipomeadiol	3.464	170.0943	P
39	o-Xylene	3.464	106.0781	P
40	(4-Methylphenyl)acetaldehyde	3.465	134.0732	P
41	2-Isopropyl-1,4-benzenediol	3.465	152.0836	P

42	Prolyl-Valine	3.472	214.1325	P
43	Istamycin C1	3.584	431.2731	P
44	(*R*)-3-Hydroxy-5-phenylpentanoic acid	3.613	194.0941	P
45	2,3-Dihydro-5-(5-methyl-2-furanyl)-1H-pyrrolizine	3.613	187.0999	P
46	4-Vinylcyclohexene	3.614	108.0939	P
47	Methoxamine	3.614	211.1209	P
48	Platydesmine	3.616	259.1210	P
49	Slaframine	3.619	198.1370	P
50	Pirbuterol	3.622	240.1473	P
51	1,2,3,4,5,6-Hexahydro-5-methyl-7Hcyclopenta[b]pyridin-7-one	3.652	151.0999	P
52	Jasmine ketolactone	3.675	208.1100	P
53	Netilmicin	3.735	475.2994	P
54	*S*-(2-Methylbutanoyl)-dihydrolipoamide	3.810	291.1335	P
55	*N*-heptanoyl-homoserine lactone	3.837	213.1366	P
56	Ethyl 3-(*N*butylacetamido)propionate	3.847	215.1529	P
57	Monomenthyl succinate	3.849	256.1676	P
58	Geranyl acetoacetate	3.850	238.1570	P
59	5-(1-Pyrrolidinylmethyl)-2-furanmethanol	3.890	181.1104	P
60	2-Methyl-1-phenyl-2-propanyl acetate	3.923	192.1151	P
61	*N*-(3-oxo-octanoyl)-homoserine lactone	3.983	241.1316	P
62	Tributyrin	4.017	302.1730	P
63	Alanyl-Valine	4.023	188.1162	P
64	n1,n8-diacetylspermidine	4.032	229.1792	P
65	(*E*)-1-Cinnamoylpyrrolidine	4.033	201.1148	P
66	Isopentenyladenine-9-*N*-glucoside	4.039	363.1911	P
67	Acetyltropine	4.097	183.1261	P
68	Valyl-Lysine	4.098	245.1743	P
69	n-decanohydroxamic acid	4.182	187.1573	P
70	*N*-[2-(4-Hydroxyphenyl)ethyl]benzamide	4.257	241.1107	P

71	Talbutal	4.259	252.1474	P
72	Phlorin	4.271	288.0846	P
73	Octyl gallate	4.292	282.1467	P
74	Humulinic acid A	4.298	266.1517	P
75	Avenic acid A	4.302	322.1392	P
76	Mukaadial	4.388	266.1520	P
77	Oseltamivir	4.388	312.2054	P
78	Triethylenemelamine	4.402	204.1129	P
79	PE(18:4(6*Z*,9*Z*,12*Z*,15*Z*)/22:6(4*Z*,7*Z*,10*Z*,13*Z*,16*Z*,19*Z*))	4.451	783.4822	P
80	2-(4-Methylphenyl)-2-propanol	4.455	150.1045	P
81	5-Phenylvaleric acid	4.455	178.0994	P
82	1-Octen-3-yl glucoside	4.498	290.1730	P
83	(*E*)-3-decen-1-ol	4.549	156.1514	P
84	Diethofencarb	4.551	267.1472	P
85	Ethyl decanoate	4.552	200.1778	P
86	Flumetover	4.553	367.1391	P
87	Imiquimod	4.636	240.1364	P
88	Isoleucyl-Lysine	4.650	259.1896	P
89	1,2,3-Tris(1-ethoxyethoxy)propane	4.672	308.2201	P
90	2-Hexenoylcholine	4.674	200.1652	P
91	1-Methyl-2-propylbenzene	4.682	134.1096	P
92	C12:1n-7	4.686	198.1621	P
93	*Gamma*-CEHC	4.687	248.1412	P
94	11-Hydroxy-9-tridecenoic acid	4.693	228.1728	P
95	Ruscopine	4.695	306.2042	P
96	Dyclonine	4.724	289.2043	P
97	(5*R*)-5-Hydroxyhexanoic acid	4.732	132.0786	P
98	1-Phenyl-6,7-dihydroxyisochroman	4.732	242.0943	P
99	2-Ethylacrylylcarnitine	4.732	244.1551	P

100	2-Phenylbutyric acid	4.732	164.0839	P
101	4-(*N*-Maleimido)phenyltrimethyl ammonium	4.738	231.1140	P
102	Alanyl-Isoleucine	4.781	202.1319	P
103	Methyl 7-epi-12-hydroxyjasmonate glucoside	4.792	402.1890	P
104	*N*-Isobutyl-2,4,8,10,12-tetradecapentaenamide	4.803	273.2094	P
105	Methyl 3-(2,3-dihydroxy-3-methylbutyl)-4-hydroxybenzoate	4.812	463.2415	P
106	Pinidine	4.847	139.1362	P
107	1,3-Diphenyltetramethyldisiloxane	4.963	286.1207	P
108	1,1,2-Triphenylpropane	5.023	272.1560	P
109	Pentosidine	5.038	378.2017	P
110	Sterebin E	5.076	338.2451	P
111	Z-Arg-Arg-NHMec	5.079	621.3043	P
112	(S)-3-Octanol glucoside	5.102	292.1884	P
113	(-)-*trans*-Carveol glucoside	5.136	314.1732	P
114	Gibberellin A105	5.137	330.1464	P
115	7,8-Dihydrovomifoliol 9-[rhamnosyl-(1->6)-glucoside]	5.160	534.2679	P
116	Elaeokanine C	5.162	211.1576	P
117	Toxin T2 tetrol	5.244	298.1418	P
118	Cyclonormammein	5.273	374.1726	P
119	4,11,13,15-Tetrahydroridentin B	5.293	268.1674	P
120	Artabsinolide A	5.310	280.1311	P
121	(-)-Hygroline	5.327	143.1312	P
122	1,1'-(Tetrahydro-6ahydroxy-2,3a,5-trimethylfuro[2,3-d]-1,3-dioxole-2,5-diyl)bisethanone	5.364	258.1104	P
123	Jasmolone glucoside	5.372	342.1681	P
124	Allopumiliotoxin 267A	5.404	267.2199	P
125	Valyl-Valine	5.429	216.1475	P
126	AF Toxin II	5.432	324.1574	P
127	Dulciol C	5.477	482.2320	P
128	Hydrocortisone succinate	5.482	462.2251	P

129	Corchoionol C 9-glucoside	5.484	386.1940	P
130	Discadenine	5.496	304.1649	P
131	(*E,E,E*)-*N*-(2-Methylpropyl)hexadeca-2,6,8-trien-10-ynamide	5.505	301.2408	P
132	Dimethylbenzyl carbinyl hexanoate	5.653	248.1775	P
133	Terazosin	5.656	387.1887	P
134	*N*-Jasmonoylisoleucine	5.693	323.2096	P
135	(2xi,6xi)-7-Methyl-3-methylene-1,2,6,7-octanetetrol	5.701	204.1365	P
136	Hexanal octane-1,3-diol acetal	5.706	228.2091	P
137	2-Methylundecanal	5.727	184.1828	P
138	Blumenol C *O*-[rhamnosyl-(1->6)-glucoside]	5.740	518.2731	P
139	Avocadienofuran	5.766	246.1984	P
140	(5alpha,10*alpha*)-3,7(11)-Eudesmadien-2-one	5.768	218.1668	P
141	Volicitin	5.770	422.2761	P
142	NAc-FnorLRF-amide	5.772	622.3565	P
143	Fluspirilene	5.808	475.2418	P
144	19(*R*)-hydroxy-PGE2	5.829	368.2199	P
145	1-(2,4,6-Trimethoxyphenyl)-1,3-butanedione	5.859	252.0996	P
146	3-Hydroxy-3-methyl-2-oxo-pentanoic acid	5.859	146.0580	P
147	Glaucamine	5.859	385.1528	P
148	Glaudine	5.859	399.1684	P
149	Sanshodiol	5.860	358.1419	P
150	Symphytine	5.861	381.2159	P
151	Tetrahydrocurcumin	5.862	372.1574	P
152	C14:1n-9	5.877	226.1934	P
153	Eriojaposide A	5.878	502.2414	P
154	Canavalioside	5.942	546.2680	P
155	Norerythrostachaldine	5.942	407.2661	P
156	(+/-)-*N,N*-Dimethyl menthyl succinamide	6.017	168.1879	P
157	Sterebin B	6.017	352.2251	P

158	Capsoside A	6.019	694.3772	P
159	15-Acetoxyscirpene-3,4-diol 4-*O*-a-D-glucopyranoside	6.026	486.2099	P
160	(*E,E*)-2,4-Decadienoic isobutylamide	6.043	223.1937	P
161	Capsaicin	6.063	305.1982	P
162	Homodihydrojasmone	6.070	180.1515	P
163	Lauroyl diethanolamide	6.073	287.2466	P
164	2-Hydroxyestrone	6.079	286.1570	P
165	10-nitro,9*Z*,12*Z*octadecadienoic acid	6.125	325.2255	P
166	20-COOH-Leukotriene B4	6.145	366.2039	P
167	(*Z*)-6-Nonenal	6.151	140.1202	P
168	Penbutolol	6.180	291.2201	P
169	Hydroxy-*alpha*-sanshool	6.204	263.1887	P
170	7,10-Hexadecadienoic acid	6.225	252.2090	P
171	4,4-Difluoropregn-5-ene-3,20-dione	6.281	350.2069	P
172	Gravelliferone	6.308	298.1569	P
173	10-hydroperoxy-8*E*,12*Z*octadecadienoic acid	6.315	312.2305	P
174	Eucalyptol	6.335	154.1358	P
175	(+)-Prosopinine	6.347	313.2619	P
176	Cuscohygrine	6.373	224.1890	P
177	15-keto-Prostaglandin E2	6.513	350.2070	P
178	1-Hydroxyacorenone	6.518	250.1567	P
179	5-*O*-β-Dmycaminosyltylonolide	6.559	597.3491	P
180	Formylfusarochromanone	6.580	320.1389	P
181	17-Methylandrosta-2,4-dieno[2,3-d]isoxazol-17*beta*-ol	6.592	327.2199	P
182	Chaksine	6.606	450.2969	P
183	Lilac alcohol	6.613	170.1307	P
184	Corchorifatty acid F	6.637	328.2250	P
185	Momilactone B	6.638	330.1832	P
186	Musabalbisiane C	6.643	568.2498	P

187	10-Hydroxy-2,8-decadiene-4,6-diynoic acid	6.665	176.0474	P
188	Monoisobutyl phthalic acid	6.665	222.0894	P
189	C16 Sphinganine	6.684	273.2670	P
190	Sphinganine	6.684	301.2983	P
191	Dihydrocapsaicin	6.692	307.2153	P
192	2-Furanmethanol	6.697	98.0367	P
193	Glyceollidin II	6.697	340.1313	P
194	2*alpha*,3*alpha*-(Difluoromethylene)-5*alpha*-androstan-17*beta*-ol acetate	6.713	366.2361	P
195	Glicoisoflavanone	6.715	384.1574	P
196	2-Tetradecanone	6.716	212.2142	P
197	Cerberoside	6.734	858.4267	P
198	1-Isomangostin hydrate	6.735	428.1835	P
199	5-(2,3-Dihydroxy-3-methylbutyl)-4-(3,4-epoxy-4-methylpentanoyl)-3,4-dihydroxy-2-isopentanoyl-2-cyclopenten-1-one	6.735	412.2102	P
200	Tigloidine	6.755	223.1575	P
201	1*alpha*,3*beta*,22*R*Trihydroxyergosta-5,24*E*dien-26-oic acid 3-*O*-b-D-glucoside 26-*O*-[b-Dglucosyl-(1->2)-b-D-glucosyl] ester	6.757	946.4786	P
202	Dihomo-γ-linolenoyl-EA	6.757	349.2966	P
203	Phytosphingosine	6.757	317.2933	P
204	2-Methoxy-estradiol-17β 3-glucuronide	6.758	478.2183	P
205	Deacetylnomilin	6.758	472.2096	P
206	Ximelagatran	6.758	473.2632	P
207	5-Dodecyldihydro-2(3H)-furanone	6.779	254.2246	P
208	Mycalamide B	6.781	517.2889	P
209	2,2-Dibutyl-3-(4-methoxyphenyl)-4-methyl-2H-1-benzopyran-7-ol acetate	6.785	422.2441	P
210	3',4',5'-Trimethoxycinnamyl alcohol acetate	6.785	266.1155	P
211	Cinegalline	6.785	430.2106	P
212	Erysothiopine	6.785	407.1024	P
213	Melleolide	6.785	400.1888	P
214	Porson	6.785	386.1731	P

215	Styrene	6.786	104.0626	P
216	Panaquinquecol 1	6.788	292.2040	P
217	16-hydroxy hexadecanoic acid	6.790	272.2353	P
218	Canescein	6.804	566.2706	P
219	Funtumine	6.846	317.2720	P
220	Petasinine	6.857	239.1525	P
221	Phenethyl decanoate	6.860	276.2090	P
222	(*S*)-Nerolidol 3-*O*-[a-LRhamnopyranosyl-(1->4)-a-Lrhamnopyranosyl-(1->2)-b-Dglucopyranoside]	6.865	676.3673	P
223	1-Methyl-2-nonyl-4(1H)-quinolinone	6.895	285.2091	P
224	Pumiliotoxin 251D	6.895	251.2251	P
225	Genipin 1-*beta*gentiobioside	6.900	550.1897	P
226	3-Hydroxy-6,8-dimethoxy-7(11)-eremophilen-12,8-olide	6.906	310.1783	P
227	Zizybeoside II	6.920	594.2168	P
228	Chrycolide	6.945	232.0184	P
229	Contignasterol	6.949	508.3387	P
230	Arachidonyl Trifluoromethyl Ketone	6.975	356.2318	P
231	3'-Hydroxy-HT2 toxin	6.976	440.2048	P
232	Plantaricin BN	6.989	484.2309	P
233	Muricatacin	6.993	284.2342	P
234	Nonyl octanoate	6.993	270.2564	P
235	Coccinin	7.002	528.2574	P
236	Palmitic amide	7.033	255.2563	P
237	Acetyl Tyrosine Ethyl Ester	7.036	251.1154	P
238	BILA 2185BS	7.044	618.3257	P
239	Cyclotetradecane	7.061	196.2191	P
240	*N*-Methylmescaline	7.076	225.1366	P
241	Myxochelin A	7.087	404.1584	P
242	Acebutolol	7.101	336.2060	P
243	Spiroxamine	7.102	297.2667	P

244	6*alpha*,9-Difluoro-11*beta*-hydroxypregn-4-ene-3,20-dione	7.107	366.2017	P
245	2,6-Di-tert-butyl-4-ethylphenol	7.113	234.1983	P
246	2-Methoxyestradiol-3-methylether	7.123	316.2023	P
247	Finaconitine	7.127	630.3150	P
248	Piperolein B	7.173	343.2147	P
249	Coutaric acid	7.174	349.2011	P
250	Paucin	7.176	468.1995	P
251	Marimastat	7.180	331.2123	P
252	Z-Gly-Pro-Leu-Gly-Pro	7.190	573.2784	P
253	10,16-dihydroxy-palmitic acid	7.192	288.2296	P
254	(9*Z*,11*R*,12*S*,13*S*,15*Z*)-12,13-Epoxy-11-hydroxy-9,15-octadecadienoic acid	7.229	310.2145	P
255	Pipercitine	7.239	349.3329	P
256	Armillaric acid	7.243	416.1832	P
257	Cincassiol B	7.244	400.2100	P
258	1-Phenyl-1,3-dodecanedione	7.250	274.1932	P
259	3-Methyl-*alpha*-ionyl acetate	7.251	250.1934	P
260	2,3-Dinor-11b-PGF2a	7.252	326.2092	P
261	*N*-Dealkylatedtolterodine	7.252	283.1932	P
262	9-Decenoylcholine	7.283	256.2276	P
263	Bleekerine	7.317	409.1758	P
264	Lentiginosine	7.331	157.1104	P
265	Estrane-3α,17α-diol	7.381	278.2248	P
266	Capryloylglycine	7.386	201.1372	P
267	3-(5,6,6-Trimethylbicyclo[2.2.1]hept-1-yl)cyclohexanol	7.400	236.2142	P
268	Avocadenofuran	7.406	248.2141	P
269	Physagulin C	7.439	542.2505	P
270	Morfamquat	7.455	466.2570	P
271	9-HOTE	7.456	294.2196	P
272	Picrasin C	7.461	422.2304	P

273	2,2-Dimethyl-3,4-bis(4-methoxyphenyl)-2H-1-benzopyran-7-ol acetate	7.462	430.1780	P
274	*Alpha*-Methylstyrene	7.462	118.0786	P
275	Armillaripin	7.462	414.2029	P
276	Austalide L	7.462	428.2201	P
277	DHAP(18:0)	7.462	436.2599	P
278	Erythroskyrin	7.462	455.2308	P
279	Cyclocalopin F	7.463	294.1104	P
280	Methyl (9*Z*)-10'-oxo-6,10'-diapo-6-carotenoate	7.501	312.1726	P
281	2,4,12-Octadecatrienoic acid piperidide	7.513	345.3018	P
282	Norpropoxyphene	7.515	325.2040	P
283	2-Hexadecanone	7.642	240.2454	P
284	Palmitoyl-EA	7.724	299.2827	P
285	1-(4-Amino-2-methylpyrimid-5-ylmethyl)-3-(*beta*hydroxyethyl)-2-methylpyridinium	7.748	259.1551	P
286	2,2,6,6-Tetramethyl-4-piperidinone	7.776	155.1311	P
287	6,10,14-Trimethyl-5,9,13-pentadecatrien-2-one	7.777	262.2289	P
288	Gabapentin	7.777	171.1260	P
289	Glycosides	7.777	584.2842	P
290	Ethyl (4*Z*)-4,7-octadienoate	7.785	168.1150	P
291	Imperialine	7.788	429.3248	P
292	Methyloctatropine	7.792	282.2435	P
293	Phlegmarine	7.836	250.2405	P
294	Methadone	7.874	309.2091	P
295	Zucchini factor B	7.906	663.4301	P
296	Pristanic acid	7.922	298.2865	P
297	Elaiophylin	7.944	1024.593<br>2	P
298	2,4,12-Octadecatrienoic acid isobutylamide	7.951	333.3016	P
299	(3a,5b)-24-oxo-24-[(2-sulfoethyl)amino]cholan-3-yl-b-Dglucopyranosiduronic acid	7.952	659.3342	P
300	Methyprylon	7.954	183.1260	P

301	(10*S*)-Juvenile hormone III diol	7.957	284.2005	P
302	Dodecanamide	7.960	199.1936	P
303	Asparagoside D	7.963	902.4873	P
304	3-Ethyl-2-hydroxy-4-methyl-2-cyclopenten-1-one	7.964	140.0837	P
305	Scopoloside II	8.003	770.4093	P
306	Stearamide	8.013	283.2878	P
307	2-Methoxyestrone 3-sulfate	8.016	380.1295	P
308	Leucomycin A9	8.017	743.4095	P
309	MG(0:0/18:1(11*Z*)/0:0)	8.017	356.2929	P
310	Myriocin	8.032	401.2777	P
311	Corchorusoside B	8.034	682.3568	P
312	2,2,7,7-Tetramethyl-1,6-dioxaspiro[4.4]non-3-ene	8.061	182.1308	P
313	Convallatoxin	8.094	550.2779	P
314	Dihydro-5-(2-octenyl)-2(3H)-furanone	8.118	196.1465	P
315	Solanocardinol	8.123	431.3404	P
316	Cortolone	8.125	366.2416	P
317	Lymecycline	8.141	602.2571	P
318	Undecylprodigiosin	8.170	393.2786	P
319	Linalyl propionate	8.173	210.1621	P
320	5,8-tetradecadienoic acid	8.182	224.1776	P
321	Lyngbyatoxin	8.272	437.3043	P
322	Tetrahymanol	8.272	442.4161	P
323	Erinacine G	8.273	464.2412	P
324	(*E*)-2-Butenal	8.275	70.0417	P
325	1,3-Octadiene	8.276	110.1095	P
326	17*beta*-Acetamidoandrost-4-en-3-one	8.276	329.2352	P
327	6-Oxocineole	8.276	168.1151	P
328	Nitramine	8.276	169.1463	P
329	p-Mentha-1,3,5,8-tetraene	8.277	132.0940	P

330	Santene	8.277	122.1094	P
331	2,2,7,7-Tetramethyl-1,6-dioxaspiro[4.4]nona-3,8-diene	8.278	180.1154	P
332	Tributyl phosphate	8.306	266.1649	P
333	Biperiden	8.319	311.2246	P
334	(*Z*)-9-Cycloheptadecen-1-one	8.330	250.2298	P
335	Isopentylideneisopentylamine	8.344	155.1675	P
336	1-Methyl-1,3-cyclohexadiene	8.345	94.0783	P
337	*cis*-1,2-Dihydro-3-ethylcatechol	8.346	140.0839	P
338	Tropine	8.346	141.1154	P
339	Lupinine	8.350	169.1468	P
340	Coniine	8.354	127.1361	P
341	Homodihydrocapsaicin	8.355	321.2293	P
342	2,3,6-Trimethylphenol	8.362	136.0889	P
343	Isometheptene	8.362	141.1518	P
344	2-Decylfuran	8.374	208.1829	P
345	Kukoamine D	8.403	530.3124	P
346	B 823-08	8.409	353.0825	P
347	Clavamycin B	8.410	362.1423	P
348	Triphenyl phosphate	8.410	326.0710	P
349	Methyl 2*E*,4*Z*-hexadecadienoate	8.412	266.2247	P
350	LysoPC(15:0)	8.435	482.3245	P
351	Carpaine	8.440	478.3766	P
352	Methyl 2-octynoate	8.455	154.0995	P
353	Dicyclomine	8.470	309.2667	P
354	12*S*-HEPE	8.513	318.2196	P
355	3L,7D,11D-phytanic acid	8.513	312.3029	P
356	Decylubiquinol	8.515	324.2304	P
357	Mycinamicin VIII	8.524	505.3391	P
358	*N*-(14-Methylhexadecanoyl)pyrrolidine	8.527	323.3189	P

359	Dodemorph	8.540	281.2720	P
360	8,8-Diethoxy-2,6-dimethyl-2-octanol	8.546	246.2195	P
361	Protoprimulagenin A 3-[rhamnosyl-(1->4)-rhamnosyl-(1->4)-[rhamnosyl-(1->2)]-glucosyl-(1->?)-glucuronide]	8.546	1234.628 3	P
362	(3a,5b,7a,12a)-24-[(carboxymethyl)amino]-1,12-dihydroxy-24-oxocholan-3-yl-b-Dglucopyranosiduronic acid	8.548	641.3412	P
363	Oleyl alcohol	8.560	268.2767	P
364	5*beta*-Gonane	8.595	232.2194	P
365	Coprocholic acid	8.604	450.3344	P
366	Polysorbate 20	8.617	522.3403	P
367	Isopimara-7,15-dienol	8.686	288.2452	P
368	Tecostanine	8.743	183.1625	P
369	TG(8:0/8:0/8:0)	8.767	470.3590	P
370	Polysorbate 60	8.769	434.2884	P
371	Laserpitin	8.770	450.2619	P
372	Hexyl heptanoate	8.789	638.2362	P
373	Ikshusterol	8.820	430.3797	P
374	Oleoyl Ethanolamide	8.849	325.2984	P
375	PS(18:0/22:5(7*Z*,10*Z*,13*Z*,16*Z*,19*Z*))	8.857	837.5560	P
376	9-Acetoxyfukinanolide	8.866	292.1674	P
377	*N*-Methylpelletierine	8.882	155.1312	P
378	Tranexamic acid	8.883	157.1103	P
379	*trans*-9, *trans*-11-octadecadienoic acid; C18:2n-7,9	8.883	280.2403	P
380	13-heptadecyn-1-ol	8.884	252.2456	P
381	2-Pentylfuran	8.884	138.1046	P
382	*cis*-Cetoleic acid	8.925	338.3187	P
383	MG(0:0/20:1(11*Z*)/0:0)	8.925	384.3240	P
384	(3*S*,6*E*,10*E*)-1,6,10,14-Phytatetraen-3-ol	8.957	290.2612	P
385	20,21,21-Trifluoro-3-methoxy-19-nor-17*alpha*-pregna-1,3,5(10),20-tetraen-17-ol	8.992	366.1806	P
386	3-Cyclohexyldodecane	9.014	252.2817	P

387	(*E,E*)-1,6-bis(4-methoxyphenyl)-1,5-hexadiene	9.040	294.1620	P
388	Isoacitretin	9.040	326.1885	P
389	Annoglabasin F	9.076	378.2408	P
390	24-Hydroxycalcitriol	9.088	432.3238	P
391	*Alpha*-CEHC	9.112	278.1520	P
392	22-Oxo-docosanoate	9.137	354.3135	P
393	*N*-n-Hexanoylglycine methyl ester	9.141	187.1208	P
394	MG(0:0/22:2(13*Z*,16*Z*)/0:0)	9.170	410.3396	P
395	Palmitoyl glucuronide	9.182	418.2934	P
396	Armillarivin	9.188	384.1937	P
397	(6*beta*,7*alpha*,12*beta*,13*beta*)-7-Hydroxy-11,16-dioxo-8,14-apianadien-22,6-olide	9.191	384.1938	P
398	1-(3-Hydroxy-4-methoxyphenyl)-1,2-ethanediol	9.208	184.0737	P
399	18-Oxocortisol	9.209	376.1883	P
400	Ampalex	9.209	241.1205	P
401	Misoprostol	9.210	382.2701	P
402	Tsangane L 3-glucoside	9.211	374.2308	P
403	β-Caryophyllene Alcohol	9.239	222.1984	P
404	Linoleoyl Ethanolamide	9.295	323.2825	P
405	10-Eicosene	9.328	280.3129	P
406	Pravastatin	9.365	424.2459	P
407	Chloropyramine	9.368	289.1358	P
408	Bioresmethrin	9.371	338.1887	P
409	MG(0:0/16:0/0:0)	9.373	330.2767	P
410	Acidissiminol epoxide	9.392	409.2252	P
411	MG(0:0/22:6(4*Z*,7*Z*,10*Z*,13*Z*,16*Z*,19*Z*)/0:0)	9.429	402.2752	P
412	(3b,6b,8b,12a)-8,12-Epoxy-7(11)-eremophilene-6-angeloyloxy-8,12-dimethoxy-3-ol	9.430	394.2353	P
413	(3'x,5'a,9'x,10'b)-O-(3-Hydroxy-6-oxo-7-drimen-11-yl)umbelliferone	9.431	396.1934	P
414	Asebotoxin II	9.432	408.2498	P
415	Methandriol dipropionate	9.433	416.2909	P

416	6-Hydroxy-8-docosanone	9.434	340.3338	P
417	[6]-Gingerdiol 3,5-diacetate	9.435	380.2202	P
418	Phenkapton	9.435	375.9361	P
419	(*E*)-3-(2-Hydroxyphenyl)-2-propenal	9.436	148.0525	P
420	Calendulaglycoside E	9.436	794.4330	P
421	Thiethylperazine	9.437	399.1787	P
422	Iriomoteolide 1a	9.450	506.3220	P
423	Tsugarioside B	9.451	616.4336	P
424	MG(0:0/18:3(6*Z*,9*Z*,12*Z*)/0:0)	9.464	352.2614	P
425	Thromboxane	9.486	296.3078	P
426	17*beta*-Hydroxy-7*alpha*methylandrost-4-en-3-one propionate	9.489	358.2509	P
427	Trimethaphan	9.494	365.1706	P
428	Galbanic acid	9.498	398.2090	P
429	17-*O*-Acetylnorajmaline	9.540	354.1960	P
430	Polidocanol	9.554	582.4344	P
431	Piscerythramine	9.594	451.2012	P
432	2-(4-Chloro-3,5-dimethylphenoxy)-*N*-(2-phenyl-2H-benzotriazol-5-yl)-acetamide	9.644	406.1195	P
433	4*beta*-(2-Aminoethylthio)catechin	9.645	365.0925	P
434	Monocrotaline	9.645	325.1526	P
435	Lycopersiconol	9.769	334.2498	P
436	6,8a-Seco-6,8a-deoxy-5-oxoavermectin "2a"aglycone	9.824	586.3503	P
437	MG(0:0/22:1(13*Z*)/0:0)	9.839	412.3549	P
438	Ganoderic acid *beta*	9.853	500.3115	P
439	1b,3a,7a,12a-Tetrahydroxy-5bcholanoic acid	9.944	424.2810	P
440	Acetyl tributyl citrate	9.944	402.2254	P
441	Arbutin	9.944	272.0899	P
442	Asteltoxin	9.944	418.1989	P
443	Cymorcin monoglucoside	9.944	328.1525	P
444	Kamahine C	9.944	268.1312	P

445	4-Carboxy-2-hydroxy-6-methoxy-6-oxohexa-2,4-dienoate	9.945	216.0270	P
446	Vanillactic acid	9.945	212.0685	P
447	2,5-Furandicarboxylic acid	9.946	156.0060	P
448	3,6-Epoxy-5,5',6,6'-tetrahydro-b,b-carotene-3',5,5',6'-tetrol	10.081	618.4312	P
449	Gorgostane skeleton	10.090	412.4050	P
450	Kanzonol L	10.111	488.2197	P
451	Balofloxacin	10.187	389.1756	P
452	Allixin	10.192	226.1206	P
453	Hellebrin	10.192	724.3291	P
454	DU 122290	10.195	362.1652	P
455	Mycinamicin III	10.230	681.4092	P
456	(1*alpha*,3*beta*,20*S*,22*R*,24*S*,25*S*)-Pubescenin	10.290	620.3563	P
457	Oleandrin	10.353	576.3296	P
458	Drotaverine	10.383	397.2254	P
459	Ganoderic acid I	10.422	532.3037	P
460	1,2-Epoxypropane	10.490	58.0418	P
461	(±)-(*Z*)-2-(5-Tetradecenyl)cyclobutanone	10.492	264.2453	P
462	Oleamide	10.492	281.2720	P
463	Perulactone B	10.493	488.2774	P
464	DG(15:0/20:1(11*Z*)/0:0)	10.494	608.5351	P
465	Lucidumol A	10.506	472.3549	P
466	DG(18:1(11*Z*)/22:5(4*Z*,7*Z*,10*Z*,13*Z*,16*Z*)/0:0)	10.554	668.5409	P
467	DG(20:3(5*Z*,8*Z*,11*Z*)/22:6(4*Z*,7*Z*,10*Z*,13*Z*,16*Z*,19*Z*)/0:0)	10.555	690.5227	P
468	PE(P-18:1(11*Z*)/15:0)	10.555	687.5198	P
469	DG(14:0/22:4(7*Z*,10*Z*,13*Z*,16*Z*)/0:0)	10.573	616.5036	P
470	Capsi-amide	10.586	269.2720	P
471	4-Nerolidylcatechol	10.595	314.2247	P
472	Drospirenone	10.685	366.2197	P
473	DGlucosyldihydrosphingosine	10.808	463.3514	P

474	2-Pentadecylfuran	10.833	278.2610	P
475	Enalkiren	10.846	656.4289	P
476	D-myo-Inositol-1,4,5-triphosphate	10.896	419.9626	P
477	Cavipetin D	10.897	418.2717	P
478	Sorbitan palmitate	10.897	402.2985	P
479	Eremopetasinorol	11.011	208.1463	P
480	1-a,24*R*,25-Trihydroxyvitamin D2	11.030	444.3223	P
481	Eletriptan	11.124	382.1706	P
482	27-Nor-5b-cholestane-3a,7a,12a,24,25-pentol	11.127	438.3346	P
483	1-Hydroxy-1-phenyl-3-hexadecanone	11.129	332.2698	P
484	OH-Spheroidene	11.135	586.4770	P
485	Stearidonyl carnitine	11.172	420.3125	P
486	4-(Methylnitrosamino)-1-(3-pyridyl)-1-butanolglucuronide	11.174	385.1467	P
487	(22*E*,24*R*)-Stigmasta-4,22-diene-3,6-dione	11.203	424.3333	P
488	Arginyl-Tryptophan	11.223	360.1912	P
489	Muzanzagenin	11.229	442.2708	P
490	Sagecoumarin	11.238	536.0936	P
491	Suillusin	11.239	370.0676	P
492	(3*beta*,22*E*,24*R*)-3-Hydroxyergosta-5,8,22-trien-7-one	11.268	410.3194	P
493	Hoduloside VII	11.304	930.5153	P
494	Cepagenin	11.305	446.3028	P
495	*Beta*-Elemonic acid	11.372	454.3445	P
496	*N*-Hexadecanoylpyrrolidine	11.386	309.3033	P
497	Coriandrone E	11.486	248.0685	P
498	MG(18:0/0:0/0:0)	11.509	358.3079	P
499	Nafoxidine	11.509	425.2343	P
500	PE(14:1(9*Z*)/15:0)	11.511	647.4505	P
501	DG(20:5(5*Z*,8*Z*,11*Z*,14*Z*,17*Z*)/24:1(15*Z*)/0:0)	11.512	724.6029	P
502	Tridemorph	11.542	297.3034	P

503	Hydrocortisone cypionate	11.548	486.2980	P
504	DG(15:0/20:5(5*Z*,8*Z*,11*Z*,14*Z*,17*Z*)/0:0)	11.777	600.4733	P
505	*N*-Palmitoylsphingosine	11.779	537.5148	P
506	DG(15:0/18:3(6*Z*,9*Z*,12*Z*)/0:0)	11.956	576.4759	P
507	(*S*)-Rutaretin	11.968	262.0843	P
508	Udenafil	11.968	516.2522	P
509	4a-Carboxy-5a-cholesta-8,24-dien-3b-ol	11.973	428.3270	P
510	Physalolactone	11.973	538.2334	P
511	Phytal	12.075	294.2926	P
512	PE(16:1(9*Z*)/22:6(4*Z*,7*Z*,10*Z*,13*Z*,16*Z*,19*Z*))	12.102	761.4982	P
513	PC(14:0/22:5(4*Z*,7*Z*,10*Z*,13*Z*,16*Z*))	12.255	780.5542	P
514	12-Ketodeoxycholic acid	12.260	390.2772	P
515	PC(16:0/18:1(9*Z*))[*S*]	12.270	760.5854	P
516	Testosterone isocaproate	12.280	386.2818	P
517	Dioctyl hexanedioate	12.282	370.3085	P
518	Strobilurin A	12.361	258.1254	P
519	L-Phosphatidic acid	12.420	596.3715	P
520	Hyperforin	12.460	536.3844	P
521	Lasonolide A	12.604	696.4240	P
522	Parishin C	12.624	728.2136	P
523	2-Aminoethylphosphocholate	12.939	515.3037	P
524	Glycerol 1-(9Zoctadecenoate) 2-tetradecanoate 3-phosphate	13.119	646.4581	P
525	DG(18:4(6*Z*,9*Z*,12*Z*,15*Z*)/22:6(4*Z*,7*Z*,10*Z*,13*Z*,16*Z*,19*Z*)/0:0)	13.120	660.4735	P
526	Tiamulin	13.720	493.3219	P
527	Streptothricin F acid	13.723	520.2592	P
528	1,2-Di-(9Z,12Zoctadecadienoyl)-snglycero-3-phosphate	13.777	696.4719	P
529	Spirolide B	13.777	693.4608	P
530	*N*-[(4E,8E)-1,3-dihydroxyoctadeca-4,8-dien-2-yl]hexadecanamide	14.007	535.4966	P
531	11*Z*-tetradecenoyl-CoA	14.009	975.2984	P

532	DG(14:0/24:1(15*Z*)/0:0)	15.807	650.5848	P

[a] – retention time [min]

[b] –compound detection in positive (P) or in negative (N) ionization mode.
