# Supplementary material for: Biological Properties of the Mucus and Eggs of Helix aspersa Müller as a Potential Cosmetic and Pharmaceutical Raw Material: A Preliminary Study
Source: Int J Mol Sci. 2024 Sep 15;25(18):9958. doi: 10.3390/ijms25189958 (PMC11432642; doi:10.3390/ijms25189958)
Supplement: Supplementary file 1 [file ijms-25-09958-s001.zip › Herman Anna - Table S13.pdf]

Table S13. Compounds identified in water-methanol* extract of lyophilized mucus of organic *Helix aspersa* snail using LC-MS.

No	Metabolite	RT[a] [min]	Mass [*m/z*]	Detection mode[b]
1	Dulcitol	0.256	182.0792	N
2	Trifluoroacetic acid	0.258	113.9929	N
3	Inabenfide	0.305	338.0821	N
4	Purine	0.313	120.0438	N
5	2-Benzothiazolesulfonamide	0.316	213.9867	N
6	D-Ribose 1-diphosphate	5.617	293.9901	N
7	Blumenol C glucoside	5.727	372.2146	N
8	Ethiprole	5.805	395.9832	N
9	Zingerone	6.230	194.0944	N
10	Bismuth subsalicylate	6.708	361.9972	N
11	Erythrityl Tetranitrate	6.808	301.9970	N
12	Nordihydrocapsiate	6.832	294.1831	N
13	BILA 2185BS	7.040	618.3252	N
14	(*S,Z*)-Lyratol acetate	7.117	194.1309	N
15	3b-Allotetrahydrocorticosterone	7.117	350.2455	N
16	(3b,6b,8b,12a)-8,12-Epoxy-7(11)-eremophilene-6-angeloyloxy-8,12-dimethoxy-3-ol	7.198	394.2358	N
17	Lauryl hydrogen sulfate	7.281	266.1552	N
18	Losartan	7.315	422.1624	N
19	L-Tyrosine methyl ester	7.343	195.0897	N
20	Arnamiol	7.459	450.1809	N
21	*N*-Undecylbenzenesulfonic acid	7.725	312.1758	N
22	2-Dodecylbenzenesulfonic acid	8.160	326.1914	N
23	Sodium Tetradecyl Sulfate	8.200	294.1863	N
24	Dihomo-γ-linolenoyl-EA	8.222	349.2982	N
25	Dinoterb	8.250	240.0751	N
26	(+)-Prosopinine	8.270	313.2615	N

27	Kukoamine D	8.406	530.3118	N
28	Alcaftadine	8.887	307.1686	N
29	Gemfibrozil	8.957	250.1569	N
30	Furmecyclox	9.279	251.1525	N
31	3-Oxochola-4,6-dien-24-oic acid	10.249	370.2509	N
32	(5b,7a,12a)-2-(3-methoxyphenyl)-2-oxoethyl ester-7,12-dihydroxy-cholan-24-oic acid	10.490	540.3444	N
33	Oleamide	10.490	281.2721	N
34	5-Dodecyldihydro-2(3H)-furanone	10.505	254.2246	N
35	Methyl tetradecanoate	10.505	242.2245	N
36	Piritramide	10.548	430.2738	N
37	DG(18:1(11*Z*)/22:5(4*Z*,7*Z*,10*Z*,13*Z*,16*Z*)/0:0)	10.551	668.5399	N
38	DG(20:3(5*Z*,8*Z*,11*Z*)/22:6(4*Z*,7*Z*,10*Z*,13*Z*,16*Z*,19*Z*)/0:0)	10.551	690.5214	N
39	MG(0:0/16:0/0:0)	10.551	330.2770	N
40	Schidigeragenin B	10.552	428.2942	N
41	Enalkiren	10.842	656.4280	N
42	Oleoylglyceronephosphate	11.169	434.2454	N
43	Butroxydim	11.27	399.2406	N
44	Adlupone	11.367	482.3394	N
45	Drotaverine	11.447	397.2253	N
46	3-Hydroxy-6,8-dimethoxy-7(11)-eremophilen-12,8-olide	11.504	310.1786	N
47	Pubesenolide	11.505	458.3051	N
48	DG(20:5(5*Z*,8*Z*,11*Z*,14*Z*,17*Z*)/24:1(15*Z*)/0:0)	11.506	724.6028	N
49	Callystatin A	11.507	456.3249	N
50	MG(18:0/0:0/0:0)	11.507	358.3081	N
51	(22*E*,24*R*)-Stigmasta-4,22-diene-3,6-dione	11.962	424.3340	N
52	5-Heptadecyl-1,3-benzenediol	11.994	348.3033	N
53	Momordicinin	12.361	438.3501	N
54	(3*beta*,22*E*,24*R*)-3-Hydroxyergosta-5,8,22-trien-7-one	12.502	410.3183	N
55	PI(18:0/22:4(10*Z*,13*Z*,16*Z*,19*Z*))	12.783	914.5898	N

1	1-Deoxy-D-glucitol	0.278	166.0841	P
2	5-Heptyltetrahydro-2- oxo-3-furancarboxylic acid	2.976	228.1365	P
3	3-hydroxy- tetradecanedioic acid	3.019	274.1782	P
5	Istamycin C1	3.144	431.2732	P
6	Sedanonic acid	3.240	210.1255	P
7	*N*-n-Hexanoylglycine methyl ester	3.348	187.1208	P
8	Netilmicin	3.447	475.2992	P
9	Homoarecoline	3.462	169.1103	P
10	Wine lactone	3.713	166.0994	P
11	Solanocapsine	3.803	430.3543	P
12	Monomenthyl succinate	3.839	256.1677	P
13	Geranyl acetoacetate	3.849	238.1570	P
14	Arginyl-Isoleucine	3.862	287.1972	P
15	Isopentenyladenine-9-*N*-glucoside	4.036	363.1914	P
16	Tributyrin	4.092	302.1732	P
17	2-(4-Methylphenyl)-2-propanol	4.200	150.1045	P
18	Jasmine ketolactone	4.253	208.1098	P
19	Phlorin	4.270	288.0845	P
20	Humulinic acid A	4.382	266.1519	P
21	Triethylenemelamine	4.394	204.1129	P
22	Octyl gallate	4.395	282.1467	P
23	4,11,13,15-Tetrahydroridentin B	4.418	268.1675	P
24	Halstoctacosanolide A	4.527	844.5359	P
25	(*E*)-3-decen-1-ol	4.545	156.1515	P
26	20-hydroxy-PGF2a	4.545	370.2355	P
27	Diethofencarb	4.549	267.1472	P
28	Flumetover	4.551	367.1392	P
29	Imiquimod	4.634	240.1364	P
30	1,2,3-Tris(1-ethoxyethoxy)propane	4.667	308.2199	P

31	11-Hydroxy-9-tridecenoic acid	4.671	228.1719	P
32	2-Hexenoylcholine	4.672	200.1650	P
33	C12:1n-7	4.685	198.1620	P
34	*Gamma*-CEHC	4.685	248.1413	P
35	Ruscopine	4.692	306.2044	P
36	19(*R*)-hydroxy-PGE2	4.707	368.2197	P
37	2,3-dihydrobenzofuran	4.730	120.0575	P
38	2-Ethylacrylylcarnitine	4.730	244.1551	P
39	2-Phenylbutyric acid	4.730	164.0839	P
40	(5*R*)-5-Hydroxyhexanoic acid	4.731	132.0786	P
41	1-Phenyl-6,7-dihydroxy-isochroman	4.731	242.0945	P
42	3-Indolecarboxylic acid	4.732	253.1313	P
43	*Beta*-hydroxylauric acid	4.732	216.1727	P
44	Arene oxide	4.758	94.0418	P
45	Alanyl-Isoleucine	4.778	202.1318	P
46	Ethyl 7-epi-12- hydroxyjasmonate glucoside	4.793	416.2046	P
47	Methyl 7-epi-12-hydroxyjasmonate glucoside	4.793	402.1891	P
48	Methyl 3-(2,3-dihydroxy- 3-methylbutyl)-4-hydroxybenzoate	4.802	254.1149	P
49	*N*-Isobutyl-2,4,8,10,12-tetradecapentaenamide	4.802	273.2092	P
50	Pinidine	4.809	139.1362	P
51	Orysastrobin	4.844	391.1858	P
52	2-Aminoadenosine	4.847	282.1079	P
53	Methyl propionate	4.961	88.0523	P
54	4'-Hydroxy-3,4,5-trimethoxystilbene	4.962	286.1209	P
55	Pentosidine	4.965	378.2018	P
56	2-Phenylethyl *beta*-D-glucopyranoside	5.010	284.1263	P
57	1,1,2-Triphenylpropane	5.017	272.1555	P
58	5,7-Megastigmadien-9-ol glucoside	5.039	356.2201	P
59	Cinncassiol A 19-glucoside	5.043	544.2515	P

60	Sterebin E	5.075	338.2457	P
61	(*S*)-3-Octanol glucoside	5.078	292.1884	P
62	Gibberellin A105	5.136	330.1466	P
63	(-)-*trans*-Carveol glucoside	5.137	314.1733	P
64	7,8-Dihydrovomifoliol 9-[rhamnosyl-(1->6)-glucoside]	5.160	534.2678	P
65	Glycerol 1-(5-hydroxydodecanoate)	5.230	290.2094	P
66	Toxin T2 tetrol	5.243	298.1418	P
67	Ganglioside GM3 (d18:0/18:1(11*Z*))	5.258	1180.749 9	P
68	Cyclonormammein	5.273	374.1726	P
69	Elaeokanine C	5.289	211.1573	P
70	Artabsinolide A	5.311	280.1312	P
71	Tanacetol B	5.357	296.1985	P
72	Jasmolone glucoside	5.372	342.1682	P
73	Triethyl citrate	5.408	276.1212	P
74	Valyl-Valine	5.428	216.1477	P
75	4-Butyl-5-ethylthiazole	5.430	169.0926	P
76	AF Toxin II	5.433	324.1577	P
77	Hydrocortisone succinate	5.483	462.2253	P
78	Corchoionol C 9-glucoside	5.485	386.1944	P
79	*O*-Methylsomniferine	5.500	622.2656	P
80	(*E,E,E*)-*N*-(2-Methylpropyl)hexadeca- 2,6,8-trien-10-ynamide	5.504	301.2408	P
81	Taraxacolide 1-*O*-b-D-glucopyranoside	5.542	428.2051	P
82	Convallatoxin	5.642	550.2776	P
83	Terazosin	5.654	387.1891	P
84	Blumenol C glucoside	5.683	372.2149	P
85	13,14-dihydro-15-keto-PGF2α	5.687	354.2410	P
86	(2xi,6xi)-7-Methyl-3- methylene-1,2,6,7-octanetetrol	5.692	204.1329	P
87	Hexanal octane-1,3-diol acetal	5.703	228.2091	P

88	Glaucarubin	5.713	496.2286	P
89	2-Methylundecanal	5.724	184.1828	P
90	Blumenol C *O*-[rhamnosyl-(1->6)-glucoside]	5.736	518.2730	P
91	(5*alpha*,10*alpha*)-3,7(11)-Eudesmadien-2-one	5.765	218.1671	P
92	Avocadienofuran	5.765	246.1985	P
93	2-Furanmethanol	5.766	98.0367	P
94	Volicitin	5.767	422.2767	P
95	Goshonoside F3	5.769	644.3390	P
96	NAc-FnorLRF-amide	5.769	622.3570	P
97	2-Hydroxymyristic Acid	5.828	244.2038	P
98	1-Octen-3-yl glucoside	5.840	290.1728	P
99	Glyceollidin II	5.856	340.1308	P
100	Sanshodiol	5.856	358.1418	P
101	Symphytine	5.856	381.2162	P
102	1-(2,4,6-Trimethoxyphenyl)-1,3- butanedione	5.857	252.0999	P
103	2-methyl-glutaric acid	5.857	146.0579	P
104	Glaucamine	5.857	385.1526	P
105	Glaudine	5.857	399.1683	P
106	Macrozamin	5.857	384.1378	P
107	Tetrahydrocurcumin	5.857	372.1574	P
108	C14:1n-9	5.872	226.1934	P
109	Eriojaposide A	5.876	502.2415	P
110	Canavalioside	5.939	546.2680	P
111	Norerythrostachaldine	5.939	407.2655	P
112	(+/-)-*N,N*-Dimethyl menthyl succinamide	6.011	168.1878	P
113	Capsoside A	6.011	694.3772	P
114	1-Hydroxyepiacorone	6.021	252.1724	P
115	15-Acetoxyscirpene-3,4-diol 4-*O*-a-D-glucopyranoside	6.022	486.2100	P
116	Capsaicin	6.057	305.1989	P

117	20-COOH-Leukotriene B4	6.136	366.2042	P
118	(+/-)-[*R*-(*E*)]-5-Isopropyl-8-methylnona- 6,8-dien-2-one	6.139	194.1672	P
119	2-Hydroxyestrone	6.141	286.1570	P
120	(*Z*)-6-Nonenal	6.145	140.1202	P
121	Penbutolol	6.173	291.2194	P
122	Granisetron	6.195	312.1937	P
123	Pseudoargiopinin III	6.204	373.2098	P
124	4-Hydroxy-3-methoxy-2,10-bisaboladien-9-one	6.209	266.1877	P
125	Lauroyl diethanolamide	6.217	287.2463	P
126	(+)-Prosopinine	6.225	313.2617	P
127	4,5-Dihydroniveusin A	6.243	396.1786	P
128	*alpha*-Butyl-*omega*-hydroxypoly(oxyethylene) poly(oxypropylene)	6.269	248.1989	P
129	Chalciporone	6.280	243.1619	P
130	Gravelliferone	6.302	298.1567	P
131	Plantaricin BN	6.332	484.2303	P
132	1,1-Diethoxy-2-hexene	6.351	172.1464	P
133	Cuscohygrine	6.370	224.1890	P
134	Dihydrocapsaicin	6.381	307.2151	P
135	Metoprolol	6.391	267.1836	P
136	Pterosin O	6.392	232.1464	P
137	8-Acetoxy-4-acoren-3-one	6.428	278.1883	P
138	9-HOTE	6.488	294.2197	P
139	Diphenylcarbazide	6.504	242.1156	P
140	(9*Z*,11*E*,13*E*,15*Z*)-4-Oxo-9,11,13,15-octadecatetraenoic acid	6.508	290.1876	P
141	15-keto-Prostaglandin E2	6.509	350.2088	P
142	5-*O*-β-D-Mycaminosyltylonolide	6.557	597.3487	P
143	Chaksine	6.604	450.2968	P
144	Monoisobutyl phthalic acid	6.661	222.0891	P
145	10-Hydroxy-2,8-decadiene-4,6-diynoic acid	6.662	176.0473	P

146	C16 Sphinganine	6.679	273.2670	P
147	p-Hydroxybenzyldesulphoglucosinolate	6.692	345.0868	P
148	2,4,12-Octadecatrienoic acid isobutylamide	6.704	333.3021	P
149	Glicoisoflavanone	6.710	384.1571	P
150	2-Tetradecanone	6.713	212.2142	P
151	Abscisic alcohol 11-glucoside	6.729	412.2102	P
152	5-(2,3-Dihydroxy-3-methylbutyl)-4-(3,4-epoxy-4-methylpentanoyl)-3,4-dihydroxy-2-isopentanoyl-2-cyclopenten-1-one	6.731	412.2100	P
153	Cerberoside	6.731	858.4225	P
154	1-Isomangostin hydrate	6.732	428.1831	P
155	Ximelagatran	6.754	473.2632	P
156	2-Methoxy-estradiol-17β 3-glucuronide	6.755	478.2181	P
157	Phytosphingosine	6.755	317.2929	P
158	CB3717	6.756	477.1650	P
159	Funtumine	6.756	317.2718	P
160	5-Dodecyldihydro-2(3H)-furanone	6.774	254.2246	P
161	Mycalamide B	6.777	517.2889	P
162	Austalide A	6.778	516.2358	P
163	Trilobolide	6.778	522.2440	P
164	3',4',5'-Trimethoxycinnamyl alcohol acetate	6.780	266.1154	P
165	2,2-Dibutyl-3-(4- methoxyphenyl)-4-methyl-2H-1- benzopyran-7-ol acetate	6.781	422.2442	P
166	Porson	6.781	386.1731	P
167	Cinegalline	6.782	430.2105	P
168	Erysothiopine	6.782	407.1023	P
169	Melleolide	6.782	400.1885	P
170	Styrene	6.784	104.0626	P
171	16-hydroxy hexadecanoic acid	6.785	272.2352	P
172	Canescein	6.799	566.2708	P
173	Ipecoside	6.799	565.2176	P

174	Notoginsenoside B	6.800	1122.581<br>4	P
175	5-Megastigmen-7-yne-3,9-diol 9-glucoside	6.808	370.1989	P
176	2-Pentadecanone	6.853	226.2296	P
177	(*S*)-Nerolidol 3-*O*-[a-L- Rhamnopyranosyl-(1->4)-a-L-rhamnopyranosyl-(1->2)-b-D-glucopyranoside]	6.861	676.3670	P
178	13,14-dihydro-15-keto-PGA2	6.875	334.2144	P
179	3-(5,6,6-Trimethylbicyclo[2.2.1]hept-1-yl)cyclohexanol	6.880	236.2140	P
180	Pumiliotoxin 251D	6.891	251.2251	P
181	1-Methyl-2-nonyl-4(1H)-quinolinone	6.892	285.2088	P
182	Genipin 1-*beta*-gentiobioside	6.896	550.1907	P
183	3-Hydroxy-6,8-dimethoxy-7(11)-eremophilen-12,8-olide	6.902	310.1781	P
184	1-Tridecene	6.913	182.2037	P
185	Zizybeoside II	6.918	594.2163	P
186	Panaquinquecol 1	6.922	292.2040	P
187	Chrycolide	6.941	232.0184	P
188	Coriandrone D	6.961	352.1522	P
189	3'-Hydroxy-HT2 toxin	6.974	440.2044	P
190	Muricatacin	6.988	284.2351	P
191	Nonyl octanoate	6.988	270.2558	P
192	6-Caffeoylsucrose	7.020	504.1482	P
193	Palmitic amide	7.029	255.2565	P
194	BILA 2185BS	7.041	618.3256	P
195	3beta-Hydroxypregn-5-ene	7.050	302.2609	P
196	Cyclotetradecane	7.058	196.2192	P
197	Carbophenothion	7.077	341.9753	P
198	Imidaprilat	7.081	377.1587	P
199	Proansamitocin	7.091	443.2290	P
200	Terbucarb	7.091	277.2038	P
201	Spiroxamine	7.098	297.2668	P

202	6*alpha*,9-Difluoro-11*beta*-hydroxypregn-4-ene-3,20-dione	7.104	366.2020	P
203	2-Methoxyestradiol-3-methylether	7.119	316.2024	P
204	2,2-Dimethyl-3,4-bis(4-methoxyphenyl)-2H-1-benzopyran-7-ol acetate	7.122	430.1785	P
205	Finaconitine	7.124	630.3154	P
206	Purothionin AII	7.125	520.2400	P
207	1-Pentadecene	7.139	210.2347	P
208	Paucin	7.174	468.1997	P
209	Z-Gly-Pro-Leu-Gly-Pro	7.188	573.2790	P
210	6-*O*-Acetylaustroinulin	7.208	364.2607	P
211	Candoxatrilat	7.230	399.2243	P
212	Armillaric acid	7.241	416.1833	P
213	Cincassiol B	7.241	400.2095	P
214	Allopumiliotoxin 267A	7.248	267.2198	P
215	Trimethylolpropane trimethacrylate	7.258	338.1729	P
216	Dodecylguanidine	7.261	227.2363	P
217	Marimastat	7.296	331.2123	P
218	Bleekerine	7.315	409.1758	P
219	Armillaripin	7.330	414.2043	P
220	6-*trans*-LTB4	7.358	336.2300	P
221	Gabapentin	7.369	171.1259	P
222	1,8-Heptadecadiene-4,6-diyne-3,10-diol	7.408	260.1778	P
223	Physagulin C	7.438	542.2511	P
224	Etiocholan-3α-ol-17-one 3-glucuronide	7.452	466.2571	P
225	Cyclocalopin F	7.459	294.1103	P
226	Erythroskyrin	7.459	455.2311	P
227	Picrasin C	7.459	422.2307	P
228	(3'x,5'a,9'x,10'b)-*O*-(3-Hydroxy-6-oxo-7-drimen-11-yl)umbelliferone	7.460	396.1938	P
229	17*beta*-Hydroxy-4-mercaptoandrost-4-en-3-one 4-acetate 17-propionate	7.460	418.2189	P
230	*Alpha*-Methylstyrene	7.460	118.0783	P

231	DHAP(18:0)	7.460	436.2601	P
232	(4-Methylphenyl)acetaldehyde	7.461	134.0732	P
233	Austalide L	7.461	428.2201	P
234	Avocadenofuran	7.553	248.2143	P
235	7,10-Hexadecadienoic acid	7.567	252.2091	P
236	Steviolbioside	7.582	642.3252	P
237	Sphinganine	7.600	301.2982	P
238	2-Hexadecanone	7.641	240.2453	P
239	Biperiden	7.678	311.2257	P
240	Zucchini factor B	7.768	663.4307	P
241	6,10,14-Trimethyl-5,9,13-pentadecatrien-2-one	7.774	262.2299	P
242	2,6-Di-tert-butyl-4-ethylphenol	7.776	234.1984	P
243	p-Hydroxyphenethyl *trans*-ferulate	7.779	314.1155	P
244	Methyl 15-cyanopentadecanoate	7.79	281.2357	P
245	Phlegmarine	7.834	250.2410	P
246	(*E*)-3-(2-Hydroxyphenyl)-2-propenal	7.837	148.0525	P
247	Methadone	7.874	309.2094	P
248	Tricycloekasantal	7.875	178.1358	P
249	Estrane-3α,17α-diol	7.880	278.2247	P
250	Pristanic acid	7.918	298.2872	P
251	Methyloctatropine	7.929	282.2434	P
252	Elaiophylin	7.941	1024.593<br>0	P
253	17-Methylandrosta-2,4-dieno[2,3-d]isoxazol-17*beta*-ol	7.946	327.2200	P
254	(3a,5b)-24-oxo-24-[(2-sulfoethyl)amino]cholan-3-yl-b-D-Glucopyranosiduronic acid	7.951	659.3341	P
255	Hematoporphyrin	7.954	598.2810	P
256	Dodecanamide	7.959	199.1938	P
257	Asparagoside D	7.961	902.4880	P
258	Santalyl acetate	7.964	262.1935	P

259	Firocoxib	7.999	336.1030	P
260	Scopoloside II	8.002	770.4093	P
261	Leucomycin A9	8.017	743.4094	P
262	MG(0:0/18:1(11*Z*)/0:0)	8.018	356.2927	P
263	Corchorusoside B	8.033	682.3565	P
264	Dihydro-5-(2-octenyl)-2(3H)-furanone	8.116	196.1464	P
265	Ethyl (4*Z*)-4,7-octadienoate	8.119	168.1151	P
266	Undecylprodigiosin	8.172	393.2784	P
267	2,2,7,7-Tetramethyl-1,6-dioxaspirol[4,4]non-3-ene	8.191	182.1308	P
268	Lyngbyatoxin	8.271	437.3045	P
269	6-Oxocineole	8.274	168.1151	P
270	17*beta*-Acetamidoandrost-4-en-3-one	8.275	329.2357	P
271	Pipercitine	8.275	349.3345	P
272	Tributyl phosphate	8.304	266.1649	P
273	1-Phenyl-1,3-dodecanedione	8.315	274.1933	P
274	Palonosetron	8.327	296.1889	P
275	Vicenistatin	8.327	500.3614	P
276	Methyl 2*E*,4*Z*-hexadecadienoate	8.333	266.2247	P
277	Isopentylideneisopentylamine	8.341	155.1676	P
278	Lentiginosine	8.343	157.1101	P
279	Isometheptene	8.360	141.1517	P
280	Methyl 2-octynoate	8.360	154.0994	P
281	4-Vinylcyclohexene	8.361	108.0936	P
282	Kukoamine D	8.403	530.3127	P
283	Triphenyl phosphate	8.410	326.0711	P
284	Clavamycin B	8.411	362.1423	P
285	LysoPC(15:0)	8.435	482.3254	P
286	Methyprylon	8.449	183.1261	P
287	Dicyclomine	8.466	309.2667	P

288	Momilactone B	8.486	330.1831	P
289	12*S*-HEPE	8.510	318.2198	P
290	3L,7D,11D-phytanic acid	8.513	312.3031	P
291	Linoleoyl Ethanolamide	8.522	323.2825	P
292	Mycinamicin VIII	8.522	505.3390	P
293	*N*-(14-Methylhexadecanoyl)pyrrolidine	8.524	323.3193	P
294	Dodemorph	8.536	281.2720	P
295	8,8-Diethoxy-2,6-dimethyl-2-octanol	8.546	246.2192	P
296	(3a,5b,7a,12a)-24-[(carboxymethyl)amino]-1,12-dihydroxy-24-oxocholan-3-yl-b-D-Glucopyranosiduronic acid	8.547	641.3412	P
297	Protoprimulagenin A 3-[rhamnosyl-(1->4)-rhamnosyl-(1->4)-[rhamnosyl-(1->2)]-glucosyl-(1->?)-glucuronide]	8.547	1234.6314	P
298	Oleyl alcohol	8.561	268.2767	P
299	Polysorbate 20	8.618	522.3403	P
300	Isopimara-7,15-dienol	8.680	288.2451	P
301	Tecostanine	8.744	183.1624	P
302	Polysorbate 60	8.768	434.2883	P
303	TG(8:0/8:0/8:0)	8.768	470.3584	P
304	Laserpitin	8.769	450.2611	P
305	Stearoylethanolamide	8.783	327.3139	P
306	Hexyl heptanoate	8.787	638.2369	P
307	8,11,14,17-icosatetraenoic acid; C20:4n-3,6,9,12	8.862	304.2393	P
308	9-Acetoxyfukinanolide	8.864	292.1676	P
309	13-heptadecyn-1-ol	8.882	252.2452	P
310	Santene	8.882	122.1097	P
311	*trans*-9, *trans*-11-octadecadienoic acid; C18:2n-7,9	8.882	280.2403	P
312	β-Caryophyllene Alcohol	8.917	222.1985	P
313	MG(0:0/20:1(11*Z*)/0:0)	8.924	384.3243	P
314	20,21,21-Trifluoro-3-methoxy-19-nor-17*alpha*-pregna-1,3,5(10),20-tetraen-17-ol	8.942	366.1811	P
315	Phytal	8.989	294.2922	P

316	Annoglabasin F	9.019	378.2406	P
317	(*E,E*)-1,6-bis(4-methoxyphenyl)-1,5-hexadiene	9.040	294.1621	P
318	Isoacitretin	9.040	326.1883	P
319	MG(0:0/22:5(4*Z*,7*Z*,10*Z*,13*Z*,16*Z*)/0:0)	9.053	404.2928	P
320	*Alpha*-CEHC	9.111	278.1521	P
321	22-Oxo-docosanoate	9.135	354.3135	P
322	Anopterine	9.176	541.3039	P
323	Armillarivin	9.188	384.1938	P
324	(6*beta*,7*alpha*,12*beta*,13*beta*)-7-Hydroxy-11,16-dioxo-8,14-apianadien-22,6-olide	9.191	384.1942	P
325	18-Oxocortisol	9.207	376.1885	P
326	PC(22:6(4*Z*,7*Z*,10*Z*,13*Z*,16*Z*,19*Z*)/22:6(4*Z*,7*Z*,10*Z*,13*Z*,16*Z*,19*Z*))	9.207	878.5716	P
327	Misoprostol	9.209	382.2705	P
328	1-(3-Hydroxy-4-methoxyphenyl)-1,2-ethanediol	9.210	184.0737	P
329	Tsangane L 3-glucoside	9.212	374.2305	P
330	DG(14:0/16:1(9*Z*)/0:0)	9.225	538.4577	P
331	Neogrifolin	9.255	328.2405	P
332	Gentamicin	9.265	477.3172	P
333	Deacetylnomilin	9.273	472.2075	P
334	10-Eicosene	9.329	280.3130	P
335	Pravastatin	9.363	424.2456	P
336	Bioresmethrin	9.368	338.1883	P
337	Trimethaphan	9.369	365.1705	P
338	Chloropyramine	9.370	289.1354	P
339	MG(0:0/16:0/0:0)	9.374	330.2769	P
340	Acidissiminol epoxide	9.391	409.2254	P
341	2-Isopropyl-1,4-benzenediol	9.406	152.0836	P
342	MG(0:0/22:6(4*Z*,7*Z*,10*Z*,13*Z*,16*Z*,19*Z*)/0:0)	9.426	402.2756	P
343	(3b,6b,8b,12a)-8,12-Epoxy-7(11)-eremophilene-6-angeloyloxy-8,12-dimethoxy-3-ol	9.428	394.2356	P
344	Methandriol dipropionate	9.430	416.2912	P

345	6-Hydroxy-8-docosanone	9.432	340.3342	P
346	Phenkapton	9.434	375.9367	P
347	Calendulaglycoside E	9.435	794.4296	P
348	Lilac alcohol	9.435	170.1311	P
349	Tsugarioside B	9.445	616.4333	P
350	Iriomoteolide 1a	9.449	506.3222	P
351	Lucidenic acid M	9.455	462.2959	P
352	MG(0:0/18:3(6*Z*,9*Z*,12*Z*)/0:0)	9.461	352.2614	P
353	5-*O*-Desmethyldonepezil	9.492	365.1991	P
354	Erinacine E	9.496	432.2511	P
355	Erythrophleguine	9.497	449.2774	P
356	Galbanic acid	9.497	398.2080	P
357	5,10-Pentadecadien-1-ol	9.511	224.2140	P
358	6*alpha*-Fluoropregn-4-ene-3,20-dione	9.54	332.2140	P
359	Polidocanol	9.554	582.4344	P
360	Heliosupine	9.570	397.2119	P
361	b-D-Glucopyranosiduronic acid, (3a,5b)-24- [(carboxymethyl)amino]- 24-oxocholan-3-yl	9.589	609.3513	P
362	17-*O*-Acetylnorajmaline	9.597	354.1961	P
363	2-(4-Chloro-3,5-dimethylphenoxy)-*N*-(2- phenyl-2H-benzotriazol-5-yl)-acetamide	9.644	406.1194	P
364	4*beta*-(2-Aminoethylthio)catechin	9.644	365.0924	P
365	Monocrotaline	9.644	325.1532	P
366	Persicaxanthin	9.648	384.2661	P
367	HDOPA	9.679	376.2251	P
368	Palmitoyl glucuronide	9.735	418.2931	P
369	Ethyl abietate	9.746	330.2558	P
370	Arachidonyl Trifluoromethyl Ketone	9.769	356.2314	P
371	Oleoyl Ethanolamide	9.789	325.2981	P
372	10,16-dihydroxy-palmitic acid	9.795	288.2296	P
373	Palmitoyl-EA	9.795	299.2824	P

374	*N*-Methylpelletierine	9.813	155.1310	P
375	Labienoxime	9.815	209.1779	P
376	Nitramine	9.816	169.1469	P
377	Tropine	9.817	141.1153	P
378	MG(0:0/22:1(13Z)/0:0)	9.838	412.3554	P
379	(3*S*,6*E*,10*E*)-1,6,10,14-Phytatetraen-3-ol	9.869	290.2612	P
380	1b,3a,7a,12a-Tetrahydroxy-5b-cholanoic acid	9.942	424.2813	P
381	4-Carboxy-2-hydroxy-6-methoxy-6-oxohexa-2,4-dienoate	9.942	216.0271	P
382	Asteltoxin	9.942	418.1994	P
383	Acetyl tributyl citrate	9.943	402.2253	P
384	Kamahine C	9.943	268.1314	P
385	2,5-Furandicarboxylic acid	9.944	156.0059	P
386	Ampalex	9.944	241.1204	P
387	Arbutin	9.944	272.0898	P
388	Cymorcin monoglucoside	9.944	328.1525	P
389	Vanillactic acid	9.945	212.0688	P
390	(*Z*)-9-Cycloheptadecen-1-one	9.983	250.2298	P
391	Gorgostane skeleton	10.090	412.4054	P
392	Luffariellolide	10.094	386.2820	P
393	Kanzonol L	10.110	488.2197	P
394	Acrovestone	10.146	554.2882	P
395	Hellebrin	10.191	724.3291	P
396	DU 122290	10.193	362.1653	P
397	Allixin	10.194	226.1207	P
398	*N*-Desmethyltamoxifen	10.202	357.2095	P
399	Physagulin A	10.217	510.2625	P
400	KOdiA-PC	10.233	664.3828	P
401	(1*alpha*,3*beta*,20*S*,22*R*,24*S*,25*S*)-Pubescenin	10.291	620.3560	P
402	[6]-Gingerdiol 3,5-diacetate	10.337	380.2201	P

403	Drotaverine	10.381	397.2251	P
404	(±)-(*Z*)-2-(5-Tetradecenyl)cyclobutanone	10.387	264.2455	P
405	Ganoderic acid I	10.420	532.3039	P
406	1,2-Epoxypropane	10.492	58.0417	P
407	Oleamide	10.493	281.2720	P
408	Perulactone B	10.494	488.2776	P
409	Lucidumol A	10.505	472.3553	P
410	DG(18:1(11*Z*)/22:5(4*Z*,7*Z*,10*Z*,13*Z*,16*Z*)/0:0)	10.555	668.5401	P
411	DG(20:3(5*Z*,8*Z*,11*Z*)/22:6(4*Z*,7*Z*,10*Z*,13*Z*,16*Z*,19*Z*)/0:0)	10.555	690.5221	P
412	DG(14:0/22:4(7*Z*,10*Z*,13*Z*,16*Z*)/0:0)	10.572	616.5042	P
413	Capsi-amide	10.588	269.2721	P
414	4-(3-Methyl-1-butenyl)-3,3',4',5-tetrahydroxystilbene	10.648	312.1365	P
415	Drospirenone	10.683	366.2197	P
416	*Delta*3,5-Deoxytigogenin	10.778	396.3023	P
417	D-Glucosyldihydrosphingosine	10.811	463.3509	P
418	2-Pentadecylfuran	10.834	278.2611	P
419	Sorbitan palmitate	10.834	402.2985	P
420	Enalkiren	10.845	656.4294	P
421	Cavipetin D	10.898	418.2720	P
422	Eremopetasinorol	11.011	208.1463	P
423	Cinitapride	11.120	402.2259	P
424	Oleoyl dopamine	11.187	417.3223	P
425	*N*-Hexadecanoylpyrrolidine	11.191	309.3034	P
426	3-Epidemissidine	11.210	399.3496	P
427	Stearamide	11.219	283.2875	P
428	Withanolide B	11.351	454.2699	P
429	*Beta*-Elemonic acid	11.371	454.3447	P
430	2,5-Dihydro-4,5-dimethyl-2-(2-methylpropyl)thiazole	11.393	171.1082	P
431	5-Methyldihydrofolic acid	11.395	457.1704	P

432	Phenethyl decanoate	11.481	276.2090	P
433	Bis(3-azidopyridinium)-1,10-decane perchlorate	11.482	380.2433	P
434	Coriandrone E	11.490	248.0685	P
435	MG(18:0/0:0/0:0)	11.509	358.3084	P
436	DG(20:5(5*Z*,8*Z*,11*Z*,14*Z*,17*Z*)/24:1(15*Z*)/0:0)	11.511	724.6031	P
437	Nafoxidine	11.512	425.2347	P
438	Tridemorph	11.540	297.3034	P
439	Hydrocortisone cypionate	11.547	486.2984	P
440	Ganoderic acid *beta*	11.820	500.3141	P
441	Proacaciberin	11.840	391.1482	P
442	Cepagenin	11.957	446.3031	P
443	Ginsenoyne A linoleate	12.063	520.3908	P
444	PC(14:0/22:5(4*Z*,7*Z*,10*Z*,13*Z*,16*Z*))	12.256	780.5530	P
445	12-Ketodeoxycholic acid	12.259	390.2773	P
446	PC(16:0/18:1(9*Z*))[*S*]	12.270	760.5858	P
447	Dioctyl hexanedioate	12.281	370.3085	P
448	Testosterone isocaproate	12.282	386.2814	P
449	Spinosin C	12.290	754.2101	P
450	Strobilurin A	12.357	258.1255	P
451	L-Phosphatidic acid	12.421	596.3716	P
452	Deterrol stearate	12.451	478.3807	P
453	Lasonolide A	12.603	696.4234	P
454	Parishin C	12.624	728.2145	P
455	PC(15:0/20:5(5*Z*,8*Z*,11*Z*,14*Z*,17*Z*))	12.707	766.5390	P
456	Hexadecyl ferulate	12.806	418.3083	P
457	2-Hexaprenyl-6-methoxyphenol	12.954	532.4286	P
458	Cer(d18:0/12:0)	13.026	483.4657	P
459	DG(18:4(6*Z*,9*Z*,12*Z*,15*Z*)/22:6(4*Z*,7*Z*,10*Z*,13*Z*,16*Z*,19*Z*)/0:0)	13.118	660.4737	P
460	Glycerol 1-(9*Z*-octadecenoate) 2-tetradecanoate 3-phosphate	13.119	646.4587	P

461	Didodecyl thiobispropanoate	13.174	514.4053	P
462	LysoPC(18:4(6*Z*,9*Z*,12*Z*,15*Z*))	13.722	516.3112	P
463	Spirolide B	13.774	693.4604	P
464	(3*beta*,5*alpha*,6*beta*,7*alpha*,22*E*,24*R*)-Ergosta-8,22-diene-3,5,6,7-tetrol	13.797	446.3392	P
465	DG(14:0/22:1(13*Z*)/0:0)	14.126	622.5535	P
466	DG(14:1(9*Z*)/24:1(15*Z*)/0:0)	14.296	648.5691	P
467	Tridodecylamine	15.639	521.5901	P
468	DG(14:0/24:1(15*Z*)/0:0)	15.808	650.5852	P

*-methanol: water (1:1, *v/v*)

[a] – retention time [min]

[b] –compound detection in positive (P) or in negative (N) ionization mode.
