# Supplementary material for: Biological Properties of the Mucus and Eggs of Helix aspersa Müller as a Potential Cosmetic and Pharmaceutical Raw Material: A Preliminary Study
Source: Int J Mol Sci. 2024 Sep 15;25(18):9958. doi: 10.3390/ijms25189958 (PMC11432642; doi:10.3390/ijms25189958)
Supplement: Supplementary file 1 [file ijms-25-09958-s001.zip › Herman Anna - Table S14.pdf]

Table S14. Compounds identified in acetonitrile extract of lyophilized mucus of organic *Helix aspersa* snail using LC-MS.

No	Metabolite	RT[a] [min]	Mass [*m/z*]	Detection mode[b]
1	Chlorobenside	0.259	267.9879	N
2	D-erythro-D-galactooctitol	0.265	242.1001	N
3	L-Rhamnulose	0.267	164.0686	N
4	Propargyl alcohol	0.267	56.0263	N
5	Dimethyl carbonate	0.268	90.0318	N
6	Dulcitol	0.268	182.0792	N
7	Glycolaldehyde	0.268	60.0212	N
8	Acrylic acid	0.269	72.0212	N
9	Maltitol	0.269	344.1319	N
10	Oxolan-3-one	0.270	86.0368	N
11	3-b-Galactopyranosyl glucose	0.273	342.1162	N
12	Todatriol glucoside	0.273	390.1535	N
13	L-Xylonate	0.280	166.0480	N
14	Buturon	0.285	236.0707	N
15	Perseitol	0.288	212.0899	N
16	Alanyl-Histidine	0.306	226.1056	N
17	2-[(5-Methylsulfinyl)-4-penten-2-ynylidene]-1,6-dioxaspiro[4.4]non-3-ene	0.318	250.0666	N
18	*N*-Valerylglycine methyl ester	2.404	173.1054	N
19	*N*-n-Hexanoylglycine methyl ester	3.469	187.1211	N
20	L-gamma-glutamyl-Lisoleucine	3.620	260.1371	N
21	*N*-(3-oxo-octanoyl)-homoserine lactone	3.982	241.1315	N
22	Capryloylglycine	4.098	201.1366	N
23	Methyl *N*-(amethylbutyryl)glycine	4.303	188.1049	N
24	*N*-heptanoyl-homoserine lactone	4.454	213.1366	N
25	D-Ribose 1-diphosphate	5.618	293.9904	N
26	Blumenol C glucoside	5.731	372.2149	N

27	Ethiprole	5.806	395.9831	N
28	Zingerone	6.231	194.0945	N
29	Flupropanate	6.561	145.9994	N
30	Bismuth subsalicylate	6.708	361.9978	N
31	Nordihydrocapsiate	6.832	294.1832	N
32	3-Hydroxy-6,8-dimethoxy-7(11)-eremophilen-12,8-olide	7.035	310.1784	N
33	BILA 2185BS	7.040	618.3251	N
34	(*S,Z*)-Lyratol acetate	7.116	194.1309	N
35	3b-Allotetrahydrocorticosterone	7.121	350.2457	N
36	Lauryl hydrogen sulfate	7.280	266.1554	N
37	Losartan	7.314	422.1627	N
38	Methotrexate	7.314	454.1730	N
39	L-Tyrosine methyl ester	7.344	195.0897	N
40	*N*-Undecylbenzenesulfonic acid	7.722	312.1758	N
41	2-Dodecylbenzenesulfonic acid	8.156	326.1912	N
42	Sodium Tetradecyl Sulfate	8.201	294.1862	N
43	Dinoterb	8.251	240.0748	N
44	(+)-Prosopinine	8.273	313.2617	N
45	Docusate	8.338	422.2335	N
46	Kukoamine D	8.406	530.3120	N
47	Alcaftadine	8.889	307.1686	N
48	Gemfibrozil	8.957	250.1572	N
49	Furmecyclox	9.281	251.1523	N
50	3-Oxochola-4,6-dien-24-oic acid	10.247	370.2508	N
51	(5b,7a,12a)-2-(3-methoxyphenyl)-2-oxoethyl ester-7,12-dihydroxy-cholan-24-oic acid	10.296	540.3443	N
52	Enalkiren	10.846	656.4295	N
53	3-Hydroxy-2-(4-morpholinylmethyl)estra-1,3,5(10)-trien-17-one	10.947	369.2305	N
54	Oleoylglycerone phosphate	11.170	434.2454	N
55	Butroxydim	11.272	399.2408	N

56	Adlupone	11.366	482.3394	N
57	(3*beta*,22*E*,24*R*)-3-Hydroxyergosta-5,8,22-trien-7-one	12.506	410.3183	N
1	L-Homocysteic acid	0.238	183.0209	P
2	1,2-Epoxypropane	0.249	58.0419	P
3	Temocaprilat	0.254	448.1109	P
4	Isoamyl nitrite	0.257	117.0791	P
5	Glucoerucin	0.259	421.0553	P
6	6-Hydroxymusizin 8-*O*-b-D-glucopyranoside	0.261	394.1255	P
7	Choline chloride	0.264	103.0997	P
8	3-Hydroxy-3-methyl-2-oxo-pentanoic acid	0.270	146.0581	P
9	3L,7D,11D-phytanic acid	0.270	312.3029	P
10	Osmundalactone	0.270	128.0474	P
11	2-Acetylfuran	0.271	110.0368	P
12	3-deoxyfructose	0.271	164.0685	P
13	2-Furanmethanol	0.274	98.0365	P
14	3-b-Galactopyranosyl glucose	0.274	342.1161	P
15	Dulcitol	0.274	182.0791	P
16	Isradipine	0.276	371.1476	P
17	3-(4-Hydroxybenzoyl)epicatechin	0.279	410.0991	P
18	3-Methylpentyl glucosinolate	0.281	403.0959	P
19	Coriandrin	0.284	230.0576	P
20	4-Guanidinobutanoic acid	0.285	145.0853	P
21	(4-Hydroxybenzoyl)choline	0.287	224.1286	P
22	Nicotinamide *N*-oxide	0.292	138.0430	P
23	(2*R**,3*R**)-1,2,3-Butanetriol	0.367	106.0629	P
24	4,5-Dihydroxyhexanoic acid lactone	0.393	130.0629	P
25	2-Amino-2-methyl-1,3-propanediol	0.397	105.0789	P
26	Trolamine	0.399	149.1052	P
27	*R*-2-Hydroxy-3-methylbutanoic acid 3-Methylbutanoyl	0.658	202.1206	P

28	Scopoline	0.805	155.0947	P
29	Dexpanthenol	0.860	205.1315	P
30	Mequinol	0.924	124.0525	P
31	Turicine	0.924	160.0976	P
32	1-nitroheptane	1.001	145.1105	P
33	2,5-Dihydro-2,4,5-trimethyloxazole	1.508	113.0841	P
34	Pseudoecgonine	1.573	185.1052	P
35	3-[(3-Methylbutyl)nitrosoamino]-2-butanone	2.026	186.1369	P
36	Homoarecoline	2.026	169.1104	P
37	Amyl 2-furoate	2.359	182.0943	P
38	2*E*-Decenedioic acid	2.361	200.1051	P
39	3-Acetyl-2,5-dimethylfuran	2.406	138.0683	P
40	Alanyl-Lysine	2.406	217.1429	P
41	*N*-Valerylglycine methyl ester	2.407	173.1054	P
42	Octylamine	2.570	129.1518	P
43	2,3-Dimethyl-2-cyclohexen-1-one	2.585	124.0887	P
44	DL-2-amino-octanoic acid	2.585	159.1260	P
45	8-Acetoxy-4-acoren-3-one	2.830	278.1881	P
46	5-Heptyltetrahydro-2-oxo-3-furancarboxylic acid	2.965	228.1361	P
47	Propionyl-L-carnitine	3.062	218.1393	P
48	Tussilagine	3.087	199.1211	P
49	LY201116	3.119	240.1264	P
50	Pyracarbolid	3.153	217.1105	P
51	Platydesminium	3.154	274.1436	P
52	96 DL-2-amino-octanoic acid	3.218	159.1260	P
53	97 *N*-Valerylglycine methyl ester	3.219	173.1052	P
54	Tranexamic acid	3.219	157.1102	P
55	Sedanonic acid	3.239	210.1256	P
56	Hexamethylene bisacetamide	3.298	200.1523	P

57	(4-Methylphenyl)acetaldehyde	3.469	134.0732	P
58	1,4-Ipomeadiol	3.470	170.0945	P
59	118 2,3-Dimethyl-2-cyclohexen-1-one	3.470	124.0891	P
60	2-Isopropyl-1,4-benzenediol	3.470	152.0840	P
61	o-Xylene	3.470	106.0783	P
62	γ-Aminobutyryl-lysine	3.470	231.1585	P
63	119 5-(2-Furanyl)-1,2,3,4,5,6-hexahydro-7H-cyclopenta[b]pyridin-7-one	3.471	203.0950	P
64	Prolyl-Valine	3.476	214.1319	P
65	Istamycin C1	3.585	431.2732	P
66	(*R*)-3-Hydroxy-5-phenylpentanoic acid	3.617	194.0949	P
67	Mescaline	3.617	211.1199	P
68	Methoxamine	3.618	211.1211	P
69	Platydesmine	3.618	259.1212	P
70	Slaframine	3.621	198.1369	P
71	Pirbuterol	3.622	240.1473	P
72	2,3-Dihydro-5-(5-methyl-2-furanyl)-1H-pyrrolizine	3.643	187.1000	P
73	1,2,3,4,5,6-Hexahydro-5-methyl-7H-cyclopenta[b]pyridin-7-one	3.656	151.0998	P
74	Netilmicin	3.736	475.2996	P
75	Solanocapsine	3.783	430.3545	P
76	2,6-Dimethoxy-4-propylphenol	3.840	196.1100	P
77	*N*-heptanoyl-homoserine lactone	3.841	213.1368	P
78	Neotussilagine	3.892	199.1209	P
79	Pseudopelletierine	3.894	153.1154	P
80	*N*-(3-oxo-octanoyl)-homoserine lactone	3.985	241.1313	P
81	Methyprylon	4.025	183.1260	P
82	Alanyl-Valine	4.028	188.1160	P
83	Acetyltropine	4.100	183.1262	P
84	Valyl-Lysine	4.101	245.1745	P
85	n-decanohydroxamic acid	4.183	187.1573	P

86	5-Fluoro-5'-Deoxyuridine	4.257	246.0656	P
87	Jasmine ketolactone	4.258	208.1101	P
88	N-Methylmescaline	4.258	225.1368	P
89	Talbutal	4.259	252.1480	P
90	2,2,7,7-Tetramethyl-1,6-dioxaspiro[4.4]nona-3,8-diene	4.298	180.1151	P
91	Mukaadial	4.386	266.1524	P
92	Triethylenemelamine	4.402	204.1126	P
93	5-Phenylvaleric acid	4.456	178.0994	P
94	4-Butyl-2-ethyl-5-methyloxazole	4.457	167.1310	P
95	1-Octen-3-yl glucoside	4.499	290.1730	P
96	Halstoctacosanolide A	4.525	844.5365	P
97	(*E*)-3-decen-1-ol	4.549	156.1515	P
98	Diethofencarb	4.553	267.1470	P
99	Flumetover	4.554	367.1396	P
100	Ethyl 3-(*N*butylacetamido)propionate	4.651	215.1521	P
101	Isoleucyl-Lysine	4.652	259.1895	P
102	1,2,3-Tris(1-ethoxyethoxy)propane	4.673	308.2200	P
103	2-Hexenoylcholine	4.674	200.1649	P
104	C12:1n-7	4.687	198.1619	P
105	Humulinic acid A	4.688	266.1519	P
106	*Gamma*-CEHC	4.689	248.1409	P
107	11-Hydroxy-9-tridecenoic acid	4.693	228.1726	P
108	Ruscopine	4.704	306.2044	P
109	Dyclonine	4.725	289.2045	P
110	2-Phenylbutyric acid	4.731	164.0839	P
111	2-Ethylacrylylcarnitine	4.733	244.1551	P
112	Alanyl-Isoleucine	4.781	202.1320	P
113	*N*-Isobutyl-2,4,8,10,12-tetradecapentaenamide	4.804	273.2095	P
114	Methyl 3-(2,3-dihydroxy-3-methylbutyl)-4-hydroxybenzoate	4.832	254.1155	P

115	Elaeokanine C	4.888	211.1574	P
116	Ganglioside GM3 (d18:1/16:0)	4.946	1152.718<br>2	P
117	2,3-dihydrobenzofuran	4.963	120.0577	P
118	1,3-Diphenyltetramethyldisiloxane	4.964	286.1211	P
119	2-Phenylethyl beta-D-glucopyranoside	5.013	284.1260	P
120	1,1,2-Triphenylpropane	5.014	272.1558	P
121	5,7-Megastigmadien-9-ol glucoside	5.040	356.2196	P
122	Sterebin E	5.078	338.2456	P
123	(*S*)-3-Octanol glucoside	5.099	292.1886	P
124	Arachidonyl Trifluoromethyl Ketone	5.101	356.2316	P
125	Decylubiquinol	5.101	324.2302	P
126	7,8-Dihydrovomifoliol 9-[rhamnosyl-(1->6)-glucoside]	5.133	534.2675	P
127	Gibberellin A105	5.136	330.1464	P
128	(-)-*trans*-Carveol glucoside	5.137	314.1732	P
129	(*S,Z*)-Lyratol acetate	5.162	194.1307	P
130	Toxin T2 tetrol	5.244	298.1418	P
131	Ganglioside GM3 (d18:0/18:1(11*Z*))	5.255	1180.750<br>3	P
132	Cyclonormammein	5.274	374.1728	P
133	(-)-Hygroline	5.327	143.1310	P
134	sn-glycero-3-Phosphocholine	5.348	258.1095	P
135	Jasmolone glucoside	5.372	342.1679	P
136	Allopumiliotoxin 267A	5.402	267.2203	P
137	Triethyl citrate	5.408	276.1212	P
138	Ethyl 7-epi-12-hydroxyjasmonate glucoside	5.409	416.2052	P
139	Hydrocortisone succinate	5.483	462.2252	P
140	Corchoionol C 9-glucoside	5.484	386.1943	P
141	Discadenine	5.495	304.1649	P
142	Isopulegone caffeate	5.539	316.1674	P

143	Satratoxin H	5.567	528.2340	P
144	Dimethylbenzyl carbinyl hexanoate	5.653	248.1777	P
145	(9*Z*,11*R*,12*S*,13*S*,15*Z*)-12,13-Epoxy-11-hydroxy- 9,15-octadecadienoic acid	5.656	310.2152	P
146	Eremopetasinorol	5.658	208.1464	P
147	(2xi,6xi)-7-Methyl-3-methylene-1,2,6,7-octanetetrol	5.701	204.1360	P
148	Hexanal octane-1,3-diol acetal	5.705	228.2092	P
149	2-Methylundecanal	5.725	184.1829	P
150	Blumenol C O-[rhamnosyl-(1->6)-glucoside]	5.738	518.2728	P
151	(5alpha,10alpha)-3,7(11)-Eudesmadien-2-one	5.767	218.1672	P
152	Avocadienofuran	5.768	246.1985	P
153	NAc-FnorLRF-amide	5.772	622.3570	P
154	Fluspirilene	5.808	475.2418	P
155	2-Hydroxymyristic Acid	5.833	244.2042	P
156	Glaudine	5.860	399.1684	P
157	Sanshodiol	5.860	358.1416	P
158	1-(2,4,6-Trimethoxyphenyl)-1,3-butanedione	5.861	252.0999	P
159	Canavalioside	5.941	546.2675	P
160	(+/-)-*N,N*-Dimethyl menthyl succinamide	6.017	168.1879	P
161	Homodihydrojasmone	6.070	180.1515	P
162	Lauroyl diethanolamide	6.071	287.2461	P
163	16b-Hydroxyestrone	6.078	286.1569	P
164	2-Hydroxyestrone	6.146	286.1570	P
165	(*Z*)-6-Nonenal	6.150	140.1202	P
166	Penbutolol	6.179	291.2201	P
167	Marimastat	6.182	331.2123	P
168	Hydroxy-*alpha*-sanshool	6.205	263.1879	P
169	10,11-Epoxy-3,7,11-trimethyl-2*E*,6*E*-tridecadienoic acid	6.211	266.1883	P
170	(+)-Prosopinine	6.227	313.2617	P
171	4,4-Difluoropregn-5-ene-3,20-dione	6.280	350.2065	P

172	Aminoparathion	6.289	261.0600	P
173	Gravelliferone	6.305	298.1569	P
174	*alpha*-Butyl-*omega*-hydroxypoly(oxyethylene) poly(oxypropylene)	6.355	248.1988	P
175	Cuscohygrine	6.373	224.1890	P
176	(10*beta*H,11xi)-11-Hydroxy-13-nor-6-eremophilen-8-one	6.418	222.1622	P
177	Ethyl decanoate	6.590	200.1778	P
178	Phenethyl decanoate	6.591	276.2090	P
179	Bambuterol	6.593	367.2119	P
180	Chaksine	6.606	450.2966	P
181	5,8-tetradecadienoic acid	6.607	224.1775	P
182	C16 Sphinganine	6.684	273.2670	P
183	Artabsinolide A	6.689	280.1313	P
184	Momilactone B	6.694	330.1831	P
185	AF Toxin II	6.696	324.1575	P
186	Dihydrocapsaicin	6.702	307.2149	P
187	2,4,12-Octadecatrienoic acid isobutylamide	6.706	333.3016	P
188	2-Tetradecanone	6.715	212.2141	P
189	5-(2,3-Dihydroxy-3-methylbutyl)-4-(3,4-epoxy-4-methylpentanoyl)-3,4-dihydroxy-2-isopentanoyl-2-cyclopenten-1-one	6.734	412.2100	P
190	Eremopetasinorone A	6.756	206.1307	P
191	Phytosphingosine	6.756	317.2932	P
192	Dihomo-γ-linolenoyl-EA	6.757	349.2965	P
193	Ximelagatran	6.757	473.2629	P
194	2-Methoxy-estradiol-17β 3-glucuronide	6.758	478.2180	P
195	Mycalamide B	6.780	517.2891	P
196	Trilobolide	6.781	522.2443	P
197	Cinegalline	6.784	430.2106	P
198	Erysothiopine	6.784	407.1024	P
199	Porson	6.784	386.1732	P

200	2,2-Dibutyl-3-(4-methoxyphenyl)-4-methyl-2H-1-benzopyran-7-ol acetate	6.785	422.2445	P
201	16-hydroxy hexadecanoic acid	6.789	272.2352	P
202	Funtumine	6.847	317.2720	P
203	2-Methyl-1-phenyl-2-propanyl acetate	6.859	192.1150	P
204	9-HOTE	6.860	294.2190	P
205	(*S*)-Nerolidol 3-*O*-[a-L-Rhamnopyranosyl-(1->4)-a-L-rhamnopyranosyl-(1->2)-b-D-glucopyranoside]	6.864	676.3675	P
206	Pumiliotoxin 251D	6.895	251.2250	P
207	1-Methyl-2-nonyl-4(1H)-quinolinone	6.896	285.2091	P
208	Panaquinquecol 1	6.931	292.2039	P
209	Diclomezine	6.947	254.0003	P
210	Contignasterol	6.948	508.3382	P
211	2,3-Dehydrosalvipisone	6.989	310.1569	P
212	Nonyl octanoate	6.990	270.2557	P
213	Muricatacin	6.992	284.2352	P
214	Acetyl Tyrosine Ethyl Ester	7.029	251.1152	P
215	Palmitic amide	7.034	255.2561	P
216	BILA 2185BS	7.044	618.3257	P
217	Cyclotetradecane	7.063	196.2192	P
218	6,10,14-Trimethyl- 5,9,13-pentadecatrien-2-one	7.103	262.2299	P
219	Herculin	7.114	251.2250	P
220	Finaconitine	7.127	630.3156	P
221	17*beta*-Acetamidoandrost-4-en-3-one	7.133	150.1046	P
222	Enalaprilat	7.174	348.1692	P
223	10,16-dihydroxy-palmitic acid	7.193	288.2302	P
224	*cis*-5-Tetradecenoylcarnitine	7.199	370.2967	P
225	Armillaric acid	7.245	416.1834	P
226	Cincassiol B	7.245	400.2102	P
227	Piperolein B	7.246	343.2142	P
228	1-Phenyl-1,3-dodecanedione	7.249	274.1923	P

229	*trans*-9, *trans*-11-octadecadienoic acid; C18:2n-7,9	7.254	280.2401	P
230	*N*-Dealkylatedtolterodine	7.256	283.1942	P
231	Bleekerine	7.320	409.1758	P
232	Lentiginosine	7.330	157.1104	P
233	Ethyl (4*Z*)-4,7-octadienoate	7.358	168.1152	P
234	Gabapentin	7.374	171.1261	P
235	6-*trans*-LTB4	7.376	336.2284	P
236	Estrane-3α,17α-diol	7.382	278.2247	P
237	9-Decenoylcholine	7.387	256.2278	P
238	Avocadenofuran	7.408	248.2143	P
239	Physagulin C	7.441	542.2504	P
240	2,2-Dimethyl-3,4-bis(4-methoxyphenyl)-2H-1-benzopyran-7-ol acetate	7.463	430.1778	P
241	Armillaripin	7.463	414.2042	P
242	Vilazodone	7.463	441.2153	P
243	Erythroskyrin	7.464	455.2307	P
244	3-(5,6,6-Trimethylbicyclo[2.2.1]h ept-1-yl)cyclohexanol	7.478	236.2139	P
245	Methyl (9*Z*)-10'-oxo-6,10'-diapo-6-carotenoate	7.502	312.1726	P
246	Norpropoxyphene	7.515	325.2042	P
247	7,10-Hexadecadienoic acid	7.532	252.2087	P
248	C14:1n-9	7.592	226.1934	P
249	Sphinganine	7.603	301.2982	P
250	2-Hexadecanone	7.644	240.2455	P
251	Spiroxamine	7.665	297.2668	P
252	Biperiden	7.683	311.2244	P
253	1-(4-Amino-2-methylpyrimid-5-ylmethyl)-3-(*beta*-hydroxyethyl)-2-methylpyridinium	7.750	259.1552	P
254	Capryloylglycine	7.774	201.1366	P
255	Zucchini factor B	7.774	663.4307	P
256	2,2,6,6-Tetramethyl-4-piperidinone	7.780	155.1307	P
257	2,6-Di-tert-butyl-4-ethylphenol	7.781	234.1983	P

258	8-Acetoxypinoresinol 4-glucoside	7.783	578.2024	P
259	Methyloctatropine	7.794	282.2435	P
260	(*E*)-3-(2-Hydroxyphenyl)-2-propenal	7.841	148.0524	P
261	Glaucamine	7.841	385.1524	P
262	Methadone	7.876	309.2089	P
263	17-Methylandrosta-2,4-dieno[2,3-d]isoxazol-17beta-ol	7.946	327.2192	P
264	Dodecanamide	7.962	199.1937	P
265	Asparagoside D	7.965	902.4878	P
266	Scopoloside II	8.004	770.4087	P
267	Stearamide	8.016	283.2876	P
268	MG(0:0/18:1(11*Z*)/0:0)	8.017	356.2923	P
269	Leucomycin A9	8.019	743.4090	P
270	Corchorusoside B	8.036	682.3565	P
271	Convallatoxin	8.093	550.2780	P
272	Dihydro-5-(2-octenyl)-2(3H)-furanone	8.119	196.1464	P
273	Mangiferursanone	8.124	426.3851	P
274	Methyl 15-cyanopentadecanoate	8.133	281.2358	P
275	Undecylprodigiosin	8.175	393.2783	P
276	2,2,7,7-Tetramethyl-1,6-dioxaspiro[4.4]non-3-ene	8.193	182.1306	P
277	Lyngbyatoxin	8.274	437.3048	P
278	Erinacine G	8.275	464.2425	P
279	Coniine	8.276	127.1361	P
280	Santene	8.277	122.1097	P
281	2-(4-Methylphenyl)-2-propanol	8.278	150.1045	P
282	Pipercitine	8.278	349.3329	P
283	6-Oxocineole	8.279	168.1151	P
284	p-Mentha-1,3,5,8-tetraene	8.280	532.3858	P
285	Tributyl phosphate	8.308	266.1649	P
286	1-Methyl-1,3-cyclohexadiene	8.346	94.0781	P

287	*cis*-1,2-Dihydro-3-ethylcatechol	8.346	140.0838	P
288	Isopentylideneisopentylamine	8.346	155.1676	P
289	Tropine	8.349	141.1155	P
290	Sorbitan oleate	8.350	428.3117	P
291	Uscharidin	8.351	530.2497	P
292	4-Vinylcyclohexene	8.361	108.0938	P
293	*N*-Methylpelletierine	8.361	155.1308	P
294	Isometheptene	8.363	141.1517	P
295	Homostachydrine	8.369	158.1183	P
296	2-Decylfuran	8.374	208.1829	P
297	Flabellidine	8.388	288.2202	P
298	Methyl 2*E*,4*Z*-hexadecadienoate	8.403	266.2246	P
299	Kukoamine D	8.405	530.3123	P
300	SB 221284	8.411	353.0820	P
301	Triphenyl phosphate	8.412	326.0709	P
302	Clavamycin B	8.414	362.1421	P
303	(*Z*)-9-Cycloheptadecen-1-one	8.419	250.2298	P
304	Panamine	8.490	315.2681	P
305	Coprocholic acid	8.510	450.3345	P
306	*N*-(14-Methylhexadecanoyl)pyrrolidine	8.527	323.3188	P
307	Dodemorph	8.539	281.2720	P
308	8,8-Diethoxy-2,6-dimethyl-2-octanol	8.547	246.2198	P
309	(3a,5b,7a,12a)-24-[(carboxymethyl)amino]-1,12-dihydroxy-24-oxocholan-3-yl-b-D-Glucopyranosiduronic acid	8.548	641.3411	P
310	Oleyl alcohol	8.562	268.2768	P
311	5*beta*-Gonane	8.600	232.2195	P
312	Polysorbate 20	8.618	522.3408	P
313	Stearoylethanolamide	8.689	327.3140	P
314	Polysorbate 60	8.768	434.2881	P
315	Laserpitin	8.769	450.2614	P

316	TG(8:0/8:0/8:0)	8.769	470.3596	P
317	Hexyl heptanoate	8.788	638.2363	P
318	Tecostanine	8.830	183.1623	P
319	9-Acetoxyfukinanolide	8.864	292.1677	P
320	13-heptadecyn-1-ol	8.880	252.2452	P
321	Methyl 2-octynoate	8.882	154.0995	P
322	MG(0:0/20:1(11*Z*)/0:0)	8.926	384.3240	P
323	Tris(butoxyethyl)phosphate	8.929	398.2436	P
324	3-Cyclohexyldodecane	9.012	252.2816	P
325	Isoacitretin	9.039	326.1882	P
326	(*E,E*)-1,6-bis(4-methoxyphenyl)-1,5-hexadiene	9.041	294.1619	P
327	24-Hydroxycalcitriol	9.091	432.3236	P
328	Anofinic acid	9.107	204.0787	P
329	*Alpha*-CEHC	9.111	278.1519	P
330	22-Oxo-docosanoate	9.136	354.3135	P
331	*N*-n-Hexanoylglycine methyl ester	9.136	187.1211	P
332	MG(0:0/22:2(13*Z*,16*Z*)/0:0)	9.167	410.3393	P
333	1-(3-Hydroxy-4-methoxyphenyl)-1,2-ethanediol	9.206	184.0737	P
334	18-Oxocortisol	9.206	376.1885	P
335	Linoleoyl Ethanolamide	9.207	323.2826	P
336	Misoprostol	9.207	382.2705	P
337	Stearidonyl carnitine	9.220	420.3097	P
338	10-Eicosene	9.328	280.3133	P
339	Pravastatin	9.364	424.2464	P
340	Bioresmethrin	9.369	338.1885	P
341	Chloropyramine	9.371	289.1356	P
342	MG(0:0/16:0/0:0)	9.375	330.2770	P
343	Calendulaglycoside E	9.432	794.4250	P
344	MG(0:0/22:6(4*Z*,7*Z*,10*Z*,13*Z*,16*Z*,19*Z*)/0:0)	9.432	402.2754	P

345	(3b,6b,8b,12a)-8,12-Epoxy-7(11)-eremophilene-6-angeloyloxy-8,12-dimethoxy-3-ol	9.433	394.2353	P
346	(3'x,5'a,9'x,10'b)-*O*-(3-Hydroxy-6-oxo-7-drimen-11-yl)umbelliferone	9.433	396.1934	P
347	Lilac alcohol	9.433	170.1309	P
348	Methyl acrylatedivinylbenzene, completely hydrolyzed, copolymer	9.433	398.1157	P
349	Phenkapton	9.433	375.9361	P
350	[6]-Gingerdiol 3,5-diacetate	9.434	380.2202	P
351	Methandriol dipropionate	9.434	416.2909	P
352	MG(0:0/18:3(6*Z*,9*Z*,12*Z*)/0:0)	9.462	352.2614	P
353	Trimethaphan	9.492	365.1703	P
354	5,10-Pentadecadien-1-ol	9.510	224.2142	P
355	Polidocanol	9.553	582.4345	P
356	17-*O*-Acetylnorajmaline	9.595	354.1961	P
357	2-oxophytanic acid	9.617	326.2824	P
358	4beta-(2-Aminoethylthio)catechin	9.642	365.0925	P
359	Monocrotaline	9.643	325.1535	P
360	2-(4-Chloro-3,5-dimethylphenoxy)-*N*-(2-phenyl-2H-benzotriazol-5-yl)-acetamide	9.645	406.1199	P
361	Palmitoyl glucuronide	9.734	418.2932	P
362	Philanthotoxin 343	9.737	435.3198	P
363	Lycopersiconol	9.769	334.2504	P
364	Oleoyl Ethanolamide	9.789	325.2982	P
365	Palmitoyl-EA	9.800	299.2826	P
366	6,8a-Seco-6,8a-deoxy-5-oxoavermectin "2a" aglycone	9.821	586.3508	P
367	Dolicholide	9.828	478.3294	P
368	MG(0:0/22:1(13Z)/0:0)	9.841	412.3553	P
369	4-Carboxy-2-hydroxy-6-methoxy-6-oxohexa-2,4-dienoate	9.942	216.0270	P
370	1b,3a,7a,12a-Tetrahydroxy-5b-cholanoic acid	9.943	424.2808	P
371	2,5-Furandicarboxylic acid	9.943	156.0059	P
372	Acetyl tributyl citrate	9.943	402.2253	P
373	Arbutin	9.943	272.0895	P

374	Asteltoxin	9.943	418.1988	P
375	Cymorcin monoglucoside	9.943	328.1522	P
376	Vanillactic acid	9.943	212.0687	P
377	Kamahine C	9.944	268.1311	P
378	Balofloxacin	10.198	389.1758	P
379	DU 122290	10.201	362.1652	P
380	Tamoxifen	10.201	371.2247	P
381	1-Methylpyrrolinium	10.205	84.0810	P
382	Drotaverine	10.381	397.2256	P
383	Arachidonyl carnitine	10.414	504.4030	P
384	Oleamide	10.492	281.2719	P
385	DG(20:3(5*Z*,8*Z*,11*Z*)/22:6(4*Z*,7*Z*,10*Z*,13*Z*,16*Z*,19*Z*)/0:0)	10.552	690.5225	P
386	DG(18:1(11*Z*)/22:5(4*Z*,7*Z*,10*Z*,13*Z*,16*Z*)/0:0)	10.553	668.5401	P
387	(9*Z*,11*E*,13*E*,15*Z*)-4-Oxo-9,11,13,15-octadecatetraenoic acid	10.563	290.1883	P
388	4-Methoxycinnamic acid	10.563	178.0630	P
389	Capsi-amide	10.578	269.2720	P
390	Drospirenone	10.684	366.2197	P
391	D-Glucosyldihydrosphingosine	10.807	463.3513	P
392	Sorbitan palmitate	10.834	402.2982	P
393	D-myo-Inositol-1,4,5-triphosphate	10.896	419.9633	P
394	Cavipetin D	10.897	418.2720	P
395	Ganodermic acid TQ	11.093	510.3347	P
396	PC(14:0/22:5(4*Z*,7*Z*,10*Z*,13*Z*,16*Z*))	12.258	780.5541	P
397	12-Ketodeoxycholic acid	12.262	390.2771	P
398	PC(16:0/18:1(9*Z*))[*S*]	12.270	760.5853	P
399	Dioctyl hexanedioate	12.284	370.3085	P
400	Testosterone isocaproate	12.286	386.2818	P
401	Hyperforin	13.176	536.3879	P
402	Delphinidin 3-lathyroside 5-glucoside	13.488	759.1958	P

403	5,6-Dihydro-5,6-dihydroxy-y,y-carotene	13.794	572.4583	P
404	Didodecyl thiobispropanoate	13.834	514.4060	P
405	Elastin	13.837	552.3613	P

[a] – retention time [min]

[b] –compound detection in positive (P) or in negative (N) ionization mode.
