# Supplementary material for: Biological Properties of the Mucus and Eggs of Helix aspersa Müller as a Potential Cosmetic and Pharmaceutical Raw Material: A Preliminary Study
Source: Int J Mol Sci. 2024 Sep 15;25(18):9958. doi: 10.3390/ijms25189958 (PMC11432642; doi:10.3390/ijms25189958)
Supplement: Supplementary file 1 [file ijms-25-09958-s001.zip › Herman Anna - Table S15.pdf]

Table S15. Compounds identified in acetonitrile-water[*] extract of lyophilized mucus of organic *Helix aspersa* snail using LC-MS.

No	Metabolite	RT[a] [min]	Mass [*m/z*]	Detection mode[b]
1	Tetrachlorobisphenol A	0.256	363.9599	N
2	Glycolaldehyde	0.268	60.0211	N
3	(*S*)-a-Amino-2,5-dihydro-5-oxo-4-isoxazolepropanoic acid *N*2-glucoside	0.269	334.1008	N
4	Acrylic acid	0.269	72.0211	N
5	Dulcitol	0.269	182.0791	N
6	L-Rhamnulose	0.269	164.0686	N
7	Oxolan-3-one	0.269	86.0368	N
8	Propargyl alcohol	0.269	56.0263	N
9	Spirodiclofen	0.269	410.1034	N
10	Maltitol	0.272	344.1319	N
11	3-b-Galactopyranosyl glucose	0.274	342.1164	N
12	D-Fructose	0.275	180.0635	N
13	L-Xylonate	0.278	166.0478	N
14	Threonate	0.281	136.0373	N
15	Valiolone	0.281	192.0636	N
16	D-Thevetose	0.311	178.0841	N
17	2,3-Dihydroxy-3-methylbutyric acid	0.316	134.0583	N
18	Nicotinamide *N*-oxide	0.316	138.0431	N
19	Trifluoroacetic acid	0.318	113.9929	N
20	Flupropanate	0.321	145.9991	N
21	2-Succinyl-5-enolpyruvyl-6-hydroxy-3-cyclohexene-1-carboxylate	0.322	328.0782	N
22	2-Benzothiazolesulfonamide	0.333	213.9865	N
23	2-[(5-Methylsulfinyl)-4-penten-2-ynylidene]-1,6-dioxaspiro[4.4]non-3-ene	0.563	250.0665	N
24	3-Oxochola-4,6-dien-24-oic acid	10.249	370.2506	N
25	(5b,7a,12a)-2-(3-methoxyphenyl)-2-oxoethyl ester-7,12-dihydroxy-cholan-24-oic acid	10.294	540.3452	N
26	Methyl tetradecanoate	10.498	242.2247	N

27	2(3H)-Furanone	10.503	84.0211	N
28	5-Dodecyldihydro-2(3H)-furanone	10.503	254.2248	N
29	Piritramide	10.546	430.2732	N
30	MG(0:0/16:0/0:0)	10.549	330.2766	N
31	DG(18:1(11*Z*)/22:5(4*Z*,7*Z*,10*Z*,13*Z*,16*Z*)/0:0)	10.551	668.5401	N
32	Schidigeragenin B	10.551	428.2947	N
33	Oleoylglycerone phosphate	11.168	434.2454	N
34	Butroxydim	11.270	399.2408	N
35	Adlupone	11.364	482.3396	N
36	Drotaverine	11.446	397.2252	N
37	DG(20:5(5*Z*,8*Z*,11*Z*,14*Z*,17*Z*)/24:1(15*Z*)/0:0)	11.506	724.6026	N
38	Pubesenolide	11.506	458.3052	N
39	MG(18:0/0:0/0:0)	11.507	358.3084	N
40	(3*beta*,22*E*,24*R*)-3-Hydroxyergosta-5,8,22-trien-7-one	12.503	410.3181	N
41	*N*-n-Hexanoylglycine methyl ester	3.467	187.1212	N
42	D-Ribose 1-diphosphate	5.615	293.9906	N
43	Blumenol C glucoside	5.731	372.2149	N
44	Ethiprole	5.804	395.9833	N
45	Zingerone	6.230	194.0945	N
46	Bismuth subsalicylate	6.708	361.9975	N
47	Eremopetasinorol	6.778	208.1467	N
48	Nordihydrocapsiate	6.833	294.1831	N
49	3-Hydroxy-6,8-dimethoxy-7(11)-eremophilen-12,8-olide	7.035	310.1784	N
50	BILA 2185BS	7.040	618.3253	N
51	(±)-Rollipyrrole	7.049	288.1476	N
52	4-Hydroxy-5-phenyltetrahydro-1,3-oxazin-2-one	7.056	193.0742	N
53	(*S*,*Z*)-Lyratol acetate	7.116	194.1309	N
54	3b-Allotetrahydrocorticosterone	7.120	350.2457	N
55	(3b,6b,8b,12a)-8,12-Epoxy-7(11)-eremophilene-6-angeloyloxy-8,12-dimethoxy-3-ol	7.200	394.2356	N

56	Lauryl hydrogen sulfate	7.280	266.1552	N
57	4'-Methoxymucidin	7.281	288.1370	N
58	Zanthodioline	7.282	305.1265	N
59	Losartan	7.315	422.1622	N
60	Methotrexate	7.315	454.1726	N
61	L-Tyrosine methyl ester	7.345	195.0897	N
62	*N*-Methyl-14-*O*-demethylepiporphyroxine	7.459	371.1367	N
63	*N*-Undecylbenzenesulfonic acid	7.723	312.1758	N
64	2-Dodecylbenzenesulfonic acid	8.157	326.1916	N
65	Sodium Tetradecyl Sulfate	8.201	294.1864	N
66	Dihomo-γ-linolenoyl-EA	8.223	349.2979	N
67	Dimethyl carbonate	8.223	90.0317	N
68	Dinoterb	8.251	240.0747	N
69	(+)-Prosopinine	8.275	313.2618	N
70	Kukoamine D	8.406	530.3113	N
71	Alcaftadine	8.888	307.1686	N
72	Gemfibrozil	8.958	250.1570	N
73	Furmecyclox	9.280	251.1522	N
74	Nisoldipine	9.388	388.1639	N
1	Isoamyl nitrite	0.214	117.0792	P
2	2-Methyl-2-(methyldithio)propanal	0.236	150.0171	P
3	Trichlopyr	0.237	254.9255	P
4	L-Homocysteic acid	0.238	183.0210	P
5	2-[(5-Methylsulfinyl)-4-penten-2-ynylidene]-1,6-dioxaspiro[4.4]non-3-ene	0.251	250.0665	P
6	Temocaprilat	0.255	448.1110	P
7	O-Carbamoyl-deacetylcephalosporin C	0.258	416.0993	P
8	2-Acetylfuran	0.270	110.0366	P
9	3-deoxyfructose	0.270	164.0685	P
10	Osmundalactone	0.270	128.0473	P

11	3-Hydroxy-3-methyl-2-oxo-pentanoic acid	0.271	146.0580	P
12	Furan	0.271	68.0261	P
13	Eflornithine	0.272	182.0869	P
14	3-b-Galactopyranosyl glucose	0.274	342.1162	P
15	Dulcitol	0.275	182.0793	P
16	Isradipine	0.275	371.1475	P
17	Sucrose	0.278	342.1163	P
18	4-Guanidinobutanoic acid	0.300	145.0853	P
19	1-Isothiocyanato-6-(methylthio)hexane	0.310	189.0651	P
20	Dacarbazine	0.310	182.0911	P
21	Kanzonol Z	0.311	406.1776	P
22	8-Hydroxypurine	0.314	138.0548	P
23	4-Guanidino-1-butanol	0.317	131.1062	P
24	2-Amino-2-methyl-1,3-propanediol	0.394	105.0789	P
25	Trolamine	0.399	149.1052	P
26	(2*R*,3*R*,4*R*)-2-Amino-4-hydroxy-3-methylpentanoic acid	0.402	147.0895	P
27	Dexpanthenol	0.873	205.1313	P
28	2,5-Dihydro-2,4,5- trimethyloxazole	1.507	113.0841	P
29	Retronecine	2.433	155.0947	P
30	Propionyl-L-carnitine	2.523	218.1393	P
31	DL-2-amino-octanoic acid	2.591	159.1261	P
32	5-Heptyltetrahydro-2-oxo-3-furancarboxylic acid	2.968	228.1363	P
33	Guaifenesin	3.033	198.0894	P
34	Geranyl acetoacetate	3.042	238.1567	P
35	Sedanonic acid	3.136	210.1258	P
36	Capryloylglycine	3.153	201.1364	P
37	E-64	3.153	357.2001	P
38	Dioscoretine	3.188	241.1679	P
39	2-Isopropyl-1,4-benzenediol	3.230	152.0839	P

40	Wine lactone	3.306	166.0994	P
41	2,3-Dimethyl-2-cyclohexen-1-one	3.469	124.0889	P
42	Homoarecoline	3.469	169.1104	P
43	γ-Aminobutyryl-lysine	3.469	231.1582	P
44	3-hydroxy-tetradecanedioic acid	3.570	274.1784	P
45	Istamycin C1	3.586	431.2732	P
46	Slaframine	3.609	198.1370	P
47	2,3-Dihydro-5-(5-methyl-2-furanyl)-1H-pyrrolizine	3.611	187.0999	P
48	Gabapentin	3.615	171.1259	P
49	4-Vinylcyclohexene	3.617	108.0939	P
50	Humulinic acid A	3.633	266.1518	P
51	Lupinate	3.640	306.1449	P
52	1,2,3,4,5,6-Hexahydro-5-methyl-7H-cyclopenta[b]pyridin-7-one	3.654	151.0998	P
53	Phlorin	3.709	288.0845	P
54	Netilmicin	3.735	475.2996	P
55	*S*-(2-Methylbutanoyl)-dihydrolipoamide	3.809	291.1337	P
56	Monomenthyl succinate	3.850	256.1677	P
57	2-Phenylbutyric acid	3.881	164.0839	P
58	*N*-(3-oxo-octanoyl)-homoserine lactone	3.983	241.1315	P
59	Alanyl-Valine	4.024	188.1160	P
60	Isopentenyladenine-9-N-glucoside	4.038	363.1910	P
61	Tributyrin	4.094	302.1733	P
62	Acetyltropine	4.098	183.1261	P
63	4,4-Difluoro-17beta-hydroxyandrost-5-en-3-one propionate	4.133	380.2180	P
64	Jasmine ketolactone	4.258	208.1098	P
65	*N*-Methylmescaline	4.258	225.1366	P
66	Octyl gallate	4.294	282.1469	P
67	2,2,7,7-Tetramethyl-1,6-dioxaspiro[4.4]nona-3,8-diene	4.297	180.1152	P
68	Avenic acid A	4.301	322.1384	P

69	Pymetrozine	4.333	217.0961	P
70	Mukaadial	4.389	266.1519	P
71	Amyl 2-furoate	4.405	182.0944	P
72	4,11,13,15-Tetrahydroridentin B	4.409	268.1675	P
73	5-Phenylvaleric acid	4.454	178.0996	P
74	Halstoctacosanolide A	4.528	844.5361	P
75	Granisetron	4.533	312.1935	P
76	(*E*)-3-decen-1-ol	4.549	156.1515	P
77	Diethofencarb	4.553	267.1473	P
78	Flumetover	4.553	367.1397	P
79	Ethyl 3-(*N*-butylacetamido)propionate	4.652	215.1522	P
80	1,2,3-Tris(1-ethoxyethoxy)propane	4.673	308.2200	P
81	2-Hexenoylcholine	4.674	200.1652	P
82	1-Methyl-2-propylbenzene	4.678	134.1096	P
83	C12:1n-7	4.688	198.1622	P
84	*Gamma*-CEHC	4.688	248.1414	P
85	8,12-Epoxy-4(15),7,11-eudesmatrien-1-one	4.689	230.1306	P
86	11-Hydroxy-9-tridecenoic acid	4.694	228.1725	P
87	2-Ethylacrylylcarnitine	4.732	244.1551	P
88	(5*R*)-5-Hydroxyhexanoic acid	4.733	132.0784	P
89	2,3-dihydrobenzofuran	4.733	120.0574	P
90	1-Phenyl-6,7-dihydroxy- isochroman	4.734	242.0946	P
91	Arene oxide	4.762	94.0418	P
92	Alanyl-Isoleucine	4.782	202.1320	P
93	Monocrotophos	4.783	223.0612	P
94	Ethyl 7-epi-12-hydroxyjasmonate glucoside	4.791	416.2052	P
95	Methyl 7-epi-12-hydroxyjasmonate glucoside	4.791	402.1891	P
96	N-Isobutyl-2,4,8,10,12-tetradecapentaenamide	4.803	273.2094	P
97	Methyl 3-(2,3-dihydroxy-3-methylbutyl)-4-hydroxybenzoate	4.821	254.1153	P

98	5,7-Megastigmadien-9-ol glucoside	4.971	356.2203	P
99	2-Phenylethyl beta-D-glucopyranoside	5.015	284.1261	P
100	1,1,2-Triphenylpropane	5.022	272.1560	P
101	Triethylenemelamine	5.044	204.1129	P
102	Sterebin E	5.079	338.2456	P
103	Z-Arg-Arg-NHMec	5.080	621.3052	P
104	(*S*)-3-Octanol glucoside	5.103	292.1885	P
105	(-)-*trans*-Carveol glucoside	5.138	314.1733	P
106	Gibberellin A105	5.138	330.1464	P
107	7,8-Dihydrovomifoliol 9-[rhamnosyl-(1->6)-glucoside]	5.163	534.2674	P
108	Imiquimod	5.241	240.1363	P
109	Toxin T2 tetrol	5.245	298.1418	P
110	20-hydroxy-PGF2a	5.251	370.2359	P
111	Ganglioside GM3(d18:0/18:1(11*Z*))	5.258	1180.751 7	P
112	Cyclonormammein	5.274	374.1729	P
113	Elaeokanine C	5.289	211.1574	P
114	Jasmolone glucoside	5.374	342.1680	P
115	Cinitapride	5.387	402.2259	P
116	Triethyl citrate	5.410	276.1212	P
117	Valyl-Valine	5.429	216.1473	P
118	AF Toxin II	5.432	324.1575	P
119	4-Butyl-5-ethylthiazole	5.458	169.0926	P
120	Hydrocortisone succinate	5.483	462.2254	P
121	Corchoionol C 9-glucoside	5.484	386.1941	P
122	(*E,E,E*)-*N*-(2-Methylpropyl)hexadeca- 2,6,8-trien-10-ynamide	5.505	301.2408	P
123	Isopulegone caffeate	5.568	316.1673	P
124	(*S,Z*)-Lyratol acetate	5.638	194.1306	P
125	Terazosin	5.654	387.1889	P

126	Eremopetasinorol	5.656	208.1464	P
127	Eremopetasinorone A	5.685	206.1306	P
128	Blumenol C glucoside	5.689	372.2150	P
129	(2xi,6xi)-7-Methyl-3-methylene-1,2,6,7-octanetetrol	5.701	204.1364	P
130	Hexanal octane-1,3-diol acetal	5.706	228.2092	P
131	2-Methylundecanal	5.727	184.1828	P
132	Blumenol C *O*-[rhamnosyl-(1->6)-glucoside]	5.740	518.2728	P
133	(5*alpha*,10*alpha*)-3,7(11)-Eudesmadien-2-one	5.769	218.1669	P
134	Avocadienofuran	5.769	246.1985	P
135	NAc-FnorLRF-amide	5.773	622.3567	P
136	Diphenoxylic acid(DPA)	5.791	424.2161	P
137	Fluspirilene	5.809	475.2417	P
138	19(*R*)-hydroxy-PGE2	5.821	368.2195	P
139	1-Octen-3-yl glucoside	5.844	290.1728	P
140	Zearalenone 4-sulfate	5.852	398.1038	P
141	1-(2,4,6-Trimethoxyphenyl)-1,3-butanedione	5.861	252.0999	P
142	Glaucamine	5.861	385.1528	P
143	Glaudine	5.861	399.1680	P
144	Glyceollidin II	5.861	340.1310	P
145	Sanshodiol	5.861	358.1418	P
146	C14:1n-9	5.877	226.1933	P
147	Eriojaposide A	5.879	502.2417	P
148	Pterosin O	5.902	232.1464	P
149	Canavalioside	5.943	546.2681	P
150	Capsoside A	6.014	694.3779	P
151	(+/-)-*N,N*-Dimethyl menthyl succinamide	6.015	168.1878	P
152	Sterebin B	6.016	352.2251	P
153	1-Hydroxyepiacorone	6.031	252.1728	P
154	Capsaicin	6.062	305.1988	P

155	Homodihydrojasmone	6.070	180.1515	P
156	Lauroyl diethanolamide	6.073	287.2465	P
157	2-Hydroxyestrone	6.080	286.1570	P
158	(+)-Prosopinine	6.136	313.2618	P
159	20-COOH-Leukotriene B4	6.142	366.2042	P
160	16b-Hydroxyestrone	6.147	286.1569	P
161	(*Z*)-6-Nonenal	6.151	140.1202	P
162	Penbutolol	6.178	291.2202	P
163	Marimastat	6.180	331.2123	P
164	4-Hydroxy-3-methoxy-2,10-bisaboladien-9-one	6.210	266.1882	P
165	(10*beta*H,11xi)-11-Hydroxy-13-nor-6-eremophilen-8-one	6.218	222.1619	P
166	*alpha*-Butyl-*omega*-hydroxypoly(oxyethylene) poly(oxypropylene)	6.276	248.1989	P
167	Chalciporone	6.292	243.1625	P
168	Gravelliferone	6.305	298.1566	P
169	Heliosupine	6.306	397.2102	P
170	*N*,2,3-Trimethyl-2-(1-methylethyl)butanamide	6.338	171.1621	P
171	1,1-Diethoxy-2-hexene	6.354	172.1464	P
172	Cuscohygrine	6.372	224.1891	P
173	Dihydrocapsaicin	6.388	307.2151	P
174	Metoprolol	6.397	267.1837	P
175	8-Acetoxy-4-acoren-3-one	6.432	278.1882	P
176	10-hydroperoxy-8*E*,12*Z*-octadecadienoic acid	6.444	312.2304	P
177	Lithocholic acid sulfate	6.512	456.2559	P
178	5-*O*-β-D-Mycaminosyltylonolide	6.534	597.3488	P
179	9-HOTE	6.593	294.2197	P
180	Chaksine	6.604	450.2969	P
181	10-Hydroxy-2,8-decadiene-4,6-diynoic acid	6.674	176.0474	P
182	C16 Sphinganine	6.683	273.2670	P
183	Sphinganine	6.683	301.2982	P

184	Acetyllycopsamine	6.697	341.1841	P
185	3-Hydroxy-6,8-dimethoxy-7(11)-eremophilen-12,8-olide	6.702	310.1771	P
186	17-Methylandrosta-2,4-dieno[2,3-d]isoxazol-17*beta*-ol	6.708	327.2204	P
187	Phenethyl decanoate	6.708	276.2090	P
188	Prochlorperazine	6.714	373.1392	P
189	2-Tetradecanone	6.715	212.2141	P
190	5-(2,3-Dihydroxy-3-methylbutyl)-4-(3,4-epoxy-4-methylpentanoyl)-3,4-dihydroxy-2-isopentanoyl-2-cyclopenten-1-one	6.735	412.2099	P
191	Tigloidine	6.756	223.1572	P
192	Phytosphingosine	6.757	317.2931	P
193	2-Methoxy-estradiol-17β 3-glucuronide	6.758	478.2182	P
194	Ximelagatran	6.758	473.2628	P
195	Funtumine	6.762	317.2719	P
196	3*beta*-Hydroxypregn-5-ene	6.778	302.2610	P
197	Mycalamide B	6.781	517.2886	P
198	3',4',5'-Trimethoxycinnamyl alcohol acetate	6.783	266.1153	P
199	Cinegalline	6.784	430.2103	P
200	Erysothiopine	6.785	407.1023	P
201	Melleolide	6.785	400.1884	P
202	Porson	6.785	386.1728	P
203	Panaquinquecol 1	6.789	292.2040	P
204	16-hydroxyhexadecanoic acid	6.792	272.2350	P
205	2-Pentadecanone	6.855	226.2299	P
206	(*S*)-Nerolidol 3-*O*-[a-L-Rhamnopyranosyl-(1->4)-a-L-rhamnopyranosyl-(1->2)-b-D-glucopyranoside]	6.864	676.3670	P
207	Palmitic amide	6.879	255.2564	P
208	5-Dodecyldihydro-2(3H)- furanone	6.883	254.2249	P
209	1-Methyl-2-nonyl-4(1H)-quinolinone	6.894	285.2092	P
210	Pumiliotoxin 251D	6.895	251.2249	P
211	Genipin 1-*beta*-gentiobioside	6.902	550.1900	P

212	1-Tridecene	6.918	182.2035	P
213	2-Hexadecanone	6.933	240.2453	P
214	Diclomezine	6.944	254.0004	P
215	4,4-Difluoropregn-5-ene-3,20-dione	6.945	350.2068	P
216	Arachidonyl Trifluoromethyl Ketone	6.973	356.2312	P
217	Plantaricin BN	6.989	484.2309	P
218	Nonyl octanoate	6.992	270.2560	P
219	Muricatacin	6.993	284.2353	P
220	2,6-Di-tert-butyl-4-ethylphenol	7.034	234.1985	P
221	2,4-Dihydroxyacetophenone 5-sulfate	7.042	232.0049	P
222	BILA 2185BS	7.044	618.3250	P
223	Cyclotetradecane	7.061	196.2192	P
224	Imidaprilat	7.084	377.1585	P
225	Myxochelin A	7.088	404.1586	P
226	Terbucarb	7.094	277.2040	P
227	Proansamitocin	7.096	443.2299	P
228	Spiroxamine	7.099	297.2666	P
229	Armillaripin	7.123	414.2043	P
230	Finaconitine	7.127	630.3160	P
231	1-Pentadecene	7.143	210.2346	P
232	*cis*-5-Tetradecenoylcarnitine	7.198	370.2968	P
233	(9*Z*,11*R*,12*S*,13*S*,15*Z*)-12,13-Epoxy-11-hydroxy-9,15-octadecadienoic acid	7.229	310.2145	P
234	Cincassiol B	7.244	400.2101	P
235	Armillaric acid	7.245	416.1834	P
236	Dihomo-γ-linolenoyl-EA	7.246	349.2965	P
237	Allopumiliotoxin 267A	7.251	267.2199	P
238	Dodecylguanidine	7.262	227.2367	P
239	Bleekerine	7.317	409.1756	P
240	6-*trans*-LTB4	7.359	336.2298	P

241	9-Decenoylcholine	7.381	256.2277	P
242	3-Methyl-*alpha*-ionyl acetate	7.382	250.1934	P
243	*N*-Dealkylatedtolterodine	7.382	283.1937	P
244	Arachidonic Acid (d8)	7.390	312.2893	P
245	12-HETE	7.403	320.2350	P
246	Avocadenofuran	7.406	248.2142	P
247	7,10-Hexadecadienoic acid	7.411	252.2090	P
248	1,8-Heptadecadiene-4,6-diyne-3,10-diol	7.413	260.1776	P
249	Physagulin C	7.438	542.2502	P
250	Etiocholan-3α-ol-17-one 3-glucuronide	7.458	466.2566	P
251	(4-Methylphenyl)acetaldehyde	7.462	134.0730	P
252	*Alpha*-Methylstyrene	7.462	118.0783	P
253	Artabsinolide A	7.462	280.1312	P
254	Austalide L	7.462	428.2200	P
255	Cyclocalopin F	7.462	294.1104	P
256	DHAP(18:0)	7.462	436.2600	P
257	Picrasin C	7.462	422.2298	P
258	(3'x,5'a,9'x,10'b)-*O*-(3-Hydroxy-6-oxo-7-drimen-11-yl)umbelliferone	7.463	396.1937	P
259	2,2-Dimethyl-3,4-bis(4-methoxyphenyl)-2H-1-benzopyran-7-ol acetate	7.463	430.1779	P
260	Erythroskyrin	7.463	455.2310	P
261	Methyl (9*Z*)-10'-oxo-6,10'-diapo-6-carotenoate	7.504	312.1726	P
262	Norpropoxyphene	7.515	325.2043	P
263	Biperiden	7.678	311.2254	P
264	Piperolein B	7.678	343.2143	P
265	1-(4-Amino-2-methylpyrimid-5-ylmethyl)-3-(*beta*hydroxyethyl)-2-methylpyridinium	7.750	259.1547	P
266	Zucchini factor B	7.768	663.4305	P
267	2,2,6,6-Tetramethyl-4-piperidinone	7.776	155.1304	P
268	6,10,14-Trimethyl-5,9,13-pentadecatrien-2-one	7.776	262.2296	P
269	Methyloctatropine	7.791	282.2433	P

270	Phlegmarine	7.836	250.2409	P
271	Methadone	7.876	309.2098	P
272	Estrane-3α,17α-diol	7.882	278.2248	P
273	Elaiophylin	7.942	1024.593<br>2	P
274	2,4,12-Octadecatrienoic acid isobutylamide	7.951	333.3017	P
275	Dodecanamide	7.960	199.1936	P
276	Santalyl acetate	7.964	262.1933	P
277	Asparagoside D	7.965	902.4875	P
278	2-Methoxyestradiol-3-methylether	7.993	316.2023	P
279	Scopoloside II	8.002	770.4092	P
280	Stearamide	8.015	283.2874	P
281	2-Methoxyestrone 3-sulfate	8.016	380.1312	P
282	MG(0:0/18:1(11*Z*)/0:0)	8.016	356.2926	P
283	Leucomycin A9	8.018	743.4090	P
284	Corchorusoside B	8.034	682.3563	P
285	Methyl 15-cyanopentadecanoate	8.055	281.2354	P
286	Pristanic acid	8.060	298.2868	P
287	Convallatoxin	8.094	550.2779	P
288	Dihydro-5-(2-octenyl)-2(3H)-furanone	8.117	196.1466	P
289	Ethyl (4*Z*)-4,7-octadienoate	8.121	168.1151	P
290	Undecylprodigiosin	8.171	393.2778	P
291	2,2,7,7-Tetramethyl-1,6-dioxaspiro[4.4]non-3-ene	8.192	182.1310	P
292	Lyngbyatoxin	8.273	437.3047	P
293	Erinacine G	8.274	464.2419	P
294	17*beta*-Acetamidoandrost-4-en-3-one	8.277	329.2355	P
295	Pipercitine	8.277	349.3329	P
296	p-Mentha-1,3,5,8-tetraene	8.277	132.0940	P
297	6-Oxocineole	8.278	168.1152	P

298	Santene	8.279	122.1095	P
299	2-(4-Methylphenyl)-2-propanol	8.280	150.1046	P
300	MG(0:0/20:2(11*Z*,14*Z*)/0:0)	8.292	382.3080	P
301	1-Phenyl-1,3-dodecanedione	8.312	274.1934	P
302	Isopentylideneisopentylamine	8.343	155.1675	P
303	Lentiginosine	8.343	157.1103	P
304	*Cis*-1,2-Dihydro-3-ethylcatechol	8.344	140.0836	P
305	1-Methyl-1,3-cyclohexadiene	8.345	94.0780	P
306	Tropine	8.348	141.1153	P
307	*N*-Methylpelletierine	8.358	155.1311	P
308	Isometheptene	8.359	141.1516	P
309	Methyl 2-octynoate	8.359	154.0994	P
310	2-Decylfuran	8.372	208.1827	P
311	Tributyl phosphate	8.387	266.1647	P
312	Kukoamine D	8.404	530.3122	P
313	SB 221284	8.411	353.0818	P
314	Triphenyl phosphate	8.411	326.0708	P
315	MK-129	8.413	367.0971	P
316	(*Z*)-9-Cycloheptadecen-1-one	8.416	250.2297	P
317	Methyprylon	8.449	183.1260	P
318	Carpaine	8.454	478.3769	P
319	Dioctyl hexanedioate	8.471	370.3084	P
320	Momilactone B	8.486	330.1833	P
321	12*S*-HEPE	8.510	318.2196	P
322	3L,7D,11D-phytanic acid	8.513	312.3030	P
323	Polidocanol	8.515	582.4343	P
324	*N*-(14-Methylhexadecanoyl)pyrrolidine	8.523	323.3190	P
325	Dodemorph	8.537	281.2721	P
326	(3a,5b,7a,12a)-24-[(carboxymethyl)amino]-1,12-dihydroxy-24-oxocholan-3-yl-b-D-Glucopyranosiduronica	8.545	641.3411	P

327	8,8-Diethoxy-2,6-dimethyl-2-octanol	8.545	246.2195	P
328	Protoprimulagenin A 3-[rhamnosyl-(1->4)-rhamnosyl-(1->4)-[rhamnosyl-(1->2)]-glucosyl-(1->?)-glucuronide]	8.547	1234.6252	P
329	Ergostan-3β-ol	8.548	402.3842	P
330	Oleyl alcohol	8.560	268.2767	P
331	Vaccenyl carnitine	8.604	425.3501	P
332	Polysorbate 20	8.617	522.3403	P
333	Stearoylethanolamide	8.686	327.3141	P
334	Tecostanine	8.743	183.1626	P
335	Polysorbate 60	8.768	434.2884	P
336	Laserpitin	8.769	450.2619	P
337	Hexyl heptanoate	8.789	638.2367	P
338	2-oxophytanic acid	8.813	326.2827	P
339	Thromboxane	8.842	296.3079	P
340	9-Acetoxyfukinanolide	8.866	292.1673	P
341	13-heptadecyn-1-ol	8.882	252.2453	P
342	*trans*-9, *trans*-11-octadecadienoic acid; C18:2n-7,9	8.883	280.2405	P
343	Nitramine	8.887	169.1466	P
344	β-Caryophyllene Alcohol	8.912	222.1986	P
345	MG(0:0/20:1(11*Z*)/0:0)	8.922	384.3241	P
346	Tris(butoxyethyl)phosphate	8.929	398.2433	P
347	Phytal	8.989	294.2924	P
348	3-Cyclohexyldodecane	9.010	252.2818	P
349	Isoacitretin	9.039	326.1883	P
350	(*E,E*)-1,6-bis(4-methoxyphenyl)-1,5-hexadiene	9.041	294.1619	P
351	MG(0:0/22:5(4*Z*,7*Z*,10*Z*,13*Z*,16*Z*)/0:0)	9.050	404.2924	P
352	Linalyl phenylacetate	9.082	272.1774	P
353	*Alpha*-CEHC	9.109	278.1519	P
354	22-Oxo-docosanoate	9.133	354.3133	P

355	*N*-n-Hexanoylglycine methyl ester	9.136	187.1209	P
356	Anofinic acid	9.157	204.0786	P
357	(*E*)-1-[4-Hydroxy-3-(3-methyl-1,3-butadienyl)phenyl]-2-(3,5-dihydroxyphenyl)ethylene	9.162	294.1252	P
358	MG(0:0/22:2(13*Z*,16*Z*)/0:0)	9.168	410.3399	P
359	(6*beta*,7*alpha*,12*beta*,13*beta*)-7-Hydroxy-11,16-dioxo-8,14-apianadien-22,6-olide	9.192	384.1941	P
360	*N*-Benzoyl-D-arginine-4-nitroanilide	9.206	398.1703	P
361	1-(3-Hydroxy-4-methoxyphenyl)-1,2-ethanediol	9.209	184.0737	P
362	Misoprostol	9.209	382.2703	P
363	Tsangane L 3-glucoside	9.209	374.2303	P
364	Neogrifolin	9.257	328.2404	P
365	Linoleoyl Ethanolamide	9.294	323.2828	P
366	10-Eicosene	9.331	280.3130	P
367	Pravastatin	9.366	424.2462	P
368	Bioresmethrin	9.369	338.1882	P
369	Chloropyramine	9.369	289.1357	P
370	Cyclopassifloside II	9.371	682.4264	P
371	MG(0:0/16:0/0:0)	9.374	330.2772	P
372	Lauroyl peroxide	9.376	398.3394	P
373	Acidissiminol epoxide	9.394	409.2253	P
374	MG(0:0/22:6(4*Z*,7*Z*,10*Z*,13*Z*,16*Z*,19*Z*)/0:0)	9.426	402.2756	P
375	(3b,6b,8b,12a)-8,12-Epoxy-7(11)-eremophilene-6-angeloyloxy-8,12-dimethoxy-3-ol	9.431	394.2358	P
376	6-Hydroxy-8-docosanone	9.431	340.3343	P
377	Methandriol dipropionate	9.431	416.2913	P
378	PE(14:0/18:3(6*Z*,9*Z*,12*Z*))	9.431	685.4667	P
379	[6]-Gingerdiol 3,5-diacetate	9.433	380.2202	P
380	(*E*)-3-(2-Hydroxyphenyl)-2-propenal	9.435	148.0524	P
381	Calendulaglycoside E	9.435	794.4342	P
382	Lilac alcohol	9.435	170.1309	P
383	Phenkapton	9.435	375.9367	P

384	Iriomoteolide 1a	9.450	506.3219	P
385	3-(5,6,6-Trimethylbicyclo[2.2.1]hept-1-yl)cyclohexanol	9.456	236.2139	P
386	Lucidenic acid M	9.456	462.2959	P
387	MG(0:0/18:3(6*Z*,9*Z*,12*Z*)/0:0)	9.462	352.2616	P
388	Trimethaphan	9.490	365.1702	P
389	Galbanic acid	9.497	398.2095	P
390	5,10-Pentadecadien-1-ol	9.510	224.2140	P
391	6*alpha*-Fluoropregn-4-ene-3,20-dione	9.596	332.2141	P
392	Monocrotaline	9.644	325.1529	P
393	2-(4-Chloro-3,5-dimethylphenoxy)-*N*-(2-phenyl-2H-benzotriazol-5-yl)-acetamide	9.645	406.1198	P
394	4*beta*-(2-Aminoethylthio)catechin	9.645	365.0926	P
395	Palmitoyl glucuronide	9.735	418.2934	P
396	Ethyl abietate	9.746	330.2555	P
397	Lycopersiconol	9.770	334.2507	P
398	Oleoyl Ethanolamide	9.791	325.2982	P
399	10,16-dihydroxy-palmitic acid	9.798	288.2300	P
400	Palmitoyl-EA	9.798	299.2825	P
401	MG(0:0/22:1(13*Z*)/0:0)	9.836	412.3554	P
402	(3*S*,6*E*,10*E*)-1,6,10,14-Phytatetraen-3-ol	9.871	290.2609	P
403	2,5-Furandicarboxylic acid	9.943	156.0060	P
404	4-Carboxy-2-hydroxy-6-methoxy-6-oxohexa-2,4-dienoate	9.944	216.0272	P
405	Arbutin	9.944	272.0899	P
406	Asteltoxin	9.944	418.1992	P
407	Cymorcin monoglucoside	9.944	328.1525	P
408	Vanillactic acid	9.944	212.0686	P
409	1b,3a,7a,12a-Tetrahydroxy-5b-cholanoic acid	9.945	424.2811	P
410	Acetyl tributyl citrate	9.945	402.2253	P
411	Ampalex	9.946	241.1204	P
412	Kamahine C	9.946	268.1314	P

413	Gorgostane skeleton	10.089	412.4050	P
414	Luffariellolide	10.094	386.2821	P
415	Kanzonol L	10.110	488.2198	P
416	Balofloxacin	10.188	389.1758	P
417	Hellebrin	10.192	724.3301	P
418	DU 122290	10.201	362.1652	P
419	(1*alpha*,3*beta*,20*S*,22*R*,24*S*,25*S*)-Pubescenin	10.291	620.3569	P
420	Oleandrin	10.354	576.3295	P
421	Drotaverine	10.381	397.2255	P
422	Ganoderic acid I	10.421	532.3043	P
423	7-Ketodeoxycholic acid	10.428	406.2699	P
424	1,2-Epoxypropane	10.490	58.0418	P
425	Dodecylbenzene	10.490	246.2347	P
426	(±)-(*Z*)-2-(5-Tetradecenyl)cyclobutanone	10.491	264.2453	P
427	Oleamide	10.491	281.2721	P
428	Lucidumol A	10.506	472.3554	P
429	DG(18:1(11*Z*)/22:5(4*Z*,7*Z*,10*Z*,13*Z*,16*Z*)/0:0)	10.554	668.5406	P
430	DG(22:6(4*Z*,7*Z*,10*Z*,13*Z*,16*Z*,19*Z*)/20:3(8*Z*,11*Z*,14*Z*)/0:0)	10.554	690.5224	P
431	DG(20:3(5*Z*,8*Z*,11*Z*)/22:6(4*Z*,7*Z*,10*Z*,13*Z*,16*Z*,19*Z*)/0:0)	10.555	690.5225	P
432	4-Methoxycinnamic acid	10.559	178.0632	P
433	DG(14:0/22:4(7*Z*,10*Z*,13*Z*,16*Z*)/0:0)	10.564	616.5039	P
434	(9*Z*,11*E*,13*E*,15*Z*)-4-Oxo-9,11,13,15-octadecatetraenoic acid	10.565	290.1883	P
435	Capsi-amide	10.586	269.2720	P
436	4-Nerolidylcatechol	10.595	314.2246	P
437	Drospirenone	10.686	366.2195	P
438	D-Glucosyldihydrosphingosine	10.809	463.3512	P
439	2-Pentadecylfuran	10.834	278.2610	P
440	Enalkiren	10.846	656.4285	P
441	b-Hydroxypropionyl-CoA	10.897	839.1324	P

442	Cavipetin D	10.898	418.2720	P
443	Diprenorphine	10.899	425.2569	P
444	Sorbitan palmitate	10.899	402.2982	P
445	Ganodermic acid TQ	11.096	510.3345	P
446	5-*beta*-cholestan-3-one	11.231	386.3538	P
447	*Beta*-Elemonic acid	11.374	454.3448	P
448	*N*-Hexadecanoylpyrrolidine	11.390	309.3035	P
449	MG(18:0/0:0/0:0)	11.510	358.3086	P
450	DG(20:5(5*Z*,8*Z*,11*Z*,14*Z*,17*Z*)/24:1(15*Z*)/0:0)	11.511	724.6032	P
451	Tridemorph	11.533	297.3033	P
452	Ethyl decanoate	11.536	200.1777	P
453	PC(14:0/22:5(4*Z*,7*Z*,10*Z*,13*Z*,16*Z*))	12.258	780.5541	P
454	12-Ketodeoxycholic acid	12.261	390.2771	P
455	PC(16:0/18:1(9*Z*))[*S*]	12.273	760.5849	P
456	Testosterone isocaproate	12.287	386.2818	P
457	Glycerol 1-(9Zoctadecenoate)2-tetradecanoate 3-phosphate	13.121	646.4590	P
458	DG(14:0/22:1(13*Z*)/0:0)	14.144	622.5532	P
459	Epifisetinidol-(4*beta*->8)-epicatechin-(6->4*beta*)-epifisetinidol	14.915	834.2151	P
460	DG(14:0/24:1(15*Z*)/0:0)	15.832	650.5847	P

*-acetonitrile: water (1;1, *v/v*)

[a] – retention time [min]

[b] –compound detection in positive (P) or in negative (N) ionization mode.
