# Supplementary material for: Biological Properties of the Mucus and Eggs of Helix aspersa Müller as a Potential Cosmetic and Pharmaceutical Raw Material: A Preliminary Study
Source: Int J Mol Sci. 2024 Sep 15;25(18):9958. doi: 10.3390/ijms25189958 (PMC11432642; doi:10.3390/ijms25189958)
Supplement: Supplementary file 1 [file ijms-25-09958-s001.zip › Herman Anna - Table S16.pdf]

Table S16. Percentage of necrotic, late apoptotic, early apoptotic and live MCF-7 and HTC-116 cells after treatment with SE. The data were determined by Accuri C6 Plus flow cytometer after 72 h of treatment with cisplatin. Cells were stained with annexin V-FITC and PI (propidium iodide).

		NECROSIS	LATE APOPTOSIS	EARLY APOPTOSIS	LIVE
**MCF-7**	CTRL, 25% MilliQ water	0.60±0.14	6.28±2.52	0.85±0.22	92.27±2.68
	SE, 6.25 m/ml	1.97±0.56	9.23±1.55	3.99±0.89	84.81±2.58
	SE, 12.5 mg/ml	3.05±0.86	16.40±1.98	11.37±2.68	69.18±4.30
	SE, 25 mg/ml	2.41±0.27	17.22±0.73	13.19±0.28	67.18±0.46
**HTC-116**	CTRL, 25% MilliQ water	1.52±0.15	6.20±0.63	7.10±0.29	85.18±0.78
	SE, 6.25 m/ml	4.47±0.86	16.33±1.29	8.76±1.59	70.44±2.01
	SE, 12.5 mg/ml	4.30±0.40	26.97±2.16	21.23±2.18	47.51±2.85
	SE, 25 mg/ml	3.99±1.10	24.97±1.32	31.50±2.50	39.54±2.47

Cisplatin (Sigma-Aldrich Chemical Company, St. Louis, MO, USA) was used as a reference compound in MTT assay as well as in annexin V-binding assay. Sigmoidal dose-response curves for cisplatin determined for MCF-7, HT-29, HCT-116 and Vero cells after 72 h of treatment were shown on Figure S1.

IC50 values determined for cisplatin for all tested cell lines are summarized in the Table S17.

The data obtained for V-binding assay for cisplatin treatment were shown in the Table S18.
