# Supplementary material for: Biological Properties of the Mucus and Eggs of Helix aspersa Müller as a Potential Cosmetic and Pharmaceutical Raw Material: A Preliminary Study
Source: Int J Mol Sci. 2024 Sep 15;25(18):9958. doi: 10.3390/ijms25189958 (PMC11432642; doi:10.3390/ijms25189958)
Supplement: Supplementary file 1 [file ijms-25-09958-s001.zip › Herman Anna - Table S17.pdf]

Table S17. Viability of MCF-7, HT-29, HCT-116 and Vero cells after 72 h of treatment. The IC50 [μM] values were calculated using an MTT-based assay and an equation: Y=100/(1+10^((LogIC50–X)*HillSlope)). 7 concentrations of cisplatin was used in 2-fold series dilution in the range of 1.56 to 100 μM.

**Cell line**	**MCF-7**	**HT-29**	**HCT-116**	**Vero**
**IC_{50} [μM]**	15.75	12.68	14.27	12.76
