# Supplementary material for: Biological Properties of the Mucus and Eggs of Helix aspersa Müller as a Potential Cosmetic and Pharmaceutical Raw Material: A Preliminary Study
Source: Int J Mol Sci. 2024 Sep 15;25(18):9958. doi: 10.3390/ijms25189958 (PMC11432642; doi:10.3390/ijms25189958)
Supplement: Supplementary file 1 [file ijms-25-09958-s001.zip › Herman Anna - Table S18.pdf]

Table S18. Percentage of necrotic, late apoptotic, early apoptotic and live MCF-7 and HTC-116 cells after treatment with cisplatin used in 0.5xIC50, IC50 and 2xIC50. The data were determined by Accuri C6 Plus flow cytometer after 72 h of treatment with cisplatin. Cells were stained with annexin V-FITC and PI (propidium iodide).

		NECROSIS	LATE APOPTOSIS	EARLY APOPTOSIS	LIVE
**MCF-7**	CTRL	1.26±0.66	7.60±4.71	0.62±0.10	90.53±5.39
	Cisplatin, 0.5xIC50	3.06±1.63	11.77±2.54	5.43±0.78	79.75±3.75
	Cisplatin, IC50	0.58±0.07	7.84±1.49	15.12±2.25	76.45±0.99
	Cisplatin, 2xIC50	0.98±0.12	5.43±0.50	30.34±2.27	63.26±2.71
**HTC-116**	CTRL	1.35±0.29	4.59±0.93	1.95±0.35	92.11±1.53
	Cisplatin, 0.5xIC50	3.31±0.22	11.96±2.28	15.02±3.29	69.71±5.47
	Cisplatin, IC50	3.73±0.80	18.27±1.53	28.83±2.40	49.17±2.93
	Cisplatin, 2xIC50	5.52±0.76	23.94±2.36	41.50±1.86	29.03±0.84
