# Supplementary material for: Biological Properties of the Mucus and Eggs of Helix aspersa Müller as a Potential Cosmetic and Pharmaceutical Raw Material: A Preliminary Study
Source: Int J Mol Sci. 2024 Sep 15;25(18):9958. doi: 10.3390/ijms25189958 (PMC11432642; doi:10.3390/ijms25189958)
Supplement: Supplementary file 1 [file ijms-25-09958-s001.zip › Herman Anna - Table S2.pdf]

Table S2. Compounds identified in methanolic extract of fresh egg of organic *Helix aspersa* snail using LC-MS.

No	Metabolite	RT[a][min]	Mass [*m/z*]	Detection mode [b]
1	3-b-Galactopyranosyl glucose	0.266	342.1162	N
2	2,4,6-Octatriynoic acid	0.274	132.0210	N
3	Dimethyl carbonate	0.369	90.0318	N
4	*N*-n-Hexanoylglycine methyl ester	3.464	187.1210	N
5	Methyl N-(a-methylbutyryl)glycine	4.297	188.1050	N
6	D-Ribose 1-diphosphate	5.62	293.9905	N
7	Cinnzeylanol	5.628	384.2149	N
8	Ethiprole	5.805	395.9833	N
9	Zingerone	6.228	194.0944	N
10	Bismuth subsalicylate	6.708	361.9979	N
11	Nordihydrocapsiate	6.831	294.1832	N
12	3-Hydroxy-6,8-dimethoxy-7(11)-eremophilen-12,8-olide	7.033	310.1782	N
13	BILA 2185BS	7.038	618.3253	N
14	4-Hydroxy-5-phenyltetrahydro-1,3-oxazin-2-one	7.055	193.0740	N
15	(*S,Z*)-Lyratol acetate	7.114	194.1309	N
16	Erinacine G	7.118	464.2390	N
17	3b-Allotetrahydrocorticosterone	7.119	350.2459	N
18	(3b,6b,8b,12a)-8,12-Epoxy-7(11)-eremophilene-6-angeloyloxy-8,12-dimethoxy-3-ol	7.201	394.2362	N
19	Zeleplon	7.281	305.1271	N
20	Lauryl hydrogen sulfate	7.282	266.1552	N
21	Methotrexate	7.313	454.1734	N
22	Losartan	7.315	422.1623	N
23	L-Tyrosine methyl ester	7.343	195.0897	N
24	Dinoterb	7.577	240.0747	N
25	*N*-Undecylbenzenesulfonic acid	7.728	312.1761	N
26	2-Dodecylbenzenesulfonic acid	8.165	326.1914	N

27	Sodium Tetradecyl Sulfate	8.205	294.1864	N
28	(+)-Prosopinine	8.275	313.2617	N
29	Kukoamine D	8.41	530.3120	N
30	(10*beta*H,11xi)-11-Hydroxy-13-nor-6-eremophilen-8-one	8.53	222.1624	N
31	Gemfibrozil	8.957	250.1570	N
32	Furmecyclox	9.279	251.1521	N
33	2-(2-Methylbutanoyl)-9-(3-methyl-2E-pentenoyl)-2b,9a-dihydroxy-4*Z*,10(14)-oplopadien-3-one	9.904	430.2721	N
34	3-Oxochola-4,6-dien-24-oic acid	10.248	370.2509	N
35	(5b,7a,12a)-2-(3-methoxyphenyl)-2-oxoethyl ester-7,12-dihydroxy-cholan-24-oic acid	10.295	540.3452	N
36	Methyl tetradecanoate	10.501	242.2246	N
37	5-Dodecyldihydro-2(3H)-furanone	10.503	254.2245	N
38	Schidigeragenin B	10.547	428.2943	N
39	DG(18:1(11*Z*)/22:5(4*Z*,7*Z*,10*Z*,13*Z*,16*Z*)/0:0)	10.55	668.5403	N
40	DG(20:3(5*Z*,8*Z*,11*Z*)/22:6(4*Z*,7*Z*,10*Z*,13*Z*,16*Z*,19*Z*)/0:0)	10.55	690.5216	N
41	(3*R*,2'*S*)-Myxol 2'-(2,4-di-*O*-methyl-α-L-fucoside)	10.551	758.5090	N
42	MG(0:0/16:0/0:0)	10.551	330.2770	N
43	Piritramide	10.553	430.2737	N
44	Enalkiren	10.844	656.4284	N
45	3-Hydroxy-2-(4-morpholinylmethyl)estra-1,3,5(10)-trien-17-one	10.948	369.2299	N
46	Butroxydim	11.27	399.2409	N
47	Adlupone	11.365	482.3395	N
48	Drotaverine	11.452	397.2251	N
49	Callystatin A	11.505	456.3258	N
50	DG(20:5(5*Z*,8*Z*,11*Z*,14*Z*,17*Z*)/24:1(15*Z*)/0:0)	11.505	724.6028	N
51	Pubesenolide	11.506	458.3048	N
52	MG(18:0/0:0/0:0)	11.507	358.3077	N
53	(22*E*,24*R*)-Stigmasta-4,22-diene-3,6-dione	11.963	424.3340	N
54	5-Heptadecyl-1,3-benzenediol	11.994	348.3028	N
55	24-Acetyl-25-cinnamoylvulgaroside	12.065	608.3351	N

56	6,8a-Seco-6,8a-deoxy-5-oxoavermectin "2a" aglycone	12.066	586.3530	N
57	Momordicinin	12.36	438.3497	N
58	(3*beta*,22*E*,24*R*)-3-Hydroxyergosta-5,8,22-trien-7-one	12.503	410.3184	N
59	3-Deoxy-D-glycero-Dgalacto-2-nonulosonic acid	13.969	268.0792	N
1	Bufexamac	0.138	223.1210	P
2	2-Amino-2-methyl-1,3-propanediol	0.253	105.0790	P
3	Trolamine	0.255	149.1052	P
4	3-Hydroxyisoheptanoic acid	0.257	146.0944	P
5	Aminocaproic acid	0.266	131.0947	P
6	Melamine	0.267	126.0658	P
7	4-Amino-2-methylenebutanoic acid	0.272	115.0633	P
8	(3-Phenylpropionyl)glycinemethyl ester	0.273	221.1052	P
9	7-Ethyl-2,3,6,7-tetrahydrocyclopent[b]azepin-8(1H)-one	0.273	177.1155	P
10	Triethylamine	0.275	101.1206	P
11	3-Deoxy-D-mannooctulosonate	0.276	238.0687	P
12	Nicotinamide N-oxide	0.276	138.0431	P
13	(2*R**,3*R**)-1,2,3-Butanetriol	0.371	106.0627	P
14	(2*R*,3*R*,4R)-2-Amino-4-hydroxy-3-methylpentanoic acid	0.385	147.0896	P
15	3-Hydroxysuberic acid	0.715	190.0842	P
16	Dexpanthenol	0.855	205.1315	P
17	2-Amino-4-hydroxy-6-(hydroxymethyl)-7,8-dihydropteridine	0.857	195.0761	P
18	2,5-Dihydro-2,4,5-trimethyloxazole	1.46	113.0841	P
19	Amyl 2-furoate	2.287	182.0944	P
20	Propionyl-L-carnitine	2.302	218.1393	P
21	2*E*-Decenedioic acid	2.33	200.1049	P
22	5-Heptyltetrahydro-2-oxo-3-furancarboxylic acid	2.953	228.1361	P
23	3-hydroxytetradecanedioic acid	3.002	274.1783	P
24	Geranyl acetoacetate	3.029	238.1571	P
25	Sedanonic acid	3.124	210.1256	P

26	Meteloidine	3.275	255.1471	P
27	Wine lactone	3.29	166.0993	P
28	*N*-n-Hexanoylglycine methyl ester	3.463	187.1209	P
29	Homoarecoline	3.464	169.1103	P
30	Istamycin C1	3.584	431.2732	P
31	2,3-Dihydro-5-(5-methyl-2-furanyl)-1H-pyrrolizine	3.596	187.0999	P
32	Phlorin	3.705	288.0845	P
33	Threo-Syringoylglycerol	3.709	244.0947	P
34	Netilmicin	3.735	475.2996	P
35	Monomenthyl succinate	3.851	256.1676	P
36	2-Methyl-1-phenyl-2-propanyl acetate	3.922	192.1149	P
37	Kinetin	3.923	215.0810	P
38	Tributyrin	4.022	302.1732	P
39	Portuloside A	4.036	330.1677	P
40	4,4-Difluoro-17*beta*hydroxyandrost-5-en-3-one propionate	4.044	380.2174	P
41	6-Epi-7-isocucurbic acid glucoside	4.213	374.1932	P
42	*N*-Methylmescaline	4.257	225.1368	P
43	Jasmine ketolactone	4.258	208.1102	P
44	Octyl gallate	4.295	282.1466	P
45	2,2,7,7-Tetramethyl-1,6-dioxaspiro[4.4]nona-3,8-diene	4.298	180.1152	P
46	Mukaadial	4.3	266.1520	P
47	2-Methyl-4-pentyloxazole	4.353	153.1156	P
48	Oseltamivir	4.39	312.2045	P
49	Triethylenemelamine	4.399	204.1129	P
50	Avenic acid A	4.4	322.1391	P
51	4,11,13,15-Tetrahydroridentin B	4.436	268.1674	P
52	PE(18:4(6*Z*,9*Z*,12*Z*,15*Z*)/22:6(4*Z*,7*Z*,10*Z*,13*Z*,16*Z*,19*Z*))	4.453	783.4830	P
53	1-Octen-3-yl glucoside	4.501	290.172	P
54	(*E*)-3-decen-1-ol	4.55	156.1516	P

55	Ethyl decanoate	4.553	200.1778	P
56	Diethofencarb	4.554	267.1473	P
57	Flumetover	4.555	367.1395	P
58	Humulinic acid A	4.579	266.1519	P
59	Imiquimod	4.634	240.1364	P
60	Ethyl 3-(*N*butylacetamido) propionate	4.65	215.1521	P
61	2-Hexenoylcholine	4.672	200.1655	P
62	1,2,3-Tris(1-ethoxyethoxy)propane	4.673	308.2200	P
63	Ethyl 7-epi-12-hydroxyjasmonate glucoside	4.676	416.2046	P
64	Methyl 7-epi-12-hydroxyjasmonate glucoside	4.676	402.1889	P
65	C12:1n-7	4.684	198.1619	P
66	*Gamma*-CEHC	4.686	248.1414	P
67	Ruscopine	4.727	306.2049	P
68	3-Indolecarboxylic acid	4.731	253.1311	P
69	1-Phenyl-6,7-dihydroxyisochroman	4.732	242.0946	P
70	2,3-dihydrobenzofuran	4.732	120.0575	P
71	2-Ethylacrylylcarnitine	4.732	244.1551	P
72	2-Phenylbutyric acid	4.732	164.0840	P
73	(5*R*)-5-Hydroxyhexanoic acid	4.733	132.0786	P
74	Alanyl-Isoleucine	4.781	202.1319	P
75	*N*-Isobutyl-2,4,8,10,12-tetradecapentaenamide	4.803	273.2095	P
76	Methyl 3-(2,3-dihydroxy-3-methylbutyl)-4-hydroxybenzoate	4.813	254.1156	P
77	Pinidine	4.881	139.1360	P
78	Oleandolide	4.883	386.2306	P
79	Allixin	4.963	226.1204	P
80	Isofucosterol glucoside	4.963	574.4225	P
81	4'-Hydroxy-3,4,5-trimethoxystilbene	4.964	286.1202	P
82	Methyl propionate	4.964	88.0523	P
83	Epothilone B	5.006	507.2679	P

84	2-Phenylethyl *beta*-Dglucopyranoside	5.012	284.1260	P
85	1,1,2-Triphenylpropane	5.024	272.1559	P
86	5,7-Megastigmadien-9-ol glucoside	5.04	356.2191	P
87	Cinncassiol A 19-glucoside	5.047	544.2523	P
88	7,8-Dihydrovomifoliol 9-[rhamnosyl-(1->6)-glucoside]	5.049	534.2680	P
89	Sterebin E	5.077	338.2459	P
90	(*S*)-3-Octanol glucoside	5.105	292.1887	P
91	(-)-*trans*-Carveol glucoside	5.137	314.1733	P
92	Gibberellin A105	5.138	330.1466	P
93	Glycerol 1-(5-hydroxydodecanoate)	5.231	290.2097	P
94	Toxin T2 tetrol	5.243	298.1418	P
95	Cyclonormammein	5.274	374.1727	P
96	Jasmolone glucoside	5.372	342.1680	P
97	4-Butyl-5-ethylthiazole	5.424	169.0924	P
98	AF Toxin II	5.431	324.1573	P
99	2-Furanmethanol	5.433	98.0368	P
100	Taraxacolide 1-*O*-b-Dglucopyranoside	5.447	428.2042	P
101	Dulciol C	5.476	482.2322	P
102	Hydrocortisone succinate	5.482	462.2251	P
103	Corchoionol C 9-glucoside	5.486	386.1943	P
104	*O*-Methylsomniferine	5.501	622.2650	P
105	(*E,E,E*)-*N*-(2-Methylpropyl)hexadeca-2,6,8-trien-10-ynamide	5.505	301.2404	P
106	Clusin	5.535	402.1676	P
107	Satratoxin H	5.566	528.2339	P
108	Terazosin	5.654	387.1893	P
109	Eremopetasinorol	5.657	208.1464	P
110	Blumenol C glucoside	5.686	372.2152	P
111	(2xi,6xi)-7-Methyl-3-methylene-1,2,6,7-octanetetrol	5.703	204.1336	P
112	Hexanal octane-1,3-diol acetal	5.706	228.2091	P

113	2-Methylundecanal	5.725	184.1829	P
114	(5*alpha*,10*alpha*)-3,7(11)-Eudesmadien-2-one	5.767	218.1671	P
115	Avocadienofuran	5.767	246.1984	P
116	Gibberellin A1 glucosyl ester	5.77	510.2103	P
117	Volicitin	5.772	422.2766	P
118	NAc-FnorLRF-amide	5.774	622.3567	P
119	Blumenol C *O*-[rhamnosyl-(1->6)-glucoside]	5.785	518.2723	P
120	Fluspirilene	5.81	475.2419	P
121	19(*R*)-hydroxy-PGE2	5.814	368.2201	P
122	2-Hydroxymyristic Acid	5.832	244.2038	P
123	Acetyllycopsamine	5.833	341.1837	P
124	Ssioriside	5.836	554.2358	P
125	Glaucamine	5.86	385.1524	P
126	Sanshodiol	5.86	358.1418	P
127	C14:1n-9	5.877	226.1935	P
128	Eriojaposide A	5.88	502.2414	P
129	Canavalioside	5.943	546.2674	P
130	Capsoside A	5.977	694.3773	P
131	(+/-)-*N,N*-Dimethyl menthyl succinamide	6.014	168.1879	P
132	15-Acetoxyscirpene-3,4-diol 4-*O*-a-Dglucopyranoside	6.025	486.2093	P
133	Capsaicin	6.062	305.1990	P
134	Homodihydrojasmone	6.069	180.1513	P
135	16b-Hydroxyestrone	6.08	286.1568	P
136	Ciclesonide	6.111	540.3097	P
137	(+)-Prosopinine	6.137	313.2617	P
138	20-COOH-Leukotriene B4	6.143	366.2042	P
139	(*Z*)-6-Nonenal	6.151	140.1201	P
140	Penbutolol	6.179	291.2201	P
141	Marimastat	6.181	331.2109	P

142	Linalyl propionate	6.204	210.1621	P
143	Pseudoargiopinin III	6.205	373.2101	P
144	10,11-Epoxy-3,7,11-trimethyl-2*E*,6*E*tridecadienoic acid	6.212	266.1883	P
145	Lauroyl diethanolamide	6.224	287.2461	P
146	Chalciporone	6.257	243.1627	P
147	Cincassiol B	6.259	400.2093	P
148	*Alpha*-Butyl-*omega*hydroxypoly(oxyethylene) poly(oxypropylene)	6.273	248.1990	P
149	3'-Hydroxy-HT2 toxin	6.292	440.2046	P
150	Gravelliferone	6.307	298.1570	P
151	*N*,2,3-Trimethyl-2-(1-methylethyl)butanamide	6.338	171.1625	P
152	Plantaricin BN	6.339	484.2307	P
153	1,1-Diethoxy-2-hexene	6.364	172.1462	P
154	Cuscohygrine	6.374	224.1888	P
155	Coccinin	6.384	528.2568	P
156	Dihydrocapsaicin	6.387	307.2146	P
157	Metoprolol	6.397	267.1836	P
158	Diphenylcarbazide	6.511	242.1157	P
159	1-Hydroxyacorenone	6.518	250.1567	P
160	Alantolactone	6.519	232.1462	P
161	Armillatin	6.577	610.4232	P
162	10-Hydroxy-2,8-decadiene-4,6-diynoic acid	6.666	176.0474	P
163	Monoisobutyl phthalic acid	6.666	222.0893	P
164	C16 Sphinganine	6.684	273.2670	P
165	Artabsinolide A	6.691	280.1313	P
166	*p*-Hydroxybenzyldesulphoglucosinolate	6.697	345.0867	P
167	Coutaric acid	6.709	349.2011	P
168	2*alpha*,3*alpha*-(Difluoromethylene)-5a*lpha*-androstan-17*beta*-ol acetate	6.716	366.2369	P
169	2-Tetradecanone	6.716	212.2142	P
170	Glicoisoflavanone	6.716	384.1569	P

171	5-(2,3-Dihydroxy-3-methylbutyl)-4-(3,4-epoxy-4-methylpentanoyl)-3,4-dihydroxy-2-isopentanoyl-2-cyclopenten-1-one	6.736	412.2097	P
172	1-Isomangostin hydrate	6.738	428.1832	P
173	Phytosphingosine	6.757	317.2930	P
174	CB3717	6.758	477.1646	P
175	Ximelagatran	6.759	473.2626	P
176	Mycalamide B	6.782	517.2892	P
177	Trilobolide	6.783	522.2441	P
178	Porson	6.786	386.1732	P
179	16-hydroxy hexadecanoic acid	6.788	272.2354	P
180	Canescein	6.805	566.2703	P
181	Granisetron	6.819	312.1936	P
182	Funtumine	6.846	317.2720	P
183	2-Pentadecanone	6.855	226.2297	P
184	Minocycline	6.863	457.1827	P
185	(*S*)-Nerolidol 3-*O*-[a-LRhamnopyranosyl-(1->4)-a-rhamnopyranosyl-(1->2)-b-Dglucopyranoside]	6.865	676.3669	P
186	17-Methylandrosta-2,4-dieno[2,3-d]isoxazol-17beta-ol	6.865	327.2200	P
187	Cubebininolide	6.865	446.1944	P
188	5-Dodecyldihydro-2(3H)-furanone	6.884	254.2247	P
189	Pumiliotoxin 251D	6.895	251.2248	P
190	Austalide F	6.896	490.2198	P
191	Genipin 1-betagentiobioside	6.901	550.1896	P
192	1-Tridecene	6.915	182.2033	P
193	Zizybeoside II	6.922	594.2162	P
194	Kanokoside C	6.94	638.2422	P
195	Choline chloride	6.949	103.0998	P
196	Chrycolide	6.949	232.0183	P
197	6-Caffeoylsucrose	6.959	504.1485	P
198	Coriandrone D	6.964	352.1522	P

199	4,5-Dihydroniveusin A	6.965	396.1785	P
200	13,14-dihydro-15-keto-PGA2	6.976	334.2142	P
201	Muricatacin	6.99	284.2354	P
202	Nonyl octanoate	6.995	270.2563	P
203	Contignasterol	6.996	508.3386	P
204	Acetyl Tyrosine Ethyl Ester	7.029	251.1157	P
205	3-Methylcyclopentadecanone	7.032	238.2297	P
206	Discodermolide	7.036	593.3908	P
207	Phosphoric acid	7.042	97.9769	P
208	BILA 2185BS	7.045	618.3259	P
209	Cyclotetradecane	7.059	196.2192	P
210	Myxochelin A	7.089	404.1583	P
211	Terbucarb	7.095	277.2042	P
212	Proansamitocin	7.096	443.2288	P
213	Spiroxamine	7.101	297.2669	P
214	6*alpha*,9-Difluoro-11*beta*-hydroxypregn-4-ene-3,20-dione	7.111	366.2017	P
215	2-Methoxyestradiol-3-methylether	7.122	316.2023	P
216	Finaconitine	7.126	630.3144	P
217	7-Hydroxy-3-(4-methoxyphenyl)-4-methylcoumarin	7.187	282.0892	P
218	Z-Gly-Pro-Leu-Gly-Pro	7.191	573.2787	P
219	10,16-dihydroxy-palmitic acid	7.193	288.2303	P
220	Armillaric acid	7.244	416.1831	P
221	Allopumiliotoxin 267A	7.251	267.2199	P
222	Bleekerine	7.317	409.1760	P
223	PGB1	7.364	336.2297	P
224	Panaquinquecol 1	7.367	292.2040	P
225	6-*trans*-LTB4	7.37	336.2304	P
226	1,8-Heptadecadiene-4,6-diyne-3,10-diol	7.398	260.1777	P
227	Armillaripin	7.398	414.2040	P

228	Physagulin C	7.439	542.2503	P
229	Etiocholan-3*α*-ol-17-one 3-glucuronide	7.456	466.2564	P
230	(4-Methylphenyl)acetaldehyde	7.461	134.0732	P
231	(3'x,5'a,9'x,10'b)-*O*-(3-Hydroxy-6-oxo-7-drimen-11-yl)umbelliferone	7.462	396.1936	P
232	2,2-Dimethyl-3,4-bis(4-methoxyphenyl)-2H-1-benzopyran-7-ol acetate	7.462	430.1780	P
233	*Alpha*-Methylstyrene	7.462	118.0783	P
234	DHAP(18:0)	7.462	436.2600	P
235	Erythroskyrin	7.462	455.2309	P
236	Picrasin C	7.462	422.2304	P
237	Austalide L	7.463	428.2201	P
238	Cyclocalopin F	7.463	294.1105	P
239	Methyl (9*Z*)-10'-oxo-6,10'-diapo-6-carotenoate	7.503	312.1726	P
240	Sphinganine	7.6	301.2982	P
241	Vilazodone	7.633	441.2150	P
242	Biperiden	7.678	311.2247	P
243	Austalide B	7.723	474.2262	P
244	Mammea E/BB	7.74	430.1997	P
245	1-(4-Amino-2-methylpyrimid-5-ylmethyl)-3-(*beta*hydroxyethyl)-2-methylpyridinium	7.75	259.1548	P
246	Pentazocine	7.778	285.2087	P
247	6,10,14-Trimethyl-5,9,13-pentadecatrien-2-one	7.779	262.2288	P
248	p-Hydroxyphenethyl *trans*-ferulate	7.785	314.1152	P
249	Methyl 15-cyanopentadecanoate	7.792	281.2356	P
250	Phlegmarine	7.834	250.2411	P
251	Methadone	7.873	309.2092	P
252	Phenethyl decanoate	7.873	276.2080	P
253	Methyloctatropine	7.877	282.2433	P
254	estrane-3*α*,17*α*-diol	7.881	278.224	P
255	Zucchini factor B	7.894	663.4308	P
256	Dihydrodioscorine	7.905	223.1575	P

257	Elaeokanine C	7.906	211.1572	P
258	9-HOTE	7.939	294.2199	P
259	Elaiophylin	7.943	1024.5943	P
260	Gabapentin	7.946	171.1260	P
261	2,4,12-Octadecatrienoic acid isobutylamide	7.95	333.3016	P
262	(3a,5b)-24-oxo-24-[(2-sulfoethyl)amino]cholan-3-yl-b-Dglucopyranosiduronic acid	7.952	659.3344	P
263	Asparagoside D	7.964	902.4879	P
264	Firocoxib	7.997	336.1033	P
265	Scopoloside II	8.002	770.4088	P
266	Stearamide	8.01	283.2876	P
267	MG(0:0/18:1(11*Z*)/0:0)	8.014	356.2933	P
268	2-Methoxyestrone 3-sulfate	8.016	380.1297	P
269	Leucomycin A9	8.018	743.4097	P
270	Corchorusoside B	8.033	682.3566	P
271	1,26-Dicaffeoylhexacosanediol	8.081	722.4753	P
272	Convallatoxin	8.093	550.2778	P
273	Ethyl (4*Z*)-4,7- octadienoate	8.119	168.1153	P
274	2-Hexadecanone	8.148	240.2454	P
275	Undecylprodigiosin	8.174	393.2782	P
276	2,2,7,7-Tetramethyl-1,6-dioxaspiro[4.4]non-3-ene	8.192	182.1309	P
277	Pipericine	8.194	335.3173	P
278	17*beta*-Acetamidoandrost-4-en-3-one	8.274	329.2351	P
279	Lyngbyatoxin	8.274	437.3045	P
280	Pipercitine	8.275	349.3328	P
281	6-Oxocineole	8.277	168.1151	P
282	Tributyl phosphate	8.305	266.1646	P
283	Ponasteroside A	8.31	626.3663	P
284	1-Phenyl-1,3-dodecanedione	8.313	274.1935	P

285	Avocadenofuran	8.334	248.2136	P
286	1-Methyl-1,3-cyclohexadiene	8.34	94.0781	P
287	Isopentylideneisopentylamine	8.342	155.1672	P
288	Lentiginosine	8.342	157.1104	P
289	Methyl 2-octynoate	8.36	154.0994	P
290	4-Vinylcyclohexene	8.361	108.0939	P
291	Homostachydrine	8.367	158.1183	P
292	Kukoamine D	8.406	530.3124	P
293	Triphenyl phosphate	8.41	326.0709	P
294	Methyprylon	8.451	183.1260	P
295	A28086B	8.474	762.4883	P
296	Momilactone B	8.484	330.1833	P
297	12*S*-HEPE	8.509	318.2193	P
298	3L,7D,11D-phytanic acid	8.51	312.3030	P
299	Polidocanol	8.518	582.4343	P
300	*N*-(14-Methylhexadecanoyl)pyrrolidine	8.521	323.3188	P
301	Linoleoyl Ethanolamide	8.523	323.2824	P
302	Mycinamicin VIII	8.523	505.3387	P
303	8,8-Diethoxy-2,6-dimethyl-2-octanol	8.543	246.2192	P
304	(3a,5b,7a,12a)-24-[(carboxymethyl)amino]-1,12-dihydroxy-24-oxocholan-3-yl-b-Dglucopyranosiduronic a	8.544	641.3413	P
305	Allosamidine	8.544	622.2697	P
306	Oleyl alcohol	8.557	268.2765	P
307	Polysorbate 20	8.617	522.3405	P
308	2*E*-Eicosenoic acid	8.686	310.2872	P
309	Laserpitin	8.767	450.2617	P
310	Polysorbate 60	8.767	434.2885	P
311	Hexyl heptanoate	8.787	638.2361	P
312	PS(18:0/22:5(7*Z*,10*Z*,13*Z*,16*Z*,19*Z*))	8.828	837.5561	P
313	9-Acetoxyfukinanolide	8.863	292.1673	P

314	*N*-Methylpelletierine	8.88	155.1310	P
315	*trans*-9, *trans*-11-octadecadienoic acid; C18:2n-7,9	8.88	280.2404	P
316	MG(0:0/20:1(11*Z*)/0:0)	8.921	384.3241	P
317	20,21,21-Trifluoro-3-methoxy-19-nor-17*alpha*-pregna-1,3,5(10),20-tetraen-17-ol	8.942	366.1809	P
318	3-Cyclohexyldodecane	9.008	252.2817	P
319	(*E,E*)-1,6-bis(4-methoxyphenyl)-1,5-hexadiene	9.036	294.1618	P
320	Isoacitretin	9.036	326.1884	P
321	Annoglabasin F	9.076	378.2406	P
322	Glutamyl-Histidine	9.105	284.1108	P
323	Anofinic acid	9.109	204.0790	P
324	*Alpha*-CEHC	9.112	278.1520	P
325	22-Oxo-docosanoate	9.131	354.3134	P
326	MG(0:0/22:2(13*Z*,16*Z*)/0:0)	9.164	410.3397	P
327	Anopterine	9.176	541.3041	P
328	(6*beta*,7*alpha*,12*beta*,13*beta*)-7-Hydroxy-11,16-dioxo-8,14-apianadien-22,6-olide	9.184	384.1940	P
329	18-Oxocortisol	9.203	376.1883	P
330	Tsangane L 3-glucoside	9.207	374.2307	P
331	Misoprostol	9.208	382.2704	P
332	Neogrifolin	9.254	328.2402	P
333	Gentamicin	9.265	477.3147	P
334	10-Eicosene	9.325	280.3132	P
335	Chloropyramine	9.367	289.1359	P
336	Bioresmethrin	9.369	338.1886	P
337	Acidissiminol epoxide	9.39	409.2254	P
338	MG(0:0/22:6(4*Z*,7*Z*,10*Z*,13*Z*,16*Z*,19*Z*)/0:0)	9.424	402.2758	P
339	Asebotoxin II	9.427	408.2507	P
340	(3b,6b,8b,12a)-8,12-Epoxy-7(11)-eremophilene-6-angeloyloxy-8,12-dimethoxy-3-ol	9.428	394.2361	P
341	6-Hydroxy-8-docosanone	9.428	340.3343	P
342	Methandriol dipropionate	9.429	416.2914	P

343	[6]-Gingerdiol 3,5-diacetate	9.431	380.2202	P
344	Calendulaglycoside E	9.432	794.4335	P
345	(*E*)-3-(2-Hydroxyphenyl)-2-propenal	9.433	148.0525	P
346	Lilac alcohol	9.433	170.1308	P
347	Iriomoteolide 1a	9.447	506.3220	P
348	3-(5,6,6-Trimethylbicyclo[2.2.1]hept-1-yl)cyclohexanol	9.455	236.2141	P
349	MG(0:0/18:3(6*Z*,9*Z*,12*Z*)/0:0)	9.458	352.2618	P
350	Thromboxane	9.482	296.3082	P
351	Trimethaphan	9.49	365.1702	P
352	MG(0:0/14:0/0:0)	9.492	302.2456	P
353	Galbanic acid	9.494	398.2094	P
354	PE(P-18:1(11*Z*)/14:1(9*Z*))	9.547	671.4895	P
355	Heliosupine	9.574	397.2116	P
356	Sorbitan oleate	9.619	428.3117	P
357	4*beta*-(2-Aminoethylthio)catechin	9.642	365.0926	P
358	2-(4-Chloro-3,5-dimethylphenoxy)-N-(2-phenyl-2H-benzotriazol-5-yl)-acetamide	9.644	406.1198	P
359	Monocrotaline	9.644	325.1536	P
360	HDOPA	9.674	376.2259	P
361	Palmitoyl glucuronide	9.733	418.2929	P
362	Ethyl abietate	9.745	330.2558	P
363	Oleoyl Ethanolamide	9.784	325.2980	P
364	4-Hydroxyvalsartan	9.787	451.2219	P
365	Palmitoyl-EA	9.795	299.2822	P
366	Tecostanine	9.796	183.1624	P
367	6,8a-Seco-6,8a-deoxy-5-oxoavermectin "2a"aglycone	9.822	586.3502	P
368	MG(0:0/22:1(13*Z*)/0:0)	9.833	412.3554	P
369	Acetyl tributyl citrate	9.928	402.2255	P
370	1b,3a,7a,12a-Tetrahydroxy-5bcholanoic acid	9.929	424.2809	P
371	Cymorcin monoglucoside	9.929	328.1522	P

372	Sufentanil	9.93	386.2024	P
373	4-Carboxy-2-hydroxy-6-methoxy-6-oxohexa-2,4-dienoate	9.939	216.0271	P
374	Kamahine C	9.939	268.1312	P
375	Diflorasone	9.941	410.1891	P
376	Vanillactic acid	9.941	212.0687	P
377	Arbutin	9.943	272.0898	P
378	Ampalex	9.945	241.1205	P
379	1-(3-Hydroxy-4-methoxyphenyl)-1,2-ethanediol	9.948	184.0736	P
380	Asteltoxin	9.949	418.2001	P
381	1,6-Dihydroxy-3,7-dimethoxy-2-(3-methyl-2-butenyl)-8-(3-hydroxy-3-methyl-1E-butenyl)-xanthone	10.027	440.1830	P
382	Palmitic amide	10.107	255.2564	P
383	Docosatrienoic Acid	10.125	334.2876	P
384	PE(22:6(4*Z*,7*Z*,10*Z*,13*Z*,16*Z*,19*Z*)/24:1(15*Z*))	10.143	873.6204	P
385	Acrovestone	10.147	554.2881	P
386	PC(P-16:0/22:6(4*Z*,7*Z*,10*Z*,13*Z*,16*Z*,19*Z*))	10.153	790.5780	P
387	Hovenidulcioside B2	10.173	708.4081	P
388	Balofloxacin	10.184	389.1756	P
389	Hellebrin	10.19	724.3297	P
390	DU 122290	10.194	362.1653	P
391	Isopimara-7,15-dienol	10.201	288.2454	P
392	Physagulin A	10.215	510.2624	P
393	Mycinamicin III	10.229	681.4089	P
394	(1*alpha*,3*beta*,20*S*,22*R*,24*S*,25*S*)-Pubescenin	10.289	620.3560	P
395	Oleandrin	10.352	576.3296	P
396	Drotaverine	10.38	397.2255	P
397	Panamine	10.388	315.2660	P
398	Ganoderic acid I	10.419	532.3035	P
399	(±)-(*Z*)-2-(5-Tetradecenyl)cyclobutanone	10.478	264.2453	P
400	DG(15:0/20:1(11*Z*)/0:0)	10.482	608.5362	P

401	Oleamide	10.482	281.2719	P
402	1,2-Epoxypropane	10.488	58.0417	P
403	Dodecylbenzene	10.49	246.2348	P
404	Perulactone B	10.49	488.2774	P
405	Lucidumol A	10.503	472.3553	P
406	DG(18:1(11*Z*)/22:5(4*Z*,7*Z*,10*Z*,13*Z*,16*Z*)/0:0)	10.54	668.5407	P
407	DG(20:3(5*Z*,8*Z*,11*Z*)/22:6(4*Z*,7*Z*,10*Z*,13*Z*,16*Z*,19*Z*)/0:0)	10.553	690.5222	P
408	MG(0:0/16:0/0:0)	10.553	330.2771	P
409	Capsi-amide	10.583	269.2720	P
410	4-Nerolidylcatechol	10.595	314.2247	P
411	Dodemorph	10.595	281.2720	P
412	Drospirenone	10.681	366.2197	P
413	D-Glucosyldihydrosphingosine	10.806	463.3506	P
414	Sorbitan palmitate	10.831	402.2978	P
415	2-Pentadecylfuran	10.834	278.2608	P
416	Enalkiren	10.844	656.4287	P
417	Erythromycin	10.861	733.4629	P
418	b-Hydroxypropionyl-CoA	10.896	839.1324	P
419	Butroxydim	10.896	399.2406	P
420	Cavipetin D	10.897	418.2718	P
421	D-myo-Inositol-1,4,5-triphosphate	10.898	419.9630	P
422	(*N*-Acetylglucosaminyl)2-diphosphodolichol	10.944	1675.1189	P
423	13,14-dihydro-15-keto-PGF2α	11.079	354.2402	P
424	Ganodermic acid TQ	11.091	510.3343	P
425	Riesling acetal	11.155	226.1571	P
426	*N*-Hexadecanoylpyrrolidine	11.189	309.3034	P
427	Camptothecin	11.234	348.1113	P
428	Withanolide B	11.348	454.2697	P
429	*Beta*-Elemonic acid	11.372	454.3449	P

430	PE(15:0/22:5(4*Z*,7*Z*,10*Z*,13*Z*,16*Z*))	11.372	751.5185	P
431	2,3-Secoporrigenin	11.472	460.2833	P
432	3'-*N*-Acetyl-4'-*O*-(10,12-octadecadienoyl)fusarochromanone	11.475	596.3824	P
433	Coriandrone E	11.489	248.0685	P
434	DG(20:5(5*Z*,8*Z*,11*Z*,14*Z*,17*Z*)/24:1(15*Z*)/0:0)	11.502	724.6031	P
435	MG(18:0/0:0/0:0)	11.508	358.3084	P
436	Tridemorph	11.539	297.3032	P
437	Hydrocortisone cypionate	11.545	486.2982	P
438	PE(16:1(9*Z*)/22:5(4*Z*,7*Z*,10*Z*,*13Z*,16*Z*))	11.709	763.5137	P
439	Phenolic phosphate	11.765	174.0082	P
440	PG(16:1(9*Z*)/20:3(5*Z*,8*Z*,11*Z*))	11.78	770.5091	P
441	Ganoderic acid *beta*	11.82	500.3138	P
442	Cepagenin	11.957	446.3034	P
443	(*S*)-Rutaretin	11.961	262.0841	P
444	Udenafil	11.967	516.2515	P
445	Phytal	12.074	294.2921	P
446	PE(16:1(9*Z*)/22:6(4*Z*,7*Z*,10*Z*,13*Z*,16*Z*,19*Z*))	12.151	761.4987	P
447	PC(14:0/22:5(4*Z*,7*Z*,10*Z*,13*Z*,16*Z*))	12.257	780.5546	P
448	12-Ketodeoxycholic acid	12.258	390.2773	P
449	PC(16:0/18:1(9*Z*))[*S*]	12.27	760.5854	P
450	Dioctyl hexanedioate	12.281	370.3087	P
451	Luffariellolide	12.281	386.2824	P
452	Oligomycin A	12.281	790.5198	P
453	PC(14:0/20:0)	12.282	762.5995	P
454	Spinosin C	12.283	754.2100	P
455	Strobilurin A	12.358	258.1255	P
456	Lasonolide A	12.602	696.4238	P
457	Hericenone C	12.621	570.3924	P
458	Parishin C	12.623	728.2144	P

459	2-Aminoethylphosphocholate	12.937	515.3037	P
460	DG(14:0/20:1(11*Z*)/0:0)	13.077	594.5220	P
461	Octylamine	13.262	129.1519	P
462	DG(14:1(9*Z*)/18:4(6*Z*,9*Z*,12*Z*,15*Z*)/0:0)	13.726	558.4301	P
463	Streptothricin F acid	13.732	520.2596	P
464	(3*beta*,5*alpha*,6*beta*,7*alpha*,22*E*,24*R*)-Ergosta-8,22-diene-3,5,6,7-tetrol	13.785	446.3390	P
465	3-*O*-*alpha*-LArabinopyranosylcinnamtannin B1	13.919	996.2276	P
466	11*Z*-tetradecenoyl-CoA	13.969	975.2994	P
467	DG(14:0/22:1(13*Z*)/0:0)	14.145	622.5537	P
468	7-Dehydrocholesterol	14.217	384.3405	P
469	DG(14:1(9*Z*)/24:1(15*Z*)/0:0)	14.29	648.5686	P
470	Epifisetinidol-(4*beta*->8)-epicatechin-(6->4*beta*)-epifisetinidol	14.906	834.2141	P
471	Tridodecylamine	15.646	521.5901	P

[a] – retention time [min]

[b] –compound detection in positive (P) or in negative (N) ionization mode.
