# Supplementary material for: Biological Properties of the Mucus and Eggs of Helix aspersa Müller as a Potential Cosmetic and Pharmaceutical Raw Material: A Preliminary Study
Source: Int J Mol Sci. 2024 Sep 15;25(18):9958. doi: 10.3390/ijms25189958 (PMC11432642; doi:10.3390/ijms25189958)
Supplement: Supplementary file 1 [file ijms-25-09958-s001.zip › Herman Anna - Table S3.pdf]

Table S3. Compounds identified in water-methanol[*] extract of fresh egg of organic *Helix aspersa* snail using LC-MS.

No	Metabolite	RT[a] [min]	Mass [*m/z*]	Detection mode [b]
1	2- Benzothiazolesulfonamide	0.322	213.9866	N
2	1,4-Dihydroxy-2- methylanthraquinone	4.718	254.0581	N
3	D-Ribose 1-diphosphate	5.619	293.9901	N
4	Cinnzeylanol	5.627	384.2147	N
5	Blumenol C glucoside	5.727	372.2143	N
6	Ethiprole	5.805	395.9833	N
7	Zingerone	6.227	194.0946	N
8	Eremopetasinorol	6.771	208.1466	N
9	Nordihydrocapsiate	6.831	294.1831	N
10	3-Hydroxy-6,8- dimethoxy-7(11)- eremophilen-12,8-olide	7.033	310.1780	N
11	BILA 2185BS	7.040	618.3251	N
12	4-Hydroxy-5- phenyltetrahydro-1,3- oxazin-2-one	7.057	193.0739	N
13	(*S,Z*)-Lyratol acetate	7.113	194.1308	N
14	(3b,6b,8b,12a)-8,12- Epoxy-7(11)- eremophilene-6- angeloyloxy-8,12- dimethoxy-3-ol	7.198	394.2354	N
15	Histidinyl-Isoleucine	7.281	268.1522	N
16	Zanthodioline	7.282	305.1271	N
17	Lauryl hydrogen sulfate	7.282	266.1552	N
18	Losartan	7.314	422.1625	N
19	Methotrexate	7.315	454.1733	N
20	L-Tyrosine methyl ester	7.342	195.0896	N
21	*N*-Undecylbenzenesulfonic acid	7.733	312.1760	N
22	2- Dodecylbenzenesulfonic acid	8.165	326.1916	N
23	Sodium Tetradecyl Sulfate	8.204	294.1864	N
24	Dinoterb	8.250	240.0748	N
25	(+)-Prosopinine	8.272	313.2618	N
26	Kukoamine D	8.408	530.3118	N

27	4-Methylphenyl octanoate	8.510	234.1621	N
28	(10*beta*H,11xi)-11- Hydroxy-13-nor-6- eremophilen-8-one	8.523	222.1621	N
29	Alcaftadine	8.888	307.1685	N
30	Gemfibrozil	8.960	250.1570	N
31	Furmecyclox	9.283	251.1522	N
32	3-Oxochola-4,6-dien-24-oic Acid	10.249	370.2507	N
33	(5b,7a,12a)-2-(3- methoxyphenyl)-2- oxoethyl ester-7,12- dihydroxy-cholan-24-oic acid	10.294	540.3441	N
34	Oleamide	10.487	281.2719	N
35	Methyl tetradecanoate	10.504	242.2245	N
36	5-Dodecyldihydro-2(3H)- furanone	10.505	254.2247	N
37	Piritramide	10.545	430.2740	N
38	(3*R*,2'*S*)-Myxol 2'-(2,4-di*O*-methyl-α-L-fucoside	10.549	758.5094	N
39	DG(18:1(11*Z*)/22:5(4*Z*,7*Z*,10*Z*,13*Z*,16*Z*)/0:0)	10.549	668.5401	N
40	DG(20:3(5*Z*,8*Z*,11*Z*)/22: 6(4*Z*,7*Z*,10*Z*,13*Z*,16*Z*,19*Z*)/0:0)	10.550	690.5220	N
41	MG(0:0/16:0/0:0)	10.550	330.2770	N
42	Schidigeragenin B	10.550	428.2946	N
43	Enalkiren	10.844	656.4281	N
44	Butroxydim	11.270	399.2409	N
45	Adlupone	11.370	482.3395	N
46	MG(18:0/0:0/0:0)	11.505	358.3084	N
47	DG(20:5(5*Z*,8*Z*,11*Z*,14*Z*,17*Z*)/24:1(15*Z*)/0:0)	11.505	724.6027	N
48	Pubesenolide	11.507	458.3051	N
49	Callystatin A	11.508	456.3259	N
50	(22*E*,24*R*)-Stigmasta4,22-diene-3,6-dio	11.962	424.3340	N
51	24-Acetyl- 25- cinnamoylvulgaroside	12.066	608.3350	N
52	6,8a-Seco-6,8a-deoxy-5- oxoavermectin "2a" aglycone	12.066	586.3529	N
53	(3*beta*,22*E*,24*R*)-3- Hydroxyergosta-5,8,22- trien-7-one	12.503	410.3185	N
54	PI(18:0/22:4(10*Z*,13*Z*,16*Z*,19*Z*))	12.784	914.5892	N
1	5-Heptyltetrahydro-2-oxo-3-furancarboxylic acid	3.136	228.1361	P

2	Sedanonic acid	3.227	210.1253	P
3	*N*-n-Hexanoylglycine methyl ester	3.330	187.1209	P
4	Netilmicin	3.457	475.2994	P
5	Homoarecoline	3.464	169.1103	P
6	Istamycin C1	3.592	431.2733	P
7	Phlorin	3.703	288.0846	P
8	5-Nonyltetrahydro-2-oxo-3-furancarboxylic acid	3.838	256.1676	P
9	(3*R*,7*R*)-1,3,7-Octanetriol	4.069	162.1257	P
10	Jasmine ketolactone	4.248	208.1098	P
11	*N*-Methylmescaline	4.248	225.1363	P
12	Oseltamivir	4.378	312.2051	P
13	Triethylenemelamine	4.392	204.1131	P
14	Amyl 2-furoate	4.397	182.0944	P
15	PE(18:4(6*Z*,9*Z*,12*Z*,15*Z*)/22:6(4*Z*,7*Z*,10*Z*,13*Z*,16*Z*,19*Z*))	4.449	783.4833	P
16	1-Octen-3-yl glucoside	4.472	290.1730	P
17	Halstoctacosanolide A	4.525	844.5358	P
18	(*E*)-3-decen-1-ol	4.541	156.1513	P
19	Diethofencarb	4.546	267.1471	P
20	Flumetover	4.546	367.1394	P
21	Ethyl 3-(*N*butylacetamido)propionate	4.647	215.1523	P
22	1,2,3-Tris(1-ethoxyethoxy)propane	4.662	308.2199	P
23	2-Hexenoylcholine	4.667	200.1652	P
24	C12:1n-7	4.679	198.1623	P
25	*Gamma*-CEHC	4.680	248.1412	P
26	Humulinic acid A	4.681	266.1517	P
27	Ruscopine	4.690	306.2046	P
28	(5*R*)-5-Hydroxyhexanoic acid	4.726	132.0786	P
29	2-Ethylacrylylcarnitine	4.726	244.1552	P
30	2-Phenylbutyric acid	4.726	164.0838	P

31	3-Indolecarboxylic acid	4.726	253.1309	P
32	1-Phenyl-6,7-dihydroxyisochroman	4.727	242.0946	P
33	2,3-dihydrobenzofuran	4.727	120.0575	P
34	*Beta*-hydroxylauric acid	4.728	216.1726	P
35	Alanyl-Isoleucine	4.776	202.1317	P
36	Methyl 7-epi-12-hydroxyjasmonate glucoside	4.789	402.1889	P
37	(*E*)-3-(2-Hydroxyphenyl)-2-propenal	4.795	148.0524	P
38	*N*-Isobutyl-2,4,8,10,12-tetradecapentaenamide	4.797	273.2095	P
39	Methyl 3-(2,3-dihydroxy-3-methylbutyl)-4-hydroxybenzoate	4.799	254.1156	P
40	1,3-Diphenyltetramethyldisiloxane	4.958	286.1208	P
41	Methyl propionate	4.958	88.0523	P
42	2-Phenylethyl *beta*-Dglucopyranoside	5.008	284.1265	P
43	1,1,2-Triphenylpropane	5.014	272.1559	P
44	5,7-Megastigmadien-9-ol glucoside	5.036	356.2194	P
45	Cinncassiol A 19-glucoside	5.048	544.2514	P
46	Sterebin E	5.073	338.2458	P
47	Z-Arg-Arg-NHMec	5.078	621.3053	P
48	(*S*)-3-Octanol glucoside	5.092	292.1885	P
49	Decylubiquinol	5.098	324.2302	P
50	7,8-Dihydrovomifoliol 9-[rhamnosyl-(1->6)-glucoside]	5.105	534.2678	P
51	(-)-*trans*-Carveol glucoside	5.133	314.1732	P
52	Gibberellin A105	5.133	330.1466	P
53	Glycerol 1-(5-hydroxydodecanoate)	5.229	290.2093	P
54	Toxin T2 tetrol	5.241	298.1419	P
55	Dihydro-5-(2-octenyl)-2(3H)-furanone	5.267	196.1466	P
56	Cyclonormammein	5.272	374.1727	P
57	Elaeokanine C	5.286	211.1573	P
58	Artabsinolide A	5.306	280.1310	P
59	Jasmolone glucoside	5.369	342.1681	P

60	NAc-FnorLRF-amide	5.371	622.3565	P
61	Ethyl 7-epi-12-hydroxyjasmonate glucoside	5.408	416.2047	P
62	AF Toxin II	5.430	324.1575	P
63	Taraxacolide 1-*O*-b-Dglucopyranoside	5.440	428.2044	P
64	Hydrocortisone succinate	5.480	462.2254	P
65	Corchoionol C 9-glucoside	5.481	386.1943	P
66	*O*-Methylsomniferine	5.499	622.2652	P
67	(*E,E,E*)-*N*-(2-Methylpropyl)hexadeca-2,6,8-trien-10-ynamide	5.502	301.2406	P
68	Epothilone C	5.504	477.2571	P
69	11-Hydroxy-9-tridecenoic acid	5.512	228.1726	P
70	8-Butanoylneosolaniol	5.577	452.2025	P
71	Eremopetasinorol	5.654	208.1465	P
72	*N*-Jasmonoylisoleucine	5.682	323.2099	P
73	Blumenol C glucoside	5.685	372.2149	P
74	(2xi,6xi)-7-Methyl-3-methylene-1,2,6,7-octanetetrol	5.699	204.1359	P
75	Hexanal octane-1,3-diol acetal	5.702	228.2090	P
76	2-Methylundecanal	5.723	184.1828	P
77	(5*alpha*,10*alpha*)-3,7(11)-Eudesmadien-2-one	5.765	218.1671	P
78	Avocadienofuran	5.765	246.1983	P
79	2-Furanmethanol	5.768	98.0367	P
80	Volicitin	5.768	422.2764	P
81	Goshonoside F3	5.770	644.3381	P
82	Blumenol C *O*-[rhamnosyl-(1->6)-glucoside]	5.785	518.2728	P
83	Fluspirilene	5.803	475.2417	P
84	2-Hydroxymyristic Acid	5.829	244.2038	P
85	Acetyllycopsamine	5.829	341.1843	P
86	19(*R*)-hydroxy-PGE2	5.832	368.2199	P
87	C14:1n-9	5.871	226.1933	P
88	Eriojaposide A	5.877	502.2414	P

89	Norerythrostachaldine	5.939	407.2655	P
90	Canavalioside	5.941	546.2676	P
91	(+/-)-*N,N*-Dimethyl menthyl succinamide	6.012	168.1881	P
92	Capsoside A	6.016	694.3772	P
93	15-Acetoxyscirpene-3,4-diol 4-*O*-a-Dglucopyranoside	6.023	486.2102	P
94	Capsaicin	6.058	305.1995	P
95	Lauroyl diethanolamide	6.072	287.2464	P
96	20-COOH-Leukotriene B4	6.140	366.2039	P
97	2-Hydroxyestrone	6.143	286.1570	P
98	Europine	6.145	329.1833	P
99	(*Z*)-6-Nonenal	6.146	140.1202	P
100	Penbutolol	6.176	291.2199	P
101	Ciclesonide	6.194	540.3098	P
102	Pseudoargiopinin III	6.201	373.2100	P
103	4-Hydroxy-3-methoxy-2,10-bisaboladien-9-one	6.208	266.1869	P
104	(+)-Prosopinine	6.228	313.2620	P
105	Cincassiol B	6.255	400.2100	P
106	*Alpha*-Butyl-*omega*hydroxypoly(oxyethylene) poly(oxypropylene)	6.266	248.1990	P
107	3'-Hydroxy-HT2 toxin	6.289	440.2048	P
108	Gravelliferone	6.304	298.1571	P
109	Eucalyptol	6.333	154.1359	P
110	1,1-Diethoxy-2-hexene	6.351	172.1464	P
111	Cuscohygrine	6.370	224.1889	P
112	Dihydrocapsaicin	6.383	307.2146	P
113	9-HOTE	6.486	294.2195	P
114	Lithocholic acid sulfate	6.585	456.2555	P
115	10-Hydroxy-2,8-decadiene-4,6-diynoic acid	6.662	176.0474	P
116	Monoisobutyl phthalic acid	6.662	222.0894	P
117	C16 Sphinganine	6.679	273.2670	P

118	Momilactone B	6.681	330.1832	P
119	*p*-Hydroxybenzyldesulphoglucosinolate	6.691	345.0870	P
120	17-Methylandrosta-2,4-dieno[2,3-d]isoxazol-17*beta*-ol	6.704	327.2191	P
121	2,4,12-Octadecatrienoic acid isobutylamide	6.708	333.3016	P
122	Glicoisoflavanone	6.711	384.1571	P
123	2-Tetradecanone	6.712	212.2141	P
124	Cabergoline	6.727	451.2954	P
125	1-Isomangostin hydrate	6.732	428.1831	P
126	5-(2,3-Dihydroxy-3-methylbutyl)-4-(3,4-epoxy-4-methylpentanoyl)-3,4-dihydroxy-2-isopentanoyl-2-cyclopenten-1-one	6.732	412.2099	P
127	Cerberoside	6.734	858.4242	P
128	Phytosphingosine	6.754	317.2933	P
129	Deacetylnomilin	6.755	472.2100	P
130	Ximelagatran	6.755	473.2627	P
131	Austalide A	6.776	516.2354	P
132	Mycalamide B	6.778	517.2889	P
133	16-hydroxy hexadecanoic acid	6.784	272.2352	P
134	Canescein	6.801	566.2708	P
135	Funtumine	6.842	317.2721	P
136	2-Pentadecanone	6.852	226.2297	P
137	(S)-Nerolidol 3-*O*-[a-LRhamnopyranosyl-(1->4)-a-Lrhamnopyranosyl-(1->2)-b-Dglucopyranoside]	6.862	676.3673	P
138	5-Dodecyldihydro-2(3H)-furanone	6.877	254.2248	P
139	Austalide F	6.893	490.2208	P
140	Pumiliotoxin 251D	6.893	251.2253	P
141	1-Methyl-2-nonyl-4(1H)-quinolinone	6.894	285.2098	P
142	Genipin 1-betagentiobioside	6.897	550.1905	P
143	1-Tridecene	6.911	182.2035	P
144	Zizybeoside II	6.918	594.2163	P
145	Panaquinquecol 1	6.930	292.2038	P

146	Kanokoside C	6.935	638.2424	P
147	Chrycolide	6.942	232.0189	P
148	Contignasterol	6.943	508.3385	P
149	4,5-Dihydroniveusin A	6.963	396.1785	P
150	Coriandrone D	6.963	352.1523	P
151	Muricatacin	6.986	284.2352	P
152	Nonyl octanoate	6.986	270.2558	P
153	Plantaricin BN	6.986	484.2309	P
154	Coccinin	6.999	528.2568	P
155	6-Caffeoylsucrose	7.018	504.1478	P
156	Acetyl Tyrosine Ethyl Ester	7.026	251.1159	P
157	Palmitic amide	7.027	617.4209	P
158	Discodermolide	7.032	593.3911	P
159	BILA 2185BS	7.041	618.3256	P
160	3*beta*-Hydroxypregn-5-ene	7.049	302.2611	P
161	Cyclotetradecane	7.056	196.2192	P
162	Terbucarb	7.090	277.2042	P
163	Proansamitocin	7.093	443.2288	P
164	6*alpha*,9-Difluoro-11beta-hydroxypregn-4-ene-3,20-dione	7.110	366.2019	P
165	2-Methoxyestradiol-3-methylether	7.112	316.2024	P
166	Finaconitine	7.124	630.3153	P
167	Paucin	7.174	468.1994	P
168	7-Hydroxy-3-(4-methoxyphenyl)-4-methylcoumarin	7.185	282.0893	P
169	Z-Gly-Pro-Leu-Gly-Pro	7.187	573.2783	P
170	10,16-dihydroxy-palmitic acid	7.190	288.2301	P
171	Armillaric acid	7.240	416.1835	P
172	Allopumiliotoxin 267A	7.248	267.2198	P
173	*trans*-9, *trans*-11-octadecadienoic acid; C18:2n-7,9	7.250	280.2405	P
174	Dodecylguanidine	7.259	227.2364	P

175	Bleekerine	7.317	409.1758	P
176	Testolactone	7.337	300.1727	P
177	Ethyl (4*Z*)-4,7-octadienoate	7.356	168.1152	P
178	Dolichyl diphosphate	7.438	520.2694	P
179	Etiocholan-3α-ol-17-one3-glucuronide	7.453	466.2569	P
180	Austalide L	7.458	428.2201	P
181	Picrasin C	7.458	422.2305	P
182	(3'x,5'a,9'x,10'b)-O-(3-Hydroxy-6-oxo-7-drimen-11-yl)umbelliferone	7.459	396.1934	P
183	2,2-Dimethyl-3,4-bis(4-methoxyphenyl)-2H-1-benzopyran-7-ol acetate	7.459	430.1780	P
184	*Alpha*-Methylstyrene	7.459	118.0782	P
185	Armillaripin	7.459	414.2045	P
186	Cyclocalopin F	7.459	294.1100	P
187	Erythroskyrin	7.459	455.2310	P
188	(4-Methylphenyl)acetaldehye	7.461	134.0729	P
189	Methyl (9*Z*)-10'-oxo-6,10'-diapo-6-carotenoate	7.500	312.1728	P
190	Norpropoxyphene	7.509	325.2046	P
191	Octadecanedioic acid	7.513	314.2459	P
192	7,10-Hexadecadienoic acid	7.564	252.2089	P
193	Sphinganine	7.598	301.2982	P
194	2-Hexadecanone	7.638	240.2456	P
195	Spiroxamine	7.663	297.2672	P
196	Biperiden	7.676	311.2247	P
197	3'-N'-Acetylfusarochromanone	7.702	334.1546	P
198	Austalide B	7.718	474.2256	P
199	Mammea E/BB	7.737	430.1991	P
200	1-(4-Amino-2-methylpyrimid-5-ylmethyl)-3-(*beta*hydroxyethyl)-2-methylpyridinium	7.747	259.1550	P
201	2,6-Di-tert-butyl-4-ethylphenol	7.775	234.1983	P
202	Gabapentin	7.779	171.1260	P

203	Methyloctatropine	7.788	282.2435	P
204	Phlegmarine	7.831	250.2408	P
205	Glaucamine	7.837	385.1526	P
206	Sanshodiol	7.837	358.1417	P
207	Methadone	7.873	309.2089	P
208	1-Pentadecene	7.876	210.2352	P
209	Estrane-3α,17α-diol	7.879	278.2248	P
210	Dihydrodioscorine	7.904	223.1572	P
211	Methyl 15-cyanopentadecanoate	7.930	281.2357	P
212	Elaiophylin	7.941	1024.593 4	P
213	(3a,5b)-24-oxo-24-[(2-sulfoethyl)amino]cholan-3-yl-b-Dglucopyranosiduronic acid	7.950	659.3342	P
214	Dodecanamide	7.957	199.1938	P
215	Asparagoside D	7.963	902.4874	P
216	Firocoxib	7.997	336.1028	P
217	Scopoloside II	8.001	770.4090	P
218	Stearamide	8.009	283.2877	P
219	2-Methoxyestrone 3-sulfate	8.010	380.1291	P
220	Leucomycin A9	8.016	743.4095	P
221	Corchorusoside B	8.033	682.3566	P
222	Convallatoxin	8.092	550.2779	P
223	Undecylprodigiosin	8.170	393.2780	P
224	Oleoyl Ethanolamide	8.196	325.2981	P
225	Pipericine	8.241	335.3174	P
226	Lyngbyatoxin	8.273	437.3047	P
227	Pipercitine	8.273	349.3331	P
228	17*beta*-Acetamidoandrost-4-en-3-one	8.274	329.2354	P
229	2-(4-Methylphenyl)-2-propanol	8.274	150.1044	P
230	Santene	8.275	122.1098	P

231	6-Oxocineole	8.276	168.1151	P
232	*p*-Mentha-1,3,5,8-tetraene	8.276	132.0941	P
233	Tributyl phosphate	8.305	266.1648	P
234	Ponasteroside A	8.310	626.3664	P
235	1-Phenyl-1,3-dodecanedione	8.312	274.1936	P
236	Avocadenofuran	8.336	248.2142	P
237	Tranexamic acid	8.341	157.1105	P
238	1-Methyl-1,3-cyclohexadiene	8.342	94.0783	P
239	Isopentylideneisopentylamine	8.344	155.1676	P
240	*cis*-1,2-Dihydro-3-ethylcatechol	8.346	140.0840	P
241	Sorbitan oleate	8.349	428.3117	P
242	Methyl 2-octynoate	8.359	154.0994	P
243	2-Decylfuran	8.369	208.1828	P
244	Mycinamicin VIII	8.392	505.3391	P
245	Kukoamine D	8.406	530.3128	P
246	Triphenyl phosphate	8.408	326.0712	P
247	Methyprylon	8.448	183.1260	P
248	Lentiginosine	8.482	157.1104	P
249	3L,7D,11D-phytanic acid	8.510	312.3028	P
250	Polidocanol	8.518	582.4342	P
251	*N*-(14-Methylhexadecanoyl)pyrrolidine	8.520	323.3189	P
252	Linoleoyl Ethanolamide	8.523	323.2830	P
253	Isopimara-7,15-dienol	8.528	288.2455	P
254	Dodemorph	8.535	281.2720	P
255	8,8-Diethoxy-2,6-dimethyl-2-octanol	8.544	246.2195	P
256	(3a,5b,7a,12a)-24-[(carboxymethyl)amino]-1,12-dihydroxy-24-oxocholan-3-yl-b-DGlucopyranosiduronic a	8.545	641.3408	P
257	Oleyl alcohol	8.558	424.3657	P
258	Vaccenyl carnitine	8.602	425.3511	P

259	Polysorbate 20	8.616	522.3404	P
260	Laserpitin	8.766	450.2620	P
261	Polysorbate 60	8.766	434.2883	P
262	Stearoylethanolamide	8.782	327.3140	P
263	Hexyl heptanoate	8.788	638.2366	P
264	10-Eicosene	8.810	280.3129	P
265	Tecostanine	8.827	183.1623	P
266	2,2,7,7-Tetramethyl-1,6-dioxaspiro[4.4]non-3-ene	8.830	182.1306	P
267	9-Acetoxyfukinanolide	8.862	292.1678	P
268	13-heptadecyn-1-ol	8.879	252.2451	P
269	4-Vinylcyclohexene	8.880	108.0939	P
270	N-Methylpelletierine	8.881	155.1314	P
271	β-Caryophyllene Alcohol	8.916	222.1986	P
272	MG(0:0/20:1(11*Z*)/0:0)	8.920	384.3242	P
273	Formebolone	8.939	344.1989	P
274	3-Cyclohexyldodecane	9.006	252.2818	P
275	Annoglabasin F	9.013	378.2406	P
276	(*E,E*)-1,6-bis(4-methoxyphenyl)-1,5-hexadiene	9.036	294.1620	P
277	Isoacitretin	9.036	326.1882	P
278	*Alpha*-CEHC	9.111	278.1520	P
279	Anofinic acid	9.112	204.0788	P
280	22-Oxo-docosanoate	9.130	354.3136	P
281	MG(0:0/22:2(13*Z*,16*Z*)/0:0)	9.166	410.3398	P
282	Anopterine	9.178	541.3042	P
283	Armillarivin	9.184	384.1938	P
284	(6*beta*,7*alpha*,12*beta*,13*beta*)-7-Hydroxy-11,16- dioxo-8,14-apianadien22,6-olide	9.187	384.1938	P
285	PC(22:6(4Z,7Z,10*Z*,13*Z*,16*Z*,19*Z*)/22:6(4*Z*,7*Z*,10*Z*,13*Z*,16*Z*,19*Z*))	9.203	878.5713	P
286	18-Oxocortisol	9.206	376.1885	P
287	Misoprostol	9.209	382.2703	P

288	Guaioxide	9.237	222.1984	P
289	Gentamicin	9.266	477.3146	P
290	Bioresmethrin	9.368	338.1883	P
291	Chloropyramine	9.369	289.1355	P
292	Acidissiminol epoxide	9.389	409.2252	P
293	MG(0:0/22:6(4*Z*,7*Z*,10*Z*,13*Z*,16*Z*,19*Z*)/0:0)	9.423	402.2755	P
294	(3b,6b,8b,12a)-8,12-Epoxy-7(11)-eremophilene-6-angeloyloxy-8,12-dimethoxy-3-ol	9.426	394.2354	P
295	Methandriol dipropionate	9.427	416.2927	P
296	6-Hydroxy-8-docosanone	9.429	340.3340	P
297	[6]-Gingerdiol 3,5-diacetate	9.431	380.2202	P
298	Ethoxysulfuron	9.432	398.0892	P
299	Phenkapton	9.432	375.9354	P
300	Calendulaglycoside E	9.433	794.4335	P
301	Lilac alcohol	9.433	170.1307	P
302	XE991	9.433	376.1592	P
303	Tsugarioside B	9.440	616.4325	P
304	Iriomoteolide 1a	9.449	506.3221	P
305	Lucidenic acid M	9.455	462.2963	P
306	MG(0:0/18:3(6*Z*,9*Z*,12*Z*)/0:0)	9.457	352.2616	P
307	Thromboxane	9.483	296.3081	P
308	5-*O*-Desmethyldonepezil	9.490	365.1993	P
309	MG(0:0/14:0/0:0)	9.490	302.2458	P
310	Erythrophleguine	9.491	449.2782	P
311	Erinacine E	9.493	432.2517	P
312	Galbanic acid	9.494	398.2094	P
313	3-*O*Sulfogalactosylceramide	9.531	907.6444	P
314	Heliosupine	9.565	397.2117	P
315	Lucidenic acid G	9.584	476.2774	P

316	2-(4-Chloro-3,5-dimethylphenoxy)-*N*-(2-phenyl-2H-benzotriazol-5-yl)-acetamide	9.642	406.1195	P
317	4*beta*-(2-Aminoethylthio)catechin	9.642	365.0925	P
318	Monocrotaline	9.643	325.1524	P
319	HDOPA	9.676	376.2249	P
320	3-(5,6,6-Trimethylbicyclo[2.2.1]hept-1-yl)cyclohexanol	9.699	236.2141	P
321	Palmitoyl glucuronide	9.735	418.2933	P
322	4-Hydroxyvalsartan	9.784	451.2218	P
323	Palmitoyl-EA	9.795	299.2824	P
324	Nitramine	9.814	169.1463	P
325	6,10,14-Trimethyl-5,9,13-pentadecatrien-2-one	9.815	262.2298	P
326	Tricycloekasantal	9.815	178.1358	P
327	2,6-Diisopropyl-3-methylphenol	9.816	192.1518	P
328	*Delta*-Methylionone	9.816	206.1668	P
329	MG(0:0/22:1(13*Z*)/0:0)	9.834	412.3557	P
330	(3*S*,6*E*,10*E*)-1,6,10,14-Phytatetraen-3-ol	9.873	290.2614	P
331	1b,3a,7a,12a-Tetrahydroxy-5b cholanoic acid	9.941	424.2808	P
332	Asteltoxin	9.942	418.1988	P
333	4-Carboxy-2-hydroxy-6-methoxy-6-oxohexa-2,4-dienoate	9.943	216.0269	P
334	Acetyl tributyl citrate	9.943	402.2254	P
335	1-(3-Hydroxy-4-methoxyphenyl)-1,2-ethanediol	9.944	184.0736	P
336	2,5-Furandicarboxylic acid	9.944	156.0059	P
337	Arbutin	9.944	272.0895	P
338	Cymorcin monoglucoside	9.944	328.1522	P
339	Kamahine C	9.944	268.1308	P
340	Vanillactic acid	9.944	212.0684	P
341	Acrovestone	10.146	554.2883	P
342	Hovenidulcioside B2	10.174	708.4098	P
343	Balofloxacin	10.182	389.1759	P

344	19,20-DiHDPA	10.184	362.2460	P
345	Hellebrin	10.188	724.3295	P
346	Allixin	10.190	226.1204	P
347	Physagulin A	10.216	510.2612	P
348	Mycinamicin III	10.230	681.4093	P
349	Oleandrin	10.350	576.3301	P
350	Drotaverine	10.378	397.2256	P
351	Drospirenone	10.390	366.2197	P
352	Ganoderic acid I	10.419	532.3037	P
353	Cinitapride	10.422	402.2256	P
354	DU 122290	10.441	362.1648	P
355	Bis(3-azidopyridinium)-1,10-decane perchlorate	10.448	380.2436	P
356	Dodecylbenzene	10.488	246.2348	P
357	(±)-(*Z*)-2-(5-Tetradecenyl)cyclobutanone	10.489	264.2454	P
358	Perulactone B	10.489	488.2782	P
359	Oleamide	10.490	281.2722	P
360	DG(15:0/20:1(11*Z*)/0:0)	10.491	608.5356	P
361	PE(22:0/24:0)	10.491	887.7347	P
362	DG(20:3(5*Z*,8*Z*,11*Z*)/22:6(4*Z*,7*Z*,10*Z*,13*Z*,16*Z*,19*Z*)/0:0)	10.551	690.5225	P
363	DG(18:1(11*Z*)/22:5(4*Z*,7*Z*,10*Z*,13*Z*,16*Z*)/0:0)	10.552	668.5406	P
364	MG(0:0/16:0/0:0)	10.552	330.2771	P
365	4-Nerolidylcatechol	10.594	314.2252	P
366	4-(3-Methyl-1-butenyl)-3,3',4',5-tetrahydroxystilbene	10.648	312.1358	P
367	Capsi-amide	10.730	269.2720	P
368	13*Z*,16*Z*-docosadienoic acid	10.738	336.3027	P
369	*D*-Glucosyldihydrosphingosine	10.807	463.3509	P
370	2-Pentadecylfuran	10.835	278.2612	P
371	Enalkiren	10.844	656.4292	P
372	Butroxydim	10.894	399.2398	P

373	Cavipetin D	10.895	418.2720	P
374	Gluconapoleiferin	10.895	403.0617	P
375	Sorbitan palmitate	10.895	402.2981	P
376	*D*-myo-Inositol-1,4,5-triphosphate	10.896	419.9642	P
377	Diprenorphine	10.912	425.2572	P
378	Petasitenine	11.072	381.1769	P
379	(ent-2b,4S,9a)-2,4,9-Trihydroxy-10(14)-oplopen-3-one 2-(2-methylbutanoate) 9-(3-methyl-2E-pentenoate)	11.131	448.2829	P
380	Linalyl propionate	11.154	210.1617	P
381	Riesling acetal	11.154	226.1569	P
382	*N*-Hexadecanoylpyrrolidine	11.189	309.3031	P
383	Camptothecin	11.238	348.1099	P
384	Withanolide B	11.349	454.2697	P
385	Dodecaprenyl diphosphate	11.416	994.6973	P
386	3'-*N*-Acetyl-4'-*O*-(10,12-octadecadienoyl)fusarochromanone	11.478	596.3826	P
387	5-Hexyltetrahydro-2-furanoctanoic acid	11.484	298.2510	P
388	MG(18:0/0:0/0:0)	11.508	358.3085	P
389	DG(20:5(5*Z*,8*Z*,11*Z*,14*Z*,17*Z*)/24:1(15*Z*)/0:0)	11.509	724.6029	P
390	1,2-Epoxypropane	11.510	58.0417	P
391	Tridemorph	11.533	297.3033	P
392	Phenethyl decanoate	11.536	276.2090	P
393	Hydrocortisone cypionate	11.545	486.2984	P
394	Sorbitan stearate	11.757	430.3301	P
395	Ganoderic acid beta	11.888	500.3141	P
396	Prorocentrolide	11.888	979.5992	P
397	Cepagenin	11.955	574.3272	P
398	(*S*)-Rutaretin	11.964	262.0843	P
399	Phytal	12.076	294.2912	P
400	12-Ketodeoxycholic acid	12.260	390.2773	P

401	PC(16:0/18:1(9*Z*))[*S*]	12.268	760.5854	P
402	Dioctyl hexanedioate	12.280	370.3084	P
403	Luffariellolide	12.282	386.2817	P
404	Strobilurin A	12.360	258.1257	P
405	Lasonolide A	12.602	696.4236	P
406	Parishin C	12.623	728.2142	P
407	Hericenone C	12.624	570.3923	P
408	Ganoderic acid V	12.711	528.3474	P
409	DG(14:0/20:1(11*Z*)/0:0)	13.080	594.5219	P
410	Didodecyl thiobispropanoate	13.175	514.4062	P
411	DG(14:1(9*Z*)/18:4(6*Z*,9*Z*,12*Z*,15*Z*)/0:0)	13.700	558.4295	P
412	Dioncophyllinol B	13.722	379.1784	P
413	3*beta*,5*alpha*,6*beta*,7*alpha*,22*E*,24*R*)-Ergosta-8,22-diene-3,5,6,7-tetrol	13.797	446.3395	P
414	Elastin	13.836	552.3614	P
415	5,6-Dihydro-5,6-dihydroxy-y,y-carotene	13.845	572.4586	P
416	DG(14:0/22:1(13*Z*)/0:0)	14.149	622.5537	P
417	4a-Methyl-5a-cholesta-8,24-dien-3-one	14.186	396.3389	P
418	7-Dehydrocholesterol	14.213	384.3396	P
419	DG(14:1(9*Z*)/24:1(15*Z*)/0:0)	14.325	648.5691	P
420	Tridodecylamine	15.684	521.5901	P
421	DG(14:0/24:1(15*Z*)/0:0)	15.849	650.5849	P

*- methanol: water (1:1, *v/v*)

[a] – retention time [min]

[b] –compound detection in positive (P) or in negative (N) ionization mode.
