# Supplementary material for: Biological Properties of the Mucus and Eggs of Helix aspersa Müller as a Potential Cosmetic and Pharmaceutical Raw Material: A Preliminary Study
Source: Int J Mol Sci. 2024 Sep 15;25(18):9958. doi: 10.3390/ijms25189958 (PMC11432642; doi:10.3390/ijms25189958)
Supplement: Supplementary file 1 [file ijms-25-09958-s001.zip › Herman Anna - Table S4.pdf]

Table S4. Compounds identified in acetonitrile extract of fresh egg of organic *Helix aspersa* snail using LC-MS.

No	Metabolite	RT[a] [min]	Mass [*m/z*]	Detection mode[b]
1	Trifluoroacetic acid	0.255	113.9928	N
2	Methyl *N*-(amethylbutyryl)glycine	4.301	188.1050	N
3	D-Ribose 1-diphosphate	5.622	293.9902	N
4	Ethiprole	5.806	395.9833	N
5	Flupropanate	6.065	145.9992	N
6	Zingerone	6.230	194.0944	N
7	Athidathion	6.331	329.9917	N
8	Bismuth subsalicylate	6.709	361.9976	N
9	Eremopetasinoro	6.775	208.1463	N
10	Nordihydrocapsiate	6.833	294.1831	N
11	3-Hydroxy-6,8- dimethoxy-7(11)- eremophilen-12,8-olide	7.032	310.1781	N
12	3bAllotetrahydrocorticosterone	7.119	350.2456	N
13	Losartan	7.313	422.1621	N
14	Methotrexate	7.314	454.1734	N
15	L-Tyrosine methyl ester	7.341	195.0897	N
16	Dinoterb	7.578	240.0748	N
17	*N*-Undecylbenzenesulfonic acid	7.728	312.1760	N
18	2- Dodecylbenzenesulfonic acid	8.161	326.1915	N
19	Sodium Tetradecyl Sulfate	8.205	294.1865	N
20	Furmecyclox	9.280	251.1523	N
21	(5b,7a,12a)-2-(3-methoxyphenyl)-2- oxoethyl ester-7,12- dihydroxy-cholan-24-oic acid	10.295	540.3447	N
22	Enalkiren	10.844	656.4287	N
23	Adlupone	11.362	482.3396	N
1	2-Amino-2-methyl-1,3-propanediol	0.255	105.0790	P
2	Dulcitol	0.255	182.0792	P
3	Ethyl propionate	0.260	102.0680	P

4	Choline chloride	0.261	103.0996	P
5	Pandamarilactam 3x	0.261	235.1210	P
6	L-Alloisoleucine	0.266	131.0946	P
7	Asulam	0.268	230.0365	P
8	Isoamyl nitrite	0.270	117.0790	P
9	Penicillin O	0.270	330.0698	P
10	19-Hydroxy-8-*O*-methyltetrangulol	0.271	334.0837	P
11	Benzo[b]naphtho[2,1- d]thiophene	0.271	234.0511	P
12	(*S*)-Rutaretin	0.272	262.0840	P
13	4',7-Di-*O*-methylcatechin	0.275	318.1104	P
14	4-Amino-2-methylenebutanoic acid	0.275	115.0633	P
15	Niazirin	0.275	279.1105	P
16	Angoline	0.276	379.1439	P
17	Arginyl-Hydroxyproline	0.276	287.1587	P
18	Proline betaine	0.280	144.0986	P
19	Triethylamine	0.281	101.1204	P
20	Bufexamac	0.282	223.1209	P
21	Nicotinamide *N*-oxide	0.284	138.0431	P
22	2-(Methylthio)-3H- phenoxazin-3-one	0.365	243.0360	P
23	Trolamine	0.406	149.1053	P
24	*R*-2-Hydroxy-3-methylbutanoic acid 3-Methylbutanoyl	0.656	202.1206	P
25	3-Hydroxysuberic acid	0.726	190.0840	P
26	Dexpanthenol	0.857	205.1315	P
27	2-Amino-4-hydroxy-6-(hydroxymethyl)-7,8-dihydropteridine	0.868	195.0761	P
28	Polypropylene glycol (mw 1,200-3,000)	0.998	134.0943	P
29	5-(2-Methylpropyl)tetrahydro-2-oxo-3-furancarboxylic acid	1.197	186.0894	P
30	2,5-Dihydro-2,4,5-trimethyloxazole	1.507	113.0841	P
31	Amyl 2-furoate	2.062	182.0943	P
32	2*E*-Decenedioic acid	2.345	200.1049	P

33	Artemidinol	2.349	216.0788	P
34	Retronecine	2.398	155.0947	P
35	*N*-Valerylglycine methyl ester	2.399	173.1052	P
36	Propionyl-L-carnitine	2.507	218.1393	P
37	2,6-Dimethoxy-4-methylphenol	2.512	168.0786	P
38	DL-2-amino-octanoic acid	2.582	159.1259	P
39	5-Heptyltetrahydro-2- oxo-3-furancarboxylic acid	2.961	228.1362	P
40	Sedanonic acid	2.963	210.1252	P
41	5-(3*E*Pentenyl)tetrahydro-2-oxo-3-furancarboxylic acid	3.016	198.0892	P
42	Geranyl acetoacetate	3.037	238.1570	P
43	3-hydroxy- tetradecanedioic acid	3.051	274.1780	P
44	2-Isopropyl-1,4- benzenediol	3.227	152.0837	P
45	(1'*R*)-Nepetalic acid	3.301	184.1099	P
46	Wine lactone	3.302	166.0994	P
47	*γ*-Aminobutyryl-lysine	3.468	231.1584	P
48	*N*-n-Hexanoylglycine methyl ester	3.470	187.1210	P
49	Homoarecoline	3.470	169.1103	P
50	2,3-Dimethyl-2- cyclohexen-1-one	3.471	124.0890	P
51	Varenicline	3.509	211.1110	P
52	Istamycin C1	3.588	431.2730	P
53	2,3-Dihydro-5-(5-methyl- 2-furanyl)-1H-pyrrolizine	3.620	187.0991	P
54	Threo-Syringoylglycerol	3.713	244.0948	P
55	3-hydroxytetradecanedioic acid	3.717	274.1780	P
56	Netilmicin	3.738	475.2993	P
57	Monomenthyl succinate	3.853	256.1675	P
58	Arginyl-Isoleucine	3.867	287.1965	P
59	2-Methyl-1-phenyl-2- propanyl acetate	3.927	192.1151	P
60	*N*-(3-oxo-octanoyl)- homoserine lactone	3.987	241.1316	P
61	Ethyl (4*Z*)-4,7-octadienoate	4.032	168.1151	P

62	Isopentenyladenine-9-N- glucoside	4.040	363.1911	P
63	Tributyrin	4.096	302.1733	P
64	Capryloylglycine	4.098	201.1366	P
65	Acetyltropine	4.100	183.1258	P
66	1,11-Undecanedicarboxylic acid	4.186	244.1679	P
67	*N*-Methylmescaline	4.259	225.1367	P
68	Phlorin	4.274	288.0850	P
69	Octyl gallate	4.295	282.1465	P
70	(*S,Z*)-Lyratol acetate	4.340	194.1297	P
71	1-Hydroxyacorenone	4.372	250.1563	P
72	Lupinate	4.386	306.1441	P
73	Avenic acid A	4.402	322.1381	P
74	PE(18:4(6*Z*,9*Z*,12*Z*,15*Z*)/22:6(4*Z*,7*Z*,10*Z*,13*Z*,16*Z*,19*Z*))	4.452	783.4833	P
75	1-Octen-3-yl glucoside	4.500	290.1725	P
76	Jasmine ketolactone	4.518	208.1101	P
77	Halstoctacosanolide A	4.528	844.5359	P
78	(*E*)-3-decen-1-ol	4.548	156.1516	P
79	Ethyl decanoate	4.551	200.1777	P
80	Diethofencarb	4.552	267.1472	P
81	Flumetover	4.554	367.1395	P
82	Humulinic acid A	4.580	266.1521	P
83	Ethyl 3-(*N*butylacetamido)propionate	4.651	215.1524	P
84	1,2,3-Tris(1-ethoxyethoxy)propane	4.671	308.2200	P
85	2,2,7,7-Tetramethyl-1,6-dioxaspiro[4.4]nona-3,8-diene	4.679	180.1151	P
86	C12:1n-7	4.685	198.1622	P
87	Ruscopine	4.685	306.2044	P
88	*Gamma*-CEHC	4.688	248.1412	P
89	(5*R*)-5-Hydroxyhexanoic acid	4.731	132.0786	P
90	2-Phenylbutyric acid	4.731	164.0838	P

91	2-Ethylacrylylcarnitine	4.732	244.1551	P
92	2,3-dihydrobenzofuran	4.732	120.0575	P
93	1-Phenyl-6,7-dihydroxyisochroman	4.734	242.0946	P
94	4-(*N*Maleimido)phenyltrimethylammonium	4.741	231.1142	P
95	Arene oxide	4.762	94.0417	P
96	Sterebin E	4.773	338.2443	P
97	Alanyl-Isoleucine	4.781	202.1318	P
98	Methyl 7-epi-12-hydroxyjasmonate glucoside	4.793	402.1889	P
99	N-Isobutyl-2,4,8,10,12-tetradecapentaenamide	4.804	273.2092	P
100	Gravolenic acid	4.805	280.0947	P
101	(*E*)-3-(2-Hydroxyphenyl)- 2-propenal	4.831	148.0526	P
102	Methyl 3-(2,3-dihydroxy- 3-methylbutyl)-4-hydroxybenzoate	4.831	254.1157	P
103	Pinidine	4.852	139.1361	P
104	4,11,13,15-Tetrahydroridentin B	4.893	268.1676	P
105	4'-Hydroxy-3,4,5-trimethoxystilbene	4.963	286.1204	P
106	Selegiline	4.989	187.1365	P
107	Etamiphylline	5.010	279.1702	P
108	1,1,2-Triphenylpropane	5.022	272.1555	P
109	5,7-Megastigmadien-9-ol glucoside	5.039	356.2200	P
110	(*S*)-3-Octanol glucoside	5.102	292.1887	P
111	7,8-Dihydrovomifoliol 9- [rhamnosyl-(1->6)- glucoside]	5.103	534.2678	P
112	(-)-*trans*-Carveol glucoside	5.137	314.1732	P
113	Gibberellin A105	5.137	330.1467	P
114	Cymorcin monoglucoside	5.171	328.1520	P
115	Toxin T2 tetrol	5.244	298.1415	P
116	20-hydroxy-PGF2a	5.244	370.2343	P
117	Cyclonormammein	5.274	374.1725	P
118	PG(16:0/18:0)	5.279	750.5373	P
119	Elaeokanine C	5.288	211.1571	P

120	Artabsinolide A	5.308	280.1310	P
121	Tanacetol B	5.367	296.1988	P
122	Jasmolone glucoside	5.371	342.1679	P
123	Asteltoxin	5.384	418.1993	P
124	Acetyl tributyl citrate	5.386	402.2257	P
125	AF Toxin II	5.432	324.1568	P
126	Hydrocortisone succinate	5.483	462.2253	P
127	Corchoionol C 9- glucoside	5.485	386.1943	P
128	4-Butyl-5-ethylthiazole	5.498	169.0925	P
129	*O*-Methylsomniferine	5.502	622.2665	P
130	11-Hydroxy-9-tridecenoic acid	5.514	228.1724	P
131	Isopulegone caffeate	5.539	316.1674	P
132	Terazosin	5.654	387.1895	P
133	Eremopetasinorol	5.657	208.1466	P
134	(2xi,6xi)-7-Methyl-3- methylene-1,2,6,7-octanetetrol	5.703	204.1363	P
135	Hexanal octane-1,3-diol acetal	5.704	228.2091	P
136	2,6-di-tert- butylbenzoquinone	5.710	220.1466	P
137	1-(4-Amino-2- methylpyrimid-5-ylmethyl)-3-(*beta*- hydroxyethyl)-2- methylpyridinium	5.711	259.1551	P
138	2-Methylundecanal	5.726	184.1828	P
139	Blumenol C *O*- [rhamnosyl-(1->6)- glucoside]	5.739	518.2729	P
140	Avocadienofuran	5.766	246.1985	P
141	(5*alpha*,10*alpha*)-3,7(11)-Eudesmadien-2-one	5.767	218.1670	P
142	Volicitin	5.771	422.2764	P
143	NAc-FnorLRF-amide	5.774	622.3565	P
144	Fluspirilene	5.810	475.2420	P
145	2-Hydroxymyristic Acid	5.833	244.2035	P
146	19(R)-hydroxy-PGE2	5.837	368.2199	P
147	1-(2,4,6-Trimethoxyphenyl)-1,3- butanedione	5.860	252.0998	P
148	Glaudine	5.861	399.1689	P

149	C14:1n-9	5.876	226.1932	P
150	Canavalioside	5.943	546.2679	P
151	*N*-Acetyl-2,6- diethylaniline	5.958	191.1312	P
152	(+/-)-*N,N*-Dimethyl menthyl succinamide	6.014	168.1879	P
153	Capsoside A	6.017	694.3773	P
154	PGH2	6.023	352.2249	P
155	1-Hydroxyepiacorone	6.028	252.1727	P
156	Capsaicin	6.061	305.1989	P
157	Homodihydrojasmone	6.071	180.1516	P
158	20-COOH-Leukotriene B4	6.143	366.2046	P
159	2-Hydroxyestrone	6.145	286.1571	P
160	(*Z*)-6-Nonenal	6.152	140.1202	P
161	Heliosupine	6.171	397.2097	P
162	Penbutolol	6.180	291.2202	P
163	Retapamulin	6.189	517.3250	P
164	Granisetron	6.202	312.1940	P
165	4-Hydroxy-3-methoxy- 2,10-bisaboladien-9-one	6.211	266.1882	P
166	(+)-Prosopinine	6.231	313.2614	P
167	Chalciporone	6.284	243.1620	P
168	Aminoparathion	6.290	261.0599	P
169	Europine	6.303	329.1833	P
170	Gravelliferone	6.307	298.1571	P
171	*N*,2,3-Trimethyl-2-(1- methylethyl)butanamide	6.336	171.1621	P
172	1,1-Diethoxy-2-hexene	6.355	172.1465	P
173	*alpha*-Butyl-*omega*-hydroxypoly(oxyethylene) poly(oxypropylene)	6.356	248.1989	P
174	Cuscohygrine	6.372	224.1889	P
175	Pterosin O	6.395	232.1466	P
176	Metoprolol	6.397	267.1835	P
177	8-Acetoxy-4-acoren-3- one	6.432	278.1877	P

178	15-keto-Prostaglandin E2	6.513	350.2085	P
179	Momilactone B	6.567	330.1835	P
180	9-HOTE	6.570	294.2195	P
181	Chaksine	6.609	450.2968	P
182	Monoisobutyl phthalic acid	6.666	222.0892	P
183	10-Hydroxy-2,8- decadiene-4,6-diynoic acid	6.667	176.0474	P
184	C16 Sphinganine	6.681	273.2672	P
185	*p*- Hydroxybenzyldesulphoglucosinolate	6.695	345.0869	P
186	2-Furanmethanol	6.697	98.0367	P
187	Dihydrocapsaicin	6.704	307.2148	P
188	3-Hydroxy-6,8- dimethoxy-7(11)- eremophilen-12,8-olide	6.704	310.1782	P
189	17-Methylandrosta-2,4-dieno[2,3-d]isoxazol-17beta-ol	6.709	327.2195	P
190	2-Tetradecanone	6.714	212.2141	P
191	Glicoisoflavanone	6.715	384.1566	P
192	5-(2,3-Dihydroxy-3-methylbutyl)-4-(3,4-epoxy-4-methylpentanoyl)-3,4-dihydroxy-2-isopentanoyl-2-cyclopenten-1-one	6.734	412.2098	P
193	Tolmetin glucuronide	6.735	433.1383	P
194	Tigloidine	6.754	223.1570	P
195	Phytosphingosine	6.756	317.2929	P
196	Ximelagatran	6.758	473.2628	P
197	Cerivastatin	6.759	459.2440	P
198	Mycalamide B	6.781	517.2891	P
199	Trilobolide	6.781	522.2441	P
200	Erysothiopine	6.783	407.1027	P
201	Porson	6.784	386.1731	P
202	Cinegalline	6.784	430.2107	P
203	16-hydroxy hexadecanoic acid	6.786	272.2353	P
204	Canescein	6.804	566.2703	P
205	Funtumine	6.844	317.2720	P

206	2-Pentadecanone	6.852	226.2296	P
207	(*S*)-Nerolidol 3-*O*-[a-LRhamnopyranosyl-(1->4)-a-Lrhamnopyranosyl-(1->2)-b-Dglucopyranoside]	6.865	676.3672	P
208	13,14-dihydro-15-keto-PGA2	6.878	334.2146	P
209	5-Dodecyldihydro-2(3H)-furanone	6.881	254.2248	P
210	3-(5,6,6-Trimethylbicyclo[2.2.1]h ept-1-yl)cyclohexanol	6.881	236.2141	P
211	Pumiliotoxin 251D	6.892	251.2251	P
212	Genipin 1-betagentiobioside	6.899	550.1900	P
213	Diclomezine	6.941	254.0001	P
214	4,5-Dihydroniveusin A	6.963	396.1780	P
215	3'-Hydroxy-HT2 toxin	6.975	440.2042	P
216	Muricatacin	6.989	284.2352	P
217	Plantaricin BN	6.990	484.2308	P
218	Coccinin	7.001	528.2573	P
219	Lauroyl diethanolamide	7.005	287.2462	P
220	Palmitic amide	7.031	255.2563	P
221	BILA 2185BS	7.043	618.3255	P
222	Phosphoric acid	7.043	97.9769	P
223	3*beta*-Hydroxypregn-5-ene	7.050	302.2608	P
224	Cyclotetradecane	7.059	196.2192	P
225	Imidaprilat	7.081	377.1590	P
226	Myxochelin A	7.089	404.1577	P
227	Terbucarb	7.094	277.2040	P
228	2-Methoxyestradiol-3-methylether	7.122	316.2023	P
229	Nonyl octanoate	7.145	270.2558	P
230	7-Hydroxy-3-(4-methoxyphenyl)-4-methylcoumarin	7.186	282.0895	P
231	10,16-dihydroxy-palmitic acid	7.193	288.2301	P
232	*Cis*-5-Tetradecenoylcarnitine	7.197	370.2970	P
233	Cincassiol B	7.242	400.2099	P

234	Armillaric acid	7.243	416.1831	P
235	6-*O*-Acetylaustroinulin	7.244	364.2616	P
236	Allopumiliotoxin 267A	7.249	267.2199	P
237	*trans*-9, *trans*-11- octadecadienoic acid; C18:2n-7,9	7.251	280.2403	P
238	Dodecylguanidine	7.260	227.2362	P
239	Bleekerine	7.317	409.1758	P
240	6-*trans*-LTB4	7.357	336.2301	P
241	9-Decenoylcholine	7.379	256.2279	P
242	3-Methyl-*alpha*-ionyl acetate	7.380	250.1932	P
243	Panaquinquecol 1	7.381	292.2038	P
244	*N*-Dealkylatedtolterodine	7.383	283.1933	P
245	1,8-Heptadecadiene-4,6- diyne-3,10-diol	7.403	260.1777	P
246	Physagulin C	7.437	542.2506	P
247	Cyclocalopin F	7.459	294.1105	P
248	Erythroskyrin	7.459	455.2309	P
249	Austalide L	7.460	428.2200	P
250	(3'x,5'a,9'x,10'b)-*O*-(3-Hydroxy-6-oxo-7-drimen- 11-yl)umbelliferone	7.460	396.1938	P
251	DHAP(18:0)	7.460	436.2597	P
252	(4-Methylphenyl)acetaldehyde	7.461	134.0731	P
253	Ethyl vanillin isobutyrate	7.472	236.1050	P
254	Methyl (9*Z*)-10'-oxo-6,10'-diapo-6-carotenoate	7.500	312.1726	P
255	Norpropoxyphene	7.511	325.2035	P
256	7,10-Hexadecadienoic acid	7.570	252.2090	P
257	Sphinganine	7.598	301.2983	P
258	2,2-Dimethyl-3,4-bis(4-methoxyphenyl)-2H-1-benzopyran-7-ol acetate	7.631	430.1785	P
259	Vilazodone	7.631	441.2160	P
260	Armillaripin	7.632	414.2045	P
261	2-Hexadecanone	7.637	240.2455	P
262	Biperiden	7.677	311.2247	P

263	Spiroxamine	7.729	297.2668	P
264	Zucchini factor B	7.763	663.4307	P
265	6,10,14-Trimethyl- 5,9,13-pentadecatrien-2- one	7.773	262.2294	P
266	2,6-Di-tert-butyl-4-ethylphenol	7.775	234.1983	P
267	Methyloctatropine	7.790	282.2433	P
268	Phlegmarine	7.831	250.2410	P
269	Glaucamine	7.836	385.1524	P
270	Sanshodiol	7.836	358.1417	P
271	Phenethyl decanoate	7.870	276.2089	P
272	Methadone	7.871	309.2089	P
273	Methyl 15-cyanopentadecanoate	7.882	281.2357	P
274	Elaiophylin	7.942	1024.593<br>9	P
275	(10*S*)-Juvenile hormone III diol	7.951	284.1997	P
276	Dodecanamide	7.960	199.1937	P
277	Asparagoside D	7.962	902.4884	P
278	Santalyl acetate	7.962	262.1934	P
279	3*beta*-Fluoro-5*beta*pregnan-20-one	7.972	320.2521	P
280	Scopoloside II	8.002	770.4094	P
281	Stearamide	8.008	283.2877	P
282	MG(0:0/18:1(11*Z*)/0:0)	8.012	356.2926	P
283	Leucomycin A9	8.016	743.4093	P
284	Corchorusoside B	8.032	682.3570	P
285	Undecylprodigiosin	8.173	393.2783	P
286	Pipericine	8.273	335.3174	P
287	Lyngbyatoxin	8.275	437.3043	P
288	17*beta*-Acetamidoandrost-4-en-3-one	8.275	329.2350	P
289	Tributyl phosphate	8.305	266.1648	P
290	1-Phenyl-1,3- dodecanedione	8.312	274.1933	P

291	Estrane-3α,17α-diol	8.322	278.2247	P
292	Lentiginosine	8.339	157.1105	P
293	Methyl 2-octynoate	8.358	154.0994	P
294	Methyl 2*E*,4*Z*- hexadecadienoate	8.397	266.2246	P
295	Kukoamine D	8.405	530.3125	P
296	Triphenyl phosphate	8.408	326.0711	P
297	B 823-08	8.409	353.0822	P
298	Methyprylon	8.449	183.1261	P
299	A28086B	8.496	762.4882	P
300	12*S*-HEPE	8.509	318.2198	P
301	3L,7D,11D-phytanic acid	8.511	312.3032	P
302	Polidocanol	8.517	582.4343	P
303	*N*-(14-Methylhexadecanoyl)pyrrolidine	8.520	323.3191	P
304	Dodemorph	8.533	281.2718	P
305	8,8-Diethoxy-2,6-dimethyl-2-octanol	8.542	246.2194	P
306	Protoprimulagenin A 3-[rhamnosyl-(1->4)-rhamnosyl-(1->4)-[rhamnosyl-(1->2)]-glucosyl-(1->?)-glucuronide]	8.543	1234.5933	P
307	(3a,5b,7a,12a)-24-[(carboxymethyl)amino]-1,12-dihydroxy-24-oxocholan-3-yl-b-Dglucopyranosiduronic a	8.544	641.3415	P
308	Polysorbate 20	8.615	522.3409	P
309	Polysorbate 60	8.765	434.2882	P
310	Laserpitin	8.766	450.2614	P
311	Hexyl heptanoate	8.787	638.2362	P
312	9-Acetoxyfukinanolide	8.865	292.1675	P
313	Gabapentin	8.879	171.1262	P
314	*N*-Methylpelletierine	8.880	155.1309	P
315	MG(0:0/20:1(11Z)/0:0)	8.920	384.3240	P
316	Tris(butoxyethyl)phosphate	8.922	398.2436	P
317	3-Cyclohexyldodecane	9.007	252.2818	P

318	Isoacitretin	9.035	326.1883	P
319	MG(0:0/22:5(4*Z*,7*Z*,10*Z*,13*Z*,16Z)/0:0)	9.048	404.2923	P
320	*Alpha*-CEHC	9.107	278.1520	P
321	22-Oxo-docosanoate	9.131	354.3133	P
322	19-Hydroxycinnzeylanol 19-glucoside	9.156	562.2622	P
323	(*E*)-1-[4-Hydroxy-3-(3-methyl-1,3-butadienyl)phenyl]-2- (3,5-dihydroxyphenyl)ethylene	9.158	294.1259	P
324	MG(0:0/22:2(13Z,16Z)/0:0)	9.162	410.3401	P
325	(*E,E*)-1,6-bis(4-methoxyphenyl)-1,5- hexadiene	9.190	294.1621	P
326	1-(3-Hydroxy-4-methoxyphenyl)-1,2-ethanediol	9.201	376.1884	P
327	18-Oxocortisol	9.203	418.2681	P
328	Misoprostol	9.203	382.2701	P
329	Lansioside A	9.214	659.4377	P
330	Linoleoyl Ethanolamide	9.291	323.2826	P
331	Chloropyramine	9.364	289.1357	P
332	Bioresmethrin	9.366	338.1885	P
333	MG(0:0/22:6(4*Z*,7*Z*,10*Z*,13*Z*,16*Z*,19*Z*)/0:0)	9.428	402.2758	P
334	(3b,6b,8b,12a)-8,12-Epoxy-7(11)-eremophilene-6- angeloyloxy-8,12- dimethoxy-3-ol	9.430	394.2359	P
335	[6]-Gingerdiol 3,5- diacetate	9.430	380.2203	P
336	Methandriol dipropionate	9.431	416.2915	P
337	Lilac alcohol	9.431	170.1307	P
338	3'-Hydroxy-T2-triol	9.431	398.1923	P
339	Iriomoteolide 1a	9.445	506.3222	P
340	MG(0:0/18:3(6*Z*,9*Z*,12*Z*)/0:0)	9.457	352.2621	P
341	2-(4-Chloro-3,5-dimethylphenoxy)-*N*-(2-phenyl-2H-benzotriazol-5-yl)-acetamide	9.640	406.1195	P
342	Monocrotaline	9.640	325.1528	P
343	4*beta*-(2-Aminoethylthio)catechin	9.641	365.0929	P
344	Palmitoyl glucuronide	9.731	418.2936	P
345	Palmitoyl-EA	9.795	299.2817	P

346	6,8a-Seco-6,8a-deoxy-5-oxoavermectin "2a" aglycone	9.817	586.3510	P
347	MG(0:0/22:1(13*Z*)/0:0)	9.833	412.3554	P
348	Ampalex	9.939	241.1204	P
349	1b,3a,7a,12a-Tetrahydroxy-5b cholanoic acid	9.941	424.2811	P
350	Arbutin	9.941	272.0897	P
351	Vanillactic acid	9.942	212.0685	P
352	Diflorasone	9.942	410.1890	P
353	Glucosylceramide (d18:1/9*Z*-18:1)	10.009	725.5791	P
354	Balofloxacin	10.193	389.1760	P
355	Tamoxifen	10.197	371.2251	P
356	DU 122290	10.199	362.1653	P
357	(±)-(*Z*)-2-(5-Tetradecenyl)cyclobutanone	10.489	264.2456	P
358	7*alpha*,12*alpha*-Dihydroxy-3-oxochol-4-en-24-oic Acid	10.490	404.2571	P
359	Lucidumol A	10.501	472.3556	P
360	Oleamide	10.589	281.2719	P
361	4-Nerolidylcatechol	10.589	314.2245	P
362	Drospirenone	10.682	366.2199	P
363	Capsi-amide	10.732	269.2718	P
364	(3*S*,6*E*,10*E*)-1,6,10,14-Phytatetraen-3-ol	10.737	290.2611	P
365	D-Glucosyldihydrosphingosine	10.807	463.3513	P
366	2-Pentadecylfuran	10.831	278.2611	P
367	Enalkiren	10.843	656.4294	P
368	Cavipetin D	10.893	418.2718	P
369	b-Hydroxypropionyl-CoA	10.893	839.1316	P
370	Sorbitan palmitate	10.894	402.2985	P
371	Ganodermic acid TQ	11.091	510.3346	P
372	*N*-hexadecanoylpyrrolidine	11.387	309.3033	P
373	Phenolic phosphate	11.763	174.0081	P
374	PC(14:0/22:5(4*Z*,7*Z*,10*Z*,13*Z*,16*Z*))	12.254	780.5531	P

375	12-Ketodeoxycholic acid	12.256	390.2773	P
376	PC(16:0/18:1(9*Z*))[*S*]	12.269	760.5859	P
377	Dioctyl hexanedioate	12.281	370.3085	P
378	Luffariellolide	12.282	386.2828	P
379	Hyperforin	13.784	536.3877	P
380	(3*beta*,5*alpha*,6*beta*,7*alpha*,22*E*,24*R*)-Ergosta-8,22-diene-3,5,6,7-tetrol	13.790	446.3394	P
381	Didodecyl thiobispropanoate	13.835	514.4059	P

[a] – retention time [min]

[b] –compound detection in positive (P) or in negative (N) ionization mode.
