# Supplementary material for: Biological Properties of the Mucus and Eggs of Helix aspersa Müller as a Potential Cosmetic and Pharmaceutical Raw Material: A Preliminary Study
Source: Int J Mol Sci. 2024 Sep 15;25(18):9958. doi: 10.3390/ijms25189958 (PMC11432642; doi:10.3390/ijms25189958)
Supplement: Supplementary file 1 [file ijms-25-09958-s001.zip › Herman Anna - Table S5.pdf]

Table S5. Compounds identified in acetonitrile-water* extract of fresh egg of organic *Helix aspersa* snail using LC-MS.

No	Metabolite	RT[a] [min]	Mass [*m/z*]	Detection mode[b]
1	Trifluoroacetic acid	0.253	113.9929	N
2	3-Deoxyarabinohexonic acid	0.259	180.0635	N
3	3-b-Galactopyranosyl glucose	0.27	342.1164	N
4	*N*-n-Hexanoylglycine methyl ester	3.465	187.1210	N
5	Methyl *N*-(amethylbutyryl)glycine	4.303	188.1049	N
6	1,4-Dihydroxy-2-methylanthraquinone	4.725	254.0581	N
7	Apigenin	5.358	270.0527	N
8	Methazolamide	5.608	236.0049	N
9	D-Ribose 1-diphosphate	5.617	293.9902	N
10	Ethiprole	5.806	395.9833	N
11	Zingerone	6.228	194.0944	N
12	Bismuth subsalicylate	6.706	361.9977	N
13	Nordihydrocapsiate	6.832	294.1832	N
14	BILA 2185BS	7.039	618.3253	N
15	4-Hydroxy-5-phenyltetrahydro-1,3-oxazin-2-one	7.055	193.0741	N
16	Flupropanate	7.089	145.9992	N
17	(*S,Z*)-Lyratol acetate	7.115	194.1308	N
18	3b-Allotetrahydrocorticosterone	7.119	350.2455	N
19	(3b,6b,8b,12a)-8,12-Epoxy-7(11)-eremophilene-6-angeloyloxy-8,12-dimethoxy-3-ol	7.200	394.2353	N
20	Lauryl hydrogen sulfate	7.284	266.1551	N
21	Zanthodioline	7.287	305.1267	N
22	Losartan	7.314	422.1622	N
23	Methotrexate	7.315	454.1729	N
24	L-Tyrosine methyl ester	7.342	195.0897	N
25	*N*-Methyl-14-Odemethylepiporphyroxine	7.459	371.1366	N
26	*N*- Undecylbenzenesulfonic acid	7.727	312.1760	N

27	2-Dodecylbenzenesulfonic acid	8.163	326.1915	N
28	Sodium Tetradecyl Sulfate	8.204	294.1863	N
29	Dinoterb	8.248	240.0749	N
30	(+)-Prosopinine	8.272	313.2618	N
31	Kukoamine D	8.410	530.3122	N
32	Alcaftadine	8.885	307.1683	N
33	Gemfibrozil	8.954	250.1570	N
34	Furmecyclox	9.278	251.1524	N
35	Nisoldipine	9.389	388.1633	N
36	(5b,7a,12a)-2-(3-methoxyphenyl)-2-oxoethyl ester-7,12-dihydroxy-cholan-24-oic acid	10.293	540.3451	N
37	2-Mercaptopropanoic acid	10.325	106.0089	N
38	Oleamide	10.487	281.2709	N
39	Methyl tetradecanoate	10.500	242.2249	N
40	5-Dodecyldihydro-2(3H)-furanone	10.501	254.2246	N
41	Armillatin	10.841	610.4230	N
42	3-Hydroxy-2-(4-morpholinylmethyl)estra-1,3,5(10)-trien-17-one	10.947	369.2303	N
43	Ganoderic acid V	11.122	528.3449	N
44	Tridecanoic acid, 4,8,12-trimethyl-; 4,8,12-Trimethyltridecanoic acid	11.133	256.2402	N
45	Butroxydim	11.268	399.2407	N
46	Adlupone	11.367	482.3394	N
47	Drotaverine	11.443	397.2257	N
48	Stearic acid	11.955	284.2717	N
49	24-Acetyl-25-cinnamoylvulgaroside	12.064	608.3352	N
50	(3*beta*,22*E*,24*R*)-3-Hydroxyergosta-5,8,22-trien-7-one	12.501	410.3180	N
1	Asulam	0.263	230.0365	P
2	Pandamarilactam 3x	0.264	235.1210	P
3	Aminocaproic acid	0.266	131.0947	P
4	Isoamyl nitrite	0.267	117.0791	P
5	L-Carnitine	0.267	162.1129	P

6	Sucrose	0.268	342.1165	P
7	4'-Hydroxy-2- biphenylcarboxylic acid	0.271	214.0627	P
8	Benzo[b]naphtho[2,1- d]thiophene	0.271	234.0509	P
9	D-Ribitol 5-phosphate	0.272	232.0351	P
10	Penicillin O	0.272	330.0701	P
11	Marmesin rhamnoside	0.275	392.1471	P
12	1-Deoxy-D-glucitol	0.293	166.0842	P
13	Bethanidine	0.317	177.1274	P
14	2-Methyl-4-pentenal	0.318	98.0728	P
15	2-Amino-2-methyl-1,3-propanediol	0.391	105.0789	P
16	Trolamine	0.396	149.1052	P
17	Dexpanthenol	0.867	205.1315	P
18	2,5-Dihydro-2,4,5-trimethyloxazole	1.520	113.0840	P
19	2E-Decenedioic acid	2.517	200.1050	P
20	O-hexanoyl-R-carnitine	2.780	260.1857	P
21	5-Heptyltetrahydro-2-oxo-3-furancarboxylic acid	2.963	228.1361	P
22	3-hydroxytetradecanedioic acid	3.028	274.1778	P
23	Sedanonic acid	3.132	210.1247	P
24	Capryloylglycine	3.149	326.1943	P
25	Aprindine	3.168	322.2419	P
26	Wine lactone	3.307	166.0994	P
27	*N*-n-Hexanoylglycine methyl ester	3.465	187.1209	P
28	Homoarecoline	3.466	169.1103	P
29	Varenicline	3.508	211.1110	P
30	(*E*)-2-Methyl-2-buten-1-ol *O*-beta-DGlucopyranoside	3.569	248.1245	P
31	Istamycin C1	3.584	431.2733	P
32	Phlorin	3.709	288.0845	P
33	Threo-Syringoylglycerol	3.712	244.0949	P
34	Netilmicin	3.735	475.2996	P

35	Geranyl acetoacetate	3.850	238.1570	P
36	Monomenthyl succinate	3.850	256.1675	P
37	Arginyl-Isoleucine	3.866	287.1969	P
38	2-Methyl-1-phenyl-2-propanyl acetate	3.923	192.1151	P
39	*N*-(3-oxo-octanoyl)-homoserine lactone	3.983	241.1316	P
40	Methyprylon	4.021	183.1261	P
41	Tributyrin	4.024	302.1729	P
42	Isopentenyladenine-9-*N*-glucoside	4.043	363.1911	P
43	Jasmine ketolactone	4.256	208.1098	P
44	*N*-Methylmescaline	4.257	225.1367	P
45	2,2,7,7-Tetramethyl-1,6- dioxaspiro[4.4]nona-3,8- diene	4.296	180.1152	P
46	Humulinic acid A	4.387	266.1519	P
47	Oseltamivir	4.387	312.2052	P
48	Triethylenemelamine	4.398	204.1132	P
49	Octyl gallate	4.399	282.1466	P
50	Amyl 2-furoate	4.405	182.0945	P
51	Daphnetoxin	4.422	482.1915	P
52	1-Octen-3-yl glucoside	4.499	290.1729	P
53	Halstoctacosanolide A	4.527	844.5357	P
54	Gentamicin C2b	4.540	463.3022	P
55	(*E*)-3-decen-1-ol	4.549	156.1515	P
56	Ethyl decanoate	4.551	200.1777	P
57	Flumetover	4.551	367.1393	P
58	Diethofencarb	4.553	267.1471	P
59	1,2,3-Tris(1-ethoxyethoxy)propane	4.670	308.2200	P
60	2-Hexenoylcholine	4.671	200.1652	P
61	Aspergillic acid	4.676	224.1526	P
62	C12:1n-7	4.684	198.1620	P
63	*Gamma*-CEHC	4.686	248.1412	P

64	11-Hydroxy-9-tridecenoic acid	4.694	228.1723	P
65	Methyl 7-epi-12- hydroxyjasmonate glucoside	4.721	402.1890	P
66	1-Phenyl-6,7-dihydroxyisochroman	4.730	242.0945	P
67	2,3-dihydrobenzofuran	4.730	120.0576	P
68	2-Phenylbutyric acid	4.730	164.0838	P
69	(5*R*)-5-Hydroxyhexanoic acid	4.731	132.0787	P
70	2-Ethylacrylylcarnitine	4.731	244.1551	P
71	Kinetin-9-*N*-glucoside	4.760	377.1324	P
72	Alanyl-Isoleucine	4.780	202.1317	P
73	*N*-Isobutyl-2,4,8,10,12-tetradecapentaenamide	4.801	273.2094	P
74	Gravolenic acid	4.803	280.0952	P
75	Pinidine	4.838	139.1361	P
76	2-Aminoadenosine	4.847	282.1082	P
77	GW 1929	4.874	495.2135	P
78	4,11,13,15-Tetrahydroridentin B	4.908	268.1675	P
79	Ganglioside GM3(d18:1/16:0)	4.945	1152.7190	P
80	4'-Hydroxy-3,4,5-trimethoxystilbene	4.961	286.1202	P
81	Metaldehyde	4.963	176.1049	P
82	2-Phenylethyl *beta*-Dglucopyranoside	5.012	284.1264	P
83	1,1,2-Triphenylpropane	5.020	272.1555	P
84	5,7-Megastigmadien-9-ol glucoside	5.037	356.2196	P
85	Sterebin E	5.075	338.2461	P
86	Z-Arg-Arg-NHMec	5.079	621.3052	P
87	(*S*)-3-Octanol glucoside	5.099	292.1882	P
88	(-)-*trans*-Carveol glucoside	5.135	314.1733	P
89	Gibberellin A105	5.136	330.1464	P
90	7,8-Dihydrovomifoliol 9-[rhamnosyl-(1->6)-glucoside]	5.162	534.2671	P
91	Toxin T2 tetrol	5.242	298.1413	P
92	Dihydro-5-(2-octenyl)-2(3H)-furanone	5.268	196.1464	P

93	Cyclonormammein	5.273	374.1727	P
94	Jasmolone glucoside	5.371	342.1680	P
95	Asteltoxin	5.384	418.1992	P
96	Cinitapride	5.385	402.2257	P
97	Ethyl 7-epi-12-hydroxyjasmonate glucoside	5.411	416.2046	P
98	AF Toxin II	5.429	324.1575	P
99	Dulciol C	5.476	482.2317	P
100	Hydrocortisone succinate	5.481	462.2255	P
101	*O*-Methylsomniferine	5.500	622.2658	P
102	(*E,E,E*)-*N*-(2-Methylpropyl)hexadeca-2,6,8-trien-10-ynamide	5.503	301.2407	P
103	4-Butyl-5-ethylthiazole	5.521	169.0926	P
104	Isopulegone caffeate	5.555	316.1675	P
105	Satratoxin H	5.567	528.2334	P
106	Blumenol C glucoside	5.685	372.2148	P
107	(2xi,6xi)-7-Methyl-3-methylene-1,2,6,7-octanetetrol	5.702	204.1363	P
108	Hexanal octane-1,3-diol acetal	5.702	228.2090	P
109	2-Methylundecanal	5.723	184.1828	P
110	Blumenol C O- [rhamnosyl-(1->6)- glucoside]	5.737	518.2732	P
111	(5*alpha*,10*alpha*)-3,7(11)-Eudesmadien-2-one	5.764	218.1668	P
112	Avocadienofuran	5.764	246.1984	P
113	2-Furanmethanol	5.765	98.0367	P
114	Volicitin	5.768	422.2767	P
115	NAc-FnorLRF-amide	5.772	622.3561	P
116	Fluspirilene	5.804	475.2419	P
117	19(*R*)-hydroxy-PGE2	5.837	368.2199	P
118	Glaucamine	5.857	385.1530	P
119	Sanshodiol	5.858	358.1420	P
120	C14:1n-9	5.872	226.1932	P
121	Eriojaposide A	5.874	502.2413	P

122	Canavalioside	5.940	546.2678	P
123	Norerythrostachaldine	5.940	407.2654	P
124	Ssioriside	5.974	554.2360	P
125	(+/-)-*N,N*-Dimethyl menthyl succinamide	6.014	168.1878	P
126	Capsoside A	6.014	694.3777	P
127	Cinncassiol A 19- glucoside	6.017	544.2521	P
128	15-Acetoxyscirpene-3,4- diol 4-*O*-a-D- glucopyranoside	6.023	486.2101	P
129	Capsaicin	6.059	305.1987	P
130	Homodihydrojasmone	6.068	180.1513	P
131	Lauroyl diethanolamide	6.070	287.2463	P
132	2-Carboxy-1-[5-(2- carboxy-1-pyrrolidinyl)- 2-hydroxy-2,4-pentadienylidene]pyrrolid inium	6.078	309.1466	P
133	(+)-Prosopinine	6.138	313.2614	P
134	20-COOH-Leukotriene B4	6.140	366.2043	P
135	2-Hydroxyestrone	6.144	286.1570	P
136	(*Z*)-6-Nonenal	6.150	140.1202	P
137	Penbutolol	6.176	291.2202	P
138	1*alpha*,5*alpha*- Dimercaptoandrostane- 3*alpha*,17*beta*-diol	6.202	356.1840	P
139	4-Hydroxy-3-methoxy- 2,10-bisaboladien-9-one	6.206	266.1880	P
140	4,5-Dihydroniveusin A	6.244	366.2043	P
141	Chalciporone	6.281	140.1202	P
142	3'-Hydroxy-HT2 toxin	6.288	414.2367	P
143	Gravelliferone	6.303	635.3152	P
144	*N*,2,3-Trimethyl-2-(1- methylethyl)butanamide	6.331	331.2720	P
145	Plantaricin BN	6.336	534.3516	P
146	*alpha*-Butyl-*omega*- hydroxypoly(oxyethylene) poly(oxypropylene)	6.355	266.1880	P
147	Cuscohygrine	6.371	239.1886	P
148	Dihydrocapsaicin	6.383	307.2151	P
149	1-Hydroxyacorenone	6.513	250.1567	P
150	Armillatin	6.534	610.4231	P

151	Momilactone B	6.644	330.1830	P
152	10-Hydroxy-2,8- decadiene-4,6-diynoic acid	6.662	176.0474	P
153	Monoisobutyl phthalic acid	6.663	222.0892	P
154	C16 Sphinganine	6.678	273.2671	P
155	*p*-Hydroxybenzyldesulphoglucosinolate	6.692	345.0868	P
156	Fetidine	6.693	682.3269	P
157	Phenethyl decanoate	6.701	276.2089	P
158	17-Methylandrosta-2,4-dieno[2,3-d]isoxazol-17*beta*-ol	6.706	327.2201	P
159	2,4,12-Octadecatrienoic acid isobutylamide	6.707	333.3018	P
160	Glicoisoflavanone	6.711	384.1573	P
161	2-Tetradecanone	6.712	212.2143	P
162	1-Isomangostin hydrate	6.732	428.1833	P
163	5-(2,3-Dihydroxy-3-methylbutyl)-4-(3,4-epoxy-4-methylpentanoyl)-3,4-dihydroxy-2-isopentanoyl-2-cyclopenten-1-one	6.732	412.2101	P
164	1*alpha*,3*beta*,22*R*Trihydroxyergosta-5,24Edien-26-oic acid 3-*O*-b-D-glucoside 26-*O*-[b-Dglucosyl-(1->2)-b-Dglucosyl] ester	6.754	946.4696	P
165	Phytosphingosine	6.754	317.2931	P
166	Deacetylnomilin	6.755	472.2095	P
167	Ximelagatran	6.755	473.2630	P
168	Austalide A	6.778	516.2355	P
169	Mycalamide B	6.779	517.2889	P
170	Trilobolide	6.780	522.2440	P
171	Porson	6.783	386.1727	P
172	16-hydroxy hexadecanoic acid	6.785	272.2352	P
173	Canescein	6.802	566.2705	P
174	Ipecoside	6.802	565.2176	P
175	Funtumine	6.842	317.2720	P
176	2-Pentadecanone	6.852	226.2299	P
177	(*S*)-Nerolidol 3-*O*-[a-LRhamnopyranosyl-(1->4)-a-Lrhamnopyranosyl-(1->2)-b-Dglucopyranoside]	6.862	676.3671	P
178	Palmitic amide	6.874	255.2565	P

179	5-Dodecyldihydro-2(3H)-furanone	6.879	254.2247	P
180	Pumiliotoxin 251D	6.893	251.2249	P
181	Genipin 1-*beta*gentiobioside	6.898	550.1898	P
182	Zizybeoside II	6.918	594.2167	P
183	2-Hexadecanone	6.929	240.2453	P
184	Kanokoside C	6.937	638.2426	P
185	Chrycolide	6.939	232.0183	P
186	Coriandrone D	6.960	352.1520	P
187	Nonyl octanoate	6.987	270.2560	P
188	Muricatacin	6.989	284.2351	P
189	Coccinin	7.001	528.2570	P
190	6-Caffeoylsucrose	7.020	504.1482	P
191	Acetyl Tyrosine Ethyl Ester	7.028	251.1158	P
192	BILA 2185BS	7.041	618.3260	P
193	Cyclotetradecane	7.056	196.2192	P
194	Xylopinine	7.080	355.1766	P
195	Osajin	7.086	404.1606	P
196	Spiroxamine	7.100	297.2668	P
197	Purothionin AII	7.123	520.2400	P
198	Finaconitine	7.124	630.3153	P
199	Paucin	7.173	468.1999	P
200	9-HOTE	7.178	294.2198	P
201	7-Hydroxy-3-(4-methoxyphenyl)-4-methylcoumarin	7.184	282.0895	P
202	Z-Gly-Pro-Leu-Gly-Pro	7.189	573.2787	P
203	10,16-dihydroxy-palmitic acid	7.190	288.2302	P
204	*cis*-5-Tetradecenoylcarnitine	7.196	370.2969	P
205	Armillaric acid	7.240	416.1831	P
206	Cincassiol B	7.240	400.2097	P
207	Allopumiliotoxin 267A	7.247	267.2201	P

208	*trans*-9, *trans*-11- octadecadienoic acid; C18:2n-7,9	7.250	280.2406	P
209	Trimethylolpropane trimethacrylate	7.257	338.1730	P
210	Ethyl (4*Z*)-4,7-octadienoate	7.281	168.1152	P
211	Panaquinquecol 1	7.293	292.2039	P
212	Bleekerine	7.315	409.1759	P
213	Testolactone	7.337	300.1728	P
214	Estrane-3α,17α-diol	7.378	278.2244	P
215	*N*-Dealkylatedtolterodine	7.378	283.1936	P
216	7,10-Hexadecadienoic acid	7.409	252.2089	P
217	Physagulin C	7.436	542.2509	P
218	Etiocholan-3α-ol-17-one 3-glucuronide	7.452	466.2571	P
219	(3'x,5'a,9'x,10'b)-*O*-(3-Hydroxy-6-oxo-7-drimen- 11-yl)umbelliferone	7.457	396.1936	P
220	Cyclocalopin F	7.457	294.1108	P
221	Picrasin C	7.457	422.2310	P
222	2,2-Dimethyl-3,4-bis(4-methoxyphenyl)-2H-1-benzopyran-7-ol acetate	7.458	430.1779	P
223	*alpha*-Methylstyrene	7.458	118.0783	P
224	Armillaripin	7.458	414.2042	P
225	Austalide L	7.458	428.2202	P
226	Erythroskyrin	7.458	455.2310	P
227	Artabsinolide A	7.460	280.1313	P
228	Methyl (9*Z*)-10'-oxo-6,10'-diapo-6-carotenoate	7.499	312.1724	P
229	Norpropoxyphene	7.512	325.2041	P
230	Fumonisin A2	7.550	747.4047	P
231	Steviolbioside	7.581	642.3254	P
232	Sphinganine	7.598	301.2983	P
233	Avocadenofuran	7.628	248.2138	P
234	8-Pentanoylneosolaniol	7.659	466.2204	P
235	Biperiden	7.676	311.2245	P

236	Palmitoyl-EA	7.721	299.2825	P
237	1-(4-Amino-2-methylpyrimid-5-ylmethyl)-3-(betahydroxyethyl)-2-methylpyridinium	7.746	259.1548	P
238	Zucchini factor B	7.764	663.4308	P
239	Methyl 15-cyanopentadecanoate	7.788	281.2354	P
240	(*E*)-3-(2-Hydroxyphenyl)- 2-propenal	7.836	148.0525	P
241	1-Methyl-2-nonyl-4(1H)-quinolinone	7.859	285.2088	P
242	Methyloctatropine	7.879	282.2436	P
243	5-Hexyltetrahydro-2-furanoctanoic acid	7.905	298.2502	P
244	Dihydrodioscorine	7.905	223.1575	P
245	Elaeokanine C	7.905	211.1573	P
246	6,10,14-Trimethyl- 5,9,13-pentadecatrien-2- one	7.913	262.2299	P
247	Elaiophylin	7.941	1024.5931	P
248	Coutaric acid	7.947	349.2009	P
249	(3a,5b)-24-oxo-24-[(2-sulfoethyl)amino]cholan-<br>3-yl-b-Dglucopyranosiduronic acid	7.949	659.3338	P
250	Hematoporphyrin	7.955	598.2811	P
251	Dodecanamide	7.958	199.1937	P
252	Asparagoside D	7.963	902.4876	P
253	2-Methoxyestradiol-3-methylether	7.991	316.2038	P
254	Scopoloside II	8.002	770.4088	P
255	2-Methoxyestrone 3-sulfate	8.013	380.1296	P
256	MG(0:0/18:1(11*Z*)/0:0)	8.014	356.2927	P
257	Leucomycin A9	8.016	743.4092	P
258	Corchorusoside B	8.031	682.3561	P
259	Methadone	8.035	309.2096	P
260	Pristanic acid	8.055	298.2866	P
261	Convallatoxin	8.091	550.2772	P
262	Undecylprodigiosin	8.175	393.2785	P
263	17*beta*-Acetamidoandrost-4-en-3-one	8.273	329.2353	P

264	Pipercitine	8.274	349.3330	P
265	Santene	8.274	122.1097	P
266	Lyngbyatoxin	8.275	437.3044	P
267	Tributyl phosphate	8.304	266.1649	P
268	1-Phenyl-1,3- dodecanedione	8.310	274.1933	P
269	6-Oxocineole	8.346	168.1152	P
270	Pipericine	8.350	335.3182	P
271	Uscharidin	8.351	530.2499	P
272	Methyl 2-octynoate	8.359	154.0994	P
273	4-Vinylcyclohexene	8.361	108.0938	P
274	2-Decylfuran	8.369	208.1829	P
275	Triphenyl phosphate	8.408	326.0707	P
276	Red chlorophyll catabolite	8.412	626.2767	P
277	Carpaine	8.457	478.3777	P
278	Dicyclomine	8.467	309.2668	P
279	12*S*-HEPE	8.510	318.2197	P
280	3L,7D,11D-phytanic acid	8.510	312.3030	P
281	*N*-(14-Methylhexadecanoyl)pyrrolidine	8.520	323.3187	P
282	Polidocanol	8.520	582.4343	P
283	Mycinamicin VIII	8.522	505.3388	P
284	8,8-Diethoxy-2,6-dimethyl-2-octanol	8.541	246.2196	P
285	(3a,5b,7a,12a)-24-[(carboxymethyl)amino]-1,12-dihydroxy-24-oxocholan-3-yl-b-Dglucopyranosiduronic a	8.544	641.3409	P
286	Protoprimulagenin A 3-[rhamnosyl-(1->4)-rhamnosyl-(1->4)-[rhamnosyl-(1->2)]-glucosyl-(1->?)-glucuronide]	8.544	1234.6276	P
287	Oleyl alcohol	8.555	268.2768	P
288	Vaccenyl carnitine	8.602	425.3509	P
289	Polysorbate 20	8.616	522.3407	P
290	Stearoylethanolamide	8.684	327.3138	P
291	Lucidenic acid K	8.766	472.2437	P
292	Polysorbate 60	8.767	434.2882	P

293	Hexyl heptanoate	8.787	638.2366	P
294	2,2,7,7-Tetramethyl-1,6-dioxaspiro[4.4]non-3-ene	8.827	182.1304	P
295	Tecostanine	8.827	183.1625	P
296	9-Acetoxyfukinanolide	8.865	292.1676	P
297	Gabapentin	8.879	171.1260	P
298	*N*-Methylpelletierine	8.880	155.1311	P
299	Guaioxide	8.915	222.1982	P
300	MG(0:0/20:1(11*Z*)/0:0)	8.920	384.3240	P
301	Tris(butoxyethyl)phosphate	8.925	398.2434	P
302	20,21,21-Trifluoro-3-methoxy-19-nor-17*alpha*-pregna-1,3,5(10),20-tetraen-17-ol	8.942	366.1812	P
303	Formebolone	8.989	344.1987	P
304	Phytal	8.989	294.2920	P
305	Isoacitretin	9.037	326.1882	P
306	(*E,E*)-1,6-bis(4-methoxyphenyl)-1,5-hexadiene	9.039	294.1622	P
307	Annoglabasin F	9.077	378.2404	P
308	*Alpha*-CEHC	9.100	278.1519	P
309	Anofinic acid	9.108	204.0786	P
310	22-Oxo-docosanoate	9.130	354.3137	P
311	(*E*)-1-[4-Hydroxy-3-(3-methyl-1,3-butadienyl)phenyl]-2-(3,5-dihydroxyphenyl)ethylene	9.162	294.1254	P
312	MG(0:0/22:2(13*Z*,16*Z*)/0:0)	9.165	410.3397	P
313	Armillarivin	9.185	384.1937	P
314	(6*beta*,7*alpha*,12*beta*,13*beta*)-7-Hydroxy-11,16- dioxo-8,14-apianadien- 22,6-olide	9.188	384.1929	P
315	18-Oxocortisol	9.204	376.1884	P
316	1-(3-Hydroxy-4-methoxyphenyl)-1,2-ethanediol	9.207	184.0734	P
317	Ampalex	9.207	241.1204	P
318	Misoprostol	9.207	382.2704	P
319	Tsangane L 3-glucoside	9.209	374.2309	P
320	β-Caryophyllene Alcohol	9.238	222.1985	P

321	Gentamicin	9.265	477.3144	P
322	Linoleoyl Ethanolamide	9.295	323.2826	P
323	10-Eicosene	9.326	280.3133	P
324	Bioresmethrin	9.368	338.1882	P
325	Chloropyramine	9.369	289.1356	P
326	Cyclopassifloside II	9.372	682.4269	P
327	MG(0:0/16:0/0:0)	9.376	330.2771	P
328	Acidissiminol epoxide	9.389	409.2251	P
329	MG(0:0/22:6(4*Z*,7*Z*,10*Z*,13*Z*,16*Z*,19*Z*)/0:0)	9.424	402.2755	P
330	(3b,6b,8b,12a)-8,12-Epoxy-7(11)- eremophilene-6- angeloyloxy-8,12- dimethoxy-3-ol	9.428	394.2354	P
331	Asebotoxin II	9.428	408.2502	P
332	Methandriol dipropionate	9.429	416.2912	P
333	[6]-Gingerdiol 3,5- diacetate	9.431	380.2203	P
334	Lilac alcohol	9.433	170.1306	P
335	Calendulaglycoside E	9.434	794.4397	P
336	Iriomoteolide 1a	9.450	506.3221	P
337	3-(5,6,6-Trimethylbicyclo[2.2.1]h ept-1-yl)cyclohexanol	9.454	236.2142	P
338	Lucidenic acid M	9.456	462.2959	P
339	MG(0:0/18:3(6*Z*,9*Z*,12*Z*)/0:0)	9.461	352.2614	P
340	Allixin	9.465	226.1209	P
341	Galbanic acid	9.494	398.2094	P
342	Heliosupine	9.570	397.2113	P
343	2-(4-Chloro-3,5-dimethylphenoxy)-*N*-(2-phenyl-2H-benzotriazol-5-yl)-acetamide	9.641	406.1195	P
344	Monocrotaline	9.642	325.1526	P
345	4*beta*-(2-Aminoethylthio)catechin	9.643	365.0923	P
346	Oleoyl Ethanolamide	9.789	325.2983	P
347	Nitramine	9.813	169.1469	P
348	Tropine	9.817	141.1153	P

349	MG(0:0/22:1(13*Z*)/0:0)	9.833	412.3553	P
350	1b,3a,7a,12a-Tetrahydroxy-5b cholanoic acid	9.940	424.2813	P
351	2,5-Furandicarboxylic acid	9.942	156.0061	P
352	Acetyl tributyl citrate	9.942	402.2256	P
353	Arbutin	9.942	272.0898	P
354	4-Carboxy-2-hydroxy-6-methoxy-6-oxohexa-2,4-dienoate	9.943	216.0271	P
355	Kamahine C	9.944	268.1315	P
356	Vanillactic acid	9.944	212.0686	P
357	(*Z*)-9-Cycloheptadecen-1-one	9.981	250.2297	P
358	Isopimara-7,15-dienol	10.156	288.2454	P
359	Balofloxacin	10.185	389.1759	P
360	Hellebrin	10.192	724.3298	P
361	DU 122290	10.200	362.1652	P
362	1,1'-(1,4-Dihydro-4-nonyl-3,5-pyridinediyl)bis[1-decanone]	10.224	515.4681	P
363	Mycinamicin III	10.231	681.4103	P
364	(1*alph*a,3*beta*,20*S*,22*R*,24*S*,25*S*)-Pubescenin	10.289	620.3558	P
365	Oleandrin	10.352	576.3298	P
366	Drotaverine	10.381	397.2252	P
367	Drospirenone	10.392	366.2196	P
368	Arachidonyl carnitine	10.412	504.4028	P
369	Ganoderic acid I	10.419	532.3038	P
370	Dodecylbenzene	10.488	246.2349	P
371	Oleamide	10.489	281.2721	P
372	(±)-(*Z*)-2-(5-Tetradecenyl)cyclobutanone	10.490	264.2454	P
373	PE(22:0/24:0)	10.490	887.7355	P
374	DG(15:0/20:1(11*Z*)/0:0)	10.491	608.5355	P
375	DG(18:1(11*Z*)/22:5(4*Z*,7*Z*,10*Z*,13*Z*,16*Z*)/0:0)	10.551	668.5400	P
376	DG(20:3(5*Z*,8*Z*,11*Z*)/22:6(4*Z*,7*Z*,10*Z*,13*Z*,16*Z*,19*Z*)/0:0)	10.551	690.5221	P
377	Capsi-amide	10.583	269.2719	P

378	4-Nerolidylcatechol	10.597	314.2241	P
379	(3S,6E,10E)-1,6,10,14-Phytatetraen-3-ol	10.738	290.2611	P
380	Dglucosyldihydrosphingosine	10.807	463.3513	P
381	Cavipetin D	10.894	418.2717	P
382	D-myo-Inositol-1,4,5-triphosphate	10.894	419.9628	P
383	Spiramycin	10.894	840.5340	P
384	Vinaginsenoside R1	10.895	842.4946	P
385	Sorbitan palmitate	10.896	402.2985	P
386	Eremopetasinorol	11.012	208.1460	P
387	Ganodermic acid TQ	11.111	510.3346	P
388	Ganoderic acid V	11.112	528.3453	P
389	Stearamide	11.120	283.2876	P
390	Linalyl propionate	11.158	210.1617	P
391	Riesling acetal	11.159	226.1571	P
392	*N*-hexadecanoylpyrrolidine	11.386	309.3033	P
393	Coriandrone E	11.504	248.0686	P
394	DG(20:5(5*Z*,8*Z*,11*Z*,14*Z*,17*Z*)/24:1(15*Z*)/0:0)	11.507	724.6029	P
395	MG(18:0/0:0/0:0)	11.508	358.3084	P
396	Tridemorph	11.668	297.3028	P
397	TG(18:1(9*Z*)/18:1(9*Z*)/18:1(9*Z*))	11.962	884.7831	P
398	7-Oxostigmasterol	12.255	426.3487	P
399	PC(14:0/22:5(4*Z*,7*Z*,10*Z*,13*Z*,16*Z*))	12.255	780.5544	P
400	12-Ketodeoxycholic acid	12.259	390.2772	P
401	PC(16:0/18:1(9*Z*))[*S*]	12.270	760.5854	P
402	Dioctyl hexanedioate	12.282	370.3081	P
403	Testosterone isocaproate	12.284	386.2814	P
404	2-Aminoethylphosphocholate	13.717	515.3046	P
405	DG(14:0/22:1(13*Z*)/0:0)	14.164	622.5545	P
406	4a-Methyl-5a-cholesta-8,24-dien-3-one	14.188	396.3393	P

407	7-Dehydrocholesterol	14.213	384.3396	P

*-acetonitrile:water (1;1, *v/v*)

[a] – retention time [min]

[b] –compound detection in positive (P) or in negative (N) ionization mode.
