# Supplementary material for: Biological Properties of the Mucus and Eggs of Helix aspersa Müller as a Potential Cosmetic and Pharmaceutical Raw Material: A Preliminary Study
Source: Int J Mol Sci. 2024 Sep 15;25(18):9958. doi: 10.3390/ijms25189958 (PMC11432642; doi:10.3390/ijms25189958)
Supplement: Supplementary file 1 [file ijms-25-09958-s001.zip › Herman Anna - Table S6.pdf]

Table S6. Compounds identified in water extract of lyophilized egg of organic *Helix aspersa* snail using LC-MS.

No	Metabolite	RT[a] [min]	Mass [*m/z*]	Detection mode[b]
1	Dulcitol	0.257	182.0791	N
2	Dimethyl carbonate	0.262	90.0317	N
3	3-b-Galactopyranosylglucose	0.275	342.1162	N
4	Trifluoroacetic acid	0.354	113.9929	N
5	Dexpanthenol	0.879	205.1316	N
6	3,5-Dinitroguaiacol	4.672	214.0227	N
7	D-Ribose 1-diphosphate	5.617	293.9899	N
8	Blumenol C glucoside	5.726	372.2148	N
9	Ethiprole	5.806	395.9834	N
10	Eremopetasinorol	6.773	208.1464	N
11	3-Hydroxy-6,8-dimethoxy-7(11)-eremophilen-12,8-olide	7.034	310.1779	N
12	BILA 2185BS	7.040	618.3252	N
13	Methyl 2-benzamidoacetate	7.057	193.0735	N
14	(*S,Z*)-Lyratol acetate	7.114	194.1306	N
15	Lauryl hydrogen sulfate	7.281	266.1553	N
16	Losartan	7.315	422.1621	N
17	Methotrexate	7.316	454.1732	N
18	L-Tyrosine methyl ester	7.344	195.0896	N
19	*N*-Methyl-14-Odemethylepiporphyroxine	7.460	371.1372	N
20	Dinoterb	7.581	240.0750	N
21	*N*-Undecylbenzenesulfonic acid	7.725	312.1760	N
22	2-Dodecylbenzenesulfonic acid	8.163	326.1916	N
23	Sodium Tetradecyl Sulfate	8.202	294.1864	N
24	Kukoamine D	8.407	530.3124	N
25	Alcaftadine	8.885	307.1686	N
26	Gemfibrozil	8.960	250.1569	N

27	Furmecyclox	9.281	251.1523	N
28	3-Oxochola-4,6-dien-24-oic acid	10.250	370.2505	N
29	(5b,7a,12a)-2-(3-methoxyphenyl)-2-oxoethyl ester-7,12-dihydroxy-cholan-24-oic acid	10.297	540.3447	N
30	Enalkiren	10.848	656.4283	N
31	Butroxydim	11.272	399.2409	N
32	Adlupone	11.366	482.3392	N
1	Trichlopyr	0.238	254.9260	P
2	L-Homocysteic acid	0.240	183.0210	P
3	Dulcitol	0.257	182.0789	P
4	D-1-Aminopropan-2-ol *O*-phosphate	0.258	155.0350	P
5	Dihydrocaffeic acid 3-*O*- glucuronide	0.258	358.0897	P
6	2-Amino-2-methyl-1,3- propanediol	0.259	105.0789	P
7	3-Hydroxyisoheptanoic acid	0.260	146.0943	P
8	Choline chloride	0.264	103.0997	P
9	3-Amino-2-piperidone	0.266	114.0795	P
10	Tetrahydrofurfuryl acetate	0.270	144.0785	P
11	Isoamyl nitrite	0.273	117.0790	P
12	4,5-Dihydroxyhexanoic acid lactone	0.274	130.0629	P
13	3-b-Galactopyranosyl glucose	0.276	342.1165	P
14	3-Guanidinopropanoate	0.277	131.0696	P
15	*N*-nitrosomethanamine	0.277	60.0321	P
16	Porric acid A	0.277	302.0787	P
17	3-(*N*-Nitrosomethylamino)propionitrile	0.278	113.0589	P
18	4-Amino-2- methylenebutanoic acid	0.279	115.0633	P
19	2-hydroxy-decanedioic acid	0.284	218.1156	P
20	1-Deoxy-D-glucitol	0.303	166.0842	P
21	Octanoic acid, 7- hydroxy-, (*S*)-	0.312	160.1100	P
22	1-nitroheptane	0.315	145.1104	P
23	4-Guanidinobutanoic acid	0.316	145.0851	P

24	Nicotinamide *N*-oxide	0.32	138.0431	P
25	Phenethicillin	0.353	364.1099	P
26	8-Hydroxypurine	0.359	138.0547	P
27	Purine	0.360	120.0436	P
28	*N*-Nitrosopyrrolidine	0.366	100.0637	P
29	*Beta*-Alaninamide	0.374	88.0633	P
30	(*S*)-Piperazine-2- carboxamide	0.376	129.0898	P
31	L-Pipecolic acid	0.411	129.0790	P
32	(2*R*,3*R*,4*R*)-2-Amino-4-hydroxy-3- methylpentanoic acid	0.412	147.0896	P
33	Trolamine	0.413	149.1053	P
34	L-2-Amino-5- hydroxypentanoic acid	0.438	133.0740	P
35	Methyl propionate	0.503	88.0524	P
36	Polypropylene glycol (m w 1,200-3,000)	0.871	134.0943	P
37	(3-Phenylpropionyl)glycine methyl ester	0.877	221.1052	P
38	Dexpanthenol	0.879	205.1315	P
39	Isoleucyl-Threonine	0.898	232.1425	P
40	2,5-Dihydro-2,4,5- trimethyloxazole	1.535	113.0840	P
41	2,3-Butanediol glucoside	1.575	252.1212	P
42	*γ*-Aminobutyryl-lysine	2.466	231.1584	P
43	Octylamine	2.601	129.1518	P
44	Homoarecoline	3.473	169.1104	P
45	*N*-n-Hexanoylglycine methyl ester	3.473	187.1210	P
46	Istamycin C1	3.590	431.2733	P
47	Gabapentin	3.617	171.1260	P
48	Neryl glucoside	3.671	316.1885	P
49	Netilmicin	3.740	475.2993	P
50	Solanocapsine	3.784	430.3545	P
51	Dicyclohexylamine	4.028	181.1830	P
52	1,11-Undecanedicarboxylic acid	4.191	244.1672	P

53	PE(18:4(6*Z*,9*Z*,12*Z*,15*Z*)/22:6(4*Z*,7*Z*,10*Z*,13*Z*,16*Z*,19*Z*))	4.452	783.4831	P
54	1-Octen-3-yl glucoside	4.500	290.1730	P
55	*N*-[(Ethoxycarbonyl)methyl)-p-menthane-3- carboxamide	4.506	269.1991	P
56	Halstoctacosanolide A	4.528	844.5362	P
57	(*E*)-3-decen-1-ol	4.553	156.1514	P
58	Ethyl 3-(*N*- butylacetamido)propionate	4.653	215.1523	P
59	1,2,3-Tris(1-ethoxyethoxy)propane	4.674	308.2200	P
60	C12:1n-7	4.692	198.1621	P
61	Ruscopine	4.705	306.2043	P
62	2-Ethylacrylylcarnitine	4.734	244.1549	P
63	2-Phenylbutyric acid	4.736	164.0842	P
64	Methyl 3-(2,3-dihydroxy- 3-methylbutyl)-4- hydroxybenzoate	4.818	254.1156	P
65	Ganglioside GA1 (d18:1/16:0)	4.836	1226.7561	P
66	4'-Hydroxy-3,4,5-trimethoxystilbene	4.966	286.1210	P
67	1,1,2-Triphenylpropane	5.023	272.1555	P
68	Sterebin E	5.080	338.2457	P
69	(*S*)-3-Octanol glucoside	5.099	292.1892	P
70	Gibberellin A105	5.138	330.1467	P
71	(-)-*trans*-Carveol glucoside	5.139	314.1733	P
72	Ganglioside GM3 (d18:0/18:1(11*Z*))	5.258	1180.7503	P
73	Cyclonormammein	5.275	374.1725	P
74	Toxin T2 tetrol	5.310	298.1418	P
75	Triethyl citrate	5.409	276.1209	P
76	Jasmolone glucoside	5.432	342.1680	P
77	11-Hydroxy-9-tridecenoic acid	5.479	228.1724	P
78	Bis-*N*-butyl phthalate	5.483	467.1800	P
79	Corchoionol C 9-glucoside	5.539	386.1942	P
80	Eremopetasinorol	5.657	208.1465	P
81	Hexanal octane-1,3-diol acetal	5.709	228.2090	P

82	(5*alpha*,10*alpha*)-3,7(11)-Eudesmadien-2-one	5.770	218.1671	P
83	Avocadienofuran	5.771	246.1985	P
84	Blumenol C *O*-[rhamnosyl-(1->6)-glucoside]	5.786	518.2725	P
85	2,2,4,4,-Tetramethyl-6-(1-oxopropyl)-1,3,5-cyclohexanetrione	5.819	238.1206	P
86	Glaucamine	5.860	385.1526	P
87	Sanshodiol	5.861	358.1418	P
88	Cinegalline	5.919	430.2107	P
89	*N*-Acetyl-2,6-diethylaniline	5.958	191.1313	P
90	Homodihydrojasmone	6.070	180.1515	P
91	2-Hydroxyestrone	6.080	286.1570	P
92	Lupinine	6.088	169.1468	P
93	(+/-)-[*R*-(*E*)]-5-Isopropyl-8-methylnona-6,8-dien-2-one	6.140	194.1671	P
94	4-Hydroxy-3-methoxy-2,10-bisaboladien-9-one	6.212	266.1881	P
95	*alpha*-Butyl-*omega*hydroxypoly(oxyethylene) poly(oxypropylene)	6.273	248.1989	P
96	Chalciporone	6.286	243.1619	P
97	Gravelliferone	6.307	298.1567	P
98	Eucalyptol	6.336	154.1358	P
99	(+)-Prosopinine	6.345	313.2618	P
100	1,1-Diethoxy-2-hexene	6.356	172.1464	P
101	Cuscohygrine	6.374	224.1890	P
102	Momilactone B	6.567	330.1833	P
103	Lilac alcohol	6.612	170.1306	P
104	Canavalioside	6.642	546.2681	P
105	Monoisobutyl phthalic acid	6.666	222.0893	P
106	10-Hydroxy-2,8-decadiene-4,6-diynoic acid	6.667	176.0473	P
107	C16 Sphinganine	6.687	273.2667	P
108	Isorenieratene/(Leprotene)	6.768	528.3774	P
109	Erysothiopine	6.785	407.1026	P
110	Porson	6.785	386.1729	P

111	16-hydroxy hexadecanoic acid	6.795	272.2353	P
112	Funtumine	6.848	317.2718	P
113	5-Dodecyldihydro-2(3H)-furanone	6.883	254.2247	P
114	Diclomezine	6.944	254.0003	P
115	7-(4-Hydroxy-3-methoxyphenyl)-5-methoxy-1-phenyl-3-heptanone	6.969	342.1833	P
116	Muricatacin	6.993	284.2352	P
117	Nonyl octanoate	6.993	270.2559	P
118	Lauroyl diethanolamide	7.011	287.2464	P
119	Acetyl Tyrosine Ethyl Ester	7.030	251.1160	P
120	Palmitic amide	7.035	255.2563	P
121	BILA 2185BS	7.043	618.3259	P
122	Cyclotetradecane	7.071	196.2188	P
123	Terbucarb	7.092	277.2041	P
124	Finaconitine	7.125	630.3149	P
125	2-Tetradecanone	7.189	212.2141	P
126	10,16-dihydroxy-palmitic acid	7.193	288.2302	P
127	Armillaric acid	7.243	416.1835	P
128	Cincassiol B	7.243	400.2101	P
129	Bleekerine	7.318	409.1755	P
130	Testolactone	7.342	300.1724	P
131	Allopumiliotoxin 267A	7.385	267.2203	P
132	Nervonoylacetone	7.399	406.3798	P
133	Physagulin C	7.439	542.2505	P
134	2,2-Dimethyl-3,4-bis(4- methoxyphenyl)-2H-1- benzopyran-7-ol acetate	7.461	430.1783	P
135	Armillaripin	7.462	414.2044	P
136	Vilazodone	7.463	441.2149	P
137	Methyl (9*Z*)-10'-oxo-6,10'-diapo-6- carotenoate	7.500	312.1726	P
138	(*E,E*)-1,6-bis(4-methoxyphenyl)-1,5- hexadiene	7.501	294.1618	P
139	C14:1n-9	7.586	226.1933	P

140	Pristanic acid	7.668	298.2874	P
141	8-Acetoxypinoresinol 4- glucoside	7.777	578.2024	P
142	Glycosides	7.778	584.2839	P
143	Ethyl menthane carboxamide	7.831	211.1938	P
144	(*E*)-3-(2-Hydroxyphenyl)- 2-propenal	7.838	148.0524	P
145	Glaudine	7.838	399.1684	P
146	5-Hexyltetrahydro-2-furanoctanoic acid	7.913	298.2507	P
147	(10*S*)-Juvenile hormone III diol	7.960	284.1988	P
148	Dodecanamide	7.961	199.1938	P
149	Stearamide	8.015	283.2877	P
150	MG(0:0/18:1(11*Z*)/0:0)	8.020	356.2928	P
151	Lymecycline	8.142	602.2576	P
152	Undecylprodigiosin	8.167	393.2786	P
153	2-oxophytanic acid	8.204	326.2819	P
154	Erinacine G	8.267	464.2416	P
155	Hydrocortisone cypionate	8.267	486.2960	P
156	Lyngbyatoxin	8.268	437.3044	P
157	Tributyl phosphate	8.305	266.1647	P
158	Palonosetron	8.329	296.1894	P
159	(*S*)-Nerolidol 3-O-[a-L- rhamnopyranosyl-(1->2)-b-D-glucopyranoside]	8.357	530.3068	P
160	Flabellidine	8.381	288.2204	P
161	Kukoamine D	8.399	530.3123	P
162	B 823-08	8.408	353.0821	P
163	Dimethyl 4,4-o- Phenylene-Bis (3- Thiophanate)	8.409	342.0448	P
164	Triphenyl phosphate	8.409	326.0709	P
165	MK-129	8.411	367.0976	P
166	3L,7D,11D-phytanic acid	8.518	312.3028	P
167	*N*-(14-Methylhexadecanoyl)pyrrolidine	8.527	323.3189	P
168	Dodemorph	8.538	281.2718	P

169	8,8-Diethoxy-2,6-dimethyl-2-octanol	8.544	246.2194	P
170	Palmitoyl glucuronide	8.586	418.2930	P
171	Polysorbate 20	8.616	522.3405	P
172	Palmitoyl-EA	8.628	299.2826	P
173	Isopimara-7,15-dienol	8.681	288.2446	P
174	LysoPC(14:0)	8.684	468.3090	P
175	Sorbitan palmitate	8.687	402.2980	P
176	Polysorbate 60	8.766	434.2884	P
177	TG(8:0/8:0/8:0)	8.766	470.3593	P
178	Laserpitin	8.767	450.2619	P
179	Hexyl heptanoate	8.788	478.3259	P
180	Austrobailignan 7	8.833	342.1471	P
181	9-Acetoxyfukinanolide	8.869	292.1675	P
182	Spiroxamine	8.879	297.2666	P
183	Tris(butoxyethyl)phosphate	8.925	398.2437	P
184	MG(0:0/20:1(11*Z*)/0:0)	8.926	384.3242	P
185	Colforsin	8.962	410.2300	P
186	Phytal	8.994	294.2923	P
187	3-Cyclohexyldodecane	9.011	252.2818	P
188	10-hydroperoxy-8*E*,12*Z* octadecadienoic acid	9.014	312.2305	P
189	Isoacitretin	9.036	326.1883	P
190	24-Hydroxycalcitriol	9.092	432.3241	P
191	Anofinic acid	9.107	204.0786	P
192	*Alpha*-CEHC	9.109	278.1519	P
193	Chlordiazepoxide	9.113	299.0816	P
194	22-Oxo-docosanoate	9.136	354.3135	P
195	(*Z*)-13-Oxo-9-octadecenoic acid	9.145	296.2350	P
196	Isohumulinone A	9.199	378.2042	P
197	Epivoacorine	9.203	720.3873	P

198	18-Oxocortisol	9.204	376.1885	P
199	Misoprostol	9.204	382.2720	P
200	1-(3-Hydroxy-4- methoxyphenyl)-1,2- ethanediol	9.205	184.0736	P
201	Marshdimerin	9.205	758.3851	P
202	Tsangane L 3-glucoside	9.205	374.2304	P
203	13,14-dihydro-15-keto-PGF2α	9.286	354.2409	P
204	Piscerythramine	9.287	451.2012	P
205	10-Eicosene	9.329	280.3131	P
206	Pravastatin	9.362	424.2461	P
207	Bioresmethrin	9.367	338.1881	P
208	Chloropyramine	9.367	289.1357	P
209	Azulene	9.368	128.0626	P
210	MG(0:0/16:0/0:0)	9.369	330.2771	P
211	MG(0:0/22:6(4*Z*,7*Z*,10*Z*,13*Z*,16*Z*,19*Z*)/0:0)	9.407	402.2753	P
212	Asebotoxin II	9.408	408.2501	P
213	[6]-Gingerdiol 3,5- diacetate	9.424	380.2203	P
214	Aripiprazole	9.430	447.1460	P
215	3'-Galloylprodelphinidin B2	9.431	762.1412	P
216	6-Hydroxykaempferol 3,6-diglucoside 7-glucuronide	9.431	802.1799	P
217	Virginiamycin S1	9.432	823.3525	P
218	*S*-Adenosylmethionine	9.433	399.1451	P
219	Loratadine	9.434	382.1440	P
220	(3'x,5'a,9'x,10'b)-O-(3-Hydroxy-6-oxo-7-drimen- 11-yl)umbelliferone	9.435	396.1937	P
221	Vinblastine	9.439	810.4217	P
222	Methandriol dipropionate	9.440	416.2912	P
223	MG(0:0/18:3(6*Z*,9*Z*,12*Z*)/0:0)	9.472	352.2616	P
224	Polidocanol	9.552	582.4344	P
225	17-O-Acetylnorajmaline	9.595	354.1962	P
226	2,3-Dinor-6-keto- prostaglandin F1 a	9.595	342.2042	P

227	Monocrotaline	9.641	325.1530	P
228	2-(4-Chloro-3,5- dimethylphenoxy)-*N*-(2- phenyl-2H-benzotriazol- 5-yl)-acetamide	9.642	406.1197	P
229	4*beta*-(2- Aminoethylthio)catechin	9.643	365.0926	P
230	6*alpha*-Fluoro-17*beta*- hydroxyandrost-4-en-3- one acetate	9.672	348.2112	P
231	Glycidyl oleate	9.696	338.2820	P
232	Lycopersiconol	9.766	334.2505	P
233	6,8a-Seco-6,8a-deoxy-5- oxoavermectin "2a" aglycone	9.817	586.3504	P
234	(3b,6b,8b,12a)-8,12-Epoxy-7(11)- eremophilene-6- angeloyloxy-8,12-dimethoxy-3-ol	9.836	394.2355	P
235	Kamahine C	9.941	268.1310	P
236	Acetyl tributyl citrate	9.942	402.2255	P
237	Ampalex	9.942	241.1204	P
238	Arbutin	9.942	272.0897	P
239	Asteltoxin	9.942	418.1984	P
240	Cymorcin monoglucoside	9.942	328.1518	P
241	Fenpyroximate	9.942	421.2017	P
242	Vanillactic acid	9.942	212.0686	P
243	1b,3a,7a,12a- Tetrahydroxy-5b-cholanoic acid	9.943	424.2811	P
244	2,5-Furandicarboxylic acid	9.943	156.0059	P
245	4-Carboxy-2-hydroxy-6-methoxy-6-oxohexa-2,4-dienoate	9.943	216.0271	P
246	MEGA	9.945	325.1780	P
247	Balofloxacin	10.198	389.1760	P
248	DU 122290	10.198	362.1652	P
249	Tamoxifen	10.198	371.2249	P
250	1-Methylpyrrolinium	10.199	84.0811	P
251	Drotaverine	10.379	397.2255	P
252	Panamine	10.393	315.2664	P
253	Petromyzonol	10.414	394.3083	P
254	Capsi-amide	10.641	269.2718	P
255	Drospirenone	10.682	366.2199	P

256	Enalkiren	10.847	656.4294	P
257	Cavipetin D	10.895	418.2721	P
258	D-myo-Inositol-1,4,5-triphosphate	10.895	419.9621	P
259	Propinol adenylate	10.896	403.0890	P
260	Camptothecin	11.237	348.1108	P
261	12-Ketodeoxycholic acid	12.259	390.2771	P
262	PC(16:0/18:1(9*Z*))[*S*]	12.269	760.5852	P
263	Dioctyl hexanedioate	12.279	370.3083	P
264	Testosterone isocaproate	12.282	386.2818	P
265	Gallocatechin-(4*alpha*->8)-gallocatechin-(4*alpha*->8)-gallocatechin	13.295	914.1881	P
266	Aromatized deshydroxy-C-1027 chromophore	14.906	829.2582	P
267	DG(14:0/24:1(15*Z*)/0:0)	15.689	650.5851	P

[a] – retention time [min]

[b] –compound detection in positive (P) or in negative (N) ionization mode.
