# Supplementary material for: Biological Properties of the Mucus and Eggs of Helix aspersa Müller as a Potential Cosmetic and Pharmaceutical Raw Material: A Preliminary Study
Source: Int J Mol Sci. 2024 Sep 15;25(18):9958. doi: 10.3390/ijms25189958 (PMC11432642; doi:10.3390/ijms25189958)
Supplement: Supplementary file 1 [file ijms-25-09958-s001.zip › Herman Anna - Table S7.pdf]

Table S7. Compounds identified in metanolic extract of lyophilized egg of organic *Helix aspersa* snail using LC-MS.

No	Metabolite	RT[a] [min]	Mass [*m/z*]	Detection mode[b]
1	Dulcitol	0.254	182.0794	N
2	3-Deoxyarabinohexonic acid	0.262	180.0635	N
3	3-b-Galactopyranosylglucose	0.267	342.1163	N
4	2-Acetyl-5,8-dihydroxy-3-methoxy-1,4-naphthoquinone	0.272	262.0487	N
5	2,4,6-Octatriynoic acid	0.274	132.0194	N
6	5-(4-Hydroxybut-1-ynyl)-2,2'-bithiophene	0.274	234.0169	N
7	Dimethyl carbonate	0.285	90.0316	N
8	2-Naphthalenethiol	0.286	160.0351	N
9	Nicotinamide *N*-oxide	0.371	138.0431	N
10	*N*-n-Hexanoylglycine methyl ester	3.463	187.121	N
11	D-Ribose 1-diphosphate	5.620	293.9905	N
12	Ethiprole	5.803	395.9833	N
13	Azobenzene	6.294	182.0844	N
14	Flupropanate	6.563	145.9992	N
15	Bismuth subsalicylate	6.709	361.9977	N
16	Eremopetasinorol	6.781	208.1465	N
17	Nordihydrocapsiate	6.834	294.1832	N
18	BILA 2185BS	7.041	618.3249	N
19	Methyl 2-benzamidoacetate	7.059	193.0735	N
20	(*S,Z*)-Lyratol acetate	7.116	194.1308	N
21	3b-Allotetrahydrocorticosterone	7.122	350.2454	N
22	(3b,6b,8b,12a)-8,12-Epoxy-7(11)-eremophilene-6-angeloyloxy-8,12-dimethoxy-3-ol	7.200	394.2355	N
23	Zanthodioline	7.281	305.1265	N
24	Lauryl hydrogen sulfate	7.282	266.1552	N
25	Amifloxacin	7.283	334.1426	N
26	Losartan	7.316	422.1624	N
27	Methotrexate	7.316	454.1732	N
28	Venoterpine	7.346	149.0841	N
29	Arnamiol	7.459	450.1806	N

30	Dinoterb	7.581	240.0748	N
31	*N*-Undecylbenzenesulfonic acid	7.726	312.1758	N
32	Ethyl 2-hydroxy-3-(3-indolyl)propanoateglucoside	7.785	395.1586	N
33	2-Dodecylbenzenesulfonic acid	8.161	326.1915	N
34	Sodium Tetradecyl Sulfate	8.202	294.1863	N
35	Dihomo-γ-linolenoyl-EA	8.225	349.2981	N
36	(+)-Prosopinine	8.275	313.2614	N
37	Docusate	8.340	422.234	N
38	Kukoamine D	8.409	530.3123	N
39	Gemfibrozil	8.961	250.1569	N
40	Furmecyclox	9.282	251.1524	N
41	3-Oxochola-4,6-dien-24-oic acid	10.250	370.2512	N
42	(5b,7a,12a)-2-(3-methoxyphenyl)-2-oxoethyl ester-7,12-dihydroxy-cholan-24-oic acid	10.296	540.3448	N
43	Oleamide	10.485	281.2719	N
44	Methyl tetradecanoate	10.499	242.2248	N
45	5-Dodecyldihydro-2(3H)-furanone	10.504	254.2247	N
46	(3*R*,2'*S*)-Myxol 2'-(2,4-di-*O*-methyl-α-L-fucoside)	10.551	758.5092	N
47	Schidigeragenin B	10.551	428.2938	N
48	DG(18:1(11*Z*)/22:5(4*Z*,7*Z*,10*Z*,13*Z*,16*Z*)/0:0)	10.552	668.54	N
49	DG(20:3(5*Z*,8*Z*,11*Z*)/22:6(4*Z*,7*Z*,10*Z*,13*Z*,16*Z*,19*Z*)/0:0)	10.553	690.5217	N
50	MG(0:0/16:0/0:0)	10.553	330.2769	N
51	Piritramide	10.555	430.2738	N
52	Enalkiren	10.843	656.4283	N
53	3-Hydroxy-2-(4-morpholinylmethyl)estra-1,3,5(10)-trien-17-one	10.950	369.2305	N
54	Butroxydim	11.270	399.2403	N
55	Adlupone	11.367	482.3397	N
56	Drotaverine	11.450	397.2253	N
57	3-Hydroxy-6,8-dimethoxy-7(11)-eremophilen-12,8-olide	11.505	310.1771	N
58	Pubesenolide	11.505	458.305	N
59	DG(20:5(5*Z*,8*Z*,11*Z*,14*Z*,17*Z*)/24:1(15*Z*)/0:0)	11.506	724.6027	N
60	Callystatin A	11.507	456.3251	N
61	MG(18:0/0:0/0:0)	11.507	358.3081	N

62	(22*E*,24*R*)-Stigmasta-4,22-diene-3,6-dione	11.961	424.3338	N
63	5-Heptadecyl-1,3-benzenediol	11.996	348.303	N
64	Momordicinin	12.360	438.3502	N
65	(3*beta*,22*E*,24*R*)-3-Hydroxyergosta-5,8,22-trien-7-one	12.504	410.3181	N
1	Bufexamac	0.271	223.121	P
2	Trolamine	0.397	149.1052	P
3	2*E*-Decenedioic acid	2.364	200.1049	P
4	Amyl 2-furoate	2.475	182.0943	P
5	Propionyl-L-carnitine	2.477	218.1393	P
6	DL-2-amino-octanoic acid	2.560	159.1259	P
7	5-Heptyltetrahydro-2- oxo-3-furancarboxylic acid	2.952	228.136	P
8	Geranyl acetoacetate	3.006	238.1569	P
9	3-hydroxy- tetradecanedioic acid	3.032	274.1781	P
10	Sedanonic acid	3.122	210.1256	P
11	Capryloylglycine	3.141	201.1364	P
12	Wine lactone	3.292	166.0995	P
13	Homoarecoline	3.463	169.1105	P
14	*N*-n-Hexanoylglycine methyl ester	3.463	187.121	P
15	Istamycin C1	3.583	431.2733	P
16	1,2,3,4,5,6-Hexahydro-5- methyl-7H- cyclopenta[b]pyridin-7- one	3.649	151.0997	P
17	Phlorin	3.703	288.0846	P
18	Threo-Syringoylglycerol	3.706	244.0947	P
19	Netilmicin	3.734	475.2994	P
20	Monomenthyl succinate	3.849	256.1677	P
21	Arginyl-Isoleucine	3.866	287.1967	P
22	2-Methyl-1-phenyl-2- propanyl acetate	3.921	192.1149	P
23	*N*-(3-oxo-octanoyl)- homoserine lactone	3.983	241.1313	P
24	Tributyrin	4.023	302.1731	P
25	Isopentenyladenine-9-*N*- glucoside	4.039	363.1916	P

26	Jasmine ketolactone	4.254	208.1101	P
27	*N*-Methylmescaline	4.256	225.1367	P
28	*S*-(2-Methylbutanoyl)- dihydrolipoamide	4.363	291.1337	P
29	Mukaadial	4.384	266.152	P
30	Oseltamivir	4.387	312.205	P
31	Octyl gallate	4.399	282.1466	P
32	PE(18:4(6*Z*,9*Z*,12*Z*,15*Z*)/22:6(4*Z*,7*Z*,10*Z*,13*Z*,16*Z*,19*Z*))	4.450	783.4832	P
33	1-Octen-3-yl glucoside	4.497	290.1729	P
34	Halstoctacosanolide A	4.524	844.5361	P
35	(*E*)-3-decen-1-ol	4.549	156.1513	P
36	Diethofencarb	4.552	267.147	P
37	Ethyl decanoate	4.552	200.1776	P
38	Flumetover	4.552	367.1395	P
39	Imiquimod	4.634	240.1364	P
40	Ethyl 3-(*N*butylacetamido)propionate	4.647	215.1522	P
41	2-Hexenoylcholine	4.672	200.1653	P
42	1,2,3-Tris(1-ethoxyethoxy)propane	4.674	308.22	P
43	2,2,7,7-Tetramethyl-1,6-dioxaspiro[4,4]nona-3,8-diene	4.678	180.1151	P
44	C12:1n-7	4.684	198.1621	P
45	*Gamma*-CEHC	4.685	248.1411	P
46	Humulinic acid A	4.686	266.1517	P
47	11-Hydroxy-9-tridecenoic acid	4.694	228.1726	P
48	Ruscopine	4.700	306.2045	P
49	Ethyl 7-epi-12- hydroxyjasmonate glucoside	4.710	416.2049	P
50	Methyl 7-epi-12-hydroxyjasmonate glucoside	4.720	402.1889	P
51	2-Phenylbutyric acid	4.730	164.0838	P
52	1-Phenyl-6,7-dihydroxyisochroman	4.731	242.0947	P
53	2,3-dihydrobenzofuran	4.731	120.0576	P
54	2-Ethylacrylylcarnitine	4.731	244.1552	P

55	Alanyl-Isoleucine	4.779	202.1319	P
56	*N*-Isobutyl-2,4,8,10,12-tetradecapentaenamide	4.804	273.2094	P
57	Methyl 3-(2,3-dihydroxy-3-methylbutyl)-4-hydroxybenzoate	4.812	254.1156	P
58	Pinidine	4.848	139.1362	P
59	Epothilone B	4.899	507.2677	P
60	Methyl propionate	4.961	88.0523	P
61	1,3-Diphenyltetramethyldisiloxane	4.962	286.1209	P
62	2-Phenylethyl beta-Dglucopyranoside	5.010	284.1262	P
63	1,1,2-Triphenylpropane	5.020	272.1559	P
64	Pentosidine	5.037	378.2013	P
65	Cinncassiol A 19- glucoside	5.042	544.2514	P
66	Sterebin E	5.077	338.2457	P
67	*Z*-Arg-Arg-NHMec	5.078	621.3052	P
68	(*S*)-3-Octanol glucoside	5.099	292.1882	P
69	7,8-Dihydrovomifoliol 9- [rhamnosyl-(1->6)- glucoside]	5.104	534.2672	P
70	Gibberellin A105	5.135	330.1468	P
71	(-)-*trans*-Carveol glucoside	5.136	314.1731	P
72	Glycerol 1-(5- hydroxydodecanoate)	5.231	290.2097	P
73	Toxin T2 tetrol	5.243	298.1419	P
74	Cyclonormammein	5.274	374.1727	P
75	Elaeokanine C	5.288	211.1574	P
76	Jasmolone glucoside	5.370	342.168	P
77	Gravacridonetriol glucoside	5.412	519.1729	P
78	Valyl-Valine	5.427	216.1476	P
79	AF Toxin II	5.430	324.1574	P
80	Taraxacolide 1-*O*-b-D- glucopyranoside	5.445	428.2043	P
81	Hydrocortisone succinate	5.481	462.2249	P
82	Corchoionol C 9- glucoside	5.484	386.1942	P
83	*O*-Methylsomniferine	5.500	622.2659	P

84	(*E,E,E*)-*N*-(2-Methylpropyl)hexadeca- 2,6,8-trien-10-ynamide	5.507	301.2409	P
85	Isopulegone caffeate	5.535	316.1674	P
86	Satratoxin H	5.568	528.2336	P
87	Quinquenoside I	5.571	1014.57	P
88	Convallatoxin	5.644	550.2774	P
89	Terazosin	5.655	387.1892	P
90	Eremopetasinorol	5.656	208.1461	P
91	Blumenol C glucoside	5.688	372.2147	P
92	Hexanal octane-1,3-diol acetal	5.707	228.2089	P
93	Glaucarubin	5.714	496.2285	P
94	2-Methylundecanal	5.727	184.1827	P
95	Blumenol C *O*- [rhamnosyl-(1->6)- glucoside]	5.740	518.2728	P
96	2-Methoxyestradiol-3- methylether	5.760	316.2024	P
97	Avocadienofuran	5.769	246.1985	P
98	Volicitin	5.770	422.2766	P
99	(5*alpha*,10*alpha*)- 3,7(11)-Eudesmadien-2- one	5.771	218.1671	P
100	NAc-FnorLRF-amide	5.773	622.3567	P
101	Fluspirilene	5.809	475.242	P
102	2-Hydroxymyristic Acid	5.834	244.2038	P
103	19(*R*)-hydroxy-PGE2	5.847	368.2197	P
104	Glaucamine	5.859	385.153	P
105	Sanshodiol	5.859	358.142	P
106	C14:1n-9	5.878	226.1933	P
107	Eriojaposide A	5.879	502.2413	P
108	Canavalioside	5.942	546.2681	P
109	Sterebin B	6.016	352.2253	P
110	Capsoside A	6.017	694.3772	P
111	15-Acetoxyscirpene-3,4- diol 4-*O*-a-D- glucopyranoside	6.026	486.2102	P
112	Capsaicin	6.060	305.1991	P

113	Homodihydrojasmone	6.071	180.1515	P
114	Lauroyl diethanolamide	6.075	287.2462	P
115	20-COOH-Leukotriene B4	6.143	366.2041	P
116	2-Hydroxyestrone	6.146	286.157	P
117	(*Z*)-6-Nonenal	6.148	140.1203	P
118	Penbutolol	6.179	291.2199	P
119	10,11-Epoxy-3,7,11-trimethyl-2*E*,6*E*- tridecadienoic acid	6.211	266.1882	P
120	(+)-Prosopinine	6.230	313.2618	P
121	Chalciporone	6.280	243.1626	P
122	Gravelliferone	6.306	298.1571	P
123	Eucalyptol	6.335	154.1359	P
124	4-Methyl-4-aza-5- pregnene-3,20-dione	6.350	329.2352	P
125	1,1-Diethoxy-2-hexene	6.353	172.1465	P
126	*alpha*-Butyl-*omega*- hydroxypoly(oxyethylene) poly(oxypropylene)	6.356	248.1988	P
127	Cuscohygrine	6.373	224.1891	P
128	Dihydrocapsaicin	6.387	307.2152	P
129	1-Hydroxyacorenone	6.518	250.157	P
130	Armillatin	6.544	610.423	P
131	5-*O*-β-D-Mycaminosyltylonolide	6.566	597.3495	P
132	10-Hydroxy-2,8- decadiene-4,6-diynoic acid	6.665	176.0474	P
133	C16 Sphinganine	6.684	273.267	P
134	Sphinganine	6.684	301.2981	P
135	Artabsinolide A	6.690	280.1312	P
136	5,8-tetradecadienoic acid	6.695	224.1777	P
137	2-Furanmethanol	6.696	98.0368	P
138	p- Hydroxybenzyldesulphoglucosinolate	6.698	345.0869	P
139	17-Methylandrosta-2,4- dieno[2,3-d]isoxazol- 17*beta*-ol	6.706	327.2197	P
140	2,4,12-Octadecatrienoic acid isobutylamide	6.707	333.3017	P
141	Linopirdine	6.714	391.1689	P

142	5-(2,3-Dihydroxy-3- methylbutyl)-4-(3,4- epoxy-4- methylpentanoyl)-3,4- dihydroxy-2- isopentanoyl-2- cyclopenten-1-one	6.735	412.2102	P
143	1-Isomangostin hydrate	6.736	428.183	P
144	Cerberoside	6.736	858.426	P
145	Tigloidine	6.755	223.1572	P
146	Deacetylnomilin	6.756	472.21	P
147	1*alpha*,3*beta*,22*R*- Trihydroxyergosta-5,24*E*- dien-26-oic acid 3-*O*-b-D-glucoside 26-*O*-[b-D- glucosyl-(1->2)-b-D-glucosyl] ester	6.758	946.4849	P
148	2-Methoxy-estradiol-17β 3-glucuronide	6.758	478.2181	P
149	Ximelagatran	6.758	473.2628	P
150	Phytosphingosine	6.759	317.2932	P
151	Austalide A	6.780	516.2356	P
152	Trilobolide	6.780	522.2441	P
153	Mycalamide B	6.781	517.289	P
154	Erysothiopine	6.783	407.1025	P
155	Cinegalline	6.784	430.2106	P
156	Porson	6.784	386.1731	P
157	Panaquinquecol 1	6.787	292.2039	P
158	16-hydroxy hexadecanoic acid	6.792	272.2353	P
159	Canescein	6.803	566.2705	P
160	Funtumine	6.847	317.272	P
161	2-Pentadecanone	6.858	226.2297	P
162	Phenethyl decanoate	6.861	276.2091	P
163	5-Dodecyldihydro-2(3H)- furanone	6.884	254.2247	P
164	1-Methyl-2-nonyl-4(1H)- quinolinone	6.894	285.2092	P
165	Pumiliotoxin 251D	6.895	251.225	P
166	Genipin 1-*beta*-gentiobioside	6.901	550.1905	P
167	1-Tridecene	6.917	182.2035	P
168	Zizybeoside II	6.920	594.2165	P
169	Kanokoside C	6.938	638.242	P

170	Chrycolide	6.944	232.0185	P
171	7-(4-Hydroxy-3- methoxyphenyl)-5- methoxy-1-phenyl-3-heptanone	6.969	342.183	P
172	3'-Hydroxy-HT2 toxin	6.977	440.2048	P
173	Plantaricin BN	6.989	484.2311	P
174	Muricatacin	6.992	284.2353	P
175	Nonyl octanoate	6.993	270.2559	P
176	Coccinin	7.002	528.2567	P
177	2-Amino-7,8-dihydro-4- hydroxy-6 (diphosphooxymethyl)pteridine	7.018	355.0088	P
178	6-Caffeoylsucrose	7.023	504.148	P
179	(9*Z*,11*R*,12*S*,13*S*,15*Z*)-12,13-Epoxy-11-hydroxy- 9,15-octadecadienoic acid	7.025	310.2148	P
180	Acetyl Tyrosine Ethyl Ester	7.027	251.1161	P
181	Palmitic amide	7.034	255.2564	P
182	BILA 2185BS	7.043	618.326	P
183	Cyclotetradecane	7.063	196.2192	P
184	Xylopinine	7.083	355.1771	P
185	Myxochelin A	7.088	404.1582	P
186	Proansamitocin	7.094	443.2297	P
187	Terbucarb	7.094	277.204	P
188	Spiroxamine	7.100	297.2668	P
189	6*alpha*,9-Difluoro-11*beta*-hydroxypregn-4-ene-3,20-dione	7.108	366.202	P
190	Finaconitine	7.127	630.3152	P
191	2-Tetradecanone	7.185	212.2142	P
192	*Z*-Gly-Pro-Leu-Gly-Pro	7.188	573.2786	P
193	10,16-dihydroxy-palmitic acid	7.193	288.23	P
194	Armillaric acid	7.243	416.1833	P
195	Cincassiol B	7.243	400.21	P
196	Allopumiliotoxin 267A	7.251	267.2199	P
197	Marimastat	7.252	331.2118	P
198	Dodecylguanidine	7.265	227.2364	P

199	LysoPE(0:0/20:4(5*Z*,8*Z*,11*Z*,14*Z*))	7.298	501.2841	P
200	Bleekerine	7.318	409.1758	P
201	2,6-Di-tert-butyl-4-ethylphenol	7.366	234.1984	P
202	*N*-Dealkylatedtolterodine	7.379	283.1934	P
203	Nervonoylacetone	7.399	406.3796	P
204	1,8-Heptadecadiene-4,6-diyne-3,10-diol	7.401	260.1784	P
205	Armillaripin	7.404	414.2042	P
206	Digitalin	7.415	712.3654	P
207	Physagulin C	7.438	542.2504	P
208	Etiocholan-3α-ol-17-one 3-glucuronide	7.457	466.2562	P
209	Austalide L	7.461	428.22	P
210	Cyclocalopin F	7.461	294.1104	P
211	Picrasin C	7.461	422.2303	P
212	(4-Methylphenyl)acetaldehyde	7.462	134.0732	P
213	2,2-Dimethyl-3,4-bis(4-methoxyphenyl)-2H-1-benzopyran-7-ol acetate	7.462	430.1778	P
214	*Alpha*-Methylstyrene	7.462	118.0783	P
215	DHAP(18:0)	7.462	436.2599	P
216	Erythroskyrin	7.462	455.2308	P
217	Vilazodone	7.462	441.2155	P
218	Methyl (9*Z*)-10'-oxo-6,10'-diapo-6-carotenoate	7.503	312.1725	P
219	Norpropoxyphene	7.513	325.2042	P
220	Octadecanedioic acid	7.514	314.2459	P
221	7,10-Hexadecadienoic acid	7.569	252.209	P
222	2-Hexadecanone	7.644	240.2454	P
223	Biperiden	7.679	311.2256	P
224	3*beta*-Hydroxypregn-5-ene	7.705	302.2613	P
225	Austalide B	7.720	474.2254	P
226	Mammea E/BB	7.738	430.1995	P

227	1-(4-Amino-2-methylpyrimid-5-ylmethyl)-3-(*beta*hydroxyethyl)-2-methylpyridinium	7.750	259.155	P
228	Zucchini factor B	7.768	663.4308	P
229	6,10,14-Trimethyl- 5,9,13-pentadecatrien-2-one	7.776	262.2298	P
230	Glycosides	7.779	584.2848	P
231	p-Hydroxyphenethyl *trans*-ferulate	7.783	314.1154	P
232	Gabapentin	7.784	171.1261	P
233	Methyloctatropine	7.792	282.2434	P
234	Phlegmarine	7.837	250.2411	P
235	(*E*)-3-(2-Hydroxyphenyl)-2-propenal	7.839	148.0525	P
236	Glaudine	7.839	399.168	P
237	Estrane-3α,17α-diol	7.882	278.2247	P
238	Methadone	7.883	309.2091	P
239	Dihydrodioscorine	7.905	223.1573	P
240	5-Hexyltetrahydro-2- furanoctanoic acid	7.913	298.2511	P
241	9-HOTE	7.938	294.2197	P
242	Elaiophylin	7.944	1024.593	P
243	Dodecanamide	7.961	199.1936	P
244	Asparagoside D	7.965	902.4877	P
245	Scopoloside II	8.002	770.4095	P
246	2-Methoxyestrone 3- sulfate	8.014	380.1301	P
247	Stearamide	8.016	283.2879	P
248	Leucomycin A9	8.017	743.4097	P
249	MG(0:0/18:1(11*Z*)/0:0)	8.018	356.2929	P
250	Corchorusoside B	8.035	682.356	P
251	Pristanic acid	8.061	298.2869	P
252	Dihydro-5-(2-octenyl)- 2(3H)-furanone	8.119	196.1466	P
253	Lymecycline	8.143	602.2576	P
254	Undecylprodigiosin	8.167	393.2786	P

255	Dihydro-5-(2-octenyl)-2(3H)-furanone	8.208	625.3577	P
256	Hydrocortisone cypionate	8.269	486.2969	P
257	Lyngbyatoxin	8.270	437.3046	P
258	Erinacine G	8.271	464.2416	P
259	Pipericine	8.274	335.3173	P
260	17*beta*- Acetamidoandrost-4-en- 3-one	8.275	329.2353	P
261	2-(4-Methylphenyl)-2- propanol	8.276	150.1045	P
262	6-Oxocineole	8.277	168.115	P
263	Ethyl (4*Z*)-4,7-octadienoate	8.277	168.115	P
264	Pipercitine	8.277	349.3329	P
265	p-Mentha-1,3,5,8- tetraene	8.277	132.094	P
266	Santene	8.278	122.1096	P
267	MG(0:0/20:2(11*Z*,14*Z*)/0:0)	8.294	382.3083	P
268	Tributyl phosphate	8.307	266.1648	P
269	Ponasteroside A	8.308	626.3668	P
270	1-Phenyl-1,3- dodecanedione	8.314	274.1933	P
271	Palonosetron	8.331	296.189	P
272	Lentiginosine	8.343	157.1105	P
273	Isopentylideneisopentylamine	8.344	155.1675	P
274	1-Methyl-1,3- cyclohexadiene	8.346	94.0783	P
275	(*S*)-Nerolidol 3-*O*-[a-L- rhamnopyranosyl-(1->2)-b-D-glucopyranoside]	8.360	530.307	P
276	4-Vinylcyclohexene	8.361	108.0938	P
277	Isometheptene	8.362	141.1518	P
278	5-Methyl-2-phenyl-2- hexenal	8.384	188.1199	P
279	Flabellidine	8.387	288.2202	P
280	Mycinamicin VIII	8.394	505.3385	P
281	Kukoamine D	8.402	530.3118	P
282	Methyl 2*E*,4*Z*- hexadecadienoate	8.402	266.2246	P
283	Triphenyl phosphate	8.410	326.0708	P

284	MK-129	8.412	367.0974	P
285	SB 221284	8.412	353.0819	P
286	Methyprylon	8.450	183.1261	P
287	Methyl 2-octynoate	8.452	154.0994	P
288	Momilactone B	8.487	330.1831	P
289	12S-HEPE	8.508	318.2193	P
290	3L,7D,11D-phytanic acid	8.516	312.303	P
291	Polidocanol	8.517	582.4346	P
292	Linoleoyl Ethanolamide	8.524	323.2826	P
293	Methylhexadecanoyl)pyrrolidine	8.524	510.2944	P
294	*N*-(14-Methylhexadecanoyl)pyrrolidine	8.524	323.3192	P
295	Dodemorph	8.537	281.2721	P
296	Oleamide	8.537	281.2721	P
297	Palmitoyl-EA	8.538	299.2826	P
298	(3a,5b,7a,12a)-24-[(carboxymethyl)amino]- 1,12-dihydroxy-24- oxocholan-3-yl-b-D- Glucopyranosiduronic a	8.547	1244.543 7	P
299	8,8-Diethoxy-2,6- dimethyl-2-octanol	8.547	617.3419	P
300	Ergostan-3β-ol	8.551	767.4699	P
301	Oleyl alcohol	8.563	583.3932	P
302	Palmitoyl glucuronide	8.589	418.2933	P
303	Vaccenyl carnitine	8.608	425.3508	P
304	Polysorbate 20	8.618	522.3407	P
305	Isopimara-7,15-dienol	8.684	288.2451	P
306	Stearoylethanolamide	8.684	327.3135	P
307	LysoPC(14:0)	8.686	468.3087	P
308	Tecostanine	8.744	183.1623	P
309	Ikshusterol	8.754	430.3813	P
310	Polysorbate 60	8.769	434.2881	P
311	TG(8:0/8:0/8:0)	8.769	470.3596	P

312	Laserpitin	8.770	450.2615	P
313	2*E*-Eicosenoic acid	8.787	310.2871	P
314	Hexyl heptanoate	8.789	638.2364	P
315	2,2,7,7-Tetramethyl-1,6-dioxaspiro[4,4]non-3-ene	8.830	182.1307	P
316	Misoprostol	8.837	1484.768 9	P
317	Oleoyl Ethanolamide	8.849	325.2983	P
318	8, 11, 14, 17-icosatetraenoic acid; C20:4n-3,6,9,12	8.854	304.2397	P
319	9-Acetoxyfukinanolide	8.866	292.1672	P
320	13-heptadecyn-1-ol	8.883	252.2454	P
321	*N*-Methylpelletierine	8.883	155.131	P
322	*trans*-9, *trans*-11- octadecadienoic acid; C18:2n-7,9	8.883	280.2403	P
323	Austrobailignan 7	8.909	342.1468	P
324	β-Caryophyllene Alcohol	8.915	222.1985	P
325	MG(0:0/20:1(11*Z*)/0:0)	8.924	384.3244	P
326	Tris(butoxyethyl)phosphate	8.928	398.2437	P
327	Formebolone	8.944	344.1991	P
328	Colforsin	8.966	410.2311	P
329	Phytal	8.994	294.2922	P
330	3-Cyclohexyldodecane	9.014	252.2818	P
331	Annoglabasin F	9.018	378.2409	P
332	10-hydroperoxy-8*E*,12*Z*- octadecadienoic acid	9.032	312.2302	P
333	D-1-[(3-Carboxypropyl)amino]-1- deoxyfructose	9.032	265.1153	P
334	Isoacitretin	9.038	326.1884	P
335	(*E,E*)-1,6-bis(4-methoxyphenyl)-1,5- hexadiene	9.040	294.1623	P
336	24-Hydroxycalcitriol	9.089	432.3246	P
337	*Alpha*-CEHC	9.112	278.1519	P
338	Anofinic acid	9.115	204.0788	P
339	22-Oxo-docosanoate	9.134	354.3135	P

340	(*Z*)-13-Oxo-9-octadecenoic acid	9.145	296.2352	P
341	MG(0:0/22:2(13*Z*,16*Z*)/0:0)	9.169	410.3402	P
342	Philanthotoxin 343	9.179	435.3198	P
343	Armillarivin	9.183	384.1939	P
344	(6*beta*,7*alpha*,12*beta*,13*beta*)-7-Hydroxy-11,16- dioxo-8,14-apianadien- 22,6-olide	9.191	394.2695	P
345	Isohumulinone A	9.199	372.2879	P
346	18-Oxocortisol	9.206	258.1467	P
347	Methyl red	9.208	269.1151	P
348	1-(3-Hydroxy-4- methoxyphenyl)-1,2- ethanediol	9.209	184.0735	P
349	Marshdimerin	9.209	758.3847	P
350	PC(22:6(4*Z*,7*Z*,10*Z*,13*Z*,16*Z*,19*Z*)/22:6(4*Z*,7*Z*,10*Z*,13*Z*,16*Z*,19*Z*))	9.209	878.5728	P
351	Tsangane L 3-glucoside	9.209	374.2301	P
352	Gentamicin	9.270	477.3151	P
353	13,14-dihydro-15-keto- PGF2α	9.290	354.2404	P
354	Piscerythramine	9.291	451.2013	P
355	10-Eicosene	9.331	280.3133	P
356	Pravastatin	9.365	424.246	P
357	Bioresmethrin	9.369	338.1884	P
358	Chloropyramine	9.370	289.1359	P
359	MG(0:0/16:0/0:0)	9.373	330.2772	P
360	Lauroyl peroxide	9.378	398.3394	P
361	Acidissiminol epoxide	9.391	409.2252	P
362	MG(0:0/22:6(4*Z*,7*Z*,10*Z*,13*Z*,16*Z*,19*Z*)/0:0)	9.425	402.2754	P
363	(3'x,5'a,9'x,10'b)-*O*-(3-Hydroxy-6-oxo-7-drimen-11-yl)umbelliferone	9.429	396.1935	P
364	Aripiprazole	9.430	447.1463	P
365	Virginiamycin S1	9.431	823.3534	P
366	Lilac alcohol	9.434	170.1308	P
367	4-(Methylnitrosamino)-1-(3-pyridyl)-1-butanol glucuronide	9.435	385.147	P
368	Methandriol dipropionate	9.436	416.2911	P

369	MG(0:0/18:3(6*Z*,9*Z*,12*Z*)/0:0)	9.473	352.2614	P
370	5-*O*-Desmethyldonepezil	9.495	365.1994	P
371	Erinacine E	9.497	432.2514	P
372	Galbanic acid	9.498	398.2093	P
373	5,10-Pentadecadien-1-ol	9.509	224.2142	P
374	Heliosupine	9.566	397.2115	P
375	17-*O*-Acetylnorajmaline	9.588	354.1961	P
376	2,3-Dinor-6-ketoprostaglandin F1 a	9.598	342.2042	P
377	2-(4-Chloro-3,5-dimethylphenoxy)-*N*-(2-phenyl-2H-benzotriazol-5-yl)-acetamide	9.643	406.1195	P
378	Monocrotaline	9.644	325.1535	P
379	Monoisobutyl phthalic acid	9.644	222.0891	P
380	3-(5,6,6-Trimethylbicyclo[2,2,1]hept-1-yl)cyclohexanol	9.647	236.214	P
381	4*beta*-(2-Aminoethylthio)catechin	9.647	365.0925	P
382	HDOPA	9.676	376.2259	P
383	Gibberellin A110	9.692	348.1938	P
384	Glycidyl oleate	9.697	338.2818	P
385	Spirolide B	9.725	693.4596	P
386	Lycopersiconol	9.768	334.2503	P
387	Astemizole	9.779	458.2464	P
388	4-Hydroxyvalsartan	9.787	451.2224	P
389	MG(0:0/20:5(5*Z*,8*Z*,11*Z*,14*Z*,17*Z*)/0:0)	9.796	376.2614	P
390	6,8a-Seco-6,8a-deoxy-5-oxoavermectin "2a"aglycone	9.826	586.3508	P
391	MG(0:0/22:1(13*Z*)/0:0)	9.836	412.3555	P
392	1b,3a,7a,12a-Tetrahydroxy-5bcholanoic acid	9.942	424.281	P
393	Asteltoxin	9.942	418.1985	P
394	2,5-Furandicarboxylic acid	9.943	156.0058	P
395	4-Carboxy-2-hydroxy-6-methoxy-6-oxohexa-2,4-dienoate	9.943	216.027	P
396	Acetyl tributyl citrate	9.943	402.2256	P

397	Kamahine C	9.943	268.1311	P
398	Arbutin	9.944	272.0898	P
399	Cymorcin monoglucoside	9.944	328.1518	P
400	Vanillactic acid	9.944	212.0685	P
401	(*Z*)-9-Cycloheptadecen-1-one	9.982	250.2298	P
402	Acrovestone	10.149	554.2875	P
403	Hovenidulcioside B2	10.173	708.4087	P
404	19,20-DiHDPA	10.184	362.2461	P
405	Balofloxacin	10.184	389.1758	P
406	Hellebrin	10.189	724.3302	P
407	Allixin	10.190	226.1206	P
408	DU 122290	10.201	362.1652	P
409	Physagulin A	10.218	510.2623	P
410	Mycinamicin III	10.230	681.409	P
411	(1*alpha*,3*beta*,20*S*,22*R*,24*S*,25*S*)-Pubescenin	10.290	620.3559	P
412	Oleandrin	10.352	576.3299	P
413	13-Demethylspirolide C	10.366	691.4436	P
414	Drotaverine	10.380	397.2254	P
415	[6]-Gingerdiol 3,5-diacetate	10.404	380.2199	P
416	Salinomycin	10.414	750.4897	P
417	Petromyzonol	10.418	394.3081	P
418	Ganoderic acid I	10.420	532.3033	P
419	Bis(3-azidopyridinium)-1,10-decane perchlorate	10.448	380.2431	P
420	(±)-(*Z*)-2-(5-Tetradecenyl)cyclobutan one	10.490	264.2454	P
421	DG(15:0/20:1(11*Z*)/0:0)	10.491	608.5361	P
422	Perulactone B	10.492	488.2774	P
423	Dodecylbenzene	10.493	246.2347	P
424	Lucidumol A	10.506	472.3557	P
425	DG(20:3(5*Z*,8*Z*,11*Z*)/22:6(4*Z*,7*Z*,10*Z*,13*Z*,16*Z*,19*Z*)/0:0)	10.553	690.5221	P

426	Capsi-amide	10.587	269.2719	P
427	4-Nerolidylcatechol	10.597	314.2243	P
428	4-(3-Methyl-1-butenyl)-3,3',4',5-tetrahydroxystilbene	10.649	312.1363	P
429	Drospirenone	10.683	366.2195	P
430	2-Pentadecylfuran	10.834	278.2612	P
431	Enalkiren	10.846	656.429	P
432	Sorbitan stearate	10.895	430.3284	P
433	Cavipetin D	10.896	418.272	P
434	Propinol adenylate	10.896	403.0892	P
435	Sorbitan palmitate	10.896	402.298	P
436	b-Hydroxypropionyl-CoA	10.897	839.1313	P
437	Darifenacin	10.897	426.2296	P
438	(*N*-Acetylglucosaminyl)2-diphosphodolichol	11.023	1675.1119	P
439	Camptothecin	11.237	348.1106	P
440	Withanolide B	11.349	454.2697	P
441	*N*-Hexadecanoylpyrrolidine	11.388	309.3034	P
442	3'-*N*-Acetyl-4'-*O*-(10,12-octadecadienoyl)fusarochromanone	11.475	596.3826	P
443	1,2-Epoxypropane	11.506	58.0417	P
444	DG(20:5(5*Z*,8*Z*,11*Z*,14*Z*,17*Z*)/24:1(15*Z*)/0:0)	11.508	724.6031	P
445	MG(18:0/0:0/0:0)	11.508	358.3085	P
446	Tridemorph	11.536	297.3034	P
447	Ganoderic acid *beta*	11.818	500.3138	P
448	Cepagenin	11.955	446.3033	P
449	(*S*)-Rutaretin	11.964	262.0841	P
450	PC(14:0/22:5(4*Z*,7*Z*,10*Z*,13*Z*,16*Z*))	12.254	780.5546	P
451	12-Ketodeoxycholic acid	12.257	390.2774	P
452	PC(16:0/18:1(9*Z*))[*S*]	12.269	760.5852	P
453	Dioctyl hexanedioate	12.281	370.3083	P

454	Testosterone isocaproate	12.281	386.2815	P
455	Strobilurin A	12.359	258.1256	P
456	Lasonolide A	12.599	696.4242	P
457	Hericenone C	12.621	570.3923	P
458	Parishin C	12.623	728.2151	P
459	LysoPC(18:4(6*Z*,9*Z*,12*Z*,15*Z*))	12.935	516.3114	P
460	DG(14:0/20:1(11*Z*)/0:0)	13.035	594.5225	P
461	DG(14:0/22:2(13*Z*,16*Z*)/ 0:0)	13.156	620.5378	P
462	DG(18:0/20:3(5*Z*,8*Z*,11*Z*)/0:0)	13.335	646.5537	P
463	Dioncophyllinol B	13.715	379.1787	P
464	11Z-tetradecenoyl-CoA	13.979	975.2981	P
465	DG(14:0/22:1(13*Z*)/0:0)	14.066	622.5536	P
466	DG(14:1(9*Z*)/24:1(15*Z*)/0:0)	14.227	648.5691	P
467	Tridodecylamine	15.524	521.59	P
468	DG(14:0/24:1(15*Z*)/0:0)	15.696	650.585	P
469	DG(16:1(9*Z*)/24:1(15*Z*)/0:0)	15.942	676.6006	P

[a] – retention time [min]

[b] –compound detection in positive (P) or in negative (N) ionization mode.
