# Supplementary material for: Biological Properties of the Mucus and Eggs of Helix aspersa Müller as a Potential Cosmetic and Pharmaceutical Raw Material: A Preliminary Study
Source: Int J Mol Sci. 2024 Sep 15;25(18):9958. doi: 10.3390/ijms25189958 (PMC11432642; doi:10.3390/ijms25189958)
Supplement: Supplementary file 1 [file ijms-25-09958-s001.zip › Herman Anna - Table S8.pdf]

Table S8. Compounds identified in water-methanol[*] extract of lyophilized egg of organic *Helix aspersa* snail using LC-MS.

No	Metabolite	RT[a] [min]	Mass [*m/z*]	Detection mode[b]
1	Dimethyl carbonate	0.307	90.0318	N
2	2-Benzothiazolesulfonamide	0.321	213.9868	N
3	*N*-n-Hexanoylglycine methyl ester	3.465	187.1208	N
4	Ethiprole	5.804	395.9833	N
5	Zingerone	6.229	194.0945	N
6	Bismuth subsalicylate	6.709	361.9977	N
7	Eremopetasinorol	6.775	208.146	N
8	Nordihydrocapsiate	6.835	294.1832	N
9	3-Hydroxy-6,8-dimethoxy-7(11)-eremophilen-12,8-olide	7.033	310.1781	N
10	BILA 2185BS	7.042	618.3251	N
11	4-Hydroxy-5-phenyltetrahydro-1,3-oxazin-2-one	7.058	193.074	N
12	(*S,Z*)-Lyratol acetate	7.117	194.1306	N
13	3b-Allotetrahydrocorticosterone	7.120	350.2456	N
14	(3b,6b,8b,12a)-8,12-Epoxy-7(11)-eremophilene-6-angeloyloxy-8,12-dimethoxy-3-ol	7.201	394.2352	N
15	Lauryl hydrogen sulfate	7.281	266.1553	N
16	Amifloxacin	7.283	334.1428	N
17	Methotrexate	7.316	454.1731	N
18	Losartan	7.317	422.1623	N
19	L-Tyrosine methyl ester	7.346	195.0897	N
20	*N*-Undecylbenzenesulfonic acid	7.835	312.176	N
21	2-Dodecylbenzenesulfonic acid	8.160	326.1915	N
22	Sodium Tetradecyl Sulfate	8.202	294.1864	N
23	Dinoterb	8.251	240.0748	N
24	(+)-Prosopinine	8.274	313.2617	N
25	Docusate	8.341	422.2332	N
26	Kukoamine D	8.408	530.3123	N

27	(10*beta*H,11xi)-11-Hydroxy-13-nor-6-eremophilen-8-one	8.529	222.1622	N
28	Alcaftadine	8.886	307.1684	N
29	Gemfibrozil	8.959	250.1571	N
30	3-Oxochola-4,6-dien-24-oic acid	10.248	370.2507	N
31	(5b,7a,12a)-2-(3-methoxyphenyl)-2-oxoethyl ester-7,12-dihydroxy-cholan-24-oic acid	10.487	540.3448	N
32	Oleamide	10.489	281.2719	N
33	Methyl tetradecanoate	10.502	242.2248	N
34	Piritramide	10.548	430.2741	N
35	DG(18:1(11*Z*)/22:5(4*Z*,7*Z*,10*Z*,13*Z*,16*Z*)/0:0)	10.549	668.54	N
36	DG(20:3(5*Z*,8*Z*,11*Z*)/22:6(4*Z*,7*Z*,10*Z*,13*Z*,16*Z*,19*Z*)/0:0)	10.549	690.5217	N
37	MG(0:0/16:0/0:0)	10.551	330.277	N
38	Enalkiren	10.843	656.4287	N
39	3-Hydroxy-2-(4-morpholinylmethyl)estra-1,3,5(10)-trien-17-one	10.947	369.2303	N
40	Oleoylglycerone phosphate	11.167	434.2453	N
41	Butroxydim	11.271	399.2411	N
42	Adlupone	11.369	482.3395	N
43	Callystatin A	11.503	456.3255	N
44	DG(20:5(5*Z*,8*Z*,11*Z*,14*Z*,17*Z*)/24:1(15*Z*)/0:0)	11.506	724.6026	N
45	Pubesenolide	11.507	458.3047	N
46	MG(18:0/0:0/0:0)	11.508	358.3078	N
47	24-Acetyl-25-cinnamoylvulgaroside	12.067	608.3354	N
48	(3*beta*,22*E*,24*R*)-3-Hydroxyergosta-5,8,22-trien-7-one	12.502	410.318	N
1	Propionyl-L-carnitine	2.532	218.1393	P
2	5-Heptyltetrahydro-2- oxo-3-furancarboxylic acid	2.973	228.1362	P
3	Istamycin C1	3.112	431.2732	P
4	Capryloylglycine	3.152	201.1368	P
5	Sedanonic acid	3.240	210.1256	P
6	Wine lactone	3.289	166.0994	P
7	Netilmicin	3.452	475.2996	P

8	Homoarecoline	3.464	169.1104	P
9	Geranyl acetoacetate	3.837	238.157	P
10	Monomenthyl succinate	3.840	256.1676	P
11	*N*-(3-oxo-octanoyl)- homoserine lactone	3.976	241.1315	P
12	Tributyrin	4.010	302.1731	P
13	Acetyltropine	4.014	183.126	P
14	Alanyl-Valine	4.015	188.1162	P
15	Isopentenyladenine-9-*N*- glucoside	4.032	363.1912	P
16	Jasmine ketolactone	4.248	208.11	P
17	*N*-Methylmescaline	4.248	225.1366	P
18	Phlorin	4.265	288.0844	P
19	Humulinic acid A	4.285	266.1519	P
20	Octyl gallate	4.288	282.1467	P
21	Lupinate	4.369	306.1437	P
22	Triethylenemelamine	4.391	204.1128	P
23	Avenic acid A	4.392	322.1387	P
24	Amyl 2-furoate	4.394	182.0944	P
25	2-Methyl-4-pentyloxazole	4.502	153.1154	P
26	Halstoctacosanolide A	4.522	844.5359	P
27	Gentamicin C2b	4.534	463.3012	P
28	Flumetover	4.544	367.1392	P
29	(*E*)-3-decen-1-ol	4.545	156.1516	P
30	Diethofencarb	4.545	267.1472	P
31	Mukaadial	4.574	266.1522	P
32	Ethyl 3-(*N*- butylacetamido)propionate	4.645	215.1522	P
33	1,2,3-Tris(1-ethoxyethoxy)propane	4.663	308.2199	P
34	2-Hexenoylcholine	4.666	200.1648	P
35	2,2,7,7-Tetramethyl-1,6- dioxaspiro[4,4]nona-3,8-diene	4.674	180.1151	P
36	C12:1n-7	4.681	198.162	P

37	*Gamma*-CEHC	4.682	248.1411	P
38	Ruscopine	4.688	306.2046	P
39	(5*R*)-5-Hydroxyhexanoic acid	4.726	132.0787	P
40	1-Phenyl-6,7-dihydroxy- isochroman	4.726	242.0945	P
41	2,3-dihydrobenzofuran	4.726	120.0576	P
42	2-Ethylacrylylcarnitine	4.726	244.1551	P
43	2-Phenylbutyric acid	4.726	164.0839	P
44	3-Indolecarboxylic acid	4.726	253.1317	P
45	*beta*-hydroxylauric acid	4.728	216.1727	P
46	L-2-Methyltryptophan	4.775	218.1058	P
47	Alanyl-Isoleucine	4.776	202.132	P
48	Methyl 3-(2,3-dihydroxy-3-methylbutyl)-4-hydroxybenzoate	4.797	254.1156	P
49	*N*-Isobutyl-2,4,8,10,12- tetradecapentaenamide	4.800	273.2093	P
50	Pinidine	4.808	139.1363	P
51	Notoginsenoside R10	4.895	554.3467	P
52	4'-Hydroxy-3,4,5- trimethoxystilbene	4.958	286.1208	P
53	Allixin	4.958	226.1207	P
54	Methyl propionate	4.959	88.0524	P
55	Tetrahydroaldosterone- 3-glucuronide	4.960	540.2556	P
56	Pentosidine	4.966	378.2023	P
57	2-Phenylethyl *beta*-D- glucopyranoside	5.009	284.126	P
58	1,1,2-Triphenylpropane	5.016	272.1555	P
59	Cinncassiol A 19- glucoside	5.047	544.2522	P
60	Sterebin E	5.075	338.246	P
61	*Z*-Arg-Arg-NHMec	5.077	621.3047	P
62	(*S*)-3-Octanol glucoside	5.096	292.1888	P
63	13-Hydroxy-9-methoxy-10-oxo-11-octadecenoic acid	5.102	342.2396	P
64	7,8-Dihydrovomifoliol 9-[rhamnosyl-(1->6)-glucoside]	5.107	534.2679	P
65	(-)-*trans*-Carveol glucoside	5.133	314.1733	P

66	Gibberellin A105	5.133	330.1464	P
67	Glycerol 1-(5-hydroxydodecanoate)	5.227	290.2096	P
68	20-hydroxy-PGF2a	5.240	370.235	P
69	Toxin T2 tetrol	5.241	298.1419	P
70	Cyclonormammein	5.271	374.1726	P
71	Elaeokanine C	5.285	211.1572	P
72	4,11,13,15-Tetrahydroridentin B	5.291	268.1676	P
73	Tanacetol B	5.350	296.1989	P
74	Jasmolone glucoside	5.369	342.1681	P
75	NAc-FnorLRF-amide	5.369	622.3564	P
76	Triethyl citrate	5.405	276.121	P
77	Ethyl 7-epi-12-hydroxyjasmonate glucoside	5.407	416.2047	P
78	Valyl-Valine	5.425	216.1477	P
79	Sanshodiol	5.429	358.1422	P
80	AF Toxin II	5.430	324.1573	P
81	Taraxacolide 1-*O*-b-D- glucopyranoside	5.441	428.2045	P
82	Hydrocortisone succinate	5.480	462.2253	P
83	Corchoionol C 9- glucoside	5.482	386.1942	P
84	*O*-Methylsomniferine	5.498	622.266	P
85	11-Hydroxy-9-tridecenoic acid	5.514	228.1723	P
86	Isopulegone caffeate	5.532	316.1676	P
87	Convallatoxin	5.642	550.2772	P
88	Eremopetasinorol	5.654	208.1464	P
89	*N*-Jasmonoylisoleucine	5.680	323.2098	P
90	(2xi,6xi)-7-Methyl-3- methylene-1,2,6,7- octanetetrol	5.701	204.1363	P
91	Hexanal octane-1,3-diol acetal	5.705	228.2091	P
92	Glaucarubin	5.713	496.2289	P
93	2-Methylundecanal	5.727	184.1828	P
94	Blumenol C *O*- [rhamnosyl-(1->6)- glucoside]	5.739	518.273	P

95	(5*alpha*,10*alpha*)- 3,7(11)-Eudesmadien-2- one	5.766	218.167	P
96	Avocadienofuran	5.767	246.1985	P
97	2-Furanmethanol	5.768	98.0368	P
98	Volicitin	5.769	422.2766	P
99	Goshonoside F3	5.772	644.3384	P
100	Fluspirilene	5.807	475.2419	P
101	2-Hydroxymyristic Acid	5.831	244.2039	P
102	19(*R*)-hydroxy-PGE2	5.832	368.2199	P
103	Acetyllycopsamine	5.832	341.1839	P
104	Buspirone	5.836	385.2468	P
105	1-Octen-3-yl glucoside	5.844	290.1728	P
106	C14:1n-9	5.876	226.1933	P
107	Eriojaposide A	5.877	502.2417	P
108	Norerythrostachaldine	5.940	407.2658	P
109	Canavalioside	5.941	546.2678	P
110	Capsoside A	6.016	694.378	P
111	(+/-)-*N,N*-Dimethyl menthyl succinamide	6.018	168.1878	P
112	15-Acetoxyscirpene-3,4- diol 4-*O*-a-D- glucopyranoside	6.024	486.2099	P
113	20-COOH-Leukotriene B4	6.050	366.2035	P
114	Capsaicin	6.059	305.1993	P
115	Homodihydrojasmone	6.067	180.1515	P
116	Tricycloekasantal	6.067	178.1357	P
117	Lauroyl diethanolamide	6.073	287.2463	P
118	2-Hydroxyestrone	6.077	286.157	P
119	10-nitro,9*Z*,12*Z*- octadecadienoic acid	6.128	325.2254	P
120	(*Z*)-6-Nonenal	6.145	140.1201	P
121	Europine	6.148	329.1827	P
122	1-Phenyl-1,3- dodecanedione	6.179	274.1934	P
123	Penbutolol	6.179	291.2199	P

124	Marimastat	6.181	331.2123	P
125	Granisetron	6.202	312.1941	P
126	Pseudoargiopinin III	6.204	373.2098	P
127	4-Hydroxy-3-methoxy- 2,10-bisaboladien-9-one	6.206	266.188	P
128	(10*beta*H,11xi)-11- Hydroxy-13-nor-6- eremophilen-8-one	6.213	222.1619	P
129	(+)-Prosopinine	6.232	313.2617	P
130	*alpha*-Butyl-*omega*- hydroxypoly(oxyethylene) poly(oxypropylene)	6.277	248.1988	P
131	Gravelliferone	6.305	298.157	P
132	Furohyperforin	6.338	552.3818	P
133	Hyperforin	6.338	536.3873	P
134	Plantaricin BN	6.339	484.2305	P
135	Cuscohygrine	6.371	224.1889	P
136	Dihydrocapsaicin	6.387	307.2146	P
137	Metoprolol	6.393	267.1835	P
138	10-hydroperoxy-8*E*,12*Z*- octadecadienoic acid	6.443	312.2301	P
139	9-HOTE	6.501	294.2197	P
140	1-Hydroxyacorenone	6.516	250.1569	P
141	Pterosin O	6.517	232.1464	P
142	5-*O*-β-D-Mycaminosyltylonolide	6.556	597.3486	P
143	Formylfusarochromanone	6.578	320.1388	P
144	Discadenine	6.579	304.165	P
145	Armillatin	6.596	610.4238	P
146	Chaksine	6.611	450.2966	P
147	Musabalbisiane C	6.642	568.2496	P
148	10-Hydroxy-2,8- decadiene-4,6-diynoic acid	6.663	176.0475	P
149	C16 Sphinganine	6.684	273.2669	P
150	Sphinganine	6.684	301.2982	P
151	Artabsinolide A	6.689	280.1312	P
152	Fetidine	6.696	682.3275	P

153	2,4,12-Octadecatrienoic acid isobutylamide	6.708	333.3018	P
154	Glicoisoflavanone	6.713	384.1571	P
155	2-Tetradecanone	6.716	212.2142	P
156	1-Isomangostin hydrate	6.735	428.1829	P
157	5-(2,3-Dihydroxy-3- methylbutyl)-4-(3,4- epoxy-4- methylpentanoyl)-3,4- dihydroxy-2- isopentanoyl-2- cyclopenten-1-one	6.735	412.2098	P
158	Eremopetasinorone A	6.756	206.1305	P
159	1*alpha*,3*beta*,22*R*- Trihydroxyergosta-5,24*E*- dien-26-oic acid 3-*O*-b-D-glucoside 26-*O*-[b-D- glucosyl-(1->2)-b-D-glucosyl] ester	6.757	946.4733	P
160	CB3717	6.758	477.1651	P
161	Ximelagatran	6.758	473.2629	P
162	2-Methoxy-estradiol-17β 3-glucuronide	6.759	478.2179	P
163	Phytosphingosine	6.759	317.2931	P
164	Isorenieratene/ (Leprotene)	6.767	528.378	P
165	Asparagoside F	6.780	1034.529 3	P
166	Austalide A	6.780	516.2357	P
167	Mycalamide B	6.780	517.2892	P
168	Trilobolide	6.780	522.2444	P
169	Panaquinquecol 1	6.787	292.204	P
170	16-hydroxy hexadecanoic acid	6.788	272.2352	P
171	Ipecoside	6.803	565.2174	P
172	Canescein	6.804	566.2705	P
173	5-Megastigmen-7-yne- 3,9-diol 9-glucoside	6.813	370.1992	P
174	Funtumine	6.849	317.2719	P
175	2-Methyl-1-phenyl-2- propanyl acetate	6.858	192.1152	P
176	Cubebininolide	6.864	446.1942	P
177	(*S*)-Nerolidol 3-*O*-[a-L- Rhamnopyranosyl-(1->4)-a-L-rhamnopyranosyl-(1->2)-b-D-glucopyranoside]	6.865	676.367	P
178	13,14-dihydro-15-keto- PGA2	6.878	334.2144	P
179	5-Dodecyldihydro-2(3H)- furanone	6.884	254.2249	P

180	2,6-Di-tert-butyl-4-ethylphenol	6.895	234.1985	P
181	1-Methyl-2-nonyl-4(1H)- quinolinone	6.897	285.2093	P
182	Austalide F	6.898	490.22	P
183	Discodermolide	6.900	593.3913	P
184	Genipin 1-*beta*-gentiobioside	6.901	550.1902	P
185	3-Hydroxy-6,8- dimethoxy-7(11)- eremophilen-12,8-olide	6.907	310.1767	P
186	1-Tridecene	6.916	182.2036	P
187	Zizybeoside II	6.919	594.2163	P
188	Kni 102	6.934	595.2995	P
189	Kanokoside C	6.938	638.2426	P
190	Chrycolide	6.944	232.0185	P
191	4,4-Difluoropregn-5-ene- 3,20-dione	6.946	350.2069	P
192	6-Caffeoylsucrose	6.958	504.1477	P
193	Coriandrone D	6.962	352.1521	P
194	4,5-Dihydroniveusin A	6.964	396.1786	P
195	7-(4-Hydroxy-3- methoxyphenyl)-5- methoxy-1-phenyl-3-heptanone	6.967	342.1831	P
196	Arachidonyl Trifluoromethyl Ketone	6.972	356.2313	P
197	3'-Hydroxy-HT2 toxin	6.977	440.2043	P
198	Muricatacin	6.990	284.2343	P
199	Nonyl octanoate	6.993	270.2558	P
200	Coccinin	7.002	528.2571	P
201	2-Amino-7,8-dihydro-4- hydroxy-6- (diphosphooxymethyl)pteridine	7.017	355.0091	P
202	BILA 2185BS	7.042	618.3255	P
203	Cyclotetradecane	7.062	196.2192	P
204	Imidaprilat	7.084	377.1586	P
205	Glutamyl-Tryptophan	7.087	333.1323	P
206	Myxochelin A	7.088	404.1581	P
207	Proansamitocin	7.094	443.2289	P
208	Terbucarb	7.094	277.2041	P

209	6*alpha*,9-Difluoro- 11beta-hydroxypregn-4- ene-3,20-dione	7.109	366.2019	P
210	Pumiliotoxin 251D	7.113	251.225	P
211	2-Methoxyestradiol-3-methylether	7.122	316.2023	P
212	Armillaripin	7.123	414.2042	P
213	Finaconitine	7.127	630.3148	P
214	Paucin	7.177	468.1994	P
215	*Z*-Gly-Pro-Leu-Gly-Pro	7.190	573.2784	P
216	(9*Z*,11*R*,12*S*,13*S*,15*Z*)-12,13-Epoxy-11-hydroxy-9,15-octadecadienoic acid	7.225	310.2145	P
217	Armillaric acid	7.243	416.1834	P
218	Cincassiol B	7.243	400.2089	P
219	Allopumiliotoxin 267A	7.251	267.2197	P
220	*trans*-9, *trans*-11-octadecadienoic acid; C18:2n-7,9	7.253	280.2403	P
221	Ethyl (4*Z*)-4,7- octadienoate	7.285	168.1151	P
222	Bleekerine	7.318	409.1757	P
223	3-Ethyl-2-hydroxy-4- methyl-2-cyclopenten-1- one	7.328	140.0839	P
224	Lentiginosine	7.328	157.1104	P
225	Testolactone	7.330	300.1727	P
226	*N*-Dealkylatedtolterodine	7.335	283.1936	P
227	6-*trans*-LTB4	7.362	336.2303	P
228	Estrane-3α,17α-diol	7.378	278.2248	P
229	3-Methyl-*alpha*-ionyl acetate	7.380	250.1932	P
230	1,8-Heptadecadiene-4,6- diyne-3,10-diol	7.405	260.1775	P
231	Smilanippin A	7.456	724.4377	P
232	Etiocholan-3α-ol-17-one 3-glucuronide	7.457	466.2569	P
233	Picrasin C	7.461	422.2302	P
234	Austalide L	7.462	428.2198	P
235	Cyclocalopin F	7.462	294.1104	P
236	Erythroskyrin	7.462	455.2309	P
237	(4-Methylphenyl)acetaldehyde	7.463	134.0733	P

238	2,2-Dimethyl-3,4-bis(4- methoxyphenyl)-2H-1- benzopyran-7-ol acetate	7.463	430.1781	P
239	*Alpha*-Methylstyrene	7.463	118.0784	P
240	Vilazodone	7.463	441.2155	P
241	(*Z*)-13-Oxo-9-octadecenoic acid	7.492	296.2353	P
242	(*E,E*)-1,6-bis(4-methoxyphenyl)-1,5- hexadiene	7.500	294.1616	P
243	Methyl (9*Z*)-10'-oxo-6,10'-diapo-6-carotenoate	7.501	312.1729	P
244	7,10-Hexadecadienoic acid	7.527	252.209	P
245	Steviolbioside	7.585	642.3252	P
246	Dihomo-γ-linolenoyl-EA	7.596	349.2966	P
247	Piperolein B	7.598	343.2148	P
248	2-Hexadecanone	7.644	240.2454	P
249	Norpropoxyphene	7.658	325.2036	P
250	Biperiden	7.679	311.2249	P
251	Austalide B	7.722	474.2257	P
252	Mammea E/BB	7.737	430.199	P
253	1-(4-Amino-2-methylpyrimid-5-ylmethyl)-3-(*beta*hydroxyethyl)-2-methylpyridinium	7.749	259.1549	P
254	Zucchini factor B	7.767	663.4304	P
255	2,2,6,6-Tetramethyl-4-piperidinone	7.776	155.131	P
256	6,10,14-Trimethyl- 5,9,13-pentadecatrien-2-one	7.776	262.23	P
257	8-Acetoxypinoresinol 4-glucoside	7.778	578.2022	P
258	Gabapentin	7.784	171.1261	P
259	Methyl 15- cyanopentadecanoate	7.792	281.236	P
260	Methyloctatropine	7.792	282.2435	P
261	Glaucamine	7.838	385.1524	P
262	Phlegmarine	7.838	250.2406	P
263	(*E*)-3-(2-Hydroxyphenyl)-2-propenal	7.839	148.0524	P
264	Glaudine	7.840	399.1681	P
265	Methadone	7.874	309.209	P

266	Spiredine	7.885	353.1982	P
267	Dihydrodioscorine	7.904	223.1574	P
268	5-Hexyltetrahydro-2- furanoctanoic acid	7.910	298.2509	P
269	Spiroxamine	7.914	297.2672	P
270	*N-trans*-Feruloyloctopamine	7.917	329.1279	P
271	Pristanic acid	7.918	298.2872	P
272	Elaiophylin	7.943	1024.592 5	P
273	17-Methylandrosta-2,4- dieno[2,3-d]isoxazol- 17*beta*-ol	7.946	327.2197	P
274	Dodecanamide	7.959	199.1937	P
275	Santalyl acetate	7.962	262.1933	P
276	Asparagoside D	7.965	902.4872	P
277	Firocoxib	7.998	336.1034	P
278	Scopoloside II	8.003	770.4089	P
279	Stearamide	8.014	283.2876	P
280	2-Methoxyestrone 3- sulfate	8.015	380.1293	P
281	Leucomycin A9	8.017	743.4094	P
282	MG(0:0/18:1(11*Z*)/0:0)	8.019	356.2929	P
283	Corchorusoside B	8.032	682.3571	P
284	Dihydro-5-(2-octenyl)- 2(3H)-furanone	8.117	196.1463	P
285	Lymecycline	8.143	602.2576	P
286	Asteltoxin	8.152	418.199	P
287	Valsartan	8.152	435.226	P
288	Undecylprodigiosin	8.168	393.2784	P
289	2,2,7,7-Tetramethyl-1,6-dioxaspiro[4,4]non-3-ene	8.191	182.1309	P
290	17*beta*- Acetamidoandrost-4-en- 3-one	8.193	329.2353	P
291	Lyngbyatoxin	8.268	437.3045	P
292	Erinacine G	8.270	464.2422	P
293	Pipercitine	8.274	349.3329	P

294	Pipericine	8.274	335.3186	P
295	6-Oxocineole	8.275	168.1152	P
296	2-(4-Methylphenyl)-2- propanol	8.276	150.1044	P
297	p-Mentha-1,3,5,8- tetraene	8.276	132.0941	P
298	Coniine	8.277	127.1362	P
299	Santene	8.277	122.1096	P
300	Vicenistatin	8.281	500.3618	P
301	Methyl 2-octynoate	8.283	154.0993	P
302	MG(0:0/20:2(11*Z*,14*Z*)/0:0)	8.293	382.3083	P
303	Ponasteroside A	8.295	626.3667	P
304	Tributyl phosphate	8.304	266.165	P
305	Palonosetron	8.328	296.1895	P
306	*Gamma*-Taraxastane- 3,20-diol	8.330	444.3954	P
307	LysoPE(0:0/20:2(11*Z*,14*Z*))	8.330	505.3164	P
308	Isopentylideneisopentylamine	8.341	155.1677	P
309	Tropine	8.345	141.1156	P
310	2-Pentylfuran	8.358	138.1047	P
311	4-Vinylcyclohexene	8.359	108.094	P
312	Isometheptene	8.362	141.1519	P
313	Homostachydrine	8.368	158.1182	P
314	2-Decylfuran	8.370	208.1828	P
315	Flabellidine	8.388	288.2204	P
316	Kukoamine D	8.402	530.3125	P
317	Triphenyl phosphate	8.410	326.071	P
318	MK-129	8.411	367.0975	P
319	SB 221284	8.411	353.0821	P
320	(*Z*)-9-Cycloheptadecen- 1-one	8.418	250.2296	P
321	Methyprylon	8.447	183.126	P
322	Dicyclomine	8.467	309.267	P

323	Linoleoyl Ethanolamide	8.469	323.2825	P
324	Monoethyl phthalate	8.482	194.0579	P
325	Momilactone B	8.487	330.1832	P
326	12*S*-HEPE	8.509	318.2196	P
327	3L,7D,11D-phytanic acid	8.514	312.3028	P
328	Polidocanol	8.515	582.4346	P
329	Mycinamicin VIII	8.521	505.3384	P
330	Isopimara-7,15-dienol	8.524	288.2453	P
331	*N*-(14-Methylhexadecanoyl)pyrrolidine	8.524	323.3191	P
332	(3a,5b,7a,12a)-24-[(carboxymethyl)amino]- 1,12-dihydroxy-24- oxocholan-3-yl-b-D- Glucopyranosiduronic a	8.545	723.4436	P
333	8,8-Diethoxy-2,6- dimethyl-2-octanol	8.546	246.2195	P
334	Palmitoyl glucuronide	8.587	418.2928	P
335	5*beta*-Gonane	8.595	232.2192	P
336	Polysorbate 20	8.617	522.3407	P
337	Palmitoyl-EA	8.630	299.2825	P
338	Stearoylethanolamide	8.684	327.3139	P
339	LysoPC(14:0)	8.685	468.3091	P
340	Laserpitin	8.767	450.2611	P
341	Polysorbate 60	8.767	434.288	P
342	TG(8:0/8:0/8:0)	8.768	470.3587	P
343	Hexyl heptanoate	8.787	638.2361	P
344	Tecostanine	8.827	183.1625	P
345	3,4,3',4'-Tetrahydrospirilloxanthin	8.829	600.4926	P
346	Misoprostol	8.835	382.2719	P
347	9-Acetoxyfukinanolide	8.864	292.1674	P
348	13-heptadecyn-1-ol	8.882	252.2455	P
349	*N*-Methylpelletierine	8.882	155.1311	P
350	Nitramine	8.884	169.1465	P
351	β-Caryophyllene Alcohol	8.915	222.1984	P

352	MG(0:0/20:1(11*Z*)/0:0)	8.923	384.324	P
353	Tris(butoxyethyl)phosphate	8.926	398.2434	P
354	20,21,21-Trifluoro-3- methoxy-19-nor- 17*alpha*-pregna- 1,3,5(10),20-tetraen-17-ol	8.942	366.1808	P
355	Colforsin	8.963	496.3878	P
356	(3*S*,6*E*,10*E*)-1,6,10,14-Phytatetraen-3-ol	8.966	410.2307	P
357	3-Cyclohexyldodecane	9.013	252.2818	P
358	Annoglabasin F	9.020	378.241	P
359	Isoacitretin	9.039	326.1882	P
360	MG(0:0/22:5(4*Z*,7*Z*,10*Z*,13*Z*,16*Z*)/0:0)	9.054	404.2926	P
361	*N*-n-Hexanoylglycine methyl ester	9.075	187.1211	P
362	24-Hydroxycalcitriol	9.089	432.3245	P
363	*Alpha*-CEHC	9.101	278.1521	P
364	Monoisobutyl phthalic acid	9.107	222.0894	P
365	22-Oxo-docosanoate	9.135	354.3135	P
366	Anofinic acid	9.159	204.0786	P
367	MG(0:0/22:2(13*Z*,16*Z*)/0:0)	9.169	260.2351	P
368	Armillarivin	9.183	384.1937	P
369	(6*beta*,7*alpha*,12*beta*,13*beta*)-7-Hydroxy-11,16- dioxo-8,14-apianadien- 22,6-olide	9.190	384.1936	P
370	PC(22:6(4*Z*,7*Z*,10*Z*,13*Z*,16*Z*,19*Z*)/22:6(4*Z*,7*Z*,10*Z*,13*Z*,16*Z*,19*Z*))	9.200	878.5717	P
371	18-Oxocortisol	9.205	376.1885	P
372	Ampalex	9.208	241.1204	P
373	Tsangane L 3-glucoside	9.211	374.2307	P
374	Guaioxide	9.235	222.1983	P
375	Hydrocortamate	9.241	475.2955	P
376	Neogrifolin	9.258	328.2405	P
377	Gentamicin	9.269	477.314	P
378	13,14-dihydro-15-keto- PGF2α	9.288	354.2401	P
379	Kanzonol I	9.314	436.2246	P
380	Pravastatin	9.361	424.2458	P

381	Bioresmethrin	9.369	338.1882	P
382	Chloropyramine	9.370	289.1359	P
383	MG(0:0/16:0/0:0)	9.373	330.2769	P
384	Acidissiminol epoxide	9.390	409.2251	P
385	MG(0:0/22:6(4*Z*,7*Z*,10*Z*, 13*Z*,16*Z*,19*Z*)/0:0)	9.425	402.2758	P
386	(3'x,5'a,9'x,10'b)-O-(3-Hydroxy-6-oxo-7-drimen- 11-yl)umbelliferone	9.427	396.1937	P
387	9*alpha*-Fluoro- 11*beta*,16*alpha*,17*alpha*, 21-tetrahydroxypregn-4- ene-3,20-dione	9.427	396.1939	P
388	Asebotoxin II	9.427	408.2509	P
389	Ethoxysulfuron	9.432	398.0901	P
390	3'-Galloylprodelphinidin B2	9.433	762.1401	P
391	Apigenin 7-[glucuronyl- (1->2)-glucuronide] 4'- glucuronide	9.433	798.1518	P
392	Calendulaglycoside E	9.433	794.4246	P
393	(3b,6b,8b,12a)-8,12-Epoxy-7(11)- eremophilene-6- angeloyloxy-8,12- dimethoxy-3-ol	9.434	394.2359	P
394	Lilac alcohol	9.434	170.1307	P
395	Methandriol dipropionate	9.434	416.2925	P
396	Phenkapton	9.434	375.9352	P
397	[6]-Gingerdiol 3,5- diacetate	9.436	380.2201	P
398	Iriomoteolide 1a	9.446	506.3224	P
399	Lucidenic acid M	9.452	462.2961	P
400	Tsugarioside B	9.452	616.4311	P
401	3-(5,6,6-Trimethylbicyclo[2,2,1]hept-1-yl)cyclohexanol	9.460	236.2142	P
402	Cavipetin D	9.461	418.27	P
403	Lauroyl peroxide	9.475	398.3394	P
404	5-O-Desmethyldonepezil	9.492	365.1998	P
405	Erythrophleguine	9.494	449.2779	P
406	Galbanic acid	9.498	398.2074	P
407	Heliosupine	9.568	397.2115	P
408	b-D-Glucopyranosiduronic acid, (3a,5b)-24- [(carboxymethyl)amino]- 24-oxocholan-3-yl	9.589	609.3513	P
409	Piscerythramine	9.591	451.2013	P

410	2,3-Dinor-6-keto- prostaglandin F1 a	9.596	342.2049	P
411	2-(4-Chloro-3,5- dimethylphenoxy)-N-(2- phenyl-2H-benzotriazol- 5-yl)-acetamide	9.644	406.1198	P
412	Monocrotaline	9.645	325.1526	P
413	HDOPA	9.679	376.2255	P
414	Glycidyl oleate	9.696	338.2821	P
415	Ethyl abietate	9.747	330.256	P
416	Lycopersiconol	9.764	334.2505	P
417	4-Hydroxyvalsartan	9.786	451.2223	P
418	Oleoyl Ethanolamide	9.789	325.298	P
419	10,16-dihydroxy-palmitic acid	9.795	288.2299	P
420	MG(0:0/20:5(5*Z*,8*Z*,11*Z*,14*Z*,17*Z*)/0:0)	9.797	376.2612	P
421	Labienoxime	9.817	209.1781	P
422	6,8a-Seco-6,8a-deoxy-5-oxoavermectin "2a" aglycone	9.822	586.3509	P
423	MG(0:0/22:1(13*Z*)/0:0)	9.837	412.3552	P
424	1b,3a,7a,12a-Tetrahydroxy-5bcholanoic acid	9.941	424.2811	P
425	Kamahine C	9.942	268.1311	P
426	2,5-Furandicarboxylic acid	9.943	156.006	P
427	4-Carboxy-2-hydroxy-6-methoxy-6-oxohexa-2,4-dienoate	9.943	216.027	P
428	1-(3-Hydroxy-4- methoxyphenyl)-1,2- ethanediol	9.944	184.0737	P
429	Acetyl tributyl citrate	9.944	402.2257	P
430	Arbutin	9.944	272.0898	P
431	Cinitapride	9.944	402.2257	P
432	Cymorcin monoglucoside	9.944	328.1523	P
433	Vanillactic acid	9.945	212.0687	P
434	Gorgostane skeleton	10.086	412.4052	P
435	Luffariellolide	10.092	386.2819	P
436	Testosterone isocaproate	10.093	386.282	P
437	Palmitic amide	10.107	255.2564	P
438	Polypodoside C	10.122	752.4353	P

439	Integerressine	10.146	554.2884	P
440	Balofloxacin	10.182	389.1759	P
441	19,20-DiHDPA	10.184	362.2461	P
442	Hellebrin	10.189	724.3291	P
443	Physagulin A	10.219	510.2618	P
444	Telaprevir	10.230	679.4032	P
445	(1*alpha*,3*beta*,20*S*,22*R*,24*S*,25*S*)-Pubescenin	10.289	620.356	P
446	2*E*-Eicosenoic acid	10.294	310.2869	P
447	Oleandrin	10.352	576.3295	P
448	13-Demethylspirolide C	10.379	691.4437	P
449	Drotaverine	10.381	397.2253	P
450	Ganoderic acid I	10.420	532.3036	P
451	Petromyzonol	10.423	394.3079	P
452	DU 122290	10.443	362.165	P
453	Bis(3-azidopyridinium)- 1,10-decane perchlorate	10.448	380.2435	P
454	1,2-Epoxypropane	10.489	58.0418	P
455	(±)-(*Z*)-2-(5-Tetradecenyl)cyclobutan one	10.491	264.2454	P
456	DG(15:0/20:1(11*Z*)/0:0)	10.491	608.5356	P
457	Dodecylbenzene	10.491	246.235	P
458	Dodemorph	10.491	281.272	P
459	Oleamide	10.491	281.2722	P
460	Perulactone B	10.494	488.2777	P
461	Lucidumol A	10.505	472.3556	P
462	Corchoroside B	10.531	518.2886	P
463	DG(18:1(11*Z*)/22:5(4*Z*,7*Z*,10*Z*,13*Z*,16*Z*)/0:0)	10.552	668.5402	P
464	DG(20:3(5*Z*,8*Z*,11*Z*)/22: 6(4*Z*,7*Z*,10*Z*,13*Z*,16*Z*,19*Z*)/0:0)	10.553	690.5219	P
465	DG(14:0/22:4(7*Z*,10*Z*,13*Z*,16*Z*)/0:0)	10.571	616.504	P
466	Capsi-amide	10.585	269.2721	P
467	4-Nerolidylcatechol	10.596	314.2249	P

468	4-(3-Methyl-1-butenyl)-3,3',4',5-tetrahydroxystilbene	10.650	312.1359	P
469	Drospirenone	10.684	366.2195	P
470	2-Pentadecylfuran	10.834	278.2612	P
471	D-myo-Inositol-1,4,5-triphosphate	10.895	419.9627	P
472	Doripenem	10.896	420.1141	P
473	Sorbitan palmitate	10.896	402.2984	P
474	*N*-2-[4-(3,3-Dimethylallyloxy)phenyl] ethylcinnamide	10.938	335.1886	P
475	Erinacine C	10.962	434.2669	P
476	(*N*-Acetylglucosaminyl)2-diphosphodolichol	10.995	1675.113 7	P
477	Linalyl propionate	11.014	210.1619	P
478	*N*-Hexadecanoylpyrrolidine	11.190	309.3034	P
479	*Beta*-Elemonic acid	11.369	454.3444	P
480	DG(20:5(5*Z*,8*Z*,11*Z*,14*Z*,17*Z*)/24:1(15*Z*)/0:0)	11.509	724.6034	P
481	MG(18:0/0:0/0:0)	11.510	358.3085	P
482	Tridemorph	11.533	297.3033	P
483	Ethyl decanoate	11.535	200.1777	P
484	Phenethyl decanoate	11.535	276.2087	P
485	Hydrocortisone cypionate	11.547	486.2983	P
486	Glycoursodeoxycholic acid	11.614	449.3145	P
487	Solanocardinol	11.661	431.338	P
488	Campesteryl caffeate	11.674	562.4009	P
489	Ganoderic acid *beta*	11.822	500.3132	P
490	PC(14:0/18:3(6*Z*,9*Z*,12*Z*))	11.876	728.5215	P
491	Cepagenin	11.958	446.3035	P
492	3'-*N*-Acetyl-4'-*O*-(10,12-octadecadienoyl)fusarochromanone	11.968	596.3829	P
493	Corchoroside A	11.968	534.2813	P
494	Phytal	12.075	294.2926	P
495	12-Ketodeoxycholic acid	12.259	390.2772	P

496	Samin	12.280	250.0842	P
497	Dioctyl hexanedioate	12.282	370.3084	P
498	Heteratisine	12.286	391.2367	P
499	Strobilurin A	12.362	258.1258	P
500	Oolongtheanin	12.396	732.1309	P
501	Lasonolide A	12.603	696.4236	P
502	Hericenone C	12.624	570.3919	P
503	Oligomycin D	12.802	776.5036	P
504	2-Aminoethylphosphocholate	12.937	515.3038	P
505	3-*O*-*trans*- Feruloyleuscaphic acid	13.043	664.3953	P
506	DG(14:0/20:1(11*Z*)/0:0)	13.043	594.5223	P
507	Lansioside A	13.043	659.4397	P
508	PS(18:0/18:0)	13.050	791.5644	P
509	Didodecyl thiobispropanoate	13.146	514.406	P
510	DG(14:0/22:2(13*Z*,16*Z*)/ 0:0)	13.161	620.538	P
511	DG(18:0/20:3(5*Z*,8*Z*,11*Z*)/0:0)	13.338	646.5532	P
512	Dioncophyllinol B	13.719	379.1784	P
513	(3*beta*,5*alpha*,6*beta*,7*alpha*,22*E*,24*R*)-Ergosta- 8,22-diene-3,5,6,7-tetrol	13.791	446.3392	P
514	Elastin	13.824	552.3616	P
515	DG(16:0/24:1(15*Z*)/0:0)	13.973	678.6161	P
516	*N*-[(4*E*,8*E*)-1,3-dihydroxyoctadeca-4,8- dien-2- yl]hexadecanamide	13.998	535.4962	P
517	DG(14:0/22:1(13*Z*)/0:0)	14.078	622.5541	P
518	DG(14:1(9*Z*)/24:1(15*Z*)/ 0:0)	14.238	648.5697	P
519	DG(18:1(11*Z*)/22:2(13*Z*,16*Z*)/0:0)	14.422	674.5852	P
520	Tridodecylamine	15.548	521.5902	P
521	DG(14:0/24:1(15*Z*)/0:0)	15.716	650.5849	P

*- methanol:water (1;1, *v/v*)

[a] – retention time [min]

[b] –compound detection in positive (P) or in negative (N) ionization mode.
