# Supplementary material for: Biological Properties of the Mucus and Eggs of Helix aspersa Müller as a Potential Cosmetic and Pharmaceutical Raw Material: A Preliminary Study
Source: Int J Mol Sci. 2024 Sep 15;25(18):9958. doi: 10.3390/ijms25189958 (PMC11432642; doi:10.3390/ijms25189958)
Supplement: Supplementary file 1 [file ijms-25-09958-s001.zip › Herman Anna - Table S9.pdf]

Table S9. Compounds identified in acetonitrile extract of lyophilized egg of organic *Helix aspersa* snail using LC-MS.

No	Metabolite	RT[a] [min]	Mass [*m/z*]	Detection mode[b]
1	Trifluoroacetic acid	0.254	113.9929	N
2	Dulcitol	0.256	182.0792	N
3	2-C-Methyl-D-erythritol-4-phosphate	0.258	216.0383	N
4	3-Deoxyarabinohexonic acid	0.261	180.0636	N
5	Norfuraneol	0.261	114.0312	N
6	Acrylic acid	0.262	72.0212	N
7	3-Hydroxy-3-methylglutaric acid	0.263	162.0527	N
8	Spirodiclofen	0.263	410.1039	N
9	Quinoline-3-carboxamides	0.268	359.1059	N
10	3-b-Galactopyranosylglucose	0.270	342.1162	N
11	Vinyl caffeate	0.272	206.0581	N
12	2,4,6-Octatriynoic acid	0.273	132.0210	N
13	Glucosereductone	0.281	88.0159	N
14	*N*-Acryloylglycine	0.288	129.0428	N
15	2-Naphthalenethiol	0.292	160.0351	N
16	Dimethyl carbonate	0.302	90.0317	N
17	Methylpyrazine	0.367	94.0532	N
18	*Trans*-Aconitate	0.375	174.0165	N
19	Dexpanthenol	0.859	205.1312	N
20	p-Hydroxyphenylacetic acid	1.523	152.0473	N
21	*N*-n-Hexanoylglycine methyl ester	3.470	187.1210	N
22	Methyl *N*-(amethylbutyryl)glycine	4.299	188.1050	N
23	Blumenol C glucoside	5.726	372.2151	N
24	Ethiprole	5.805	395.9835	N
25	Zingerone	6.231	194.0944	N
26	Bismuth subsalicylate	6.710	361.9978	N

27	Eremopetasinorol	6.778	208.1465	N
28	Nordihydrocapsiate	6.836	294.1832	N
29	3-Hydroxy-6,8-dimethoxy-7(11)-eremophilen-12,8-olide	7.036	310.1781	N
30	BILA 2185BS	7.042	618.3249	N
31	4-Hydroxy-5-phenyltetrahydro-1,3-oxazin-2-one	7.059	193.0742	N
32	Flupropanate	7.091	145.9992	N
33	3b-Allotetrahydrocorticosterone	7.121	350.2455	N
34	(3b,6b,8b,12a)-8,12-Epoxy-7(11)-eremophilene-6-angeloyloxy-8,12-dimethoxy-3-ol	7.201	394.2354	N
35	4'-Methoxymucidin	7.280	288.1366	N
36	Lauryl hydrogen sulfate	7.280	266.1552	N
37	Zanthodioline	7.281	305.1268	N
38	Chromanol 293B	7.282	324.1132	N
39	Losartan	7.317	422.1624	N
40	Methotrexate	7.317	454.1732	N
41	L-Tyrosine methyl ester	7.345	195.0897	N
42	*N*-Methyl-14-Odemethylepiporphyroxine	7.461	371.1367	N
43	*N*-Undecylbenzenesulfonic acid	7.722	312.1760	N
44	Chaparrinone	7.782	378.1687	N
45	Ethyl 2-hydroxy-3-(3-indolyl)propanoate glucoside	7.783	395.1588	N
46	Latifoline	8.023	393.1794	N
47	2-Dodecylbenzenesulfonic acid	8.159	326.1914	N
48	Sodium Tetradecyl Sulfate	8.201	294.1865	N
49	Dihomo-γ-linolenoyl-EA	8.224	349.2981	N
50	Dinoterb	8.252	240.0747	N
51	Docusate	8.341	422.2329	N
52	Kukoamine D	8.407	530.3121	N
53	Alcaftadine	8.889	307.1681	N
54	2,4-di-tert-butylphenol	8.960	204.1514	N
55	Furmecyclox	9.283	251.1520	N

56	3-Oxochola-4,6-dien-24-oic Acid	10.248	370.2510	N
57	(5b,7a,12a)-2-(3-methoxyphenyl)-2-oxoethyl ester-7,12-dihydroxy-cholan-24-oic acid	10.296	540.3441	N
58	Enalkiren	10.848	656.4284	N
59	Tridecanoic acid, 4,8,12-trimethyl-; 4,8,12-Trimethyltridecanoic acid	11.136	256.2403	N
60	Methadyl Acetate	11.272	353.2355	N
61	Adlupone	11.365	482.3391	N
62	Drotaverine	11.449	397.2254	N
63	Butroxydim	11.516	399.2410	N
64	(3*beta*,22*E*,24*R*)-3-Hydroxyergosta-5,8,22-trien-7-one	12.503	410.3182	N
1	L-2-Amino-3-(1-pyrazolyl)propanoic acid	0.254	155.0696	P
2	2-Amino-2-methyl-1,3- propanediol	0.255	105.0790	P
3	3-Hydroxyisoheptanoic acid	0.256	146.0944	P
4	Trolamine	0.256	149.1052	P
5	Dulcitol	0.257	182.0791	P
6	Choline chloride	0.258	103.0998	P
7	Ethyl propionate	0.258	102.0681	P
8	Isocaproic acid	0.258	116.0837	P
9	3-Deoxyarabinohexonic acid	0.260	180.0636	P
10	5,5',6,6'-Tetrahydroxy- 3,3'-biindolyl	0.269	296.0804	P
11	Lysine	0.272	146.1061	P
12	Prothiocarb	0.272	190.1131	P
13	(2*R*,4*S*)-2,4-Diaminopentanoate	0.273	132.0903	P
14	5-Aminopentanamide	0.273	116.0953	P
15	Asp-Phe	0.273	280.1052	P
16	4,5-Dihydropiperlonguminine	0.274	275.1530	P
17	*Beta*-Alaninamide	0.274	88.0633	P
18	5-Methoxytryptophol	0.275	191.0950	P
19	Asparaginyl- Phenylalanine	0.277	279.1227	P
20	L-phenylalanyl-L- hydroxyproline	0.277	278.1274	P

21	Homoagmatine	0.280	144.1377	P
22	Juzirine	0.281	281.1048	P
23	8-Hydroxypurine	0.283	138.0547	P
24	Maleic hydrazide	0.286	112.0275	P
25	Phenethicillin	0.298	364.1083	P
26	Isoleucyl-Serine	0.363	218.1274	P
27	Purine	0.367	120.0436	P
28	2-(Methylthio)-3H- phenoxazin-3-one	0.368	243.0364	P
29	Pheneturide	0.368	206.1066	P
30	Aminocaproic acid	0.414	131.0949	P
31	*R*-2-Hydroxy-3- methylbutanoic acid 3- Methylbutanoyl	0.657	202.1206	P
32	3-Hydroxysuberic acid	0.731	190.0840	P
33	Polypropylene glycol (m w 1,200-3,000)	0.851	134.0944	P
34	(3-Phenylpropionyl)glycine methyl ester	0.858	221.1052	P
35	Dexpanthenol	0.858	205.1315	P
36	2,5-Dihydro-2,4,5- trimethyloxazole	1.506	113.0841	P
37	3-[(3-Methylbutyl)nitrosoamino]-2-butanone	2.020	186.1369	P
38	Homoarecoline	2.023	169.1103	P
39	Amyl 2-furoate	2.060	182.0943	P
40	Retronecine	2.398	155.0947	P
41	*N*-Valerylglycine methyl ester	2.402	173.1053	P
42	Propionyl-L-carnitine	2.505	218.1393	P
43	Octylamine	2.570	129.1518	P
44	DL-2-amino-octanoic acid	2.581	159.1260	P
45	1,11-Undecanedicarboxylic acid	3.098	244.1674	P
46	5-Heptyltetrahydro-2-oxo-3-furancarboxylic acid	3.136	228.1363	P
47	Capryloylglycine	3.154	201.1366	P
48	E-64	3.158	357.2003	P
49	Tranexamic acid	3.219	157.1103	P

50	Sedanonic acid	3.239	210.1255	P
51	*N*-n-Hexanoylglycine methyl ester	3.469	187.1208	P
52	2,3-Dimethyl-2-cyclohexen-1-one	3.470	124.0886	P
53	γ-Aminobutyryl-lysine	3.471	231.1582	P
54	Istamycin C1	3.588	431.2733	P
55	Neryl glucoside	3.665	316.1884	P
56	Netilmicin	3.737	475.2995	P
57	Monomenthyl succinate	3.852	256.1675	P
58	Geranyl acetoacetate	3.853	238.1571	P
59	Arginyl-Isoleucine	3.868	287.1968	P
60	2-Methyl-1-phenyl-2-propanyl acetate	3.925	192.1150	P
61	*N*-(3-oxo-octanoyl)-homoserine lactone	3.985	241.1316	P
62	Tributyrin	4.019	302.1733	P
63	Acetyltropine	4.023	183.1260	P
64	Toxin T2 tetrol	4.186	298.1416	P
65	*N*-Methylmescaline	4.258	225.1366	P
66	2,2,7,7-Tetramethyl-1,6-dioxaspiro[4.4]nona-3,8-diene	4.299	180.1153	P
67	PE(18:4(6*Z*,9*Z*,12*Z*,15*Z*)/22:6(4*Z*,7*Z*,10*Z*,13*Z*,16*Z*,19*Z*))	4.452	783.4830	P
68	1-Octen-3-yl glucoside	4.501	290.1727	P
69	*N*-[(Ethoxycarbonyl)methyl)-p-menthane-3-carboxamide	4.506	269.1994	P
70	Mycobactin S	4.526	827.5087	P
71	(*E*)-3-decen-1-ol	4.550	156.1515	P
72	Diethofencarb	4.552	267.1471	P
73	Flumetover	4.553	367.1390	P
74	Ethyl 3-(Nbutylacetamido)propionate	4.649	215.1522	P
75	1,2,3-Tris(1-ethoxyethoxy)propane	4.673	308.2200	P
76	1-Methyl-2-propylbenzene	4.680	134.1095	P
77	Aspergillic acid	4.681	224.1526	P
78	*Gamma*-CEHC	4.686	248.1413	P

79	C12:1n-7	4.687	198.1620	P
80	Humulinic acid A	4.687	266.1517	P
81	11-Hydroxy-9-tridecenoic acid	4.696	228.1722	P
82	2-Hydroxymyristic Acid	4.716	244.2038	P
83	1-Phenyl-6,7-dihydroxyisochroman	4.730	242.0940	P
84	2-Ethylacrylylcarnitine	4.731	244.1550	P
85	2-Phenylbutyric acid	4.732	164.0839	P
86	*Beta*-hydroxylauric acid	4.733	216.1727	P
87	4-(*N*Maleimido)phenyltrimethylammonium	4.740	231.1138	P
88	Alanyl-Isoleucine	4.780	202.1317	P
89	Methyl 7-epi-12-hydroxyjasmonate glucoside	4.794	402.1891	P
90	*N*-Isobutyl-2,4,8,10,12-tetradecapentaenamide	4.803	273.2095	P
91	Gravolenic acid	4.807	280.0950	P
92	(*E*)-3-(2-Hydroxyphenyl)-2-propenal	4.830	148.0526	P
93	Methyl 3-(2,3-dihydroxy-3-methylbutyl)-4-hydroxybenzoate	4.831	254.1157	P
94	Pinidine	4.847	139.1364	P
95	1,3-Diphenyltetramethyldisiloxane	4.964	286.1209	P
96	2,3-dihydrobenzofuran	4.964	120.0576	P
97	Jasmine ketolactone	4.964	208.1101	P
98	(7'*R*)-(+)-Lyoniresinol 9'-glucoside	4.992	582.2339	P
99	Selegiline	4.993	187.1364	P
100	2-Phenylethyl *beta*-Dglucopyranoside	5.013	284.1260	P
101	1,1,2-Triphenylpropane	5.023	272.1561	P
102	Pentosidine	5.039	378.2010	P
103	(*S*)-3-Octanol glucoside	5.100	292.1884	P
104	Decylubiquinol	5.104	324.2299	P

105	(-)-*trans*-Carveol glucoside	5.137	314.1730	P
106	Gibberellin A105	5.138	330.1464	P
107	Ethyl undecanoate	5.167	214.1933	P
108	Glycerol 1-(5-hydroxydodecanoate)	5.233	290.2095	P
109	PG(16:0/18:0)	5.271	750.5371	P
110	Cyclonormammein	5.275	374.1727	P
111	Elaeokanine C	5.289	211.1574	P
112	Jasmolone glucoside	5.373	342.1679	P
113	Asteltoxin	5.387	418.1992	P
114	Gravacridonetriol glucoside	5.414	519.1721	P
115	Hydrocortisone succinate	5.482	462.2253	P
116	Corchoionol C 9- glucoside	5.484	386.1941	P
117	Isopulegone caffeate	5.527	316.1677	P
118	Terazosin	5.651	387.1896	P
119	Eremopetasinorol	5.658	208.1466	P
120	(2xi,6xi)-7-Methyl-3- methylene-1,2,6,7- octanetetrol	5.701	204.1362	P
121	2-Methylundecanal	5.729	184.1828	P
122	Blumenol C *O*- [rhamnosyl-(1->6)- glucoside]	5.743	518.2722	P
123	Tracheloside	5.747	550.2064	P

124	2-Methoxyestradiol-3- methylether	5.760	316.2023	P
125	Avocadienofuran	5.771	246.1985	P
126	Volicitin	5.771	422.2766	P
127	(5*alpha*,10*alpha*)- 3,7(11)-Eudesmadien-2- one	5.772	218.1671	P
128	NAc-FnorLRF-amide	5.775	622.3569	P
129	3-Nonyl-1H-pyrazole	5.802	194.1786	P
130	Fluspirilene	5.808	475.2417	P
131	Glaucamine	5.861	385.1525	P
132	Sanshodiol	5.861	358.1419	P
133	Glaudine	5.863	399.1683	P
134	2-Decylfuran	5.876	208.1827	P
135	C14:1n-9	5.879	226.1934	P
136	Eriojaposide A	5.879	502.2413	P
137	Canavalioside	5.942	546.2679	P
138	(+/-)-*N,N*-Dimethyl menthyl succinamide	6.019	168.1879	P
139	Capsoside A	6.022	694.3777	P
140	3*beta*-Hydroxypregn-5-ene	6.025	302.2611	P
141	15-Acetoxyscirpene-3,4- diol 4-*O*-a-D- glucopyranoside	6.028	486.2105	P
142	Homodihydrojasmone	6.071	180.1515	P

143	Lauroyl diethanolamide	6.075	287.2462	P
144	2-Hydroxyestrone	6.081	286.1571	P
145	(+)-Prosopinine	6.138	313.2619	P
146	(*Z*)-6-Nonenal	6.152	140.1203	P
147	Methyl tetradecanoate	6.212	242.2245	P
148	4-Hydroxy-3-methoxy- 2,10-bisaboladien-9-one	6.215	266.1881	P
149	Proglumide	6.233	334.1900	P
150	Gravelliferone	6.309	298.1571	P
151	Phenethyl decanoate	6.326	276.2090	P
152	Eucalyptol	6.338	154.1358	P
153	*alpha*-Butyl-*omega*- hydroxypoly(oxyethylene) poly(oxypropylene)	6.359	248.1989	P
154	1,1-Diethoxy-2-hexene	6.360	172.1465	P
155	Cuscohygrine	6.375	224.1889	P
156	3-Oxopregn-4-ene- 20*beta*-carboxaldehyde dioxime	6.503	358.2621	P
157	Guaioxide	6.503	222.1983	P
158	5-*O*-β-D-Mycaminosyltylonolide	6.549	597.3501	P
159	Momilactone B	6.573	330.1831	P
160	Formylfusarochromanone	6.582	320.1388	P
161	Chaksine	6.611	450.2967	P

162	10-Hydroxy-2,8-decadiene-4,6-diynoic acid	6.667	176.0474	P
163	Monoisobutyl phthalic acid	6.667	222.0894	P
164	C16 Sphinganine	6.686	273.2672	P
165	Sphinganine	6.686	301.2983	P
166	AF Toxin II	6.699	324.1576	P
167	2-Furanmethanol	6.700	98.0367	P
168	Dihydrocapsaicin	6.706	307.2150	P
169	2,4,12-Octadecatrienoic acid isobutylamide	6.708	333.3016	P
170	Glicoisoflavanone	6.715	384.1578	P
171	2-Tetradecanone	6.719	212.2142	P
172	5-(2,3-Dihydroxy-3-methylbutyl)-4-(3,4-epoxy-4-methylpentanoyl)-3,4-dihydroxy-2-isopentanoyl-2-cyclopenten-1-one	6.736	412.2100	P
173	1-Isomangostin hydrate	6.737	428.1834	P
174	Tigloidine	6.758	223.1572	P
175	Ximelagatran	6.760	473.2626	P
176	Phytosphingosine	6.761	317.2929	P
177	Funtumine	6.762	317.2718	P
178	Isorenieratene/(Leprotene)	6.770	528.3770	P
179	Cycrimine	6.777	287.2245	P
180	Mycalamide B	6.783	517.2889	P

181	Trilobolide	6.783	522.2446	P
182	Erysothiopine	6.786	407.1024	P
183	Porson	6.786	386.1732	P
184	Cinegalline	6.788	430.2103	P
185	16-hydroxy hexadecanoic acid	6.792	272.2351	P
186	Canescein	6.806	566.2702	P
187	2-Pentadecanone	6.859	226.2298	P
188	5-Dodecyldihydro-2(3H)- furanone	6.886	254.2250	P
189	1-Methyl-2-nonyl-4(1H)- quinolinone	6.895	285.2095	P
190	Pumiliotoxin 251D	6.898	251.2251	P
191	1-Tridecene	6.918	182.2033	P
192	2-Hexadecanone	6.934	240.2455	P
193	Chrycolide	6.945	232.0184	P
194	4,5-Dihydroniveusin A	6.966	396.1780	P
195	(10*S*)-Juvenile hormone III diol phosphate	6.968	364.1649	P
196	3'-Hydroxy-HT2 toxin	6.977	440.2041	P
197	Plantaricin BN	6.991	484.2313	P
198	Muricatacin	6.996	284.2351	P
199	Coccinin	7.003	528.2568	P

200	Acetyl Tyrosine Ethyl Ester	7.030	251.1158	P
201	BILA 2185BS	7.045	618.3255	P
202	Phosphoric acid	7.045	97.9768	P
203	Cyclotetradecane	7.063	196.2191	P
204	Xylopinine	7.086	355.1766	P
205	Osajin	7.091	404.1607	P
206	Terbucarb	7.096	277.2043	P
207	6,10,14-Trimethyl- 5,9,13-pentadecatrien-2-one	7.103	262.2301	P
208	2,2-Dimethyl-3,4-bis(4- methoxyphenyl)-2H-1- benzopyran-7-ol acetate	7.125	430.1777	P
209	DHAP(18:0)	7.126	436.2598	P
210	Finaconitine	7.127	630.3157	P
211	1-Pentadecene	7.144	210.2346	P
212	Nonyl octanoate	7.150	270.2562	P
213	10,16-dihydroxy-palmitic acid	7.194	288.2302	P
214	*cis*-5- Tetradecenoylcarnitine	7.200	370.2968	P
215	Armillaric acid	7.244	416.1831	P
216	Cincassiol B	7.245	400.2098	P
217	Penbutolol	7.251	291.2195	P
218	Allopumiliotoxin 267A	7.252	267.2198	P

219	*trans*-9, *trans*-11-octadecadienoic acid; C18:2n-7,9	7.254	280.2403	P
220	4'-Methoxymucidin	7.274	288.1365	P
221	Panaquinquecol 1	7.298	292.2041	P
222	Bleekerine	7.320	409.1758	P
223	17*beta*- Acetamidoandrost-4-en- 3-one	7.376	329.2354	P
224	3-Methyl-*alpha*-ionyl acetate	7.385	250.1928	P
225	*N*-Dealkylatedtolterodine	7.385	283.1937	P
226	Arachidonic Acid (d8)	7.397	312.2890	P
227	Physagulin C	7.440	542.2509	P
228	(4-Methylphenyl)acetaldehyde	7.463	134.0730	P
229	Armillaripin	7.463	414.2045	P
230	Artabsinolide A	7.463	280.1313	P
231	Austalide L	7.463	428.2200	P
232	Cyclocalopin F	7.463	294.1103	P
233	Erythroskyrin	7.463	455.2307	P
234	*alpha*-Methylstyrene	7.464	118.0782	P
235	(3'x,5'a,9'x,10'b)-*O*-(3-Hydroxy-6-oxo-7-drimen-11-yl)umbelliferone	7.466	396.1937	P
236	Methyl (9*Z*)-10'-oxo-6,10'-diapo-6-carotenoate	7.503	312.1723	P
237	Norpropoxyphene	7.517	325.2037	P

238	2,4,12-Octadecatrienoic acid piperidide	7.553	345.3031	P
239	Piperolein B	7.678	343.2147	P
240	Biperiden	7.679	311.2254	P
241	3-(10-Heptadecenyl)phenol	7.709	330.2921	P
242	Zucchini factor B	7.768	663.4311	P
243	Glycosides	7.778	584.2845	P
244	p-Hydroxyphenethyl *trans*-ferulate	7.783	314.1158	P
245	Methyl 15-cyanopentadecanoate	7.792	281.2357	P
246	Phlegmarine	7.837	250.2411	P
247	Methadone	7.876	309.2088	P
248	Methyloctatropine	7.882	282.2437	P
249	Pristanic acid	7.920	298.2872	P
250	Elaiophylin	7.945	1024.5924	P
251	Dodecanamide	7.963	199.1937	P
252	Asparagoside D	7.966	902.4881	P
253	Scopoloside II	8.003	770.4090	P
254	17-Methylandrosta-2,4- dieno[2,3-d]isoxazol- 17*beta*-ol	8.008	327.2194	P
255	Leucomycin A9	8.019	743.4091	P
256	MG(0:0/18:1(11*Z*)/0:0)	8.020	356.2928	P

257	Corchorusoside B	8.035	682.3562	P
258	Convallatoxin	8.094	550.2776	P
259	Lymecycline	8.145	602.2576	P
260	Undecylprodigiosin	8.169	393.2780	P
261	Armillane	8.170	420.2156	P
262	UDP-*N*-acetyl-D- mannosamine	8.231	607.0767	P
263	Erinacine G	8.267	464.2409	P
264	Hydrocortisone cypionate	8.268	486.2974	P
265	Lyngbyatoxin	8.269	437.3043	P
266	Pipericine	8.278	335.3173	P
267	MG(0:0/20:2(11*Z*,14*Z*)/0:0)	8.293	382.3082	P
268	Epimetendiol	8.303	266.1648	P
269	Tributyl phosphate	8.306	266.1650	P
270	1-Phenyl-1,3- dodecanedione	8.315	274.1932	P
271	DG(22:5(7*Z*,10*Z*,13*Z*,16*Z*,19*Z*)/14:0/0:0)	8.325	614.4914	P
272	Estrane-3α,17α-diol	8.325	278.2247	P
273	Palonosetron	8.328	296.1887	P
274	Lentiginosine	8.343	157.1104	P
275	Spirilloxanthin	8.343	596.4620	P

276	Ethyl (4*Z*)-4,7-octadienoate	8.348	168.1152	P
277	Flabellidine	8.386	288.2206	P
278	5-Methyl-2-phenyl-2- hexenal	8.387	188.1202	P
279	Bipindogulomethyloside	8.402	552.2935	P
280	Kukoamine D	8.402	530.3125	P
281	Methyl 2*E*,4*Z*- hexadecadienoate	8.404	266.2250	P
282	B 823-08	8.410	353.0821	P
283	Clavamycin B	8.411	362.1422	P
284	Triphenyl phosphate	8.411	326.0711	P
285	Methyprylon	8.452	183.1260	P
286	Methyl 2-octynoate	8.454	154.0994	P
287	Dicyclomine	8.467	309.2667	P
288	3L,7D,11D-phytanic acid	8.515	312.3027	P
289	Polidocanol	8.515	582.4341	P
290	*N*-(14-Methylhexadecanoyl)pyrrolidine	8.525	323.3188	P
291	Palmitoyl-EA	8.536	299.2827	P
292	8,8-Diethoxy-2,6-dimethyl-2-octanol	8.545	246.2196	P
293	(3a,5b,7a,12a)-24-[(carboxymethyl)amino]-1,12-dihydroxy-24-oxocholan-3-yl-b-Dglucopyranosiduronic a	8.546	641.3413	P
294	Oleyl alcohol	8.561	268.2768	P

295	Palmitoyl glucuronide	8.589	418.2933	P
296	Vaccenyl carnitine	8.605	425.3507	P
297	Polysorbate 20	8.616	522.3409	P
298	Dodemorph	8.630	281.2720	P
299	Oleamide	8.631	281.2722	P
300	Isopimara-7,15-dienol	8.679	288.2454	P
301	Stearoylethanolamide	8.682	327.3137	P
302	LysoPC(14:0)	8.686	468.3090	P
303	Spiroxamine	8.743	297.2670	P
304	Laserpitin	8.768	450.2615	P
305	Polysorbate 60	8.768	434.2878	P
306	Hexyl heptanoate	8.788	638.2363	P
307	Tecostanine	8.825	183.1621	P
308	9-Acetoxyfukinanolide	8.865	292.1674	P
309	*N*-Methylpelletierine	8.880	155.1311	P
310	MG(0:0/20:1(11*Z*)/0:0)	8.923	384.3243	P
311	Tris(butoxyethyl)phosphate	8.928	398.2438	P
312	Manzamine A	8.981	548.3517	P
313	Phytal	8.991	294.2924	P

314	3-Cyclohexyldodecane	9.013	252.2818	P
315	(*E,E*)-1,6-bis(4-methoxyphenyl)-1,5-hexadiene	9.038	294.1621	P
316	Isoacitretin	9.039	326.1884	P
317	24-Hydroxycalcitriol	9.090	432.3242	P
318	(*E*)-1-[4-Hydroxy-3-(3-methyl-1,3-butadienyl)phenyl]-2-(3,5-dihydroxyphenyl)ethylene	9.109	294.1259	P
319	*Alpha*-CEHC	9.110	278.1520	P
320	22-Oxo-docosanoate	9.133	354.3135	P
321	(*Z*)-13-Oxo-9-octadecenoic acid	9.147	296.2352	P
322	Anofinic acid	9.161	204.0788	P
323	MG(0:0/22:2(13*Z*,16*Z*)/0:0)	9.168	410.3397	P
324	18-Oxocortisol	9.205	376.1884	P
325	Misoprostol	9.207	382.2704	P
326	Gentamicin	9.266	477.3157	P
327	Linoleoyl Ethanolamide	9.288	323.2826	P
328	10-Eicosene	9.326	280.3133	P
329	Pravastatin	9.363	424.2461	P
330	Chloropyramine	9.368	289.1359	P
331	Bioresmethrin	9.369	338.1884	P
332	MG(0:0/16:0/0:0)	9.373	330.2772	P

333	[6]-Gingerdiol 3,5-diacetate	9.421	380.2201	P
334	PE(14:0/18:3(6*Z*,9*Z*,12*Z*))	9.429	685.4671	P
335	Calendulaglycoside E	9.431	794.4238	P
336	(3b,6b,8b,12a)-8,12-Epoxy-7(11)-eremophilene-6-angeloyloxy-8,12-dimethoxy-3-ol	9.432	394.2357	P
337	(*E*)-3-(2-Hydroxyphenyl)-2-propenal	9.432	148.0525	P
338	Lilac alcohol	9.432	170.1308	P
339	Methandriol dipropionate	9.433	416.2913	P
340	MG(0:0/22:6(4*Z*,7*Z*,10*Z*,13*Z*,16*Z*,19*Z*)/0:0)	9.433	402.2756	P
341	Nogalonic acid	9.433	382.0701	P
342	Quazepam	9.434	386.0274	P
343	Iriomoteolide 1a	9.444	506.3225	P
344	3-(5,6,6-Trimethylbicyclo[2.2.1]hept-1-yl)cyclohexanol	9.456	236.2142	P
345	MG(0:0/18:3(6*Z*,9*Z*,12*Z*)/0:0)	9.468	352.2614	P
346	5,10-Pentadecadien-1-ol	9.509	224.2141	P
347	Piscerythramine	9.592	451.2011	P
348	2-(4-Chloro-3,5-dimethylphenoxy)-*N*-(2-phenyl-2H-benzotriazol-5-yl)-acetamide	9.642	406.1201	P
349	4*beta*-(2-Aminoethylthio)catechin	9.643	365.0926	P
350	Monocrotaline	9.644	325.1530	P
351	Glycidyl oleate	9.698	338.2818	P

352	Lycopersiconol	9.766	334.2504	P
353	6,8a-Seco-6,8a-deoxy-5-oxoavermectin "2a" aglycone	9.819	586.3508	P
354	MG(0:0/22:1(13*Z*)/0:0)	9.833	412.3555	P
355	1-(3-Hydroxy-4-methoxyphenyl)-1,2-ethanediol	9.942	184.0737	P
356	4-Carboxy-2-hydroxy-6-methoxy-6-oxohexa-2,4-dienoate	9.942	216.0271	P
357	Arbutin	9.942	272.0896	P
358	Vanillactic acid	9.942	212.0684	P
359	2,3-Dinor-6-ketoprostaglandin F1 a	9.943	342.2042	P
360	2,5-Furandicarboxylic acid	9.943	156.0060	P
361	Acetyl tributyl citrate	9.943	402.2250	P
362	Cymorcin monoglucoside	9.943	328.1524	P
363	Kamahine C	9.943	268.1311	P
364	1b,3a,7a,12a-Tetrahydroxy-5bcholanoic acid	9.944	424.2809	P
365	(*Z*)-9-Cycloheptadecen-1-one	9.980	250.2296	P
366	3*beta*-(1-Pyrrolidinyl)-5*alpha*-pregnane-11,20-dione	10.078	385.2985	P
367	Balofloxacin	10.196	389.1758	P
368	Tamoxifen	10.198	371.2250	P
369	DU 122290	10.200	362.1651	P
370	Palmitic amide	10.212	255.2562	P

371	*S*-Octyl GSH	10.380	419.2084	P
372	Bis(3-azidopyridinium)-1,10-decane perchlorate	10.493	380.2433	P
373	(9*Z*,11*E*,13*E*,15*Z*)-4-Oxo-9,11,13,15-octadecatetraenoic acid	10.564	290.1882	P
374	4-Methoxycinnamic acid	10.564	178.0634	P
375	Capsi-amide	10.582	269.2717	P
376	Drospirenone	10.683	366.2197	P
377	Enalkiren	10.849	656.4291	P
378	b-Hydroxypropionyl-CoA	10.895	839.1325	P
379	Cavipetin D	10.895	418.2720	P
380	D-myo-Inositol-1,4,5-triphosphate	10.895	419.9622	P
381	1*alpha*-O-Methylquassin	10.896	404.2185	P
382	Sorbitan palmitate	10.896	402.2983	P
383	Darifenacin	10.897	426.2301	P
384	Militarinone C	10.915	423.2414	P
385	Stearamide	11.218	283.2875	P
386	Nandrolone phenpropionate	12.254	406.2507	P
387	12-Ketodeoxycholic acid	12.257	390.2774	P
388	PC(16:0/18:1(9*Z*))[*S*]	12.269	760.5863	P
389	Dioctyl hexanedioate	12.281	370.3085	P

390	Testosterone isocaproate	12.282	386.2818	P
391	Parishin C	12.625	728.2150	P
392	Didodecyl thiobispropanoate	13.16	514.4056	P
393	Nonanoyl-CoA	13.248	907.2354	P
394	*Beta*-Citraurol	13.662	434.3186	P
395	5,6-Dihydro-5,6-dihydroxy-y,y-carotene	13.816	572.4576	P
396	Elastin	13.817	552.3611	P

[a] – retention time [min]

[b] –compound detection in positive (P) or in negative (N) ionization mode.
